# Introduction and characterization of a novel Cu(ii)-based quaternary deep eutectic solvent and its application in the efficient synthesis of triazoles and tetrazoles under mild conditions as an inexpensive, reusable, benign, and dual solvent/catalyst medium†

**DOI:** 10.1039/d4ra08090d

**Published:** 2025-02-03

**Authors:** Laleh Golestanifar, Ali Reza Sardarian

**Affiliations:** a Chemistry Department, College of Science, Shiraz University Shiraz 71946-84795 Iran sardarian@shirazu.ac.ir +98-36460788 +98-71-36137107

## Abstract

Deep eutectic solvents consist of hydrogen bond donor and acceptor components. They are a new type of ionic liquids, and they have attracted the attention of many chemists in recent years. In this work, a quaternary deep eutectic solvent (QDES) was prepared using choline chloride, glycerol, l-arginine, and copper acetate. Its physicochemical properties were determined by Fourier transform infrared spectroscopy (FT-IR), thermal analysis (TGA), differential scanning calorimetry (DSC), hydrogen potential (pH), cyclic voltammetry (CV), viscosity, density, refractive index, ionic conductivity and spectrophotometer ultraviolet-visible (UV-Vis). Further, as a novel benign solvent/catalyst for the synthesis of 1,4-disubstituted-1,2,3-triazole, 4-substituted-1*H*-1,2,3-triazole, and 5-substituted-1*H*-tetrazole derivatives were used in a click reaction strategy. The special features of this method include mild conditions, a non-toxic environment, short reaction time, easy operation, biodegradability, deep eutectic solvent/catalyst recovery, access to cheaper raw materials, and environmental compatibility.

## Abstract 2

A novel quaternary deep eutectic solvent, ChCl/Glyce/L-Arg/Cu(OAc)_2_, as a solvent/catalyst in the synthesis of 1,2,3-triazoles and 5-substituted-1*H*-tetrazoles.

## Graphical abstract

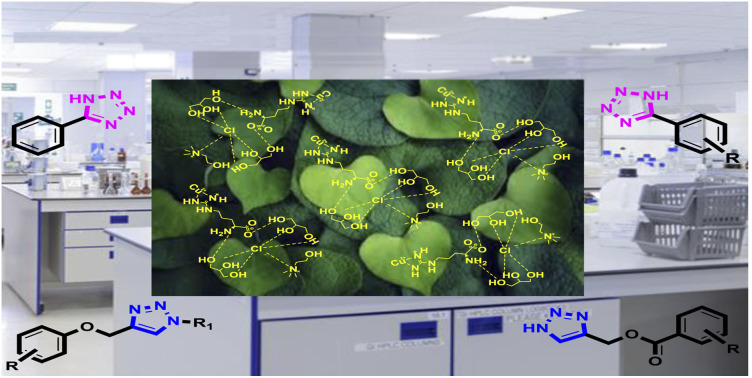

## 1. Introduction

Deep eutectic solvents refer to liquids close to the eutectic composition of mixtures (the molar ratio of components that produces the lowest melting point). They are a new alternative to ionic liquid (IL) analogs, which are now widely used and known as green counterparts of toxic organic solvents in organic transformations.^1^ In general, DESs, in contrast to ILs that are formed *via* a multistep strategy, can be prepared in one step with complete atom economy by simple mixing and heating of their components.^1^ Therefore, their preparation is consistent with green chemistry rules, environmentally and economically.^2^ In recent years, attention to green chemistry and using natural solvents and catalysts, have increased to preserve the environment. Heterocyclic chemicals have been evaluated as a lead constituent in medicinal chemistry. Because they are also copious in biopolymers such as enzymes, natural products, vitamins, and biologically active chemicals. Among heterocyclic materials, triazoles and tetrazoles have occupied a unique place. 1,2,3-Triazoles have many applications in industry, agriculture, and medicinal chemistry due to their stability in oxidation-reduction and acid-base hydrolysis. The large dipole moment of 1,2,3-triazoles causes these compounds to act as hydrogen bond acceptors that are useful for binding to biological targets and improving solubility.^3,4^ The medicinal properties of these compounds include anticancer, anti-allergy, anti-tuberculosis, anti-virus, anti-malaria, anti-AIDS, and antibiotics, and some of these compounds are now available in the market as drugs or are in clinical trials.^5,6^ The conventional methods for the synthesis of 1,2,3-triazoles are carried out using click reactions, which were reported by Sharpless and co-workers in 2001 to accelerate the synthesis of quasi-pharmaceutical molecules *via* azide–alkyne [1, 3]-dipolar cycloaddition.^7^ One of the click reactions is the copper-catalyzed azide–alkyne cycloaddition (CuAAC) reaction using various raw materials such as phenols, carboxylic acids, amines, amides, acetophenones to provide active acetylene substrates that produce 1,2,3-triazoles.^8–13^ Also, tetrazole derivatives are used in various pharmaceutical fields such as anti-arrhythmic, anti-diabetic, anti-fungal, anti-allergic, anti-AIDS and neurological diseases, and have been used in such a way that they are also present in the structure of the famous drug Losartan.^14,15^ The synthesis of tetrazoles is done using click reactions, through cyclization reaction [3 + 2] between an azide and a nitrile.^16–18^ The reported syntheses of 1,2,3-triazoles and 1*H*-tetrazoles have been are associated with pitfalls, such as the use of expensive and toxic metals, strong Lewis acids, and the *in situ* production of hydrazoic acid with a toxic and explosive nature.^14,19–21^ To avoid the use of toxic organic solvents and also expensive and harmful catalysts required in multi-step synthesis and purification for the formation of 1,2,3-triazoles and 1*H*-tetrazoles, herein, a novel QDES is designed to be prepared by employing safe, available, and inexpensive compounds such as choline chloride, glycerol, l-arginine and copper(ii) acetate and used in the green production of the desired triazoles and tetrazoles.

## 2. Experimental section

### 2.1. Materials and instrumentation

All pure chemical materials were purchased from Sigma-Aldrich and Merck. The progress of the reactions was monitored using thin-layer chromatography (TLC) on silica gel Polygram SILG/UV-254 plates. Physical and spectral information such as melting point, FT-IR, and NMR data were analyzed, along with available literature data, to characterize the obtained products. Bucci B-545 B.V.CHI apparatus was used to measure the melting point of the products. Fourier transform infrared (FT-IR) spectra were performed using a Shimadzu FT-IR 8300 spectrophotometer. The ^1^H and ^13^C NMR spectra were recorded using a Bruker Avance 400 (400 MHz) in DMSO-*d*_6_ and CDCl_3_. A typical sample was used to investigate the electrochemical behavior of ChCl/Glyce/L-Arg/Cu(ii) (1 : 2 : 0.1 : 0.03 mmol). A silver wire and a platinum electrode were utilized as the reference electrode and the working and counter electrode, respectively, along with a computer-controlled AUTOLAB (model AUT84490) for electrochemical analysis. Thermogravimetric analysis was performed using a TGA Q600 instrument under an argon atmosphere with a heating rate of 10 °C min^−1^. Differential scanning calorimeter (DSC Q600) was used to study the phase transition of the mixture ChCl/Glyce/L-Arg/Cu(ii) (1 : 2 : 0.1 : 0.03 mmol) at a heating rate of 10 °C min^−1^ under an argon atmosphere. The conductivity of this DES was measured by Metrohm (SWISS MADE, model 644 conductivity meter). The viscosity was measured with a rheometric viscometer (Anton Paar, model MCR302), and the density was measured with a digital tube densitometer (Anton Paar, model DMA4000). Refractive index values were determined using equipment from A. Kruss Optronic GmbH. The UV-Vis absorption spectrum was recorded using an ultraviolet-visible spectrophotometer (UV-Vis PerkinElmer, Lambda 25). The pH of the DES was measured with a pH meter (Metrohm, model 780).

### 2.2. General procedure

#### 2.2.1. Synthesis of [ChCl][Glyce]_2_[L-Arg]_0.1_[Cu(OAc)_2_]_0.03_

**Scheme 1:**
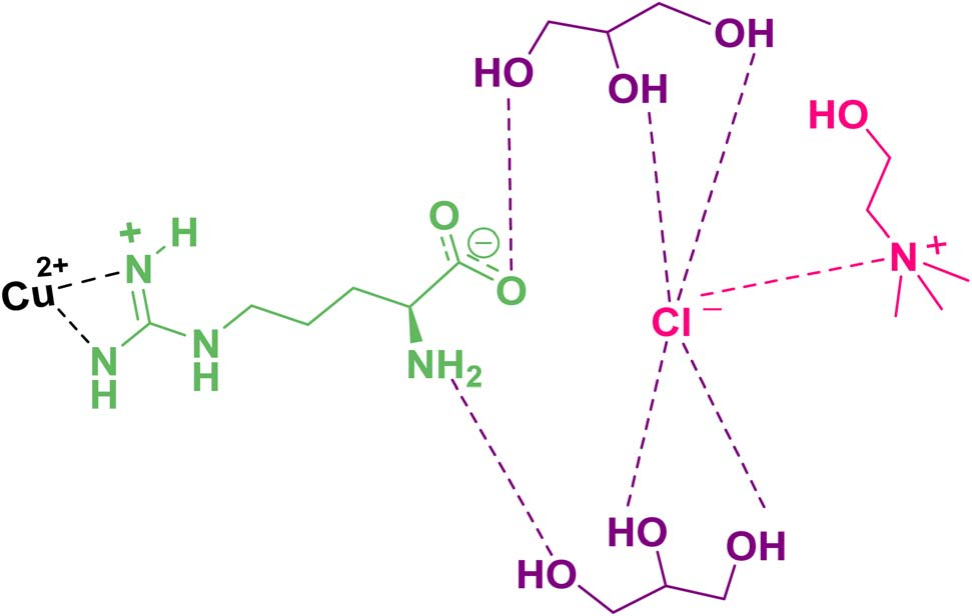
Schematic proposed structure for [ChCl][Glyce]_2_[L-Arg]_0.1_[Cu(OAc)_2_]_0.03_.

A mixture of choline chloride (1 mmol) and glycerol (2 mmol) was stirred at 80 °C for 1 h. After adding l-arginine (0.1 mmol), the resulting mixture was blended for an extra hr at the same temperature. Then, by adding copper acetate (0.03 mmol) to the mixture and stirring at the same temperature, a greenish transparent homogeneous liquid was created, which can be used as a new solvent/catalyst medium after cooling at room temperature. The following pictorial presentation might be suggested for this system (Scheme 1).

#### 2.2.2. General procedure for preparing acetylene derivatives

**Scheme 2:**
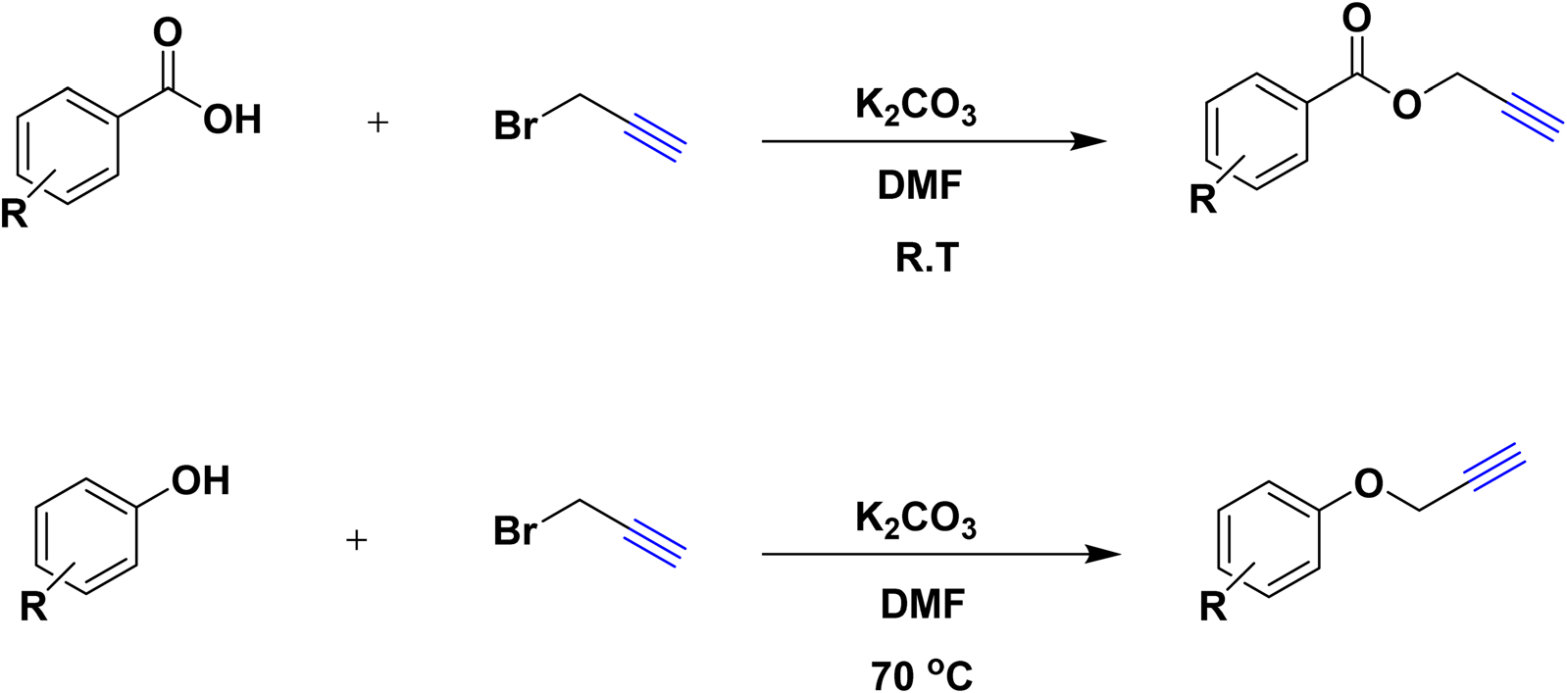
Preparing acetylene derivatives.

In a 10 mL flask, 1.2 mmol of carboxylic acid or phenol derivative, 2 mmol of K_2_CO_3,_ and 1 mmol of propargyl bromide were stirred in DMF (5 mL) for the appropriate time at room temperature (for carboxylic acids) and at 70 °C (for phenols). After completion of the experiment, a mixture of distilled water and ethyl acetate with a volume ratio of 1 : 3 was used to extract the product into the contents of the flask. The merged organic phase was dried over Mg_2_SO_4_ and filtered. The concentration of filtrate under reduced pressure provides the corresponding pure acetylene product (Scheme 2).

#### 2.2.3. General procedure for preparation of 1,2,3-triazole derivatives (1–20a)

1 mmol of acetylene derivative and 1.17 mmol of NaN_3_ in 3 mL of [ChCl][Glyce]_2_[L-Arg]_0.1_[Cu(OAc)_2_]_0.03_ was mixed with stirring at 60 °C. After completion of the reaction (checked by TLC), the reaction mixture was cooled at room temperature. Then, to extract the desired product, distilled water and chloroform were added to the reaction mixture with a volume ratio of 1 : 2, and the organic layer was filtered through anhydrous Mg_2_SO_4_ and dried under reduced pressure to obtain the crude triazole product. Finally, the desired pure triazole product was obtained through recrystallization in *n*-hexane/acetone.

#### 2.2.4. General procedure for preparation of 5-substituted-1*H*-tetrazole derivatives (21–30a)

First, sodium azide (0.09 g, 1.4 mmol) was stirred in [ChCl][Glyce]_2_[L-Arg]_0.1_[Cu(OAc)_2_]_0.03_ (2 mL) at room temperature until a clear solution was formed, then benzonitrile (1 mmol) was added to this mixture with stirring. The resultant blend was continuously stirred at 110 °C and monitored using TLC. After the completion of the reaction, the reaction mixture was cooled to room temperature, and 5 mL of 5 M HCl solution was added to it. The organic mixture was extracted with ethyl acetate (2 × 5 mL). The combined organic layer was dried by anhydrous Mg_2_SO_4_ and concentrated under ambient temperature to obtain a solid crystal. The product was purified by simple recrystallization from aqueous ethanol to provide the related pure 5-substituted 1*H*-tetrazoles.

## 3. Results and discussions

### 3.1. [ChCl][Glyce]_2_[L-Arg]_0.1_[Cu(OAc)_2_]_0.03_ characterizations

#### 3.1.1. FT-IR

**Fig. 1:**
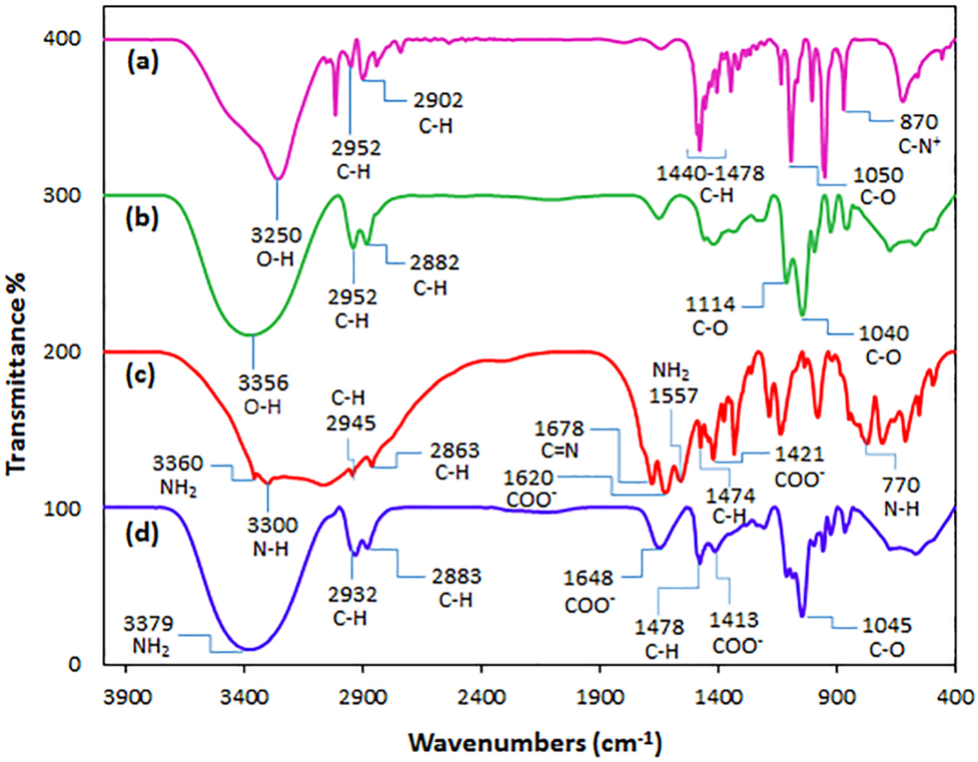
FT-IR spectra of (a) choline chloride, (b) glycerol, (c) l-arginine, and (d) the ChCl/Glyce/L-Arg/Cu (OAc)_2_ DES.

FT-IR spectra of choline chloride (a), glycerol (b), l-arginine (c), and ChCl/Glyce/L-Arg/Cu(ii) (d) are displayed in Fig. 1. In the FT-IR spectrum of choline chloride (Fig. 1a), the stretching vibrations associated with O–H, CH_2_, and CH_3_ groups were assigned to the region of 3000–3250 cm^−1^, the bending vibrations of CH_2_ and CH_3_ were transferred to the area of 1440–1478 cm^−1^. Stretching vibrations C–O at 1050 cm^−1^ and symmetrical bending C–N^+^ at 870 cm^−1^ were observed. For glycerol (Fig. 1b), the 3356 cm^−1^, 2952 cm^−1^, and 2882 cm^−1^ absorbance peaks are allocated to the O–H and C–H stretching vibrations, respectively. The regions 1114 cm^−1^ and 1040 cm^−1^ have belonged to the stretching vibrations of C–O. In l-arginine (Fig. 1c), the stretching vibrations of the NH_2_ (amino, guanidinium) and N–H (guanidinium) groups appear in the 3360 cm^−1^ and 3300 cm^−1^. The regions 2945 cm^−1^ and 2863 cm^−1^ are related to the stretching vibration groups CH_2_ and C–H. The stretching vibration C=N (guanidinium) and asymmetrical stretching COO^−^ bands are seen in 1678 cm^−1^ and 1620 cm^−1^, respectively. The related peaks of scissoring NH_2_ (amino) and CH_2_ groups are seen at 1557 cm^−1^ and 1474 cm^−1^. The absorbance peak at 1421 cm^−1^ is related to the symmetrical stretching vibration COO^−^, and the out-of-plane bending absorption peak of N–H occurred at 770 cm^−1^.^22^ For [ChCl][Glyce]_2_[L-Arg]_0.1_[Cu(OAc)_2_]_0.03_ (Fig. 1d), the stretching vibrations of NH_2_ (amino, guanidinium) groups appeared at 3379 cm^−1^. Absorption in the regions of 2932 cm^−1^ and 2883 cm^−1^ was attributed to the stretching vibrations of CH_2_ and C–H groups in the mixture, and the asymmetric stretching vibration of the COO^−^ group appeared in the region of 1648 cm^−1^. The scissor bending absorption of CH_2_ was observed in the region of 11 478 cm^−1^, and the absorption that occurred in the region of 1413 cm^−1^ is related to the symmetric stretching vibration of COO^−^. Also, symmetrical and asymmetrical stretching vibrations of Cu–N should be monitored at 430 cm^−1^ and 444 cm^−1^, but these areas could not be shown in the used device.^22^

#### 3.1.2. Cyclic voltammetry (CV)

**Fig. 2:**
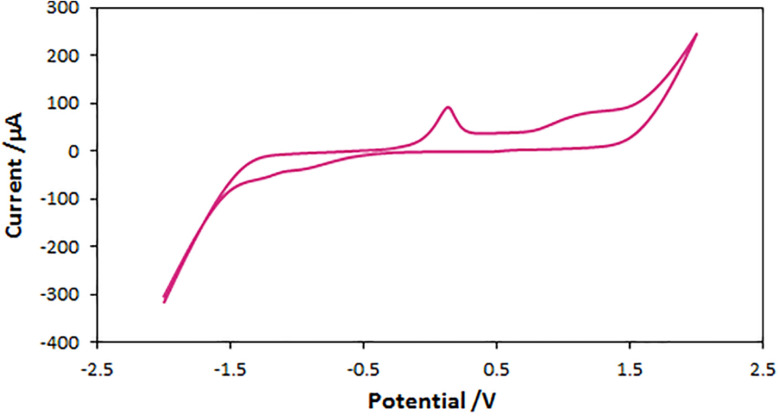
Cyclic voltammogram of the ChCl/Glyce/L-Arg/Cu (OAc)_2_ DES with a scanning speed of 50 mV s^−1^. The working electrode (Pt), reference electrode (Ag wire), and counter electrode (Pt).

To investigate the stability of QDES against oxidation-reduction, a cyclic voltammogram of the ChCl/Glyce/L-Arg/Cu(OAc)_2_ system was recorded (Fig. 2). The oxidation–reduction potential range for the silver reference electrode is from −2.5 to +2.5 V, and the scan speed is 50 mV s^−1^. There is an irreversible oxidation-reduction cyclic voltammogram with a distinct oxidation potential at ∼+0.148 V relative to the silver reference electrode for the corresponding solvent/catalyst.

#### 3.1.3. Differential scanning calorimetry (DSC)

**Fig. 3:**
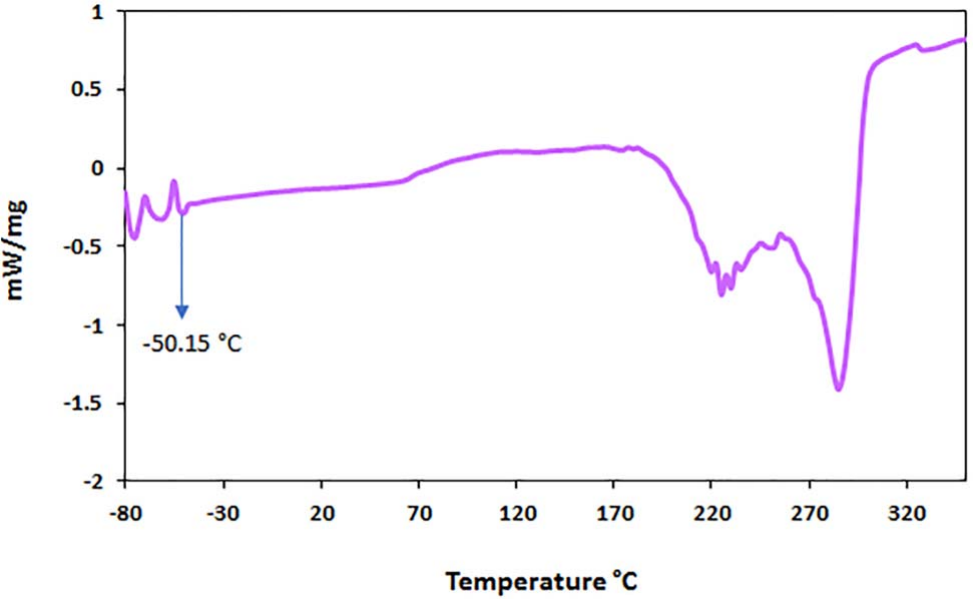
DSC diagram of the ChCl/Glyce/L-Arg/Cu (OAc)_2_ system with a heating rate of 10 °C min^−1^.

According to the melting point of choline chloride (302 °C), glycerol (17.8 °C), l-arginine (222 °C), and copper(ii) acetate (116 °C) to determine the eutectic point of the ChCl/Glyce/L-Arg/Cu (OAc)_2_ DSC analysis was performed in the temperature range of −80 °C to +350 °C (Fig. 3). As can be seen, the current DES shows a glass transition temperature in the range of −50.15 °C in its thermogram. According to the obtained thermogram, it can be said that due to the amorphous state of these DES, only the glass transition temperature is determined for them. This type of solvent/catalyst can be considered a liquid-state supramolecule because many hydrogen bonds have a stable liquid state in their structure. Therefore, the phase diagram does not specify their melting point.^23^ Appearance of two broad minimum on DSC diagram at 220–390 °C are allocated to decomposition of the QDES, which are in agreement with the TGA data.

#### 3.1.4. TGA/DTA

**Fig. 4:**
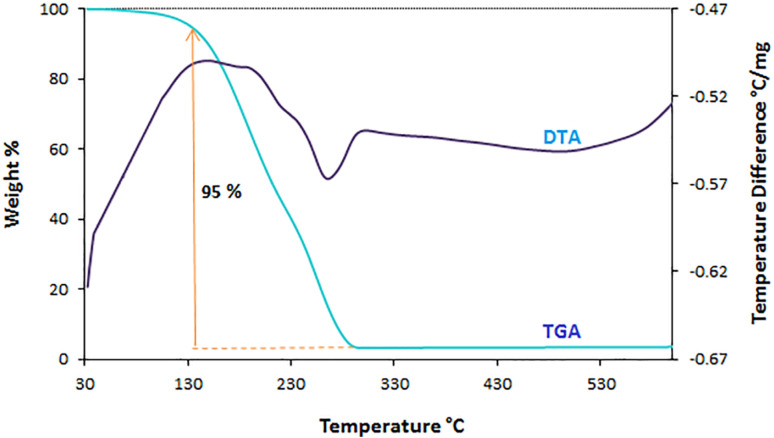
TGA and DTA for the ChCl/Glyce/L-Arg/Cu (OAc)_2_ system with a heating rate of 10 °C min^−1^.

According to the thermal decomposition of ChCl/Glyce (1 : 2 mmol, 225 °C) and ChCl/Glyce/L-Arg (1 : 2 : 0.1 mmol, 160 °C), for the solvent/catalyst [ChCl][Glyce]_2_[L-Arg]_0.1_[Cu (OAc)_2_]_0.03_, thermal gravimetric analysis and differential thermal analysis were performed at 30–400 °C (Fig. 4). The weight loss occurred in the temperature range of 130–250 °C was assigned to the complete decomposition of DES and its related components. With the increase in the chain length of the [ChCl][Glyce]_2_[L-Arg]_0.1_[Cu (OAc)_2_]_0.03_ DES, followed by the reduction of ionic interactions and the creation of more hydrogen bonds between the components, its stability decreases. As a result, it starts to degrade at a lower temperature.^23,24^

#### 3.1.5. Ionic conductivity (*K*)

Ionic conductivity measurement of [ChCl][Glyce]_2_[L-Arg]_0.1_[Cu(OAc)_2_]_0.03_ was carried out at 25 °C revealed the higher ionic conductivity (0.523 mS cm^−1^) than the electrical conductivity of [ChCl][Glyce]_2_ (1.05 mS cm^−1^ at 25 °C)^25^ and [ChCl][Glyce]_2_[L-Arg]_0.1_ (0.462 mS cm^−1^ at 23 °C), which are its precursors. This fact can be explained by the ionic character Cu(OAc)_2_.

#### 3.1.6. Viscosity (*μ*)

**Fig. 5:**
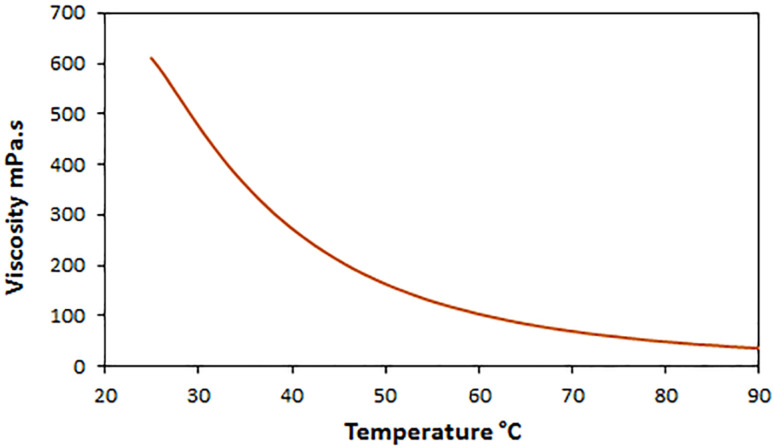
Viscosity diagram for the ChCl/Glyce/L-Arg/Cu(OAc)_2_ at different temperatures.

The viscosity value for [ChCl][Glyce]_2_[L-Arg]_0.1_[Cu(OAc)_2_]_0.03_ was measured at a shear rate of 50 s^−1^ at different temperatures and found to be 610/85 mPa s at 25 °C (Fig. 5). Regarding the higher viscosity value of [ChCl][Glyce]_2_[L-Arg]_0.1_ (658.00 mPa s).^24^ The fluidity of [ChCl][Glyce]_2_[L-Arg]_0.1_[Cu(OAc)_2_]_0.03_ appears to be increased relative to [ChCl][Glyce]_2_[L-Arg]_0.1_, which may be due to the formation of the Cu(OAc)_2_ complex with the guanidinium l-arginine functional group (Scheme 1) and as a result reorganization in the hydrogen bond network and shortening of the DES chain with increasing temperature, the mobility of molecules increases by reducing intermolecular forces (Fig. 5).

**Fig. 6:**
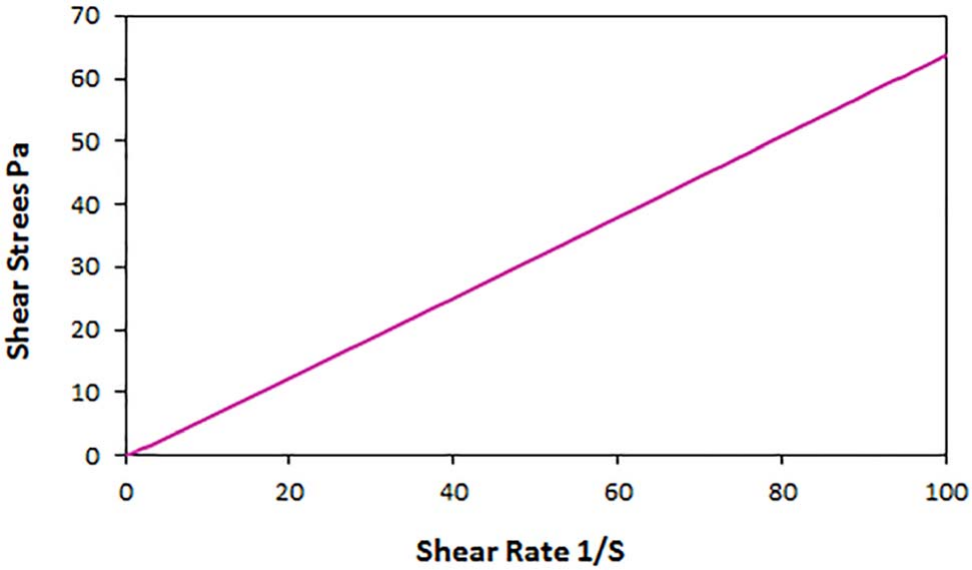
Shear stress in terms of shear rate for the ChCl/Glyce/L-Arg/Cu(OAc)_2_ fluid at 25 °C.

Also, to determine whether the ChCl/Glyce/L-Arg/Cu(OAc)_2_ fluid is Newtonian or non-Newtonian at a constant temperature (25 °C), its shear stress was measured based on the shear rate. The linear graph shows that the desired solvent/catalyst system is a Newtonian fluid (Fig. 6).

#### 3.1.7. Density (*ρ*)

Concerning the density of [ChCl] [Glyce]_2_[L-Arg]_0.1_[Cu (OAc)_2_]_0.03_ DES (1.225 g cm^−3^) in comparison to the density of [ChCl][Glyce]_2_ (1.1908 g cm^−3^), it can be concluded that the addition of l-arginine and copper acetate, which have a higher density than the density of [ChCl][Glyce]_2_ increases the density of the desired DES.^24^

#### 3.1.8. Refractive index (nD)

The value of the refractive index for the ChCl/Glyce/L-Arg/Cu (OAc)_2_ DES at 25 °C was measured as 1.7994. According to the available sources and the refractive index of [ChCl] [Glyce]_2_ (1.4868 at 25 °C)^24^ and the refractive index of copper acetate (1.545), it can be concluded that with the increase of l-arginine and copper acetate followed by an increase in the density of the ChCl/Glyce/L-Arg/Cu(OAc)_2_, the collision of light with the molecule increased and the result refractive index increased.

#### 3.1.9. Ultraviolet-visible spectrophotometer (UV-Vis)

**Fig. 7:**
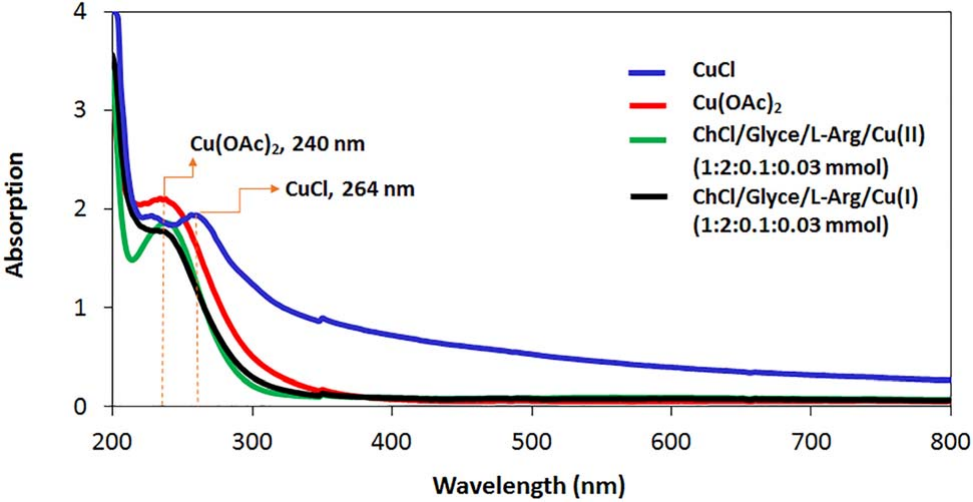
UV-visible absorption spectra for ChCl/Glyce/L-Arg/Cu(i), ChCl/Glyce/L-Arg/Cu (OAc)_2_, Cu(OAc)_2_ and CuCl in H_2_O.

UV-visible absorption spectra for the ChCl/Glyce/L-Arg/Cu(i), the ChCl/Glyce/L-Arg/Cu(ii), Cu(OAc)_2,_ and CuCl in H_2_O solvent were studied by UV-Vis spectrophotometer (Fig. 7). For the solvent/catalyst ChCl/Glyce/L-Arg/Cu(ii), the absorption peak around 240 nm can be allocated to the π → π* transition from the orbitals of the ligands to the vacant d orbital of copper +2 ions.^26^ Also, this peak confirms that the valence of copper remains +2 after complexation on the ligands. It should be mentioned that it seems that copper +1 is also oxidized to copper +2 in the presence of ligands, and this issue could be seen by its color change from green to blue, so it was decided copper acetate use in the solvent/catalyst structure.

**Fig. 8:**
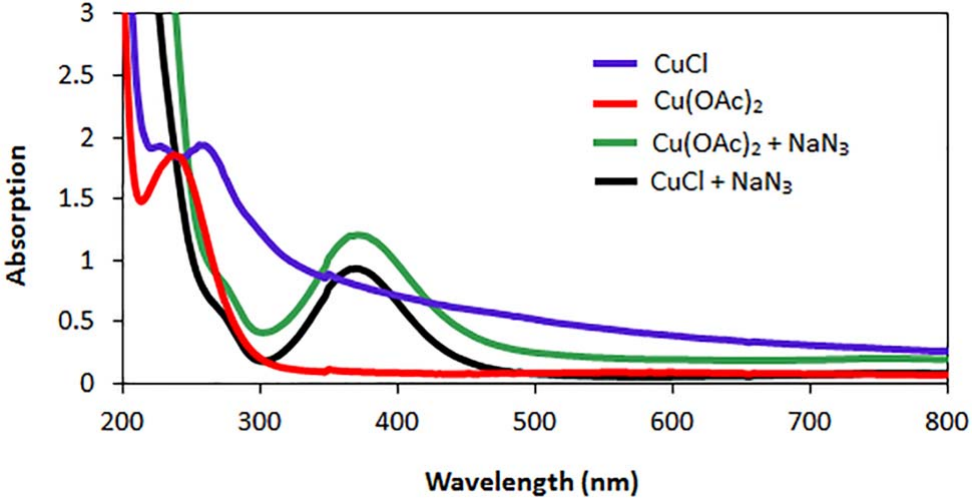
Ultraviolet-visible absorption spectra for aqueous solution CuCl, Cu(OAc)_2_, NaN_3_/Cu(OAc)_2_ and NaN_3_/CuCl.

Based on UV-vis absorptions for copper(i) chloride (290 nm) and copper(ii) acetate (280 nm),^27,28^ UV-Vis analysis for aqueous solution CuCl, Cu(OAc)_2_, NaN_3_/Cu(OAc)_2_ and NaN_3_/CuCl were performed and showed a broad absorption in the region of 300–400 nm which disclose the reduction of Cu(ii) to Cu(i) (Fig. 8).

#### 3.1.10. Hydrogen potential (pH)

First, an aqueous solution of 0.01 M of the ChCl/Glyce/L-Arg/Cu (OAc)_2_ was prepared and its pH was measured and determined to be 8.62 using a pH meter electrode. This study disclosed weaker basicity of the ChCl/Glyce/L-Arg/Cu (OAc)_2_, in comparison to the pH of the ChCl/Glyce/L-Arg (9.50), which can be due to involving the guanidine part of DES in complexation with copper acetate.

### 3.2. Evaluation of the catalytic activity of the ChCl/Glyce/L-Arg/Cu(OAc)_2_ in the preparation of 1*H*-1,2,3-triazole derivatives (1–20a)

To evaluate and optimize the ChCl/Glyce/L-Arg/Cu(OAc)_2_ as a green and new solvent/catalyst medium for the preparation of 1*H*-1,2,3-triazole derivatives, the reaction between phenylacetylene and sodium azide was taken as the model reaction. Initially, the reaction was taken in 3 mL of the prepared DES, and the expected triazole was formed in 80% yield after 3 h stirring at ambient temperature (Table 1, entry 1). Regarding dependency of the efficiency reaction to temperature, confirmed by TLC monitoring, repetition of the model reaction at 60 °C generated the desired triazole in 95% yield in 1 h (Table 1, entry 2). The efficiency of the model reaction was brought down when 1 and 2 mL of the DES were applied, so the yield of the title product reduced to 70% and 80% in the longer reaction times, 3 h and 1.5 h, separately (Table 1, entries 3 and 4). Enhancement of the molar ratio of Cu(OAc)_2_ in the DES structure, from 0.03 to 0.04 and 0.05, did not display any improvement in the efficiency of the reaction, and 4-phenyl-1*H*-triazole was formed in both cases, in excellent yield (95%) (Table 1, entries 5 and 6). When the reaction was performed in the absence of the ChCl/Glyce/L-Arg/Cu (OAc)_2_ DES, on the basis of TLC checking, all of the starting material remained intact, which is an indication of the critical role DES as a catalyst (Table 1, entry 7). For considering the effect of Cu(OAc)_2_ and [ChCl][Glyce]_2_[L-Arg]_0.1_ as the components of the desired DES, the reaction phenylacetylene with sodium azide was taken in the presence of these precursors separable. It should be noted that although the model reaction proceeded by Cu(OAc)_2_ in 20% yield at 60 °C after 6 h (Table 1, entry 8), most of the starting material remained unconsumed when the reaction was performed in [ChCl][Glyce]_2_[L-Arg]_0.1_ (Table 1, entry 9). These findings proved the synergic effect of these two precursors when they were combined and also the solvent/catalyst dual role [ChCl][Glyce]_2_[L-Arg]_0.1_[Cu(OAc)_2_]_0.03_. Based on the information gathered in Table 1, using 1 mmol phenylacetylene and 1.17 mmol sodium azide in 3 mL of [ChCl][Glyce]_2_[L-Arg]_0.1_[Cu(OAc)_2_]_0.03_ at 60 °C was selected as the optimized conditions for the providing of 1*H*-1,2,3-triazoles.

**Table 1:** Optimizing reaction conditions for the synthesis of 1*H*-1,2,3-triazole derivatives^a^

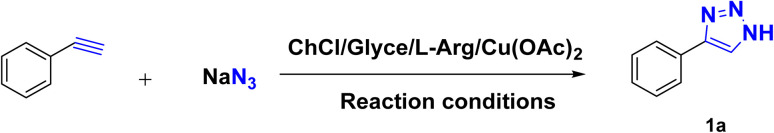						
Entry	Reaction media	Molar ratio	Catalyst amount (mL)	Temperature (°C)	Time (h)	Yield^b^ (%)
1	ChCl/Glyce/L-Arg/Cu(OAc)_2_	1 : 2 : 0.1 : 0.03	3	R.T	3	80
**2**	**ChCl/Glyce/L-Arg/Cu(OAc)**_**2**_	**1** : **2** : **0.1** : **0.03**	**3**	**60**	**1**	**95**
3	ChCl/Glyce/L-Arg/Cu(OAc)_2_	1 : 2 : 0.1 : 0.03	1	60	3	70
4	ChCl/Glyce/L-Arg/Cu(OAc)_2_	1 : 2 : 0.1 : 0.03	2	60	1.5	83
5	ChCl/Glyce/L-Arg/Cu(OAc)_2_	1 : 2 : 0.1 : 0.04	3	60	1	95
6	ChCl/Glyce/L-Arg/Cu(OAc)_2_	1 : 2 : 0.1 : 0.05	3	60	1	95
7	—	—	—	60	10	—
8	Cu(OAc)_2_	0.03	—	60	6	20
9	ChCl/Glyce/L-Arg	1 : 2 : 0.1	3	60	8	<5

a Reaction conditions: phenylacetylene (1 mmol) and sodium azide (1.17 mmol).

b Yield refers to the net products isolated.

After optimizing the reaction conditions, the click reaction strategy between phenylacetylene and the phenylacetylenes prepared from carboxylic acids and phenols with sodium azide was elected to investigate the efficiency and effectiveness of the ChCl/Glyce/L-Arg/Cu (OAc)_2_ DES as a green solvent/catalyst medium in the synthesis of 4-substituted-1*H*-1,2,3-triazole derivatives. The results are collected in Table 2. As can be seen, all acetylenes with electron-rich and electron-deficient substitutions underwent the [3 + 2] cycloaddition reaction and provided the title products in good to excellent yields (Table 2, entries 1–9). To find out the potential of the present DES in the making of 1,4-disubstituted-1,2,3-triazoles, the one-pot three-component reaction among the acetylenes prepared from carboxylic acids and phenols, sodium azide, and benzyl bromide or allyl bromide was tested. Based on the data achieved, the related products, except in cases of entries, were obtained in good to excellent yields under mild and optimized conditions (Table 2, entries 12–20). The products were often purified through recrystallization by *n*-hexane/acetone mixture after recovery of the DES.

**Table 2:** Synthesis of 1,2,3-triazole derivatives^a^

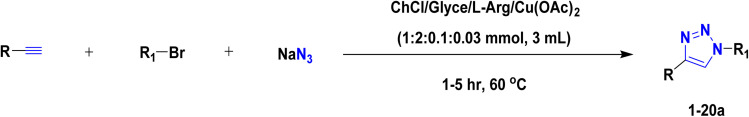					
Entry	R	R_1_	Product	TON	TOF^c^ (h^−1^)
1	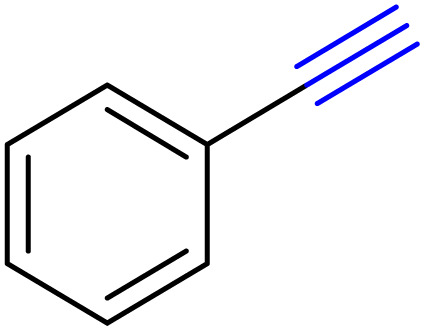	—	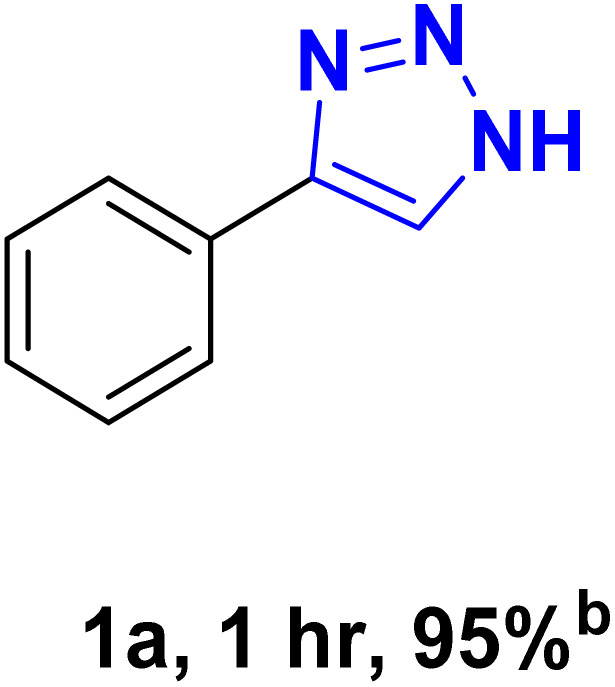	54.29	54.29
2	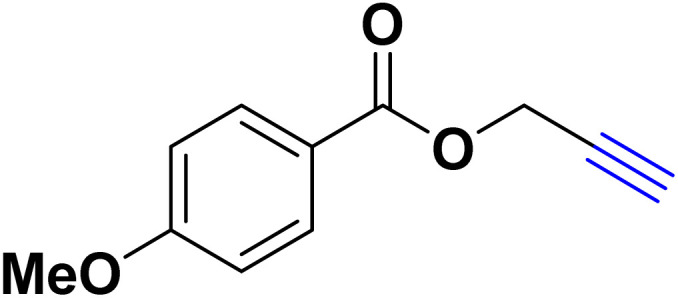	—	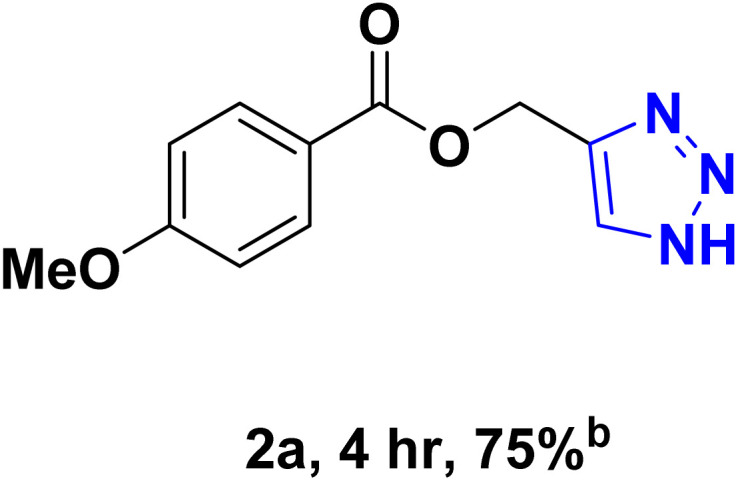	42.86	10.71
3	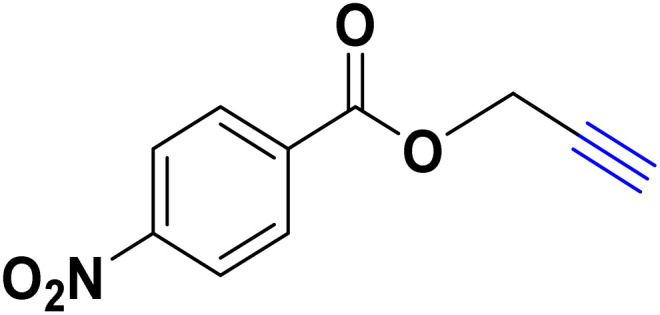	—	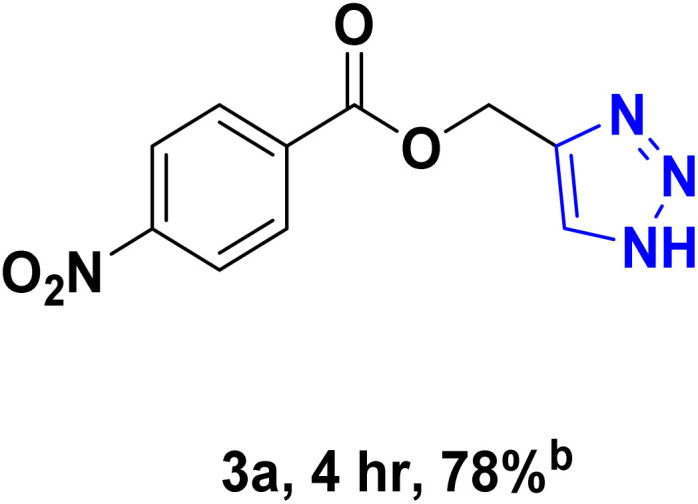	44.57	11.14
4	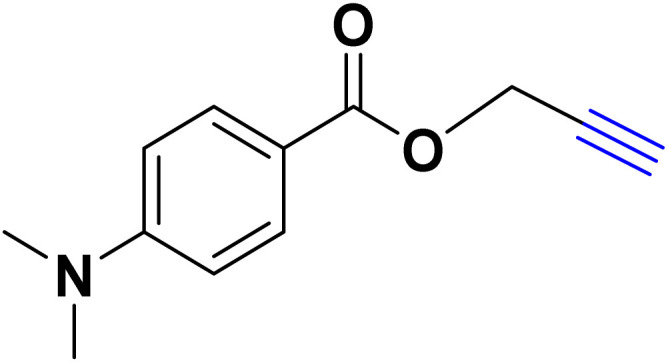	—	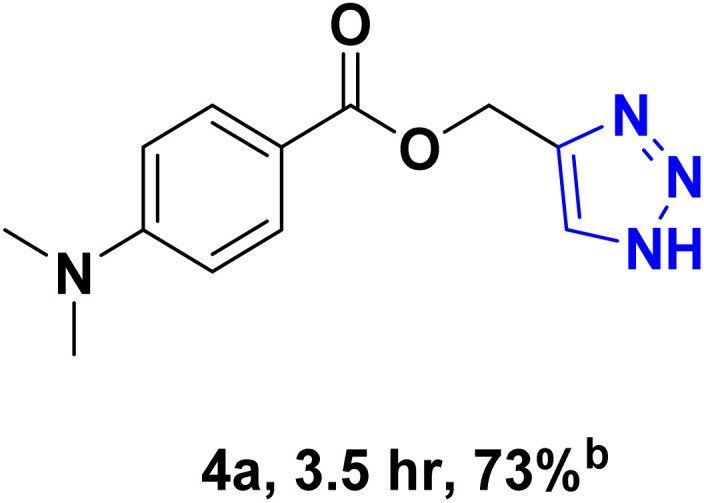	41.71	11.92
5	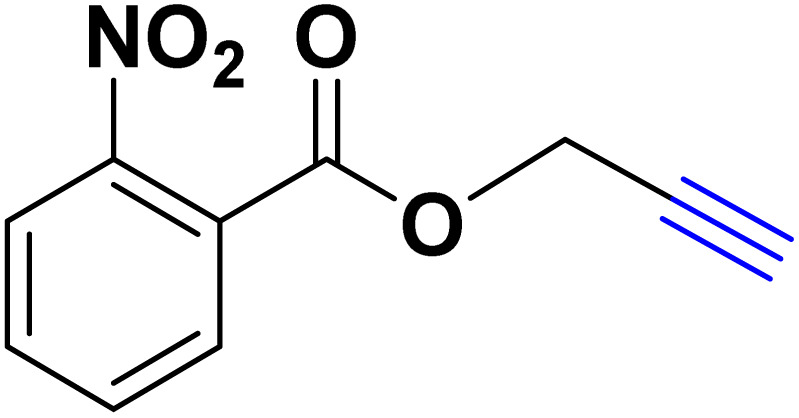	—	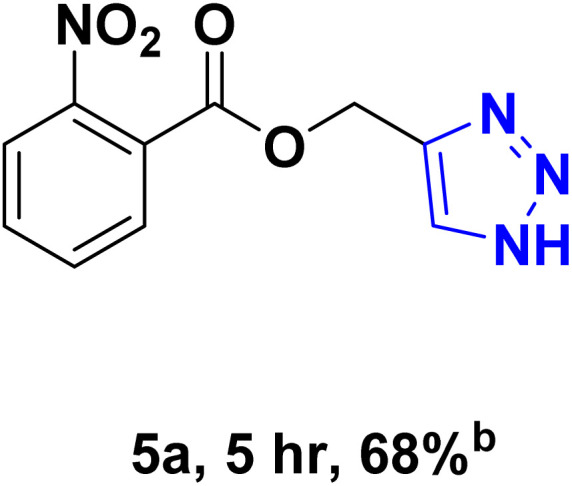	38.86	7.77
6	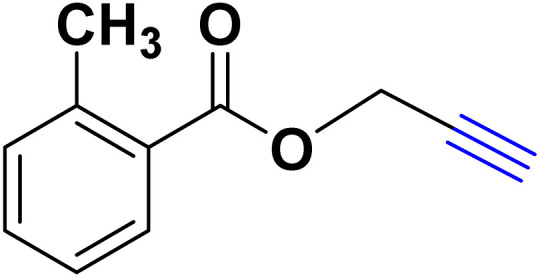	—	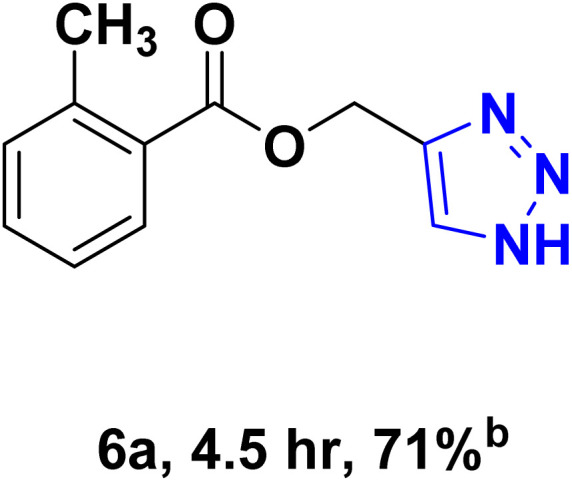	40.57	9.02
7	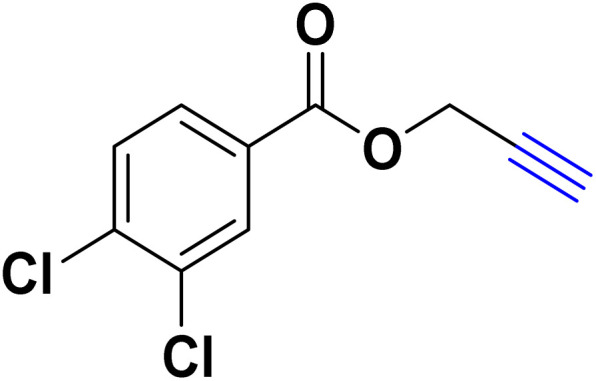	—	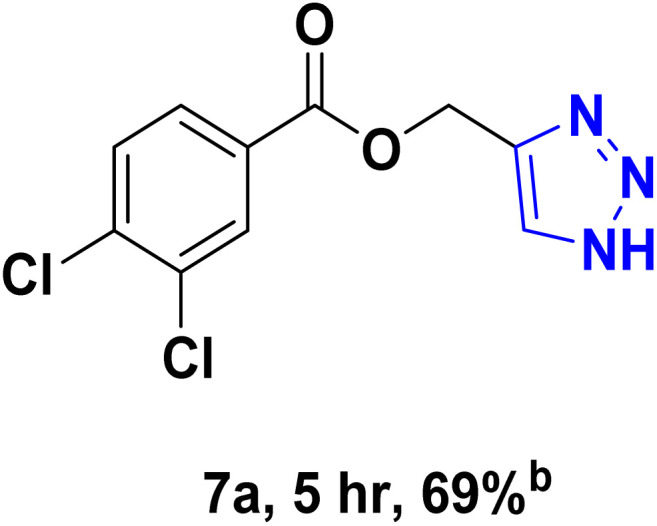	39.43	7.89
8	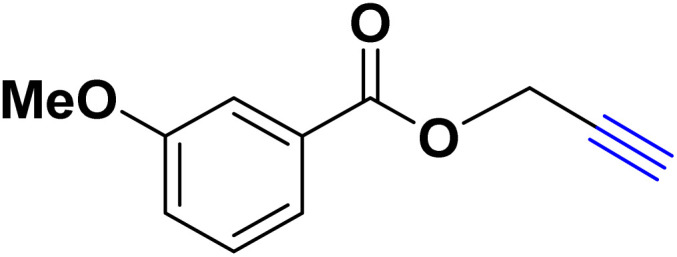	—	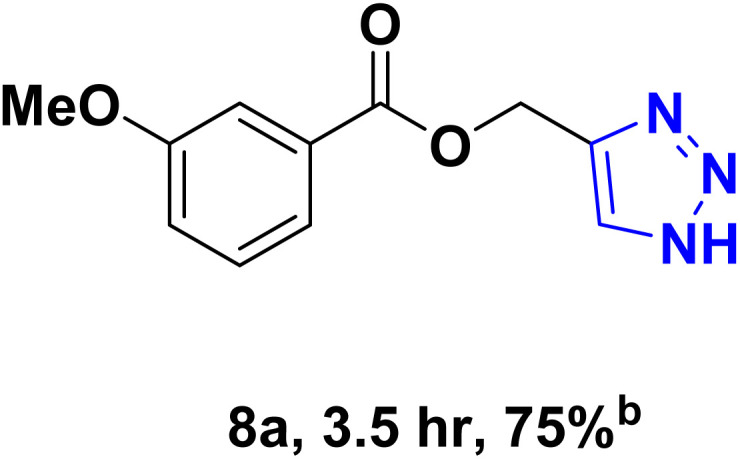	42.86	12.24
9	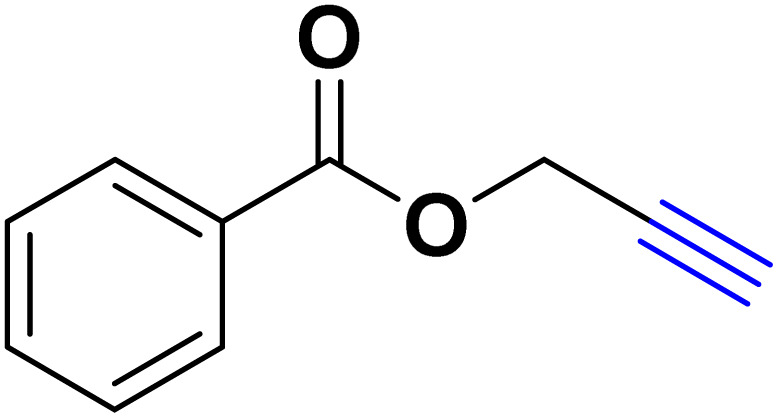	—	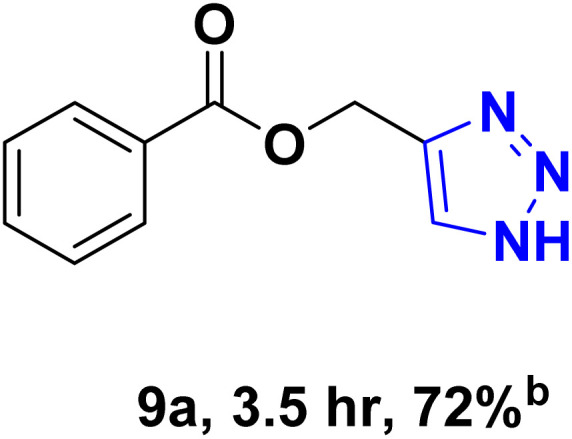	41.14	11.76
10	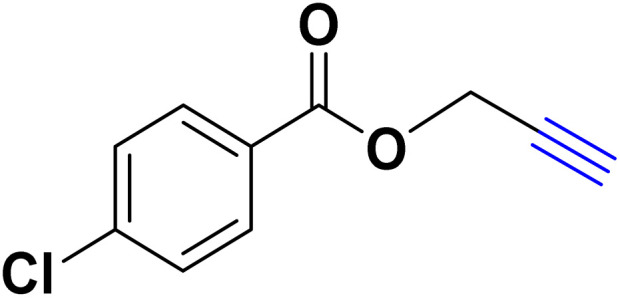	Allyl-	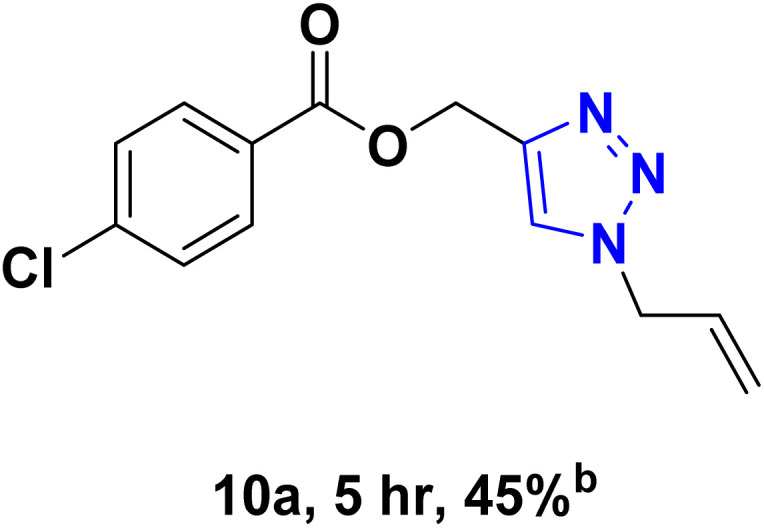	30.20	6.04
11	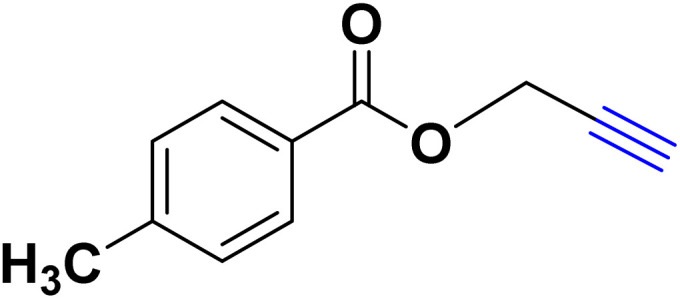	PhCH_2_-	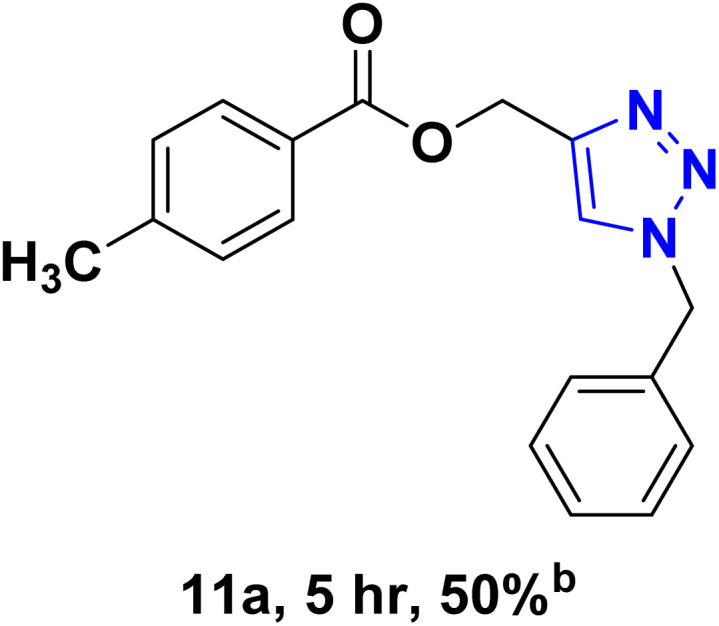	33.56	6.71
12	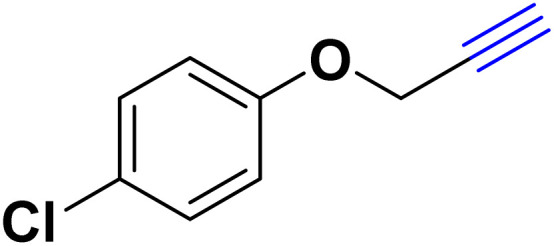	Allyl-	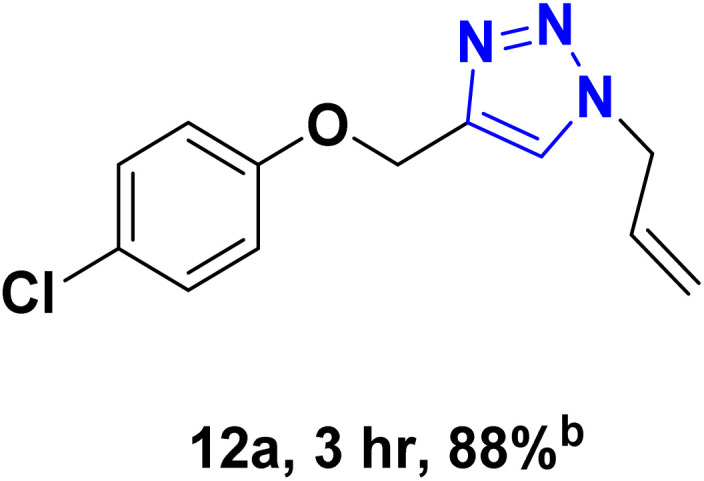	59.06	19.69
13	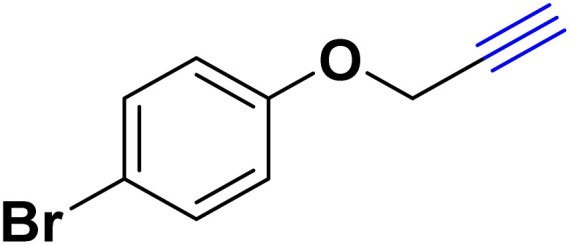	PhCH_2_-	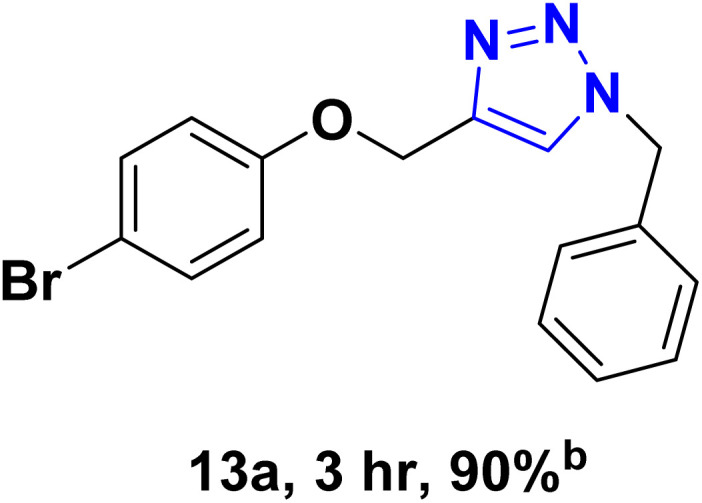	60.40	20.13
14	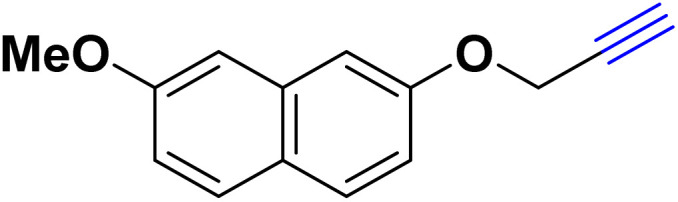	Allyl-	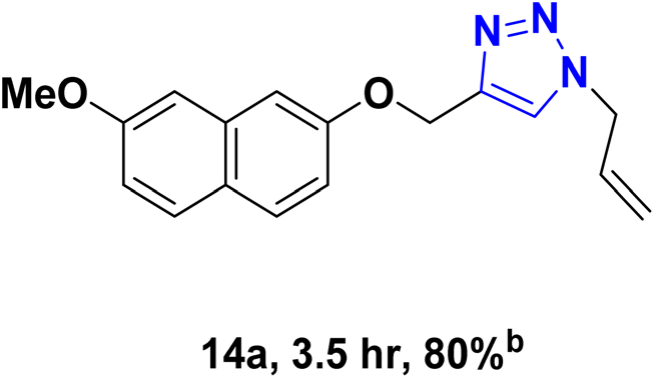	53.69	15.34
15	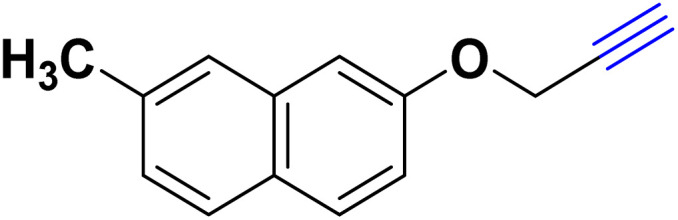	PhCH_2_-	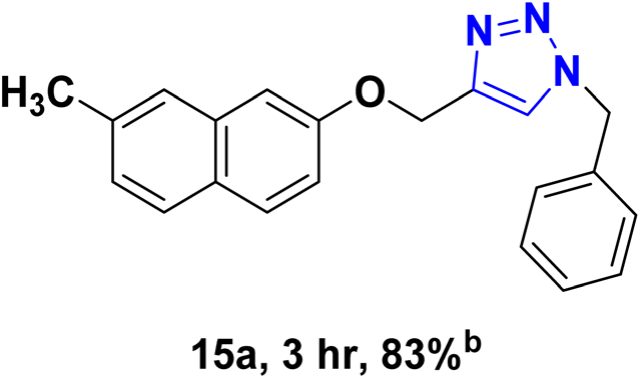	55.70	18.57
16	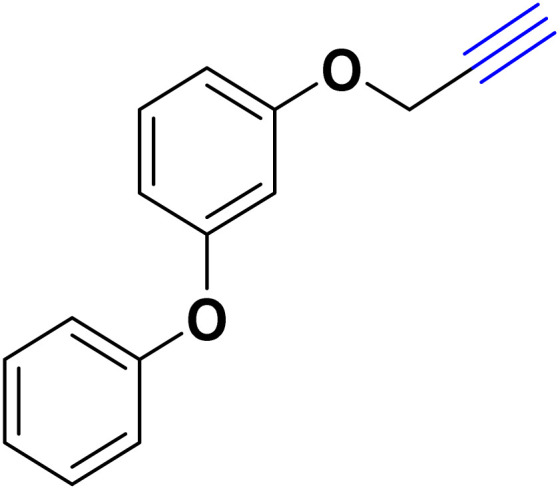	Allyl-	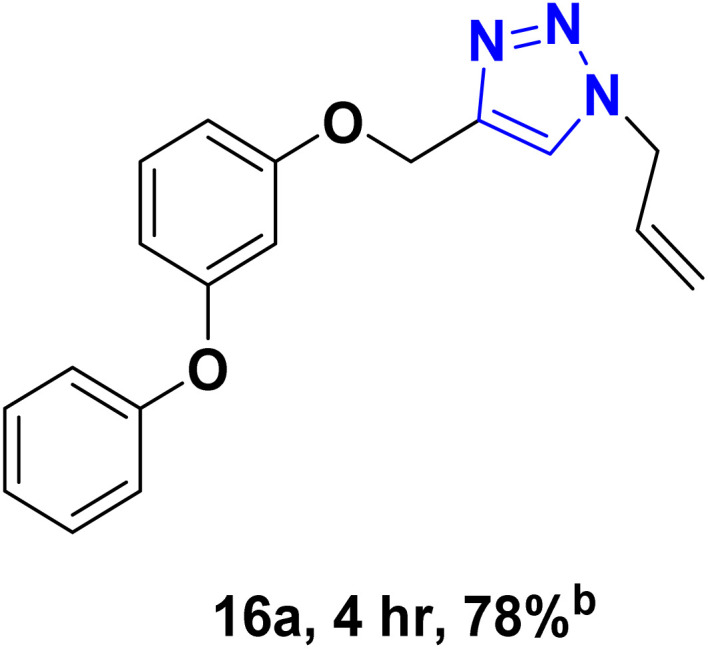	52.35	13.09
17	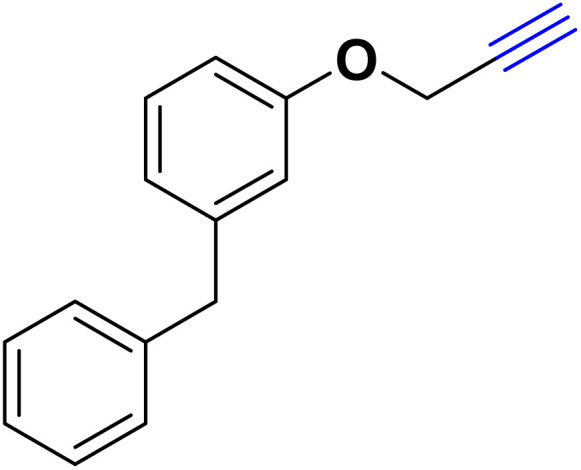	PhCH_2_-	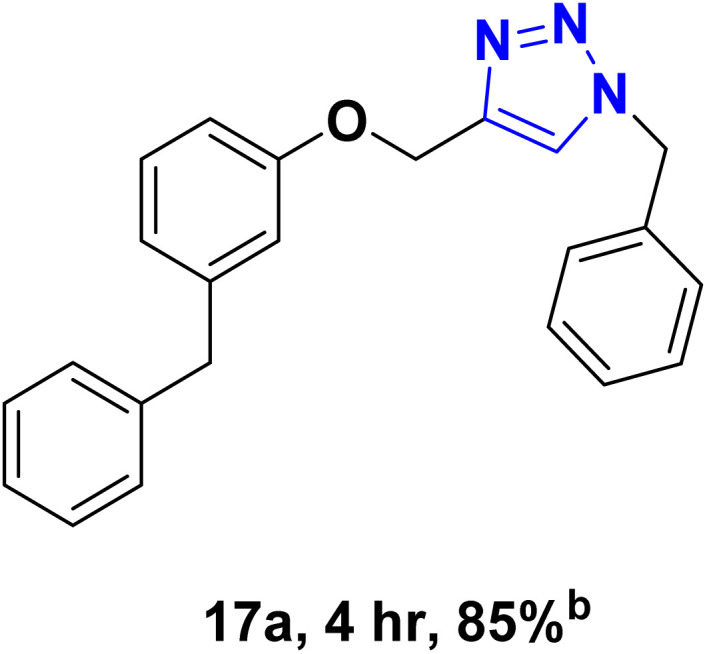	57.05	14.26
18	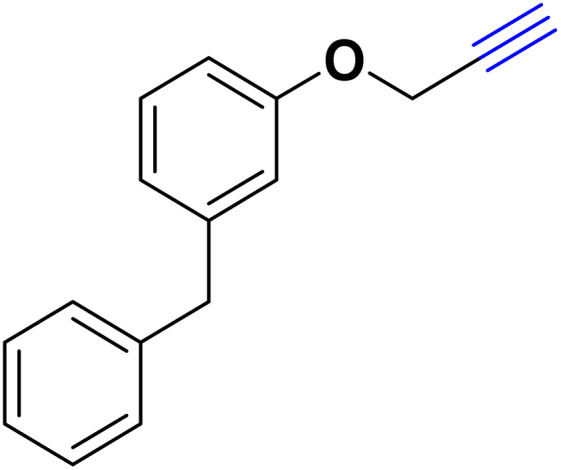	Allyl-	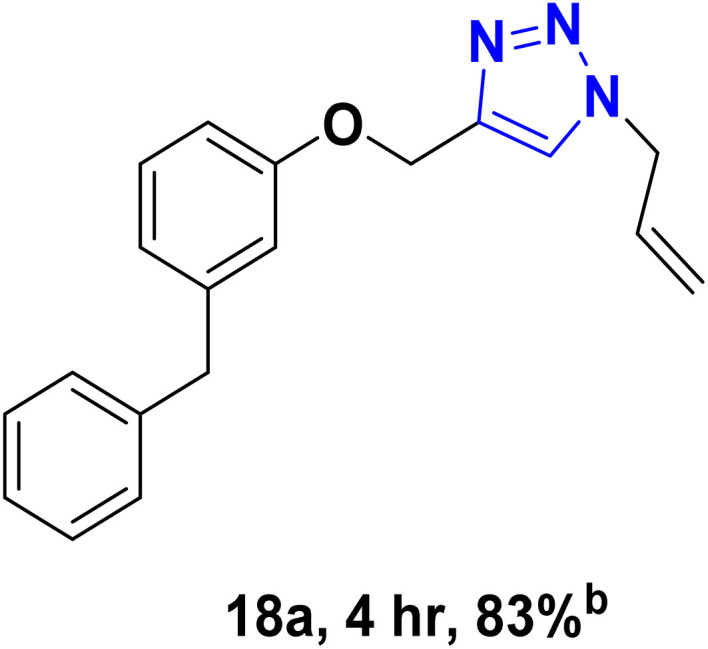	55.70	13.93
19	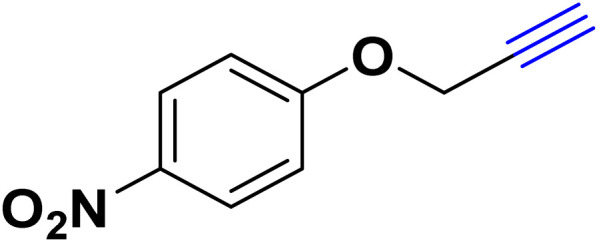	PhCH_2_-	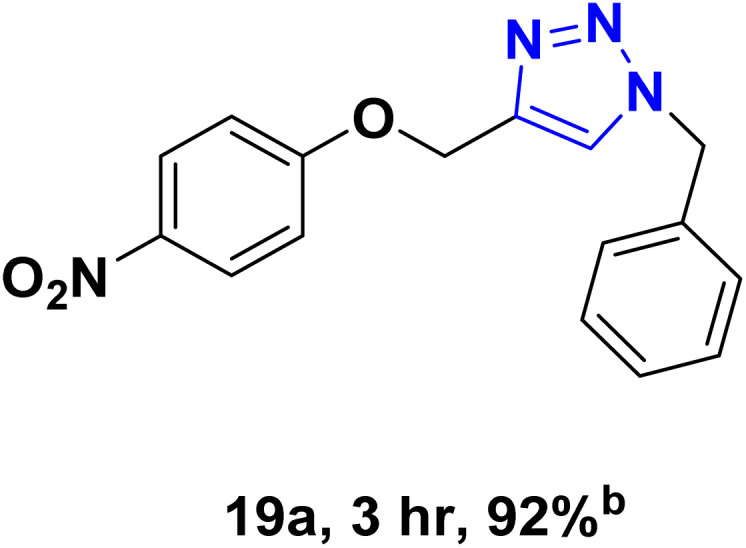	61.74	20.58
20	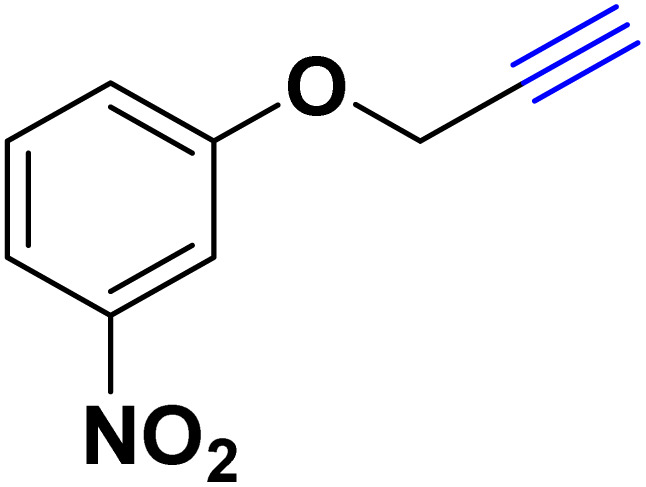	Allyl-	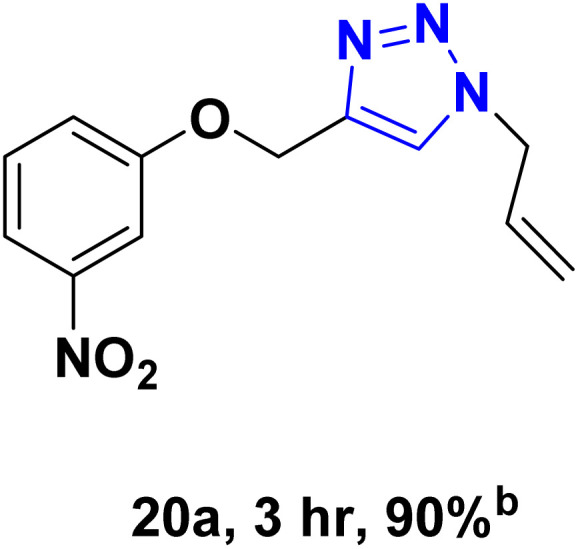	60.40	20.13

a Reaction conditions: acetylene derivative (1 mmol), organic halide (1 mmol), sodium azide (1.17 mmol) in ChCl/Glyce/L-Arg/Cu(OAc)_2_.

b Yield refers to the net products isolated.

c Defined as moles of product per mole of catalyst per h.

**Scheme 3:**
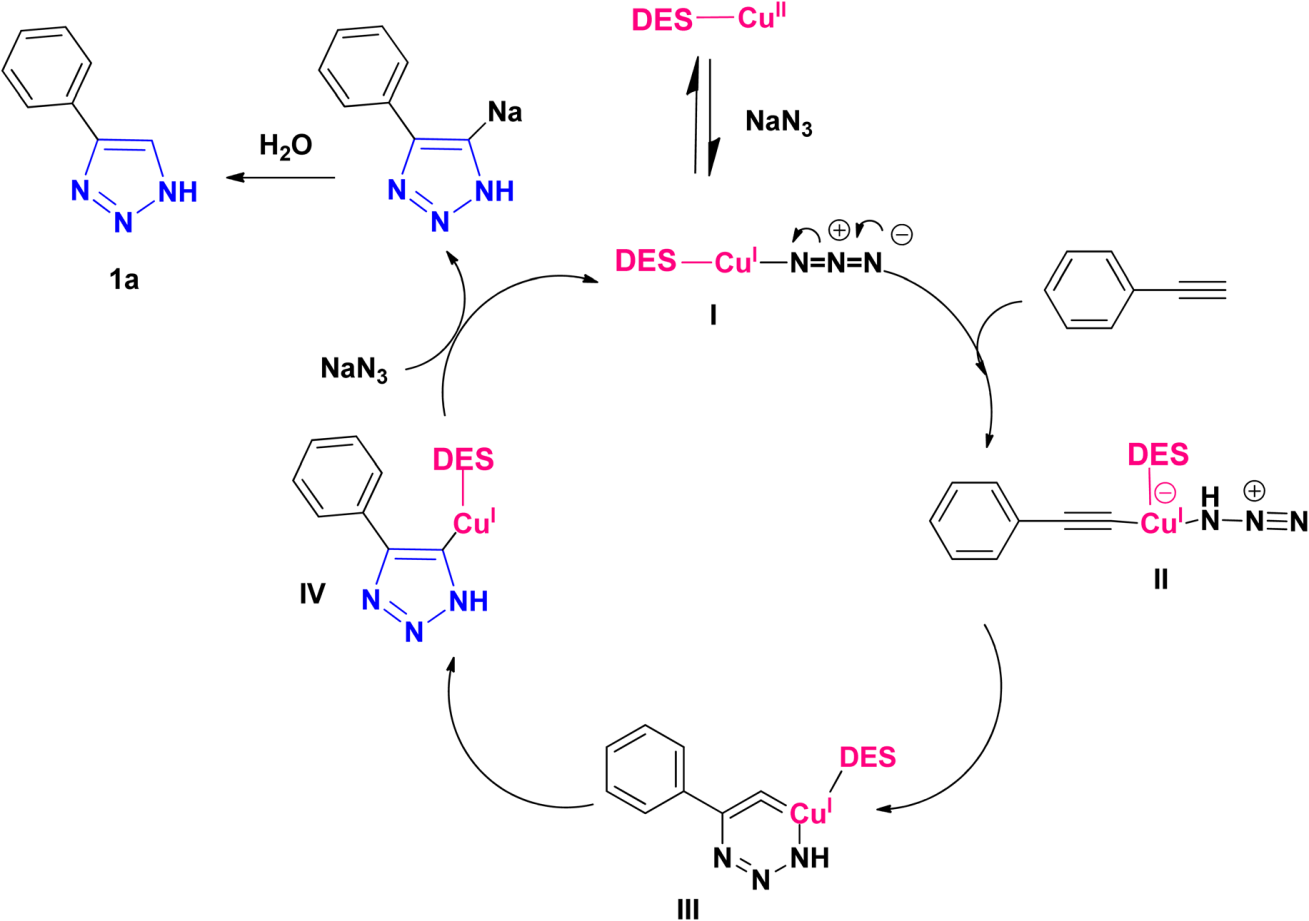
Proposed mechanism for the synthesis of 4-phenyl-1*H*-1,2,3-triazole.

According to the methods presented in the references, the Cu(i) ion can catalyze the formation of 1*H*-1,2,3-triazoles.^6,11,13^ For this reason, in some protocols for the conversion of Cu(ii) to copper(i), reducing agents such as sodium ascorbate and sodium azide have been used.^28^ Therefore, it seems reasonable to start the mechanism in the first step with the reduction of Cu(ii) to Cu(i) to provide I without using additional reducing agents, and based on this, the proposed mechanism for the synthesis of 1*H*-1,2,3-triazole derivatives using phenylacetylene and sodium azide as the reactant models are presented as follows (Scheme 3). The copper II is reduced to copper I with sodium azide in the first step to give I. In the next step, the intermediate I react with the alkyne to form II, which is converted to III after an intramolecular cyclization. Then, the intermediate III rearranges and the isomer IV is created and transformed to triazole (1a) within the reaction environment *via* the protonolysis with water.

A comparison of the effect of the ChCl/Glyce/L-Arg/Cu(OAc)_2_ as a green and new solvent/catalyst medium in the formation of 1*H*-1,2,3-triazole derivatives with some other reported catalysts is collected in Table 3. As can be observed, in contrast to application of the quaternary ChCl/Glyce/L-Arg/Cu(OAc)_2_ DES, which can act as solvent/catalyst system and is obtained *via* quantitative atom economy with just by mixing of the constructive available components, the most of these published catalysts are magnetic and on a nanoscale, which their multi-step synthesis requires a lot of cost, time, production of waste, and the use of organic solvents.

**Table 3:** Comparison of the efficiency of ChCl/Glyce/L-Arg/Cu(OAc)_2_ as a solvent/catalyst system with some catalysts reported in sources for the synthesis of phenyl 1*H*-1,2,3-triazole

Entry	Catalyst	Conditions	Time (h)	Yield a(%)	Ref.
1	Cu(i)-AMPS	H_2_O/RT	1	82	29
2	Fe_3_O_4_@LDH@cysteine–Cu(i)	Choline azide/70 °C	0.4	95	30
3	Fe_3_O_4_@SiO_2_-pAMBA-CS-Cu_2_O	H_2_O/70 °C	0.3	93	31
4	Cu_2_O@Peanut shell	EtOH: H_2_O/50 °C	1.5	93	32
5	CuFe_2_O_4_@SiO_2_@l-arginine@Cu(i)	EtOH: H_2_O/60 °C	0.6	89	33
6	Fe_3_O_4_-DOPA-CuNPs	H_2_O/120 °C	0.2	96	34
7	Sulfated tungstate	DMF/60 °C	1	95	35
8	ChCl/Glyce/L-Arg/Cu(OAc)_2_	60 °C	1	95	This work

a Yield refers to the net products isolated.

Regarding the successful application of the biocompatible and biodegradable DES, [ChCl][Glyce]_2_[L-Arg]_0.1_[Cu(OAc)_2_]_0.03_, in the synthesis of the 1,2,3-triazoles, we encouraged to check its ability as a solvent/catalyst system in the providing 5-substituted tetrazoles. Therefore, we chose the model reaction of benzonitrile and sodium azide to optimize the reaction factors. The results were registered in Table 4. We ran the model reaction under ambient temperature, 60 °C, and 90 °C generated 5-phenyl tetrazole in 60%, 80%, and 90% yield in 2 mL of the DES, respectively (Table 4, entries 1–3). The best result was achieved when 2 mL of the ChCl/Glyce/L-Arg/Cu (OAc)_2_ DES was used, and the mixture was blended for 1.5 h at 110 °C (Table 4, entry 4). Performing the model reaction in a lower volume (1 mL) of the present DES reduced its efficiency and afforded the tetrazole product to 90% (Table 4, entry 5), and using 3 mL of the DES did not lead to any improvement in the efficiency and forming 97% yield of the title compound at 110 °C after 1.5 h (Table 4, entry 6). No significant change was observed in the efficiency of the reaction when a higher molar ratio of Cu (OAc)_2_ was utilized in the DES structure and a higher volume of DES was used (Table 4, entries 7 and 8). The reaction did not proceed without the ChCl/Glyce/L-Arg/Cu (OAc)_2_ DES, which proves the DES's crucial role as a solvent/catalyst media (Table 4, entry 9). When the reaction was accomplished under solvent-free conditions in the presence of Cu (OAc)_2_ as one of the DES precursors, only 40% of 4-phenyl-1*H*-1,2,3-tetrazole was produced after 6 h at 110 °C (Table 4, entry 10). The effect of the ChCl/Glyce/L-Arg DES, as the other precursor, was also not high in offering the desired product and resulted in a yield of about 30% (Table 4, entry 11). These two last facts, along with the result achieved in entry 2, exhibited the occurrence of synergic effect phenomena between Cu(OAc)_2_ and the ChCl/Glyce/L-Arg when they were blended to form the ChCl/Glyce/L-Arg/Cu(OAc)_2_ DES. As specified by the information in Table 4, using 1 mmol benzonitrile and 1.4 mmol sodium azide in 2 mL of [ChCl][Glyce]_2_[L-Arg]_0.1_[Cu (OAc)_2_]_0.03_ at 110 °C can be used as the optimized reaction conditions for the production of 5-substituted-1*H*-tetrazoles in follows.

**Table 4:** Optimizing reaction conditions for the synthesis of 5-substituted-1*H*-tetrazoles^a^

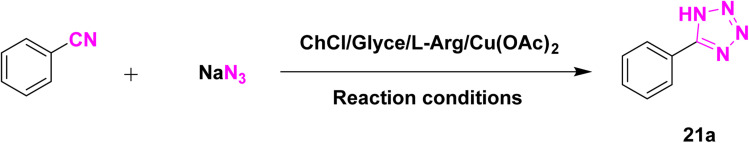						
Entry	Reaction media	Molar ratio	Catalyst amount (mL)	Temp. (°C)	Time (h)	Yield^b^ (%)
1	ChCl/Glyce/L-Arg/Cu(OAc)_2_	1 : 2 : 0.1 : 0.03	2	RT	12	60
2	ChCl/Glyce/L-Arg/Cu(OAc)_2_	1 : 2 : 0.1 : 0.03	2	60	6	80
3	ChCl/Glyce/L-Arg/Cu(OAc)_2_	1 : 2 : 0.1 : 0.03	2	90	4	90
**4**	**ChCl/Glyce/L-Arg/Cu(OAc)**_**2**_	**1** : **2** : **0.1** : **0.03**	**2**	**110**	**1.5**	**97**
5	ChCl/Glyce/L-Arg/Cu(OAc)_2_	1 : 2 : 0.1 : 0.03	1	110	2	90
6	ChCl/Glyce/L-Arg/Cu(OAc)_2_	1 : 2 : 0.1 : 0.03	3	110	1.15	97
7	ChCl/Glyce/L-Arg/Cu(OAc)_2_	1 : 2 : 0.1 : 0.04	2	110	1.5	97
8	ChCl/Glyce/L-Arg/Cu(OAc)_2_	1 : 2 : 0.1 : 0.05	2	110	1.5	97
9	—	—	—	90	12	—
10	Cu(OAc)_2_	0.03	—	110	6	40
11	ChCl/Glyce/L-Arg	1 : 2 : 0.1	2	110	10	30

a Reaction conditions: benzonitrile (1 mmol) and sodium azide (1.4 mmol).

b Yield refers to the net products isolated.

After optimizing the reaction conditions to check versatility and determine the reaction scope, varieties of the nitriles were treated with sodium azide in the ChCl/Glyce/L-Arg/Cu (OAc)_2_ DES (Table 5). The benzonitriles with electron-withdrawing and the electron-releasing substitutions undertook the reaction under the optimized conditions and offered the related tetrazoles in excellent yields (Table 5, entries 1–6, 9 and 10). Also, 4-cyanopyridine (as a heteroaromatic nitrile) and benzyl cyanide (as an aliphatic nitrile) provided the desired tetrazole in 92% and 90% yield indefinitely when reacted with sodium azide in the title DES.

**Table 5:** Synthesis of 5-substituted-1*H*-tetrazole derivatives^a^

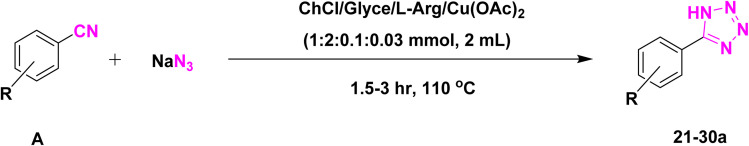				
Entry	A	Product	TON	TOF^c^ (h^−1^)
1	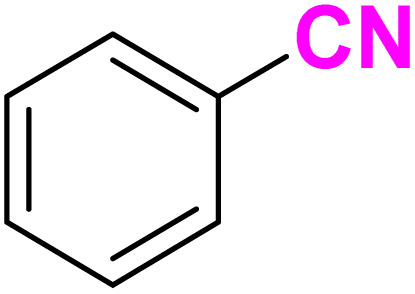	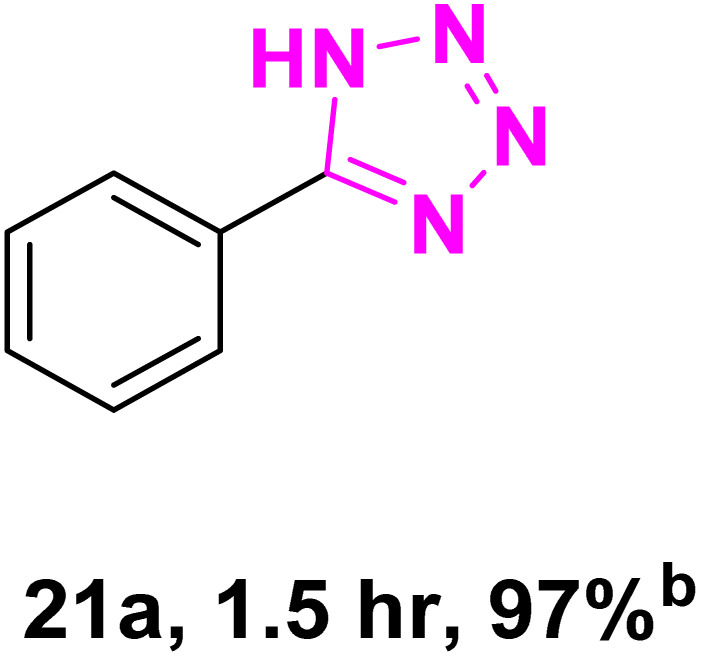	53.59	35.72
2	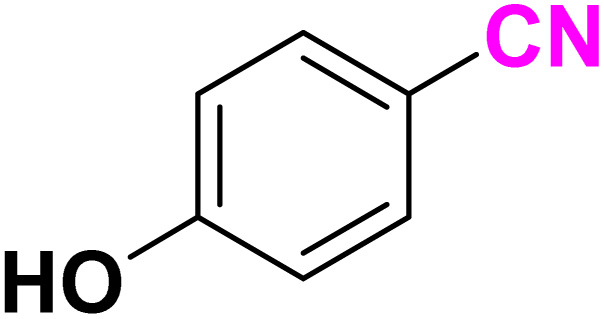	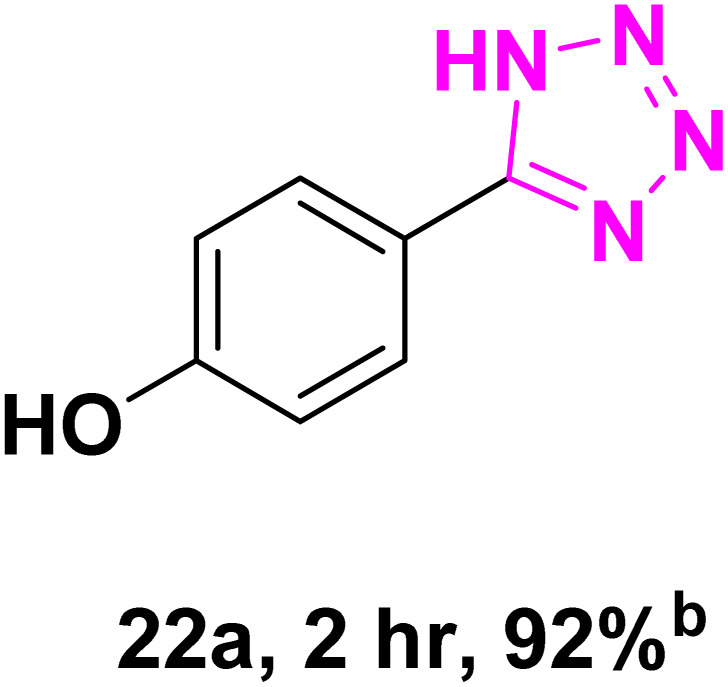	50.83	25.41
3	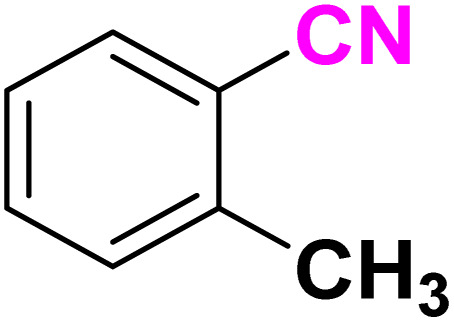	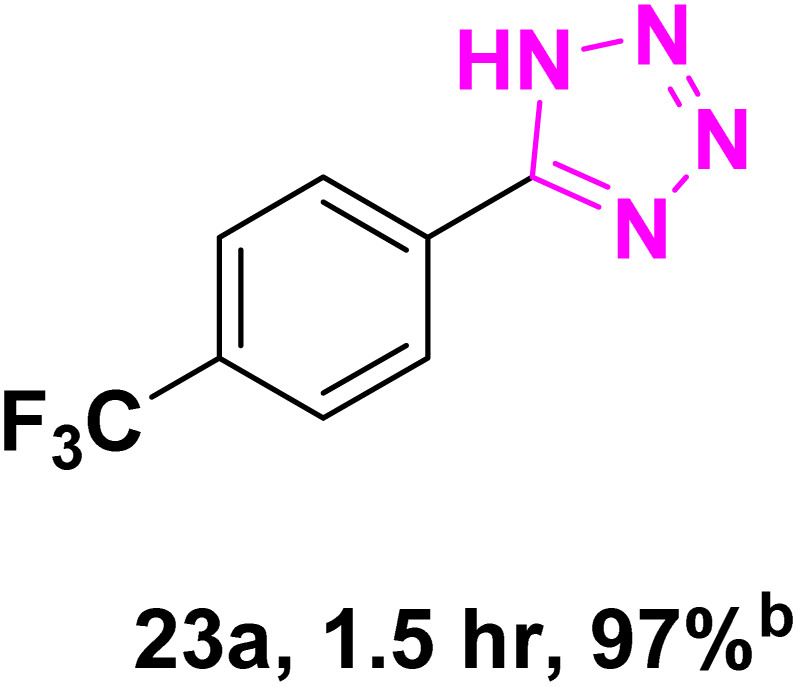	53.59	35.72
4	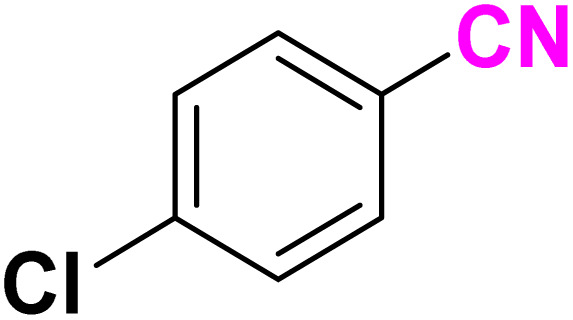	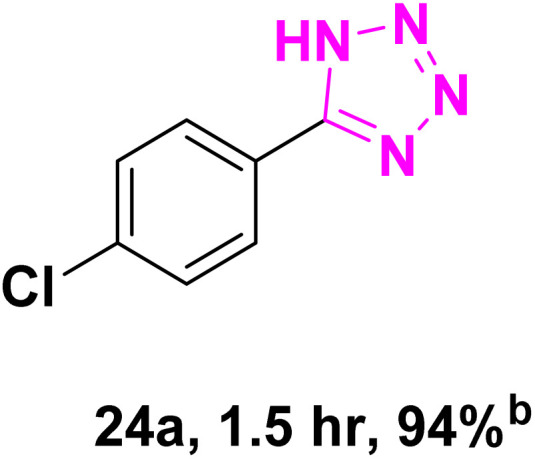	51.93	34.62
5	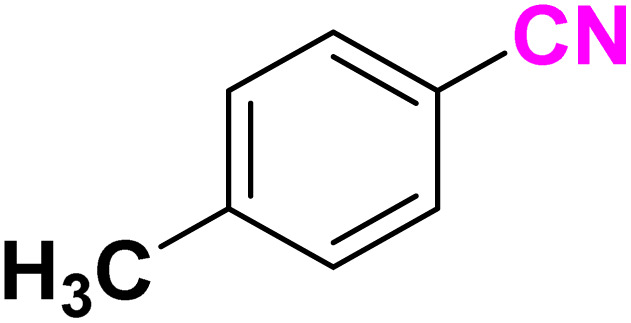	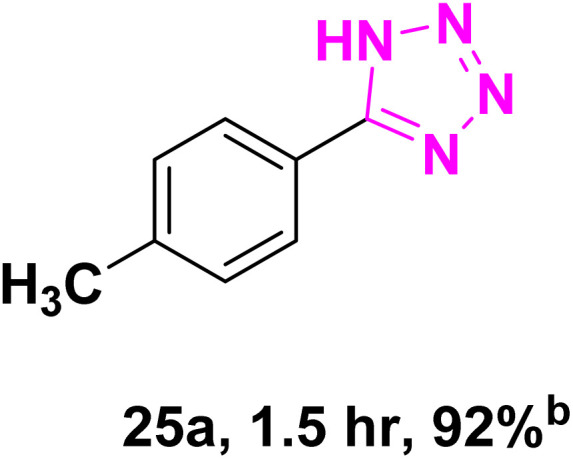	50.83	33.89
6	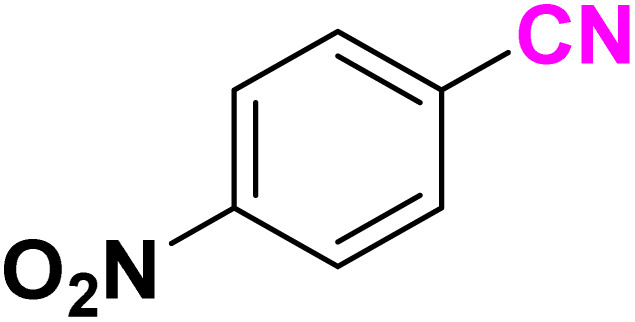	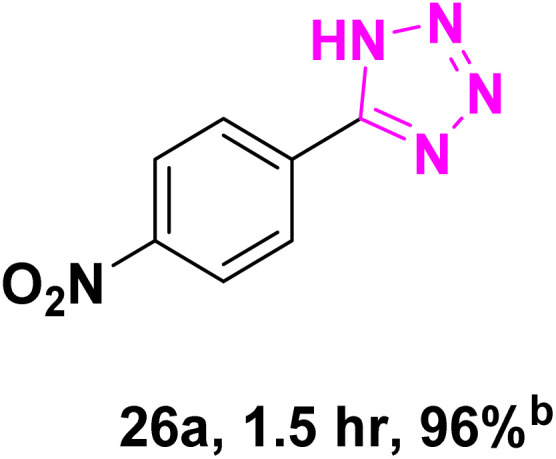	53.04	35.36
7	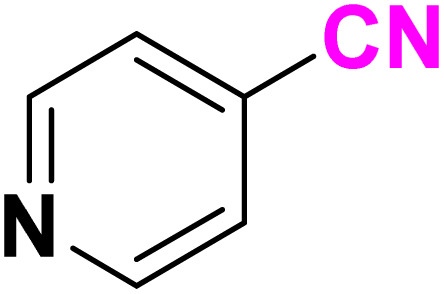	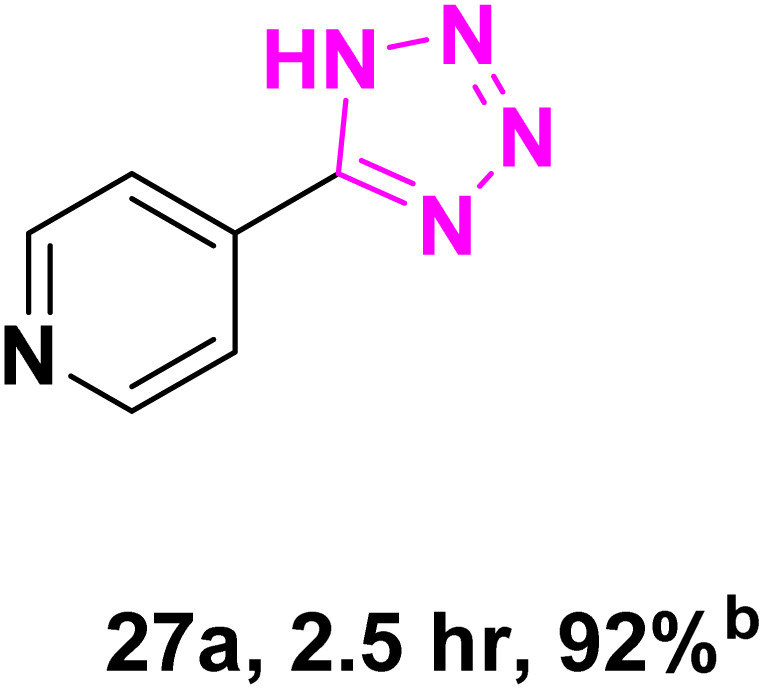	50.83	20.33
8	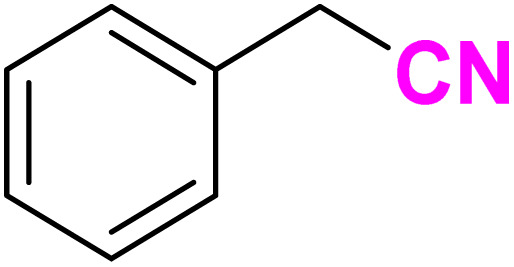	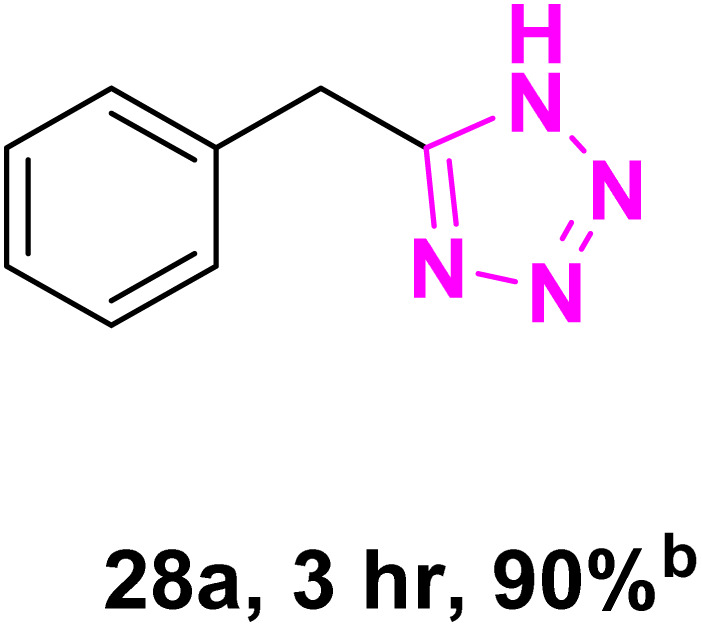	49.72	16.57
9	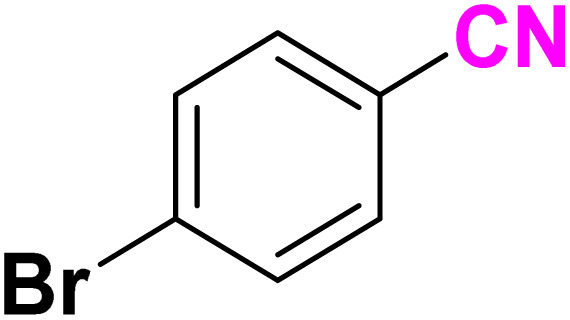	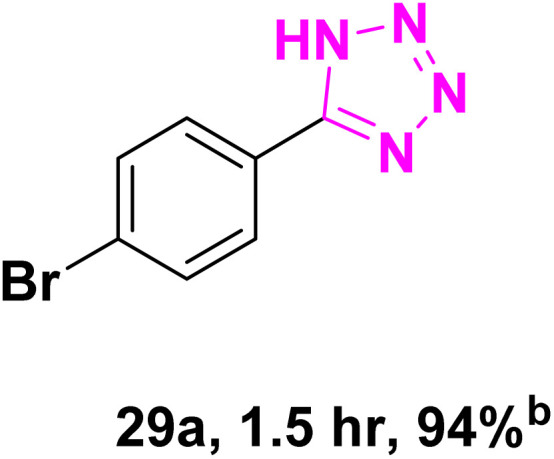	51.93	34.62
10	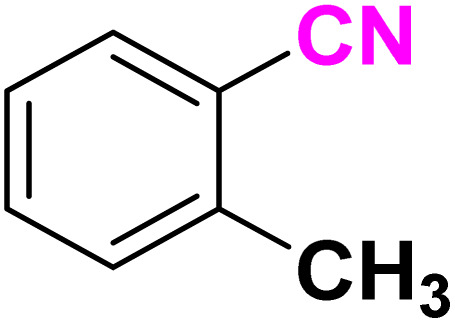	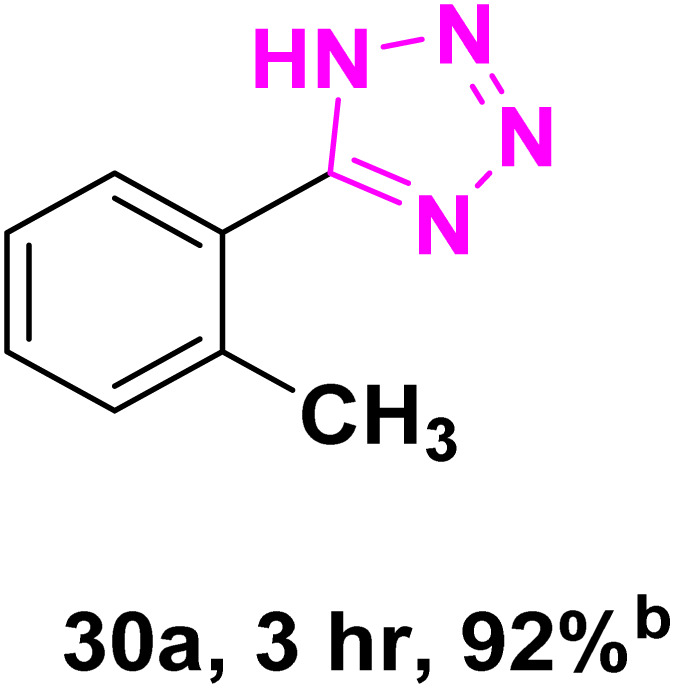	50.83	16.94

a Reaction conditions: different benzonitriles (1 mmol) and sodium azide (1.4 mmol) in ChCl/Glyce/L-Arg/Cu(OAc)_2_.

b Yield refers to the net products isolated.

c Defined as moles of product per mole of catalyst per h.

**Scheme 4:**
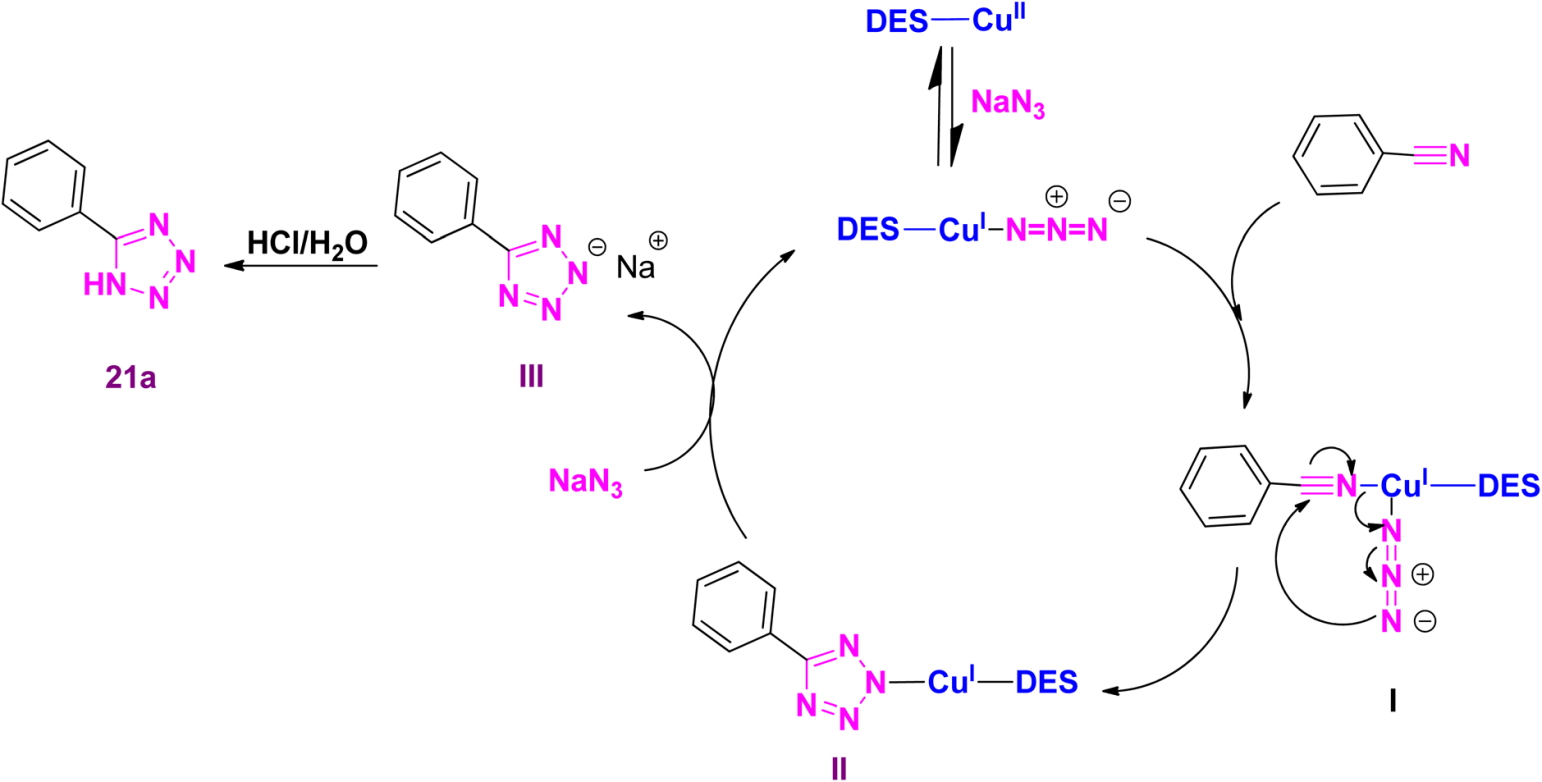
Plausible mechanism for the synthesis of 5-phenyl-1*H*-tetrazole.

An acceptable mechanism for preparing 5-substituted-1*H*-tetrazoles using sodium azide and benzonitrile as the prototype reaction is shown in Scheme 4. Initially, Cu(ii) in the DES is reduced to DES-Cu(i)–N_3_, which activates the nitrile group in benzonitrile by coordination to provide the transient species I. The intramolecular cyclization reaction in I through attacking the terminal nitrogen atom of azide on the nitrile functional group to generates the particle II. The reaction of sodium azide with II is led to regenerate DES-Cu(i)–N_3_ to initiate a new reaction cycle and produce the sodium salt of tetrazole III, which is transformed to the tetrazole 21a by acidic hydrolysis in work up step.

#### 3.2.1. Recovery of the solvent/catalyst ChCl/Glyce/L-Arg/Cu(OAc)_2_ in the preparation of 1*H*-1,2,3-triazoles derivatives and 5-substituted-1*H*-tetrazoles

**Fig. 9:**
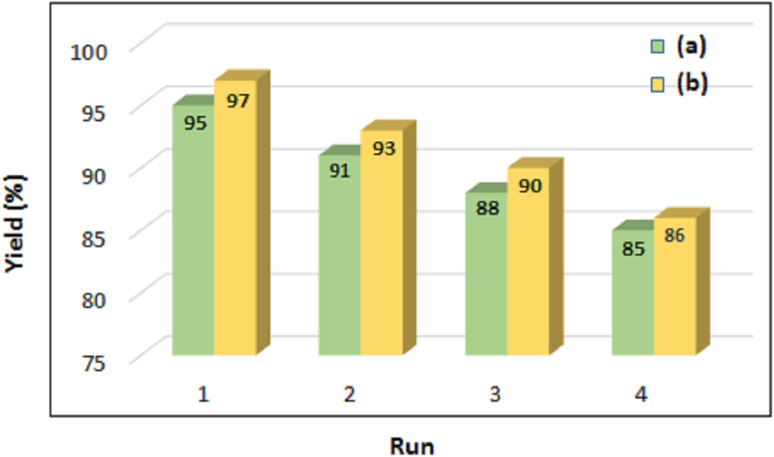
(a) Evaluation of the reusability of ChCl/Glyce/L-Arg/Cu(OAc)_2_ DES in the synthesis of 4-phenyl-1*H*-1,2,3-triazoles and (b) 5-phenyl-1*H*-tetrazoles.

**Fig. 10:**
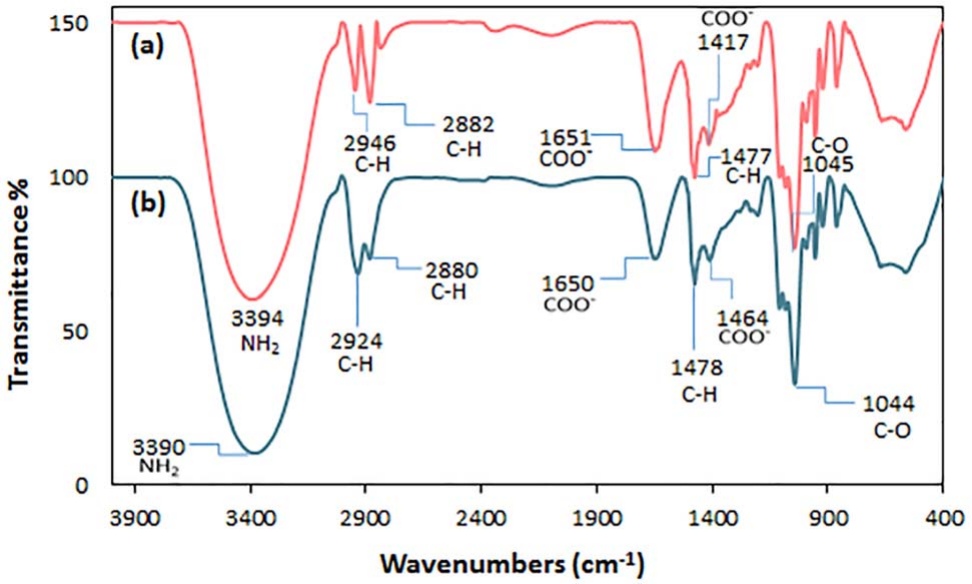
FTIR spectra of (a) fresh DES and (b) recovered DES.

One key factor determining a catalyst's effectiveness and economic viability is its recovery and reuse. Using recovered catalysts in industrial processes can reduce production costs and minimize the resulting waste. In this work, the efficiency and reusability of the ChCl/Glyce/L-Arg/Cu(OAc)_2_ DES was evaluated in the obtaining of 1*H*-1,2,3-triazole derivatives using phenylacetylene and sodium azide as the model reactants. After the completion of the reaction, distilled water and chloroform were added to the reaction medium, and the organic and aqueous phases were separated. Also, the reusability of ChCl/Glyce/L-Arg/Cu (OAc)_2_ in the production of 5-substituted-1*H*-tetrazoles was investigated using benzonitrile and sodium azide as the model reactants. After the completion of this experiment, by adding 5 M HCl and EtOAc to the reaction medium, the organic phases and the aqueous were separated. In both reactions, the aqueous layer was placed under the reduced pressure for 1 h at 60 °C to recover the ChCl/Glyce/L-Arg/Cu(ii) DES and use it in subsequent cycles. This study revealed that the DES can be four times recyclable and reusable without losing efficiency (Fig. 9a and b). This finding was confirmed by comparing the FT-IR spectra of fresh DES (Fig. 10a) and recycled DES and their similarity (Fig. 10b).

In addition, the efficiency evaluation of this method was performed in the synthesis of 5-phenyl-1*H*-tetrazole through click reaction using benzonitrile and sodium azide against some other catalysts available in the recent scientific sources, and the related data were registered in Table 6. As can be seen, the current DES, as a safe and biocompatible environment with the dual role of solvent and catalyst, can be formed by simply mixing the related biocompatible components with a complete atom economy, providing the title product with excellent efficiency at one of the relatively short times with avoiding application of toxic solvents, also toxic and expensive metal.

**Table 6:** Comparison of the efficiency of ChCl/Glyce/L-Arg/Cu(OAc)_2_ as a solvent/catalyst system with some catalysts reported in sources for the synthesis of 5-phenyl-1*H*-tetrazole

Entry	Catalyst	Conditions	Time (h)	Yield^a^ (%)	TOF^b^ (h^−1^)	Ref.
1	Boehmite@SiO_2_@Tris-Cu(i)	PEG/120 °C	2	95	1.39	36
2	Fe_3_O_4_@l-lysine-Pd(0)	H_2_O/100 °C	1	99	330	17
3	Cu(ii)-DCC-CMK-3	PEG/120 °C	1.5	97	2.65	37
4	MCM-41-SO_3_H	DMF/80 °C	2	90	0.97	38
5	Choline chloride/ZnCl_2_	140 °C	0.5	90	50.42	39
6	Choline chloride/glycerol	140 °C	2	70	9.46	39
7	[BiPy](HSO_3_)_2_Cl_2_	Ethylene glycol/100 °C	1.5	99	2.2	18
8	ChCl/Glyce/L-Arg/Cu(OAc)_2_	110 °C	1.5	97	35.72	This work

a Yield refers to the net products isolated.

b Defined as moles of product per mole of catalyst per h.

## 4. Conclusion

We have prepared a novel, biocompatible and cost-effective Cu(ii)-based quaternary deep eutectic solvent, [ChCl][Glyce]_2_[L-Arg]_0.1_[Cu (OAc)_2_]_0.03_, with a simple mixing of the non-toxic and available components using a quantitative atom economy way. After characterization, its efficiency and capability as a dual solvent/catalyst were explored for the synthesis of 1,4-disubstituted-1,2,3-triazoles, 4-substituted-1*H*-1,2,3-triazole, and 5-substituted-1*H*-tetrazole by click reaction strategy. This study disclosed the high to excellent efficiency of the QDES for the synthesis of the desired tetrazoles and triazoles under mild reaction conditions as a reusable, biodegradable, inexpensive, and non-toxic solvent/catalyst media.

## Data availability

The data that support the findings of this study are available in the ESI file.†

## Author contributions

L. G.: resources, validation, data curation, visualization. Methodology, formal analysis, investigation, writing – original draft. A. R. S.: resources, conceptualization, supervision, investigation, writing – review & editing.

## Conflicts of interest

The authors have no competing interests to declare that are relevant to the content of this article.

## Supplementary Material

RA-015-D4RA08090D-s001
