# Supplementary material for: Introduction and characterization of a novel Cu(ii)-based quaternary deep eutectic solvent and its application in the efficient synthesis of triazoles and tetrazoles under mild conditions as an inexpensive, reusable, benign, and dual solvent/catalyst medium
Source: RSC Adv. 2025 Feb 3;15(5):3389–405. doi: 10.1039/d4ra08090d (PMC11788717; doi:10.1039/d4ra08090d)
Supplement: RA-015-D4RA08090D-s001 [file RA-015-D4RA08090D-s001.pdf]

Supplementary Information (SI) for RSC Advances.
This journal is © The Royal Society of Chemistry 2025

SUPPLEMENTARY MATERIAL

Introduction and characterization of a Novel Cu (II)-Based Quaternary Deep Eutectic Solvent and Its Application in the Efficient Synthesis of Triazoles and Tetrazoles Under Mild Conditions as an Inexpensive, Reusable, Benign, and Dual Solvent/Catalyst Media

Laleh Golestanifar [a], Ali Reza Sardarian [a,*]

[1] Chemistry Department, College of Science, Shiraz University, Shiraz 71946-84795, Iran

*Corresponding author: Fax: 0098-36460788, Tel: 0098-71-36137107, Email address: sardarian@shirazu.ac.ir

Contents:

Figure 1. FT-IR spectrum of 4-Phenyl-1*H*-1,2,3-triazole (**1a**)……………………………………... (13)

Figure 2. ^{1}H NMR spectrum of 4-Phenyl-1*H*-1,2,3-triazole (**1a**)…………………………………… (13)

Figure 3. ^{13}C NMR spectrum of 4-Phenyl-1*H*-1,2,3-triazole (**1a**)…………………………….…. (14)

Figure 4. FT-IR spectrum of (1*H*-1,2,3-triazol-4-yl)methyl 4-methoxybenzoate (**2a**)…………….. (14)

Figure 5. ^{1}H NMR spectrum of (1*H*-1,2,3-triazol-4-yl)methyl 4-methoxybenzoate (**2a**)………..…. (15)

Figure 6. ^{13}C NMR spectrum of (1*H*-1,2,3-triazol-4-yl)methyl 4-methoxybenzoate (**2a**)………… (15)

Figure 7. FT-IR spectrum of (1*H*-1,2,3-triazol-4-yl)methyl 4-nitrobenzoate (**3a**)…………………. (16)

Figure 8. ^{1}H NMR spectrum of (1*H*-1,2,3-triazol-4-yl)methyl 4-nitrobenzoate (**3a**)……………… (16)

Figure 9. ^{13}C NMR spectrum of (1*H*-1,2,3-triazol-4-yl)methyl 4-nitrobenzoate (**3a**)……………… (17)

Figure 10. FT-IR spectrum of (1*H*-1,2,3-triazol-4-yl)methyl 4-(dimethylamino)benzoate (**4a**)…… (17)

Figure 11. ^{1}H NMR spectrum of (1*H*-1,2,3-triazol-4-yl)methyl 4-(dimethylamino)benzoate (**4a**)… (18)

Figure 12. ^{13}C NMR spectrum of (1*H*-1,2,3-triazol-4-yl)methyl 4-(dimethylamino)benzoate (**4a**)... (18)

Figure 13. FT-IR spectrum of (1*H*-1,2,3-triazol-4-yl)methyl 2-nitrobenzoate (**5a**)………………… (19)

Figure 14. ^{1}H NMR spectrum of (1*H*-1,2,3-triazol-4-yl)methyl 2-nitrobenzoate (**5a**)……………… (19)

Figure 15. ^{13}C NMR spectrum of (1*H*-1,2,3-triazol-4-yl)methyl 2-nitrobenzoate (**5a**)…………….(20)

Figure 16. FT-IR spectrum of (1*H*-1,2,3-triazol-4-yl)methyl 2-methylbenzoate (**6a**)……………… (20)

Figure 17. ^{1}H NMR spectrum of (1*H*-1,2,3-triazol-4-yl)methyl 2-methylbenzoate (**6a**)…………… (21)

Figure 18. ^{13}C NMR spectrum of (1*H*-1,2,3-triazol-4-yl)methyl 2-methylbenzoate (**6a**)………….. (21)

Figure 19. FT-IR spectrum of (1*H*-1,2,3-triazol-4-yl)methyl 3,4-dichlorobenzoate (**7a**)………….. (22)

Figure 20. ^{1}H NMR spectrum of (1*H*-1,2,3-triazol-4-yl)methyl 3,4-dichlorobenzoate (**7a**)……….(22)

Figure 21. ^{13}C NMR spectrum of (1*H*-1,2,3-triazol-4-yl)methyl 3,4-dichlorobenzoate (**7a**)………..(23)

Figure 22. FT-IR spectrum of (1*H*-1,2,3-triazol-4-yl)methyl 3-methoxybenzoate (**8a**)…………….(23)

Figure 23. ^{1}H NMR spectrum of (1*H*-1,2,3-triazol-4-yl)methyl 3-methoxybenzoate (**8a**)…………..(24)

Figure 24. ^{13}C NMR spectrum of (1*H*-1,2,3-triazol-4-yl)methyl 3-methoxybenzoate (**8a**)………….(24)

Figure 25. FT-IR spectrum of (1*H*-1,2,3-triazol-4-yl)methyl benzoate (**9a**)……………………….. (25)

Figure 26. ^{1}H NMR spectrum of (1*H*-1,2,3-triazol-4-yl)methyl benzoate (**9a**)……………………..(25)

Figure 27. ^{13}C NMR spectrum of (1*H*-1,2,3-triazol-4-yl)methyl benzoate (**9a**)……………………..(26)

Figure 28. FT-IR spectrum of (1-allyl-1*H*-1,2,3-triazol-4-yl)methyl 4-chlorobenzoate (**10a**)…….. (26)

Figure 29. ^{1}H NMR spectrum of (1-allyl-1*H*-1,2,3-triazol-4-yl)methyl 4-chlorobenzoate (**10a**)….. (27)

Figure 30. ^{13}C NMR spectrum of (1-allyl-1*H*-1,2,3-triazol-4-yl)methyl 4-chlorobenzoate (**10a**)…...(27)

Figure 31. FT-IR spectrum of (1-benzyl-1*H*-1,2,3-triazol-4-yl)methyl 4-methylbenzoate (**11a**)….. (28)

Figure 32. ^{1}H NMR spectrum of (1-benzyl-1*H*-1,2,3-triazol-4-yl)methyl 4-methylbenzoate (**11a**) ..(28)

Figure 33. ^{13}C NMR spectrum of (1-benzyl-1*H*-1,2,3-triazol-4-yl)methyl 4-methylbenzoate (**11a**)..(29)

Figure 34. FT-IR spectrum of 1-allyl-4-((4-chlorophenoxy)methyl)-1*H*-1,2,3-triazole (**12a**) ……..(29)

Figure 35. ^{1}H NMR spectrum of 1-allyl-4-((4-chlorophenoxy)methyl)-1*H*-1,2,3-triazole (**12a**)….. (30)

Figure 36. ^{13}C NMR spectrum of 1-allyl-4-((4-chlorophenoxy)methyl)-1*H*-1,2,3-triazole (**12a**)…. (30)

Figure 37. FT-IR spectrum of 1-benzyl-4-((4-bromophenoxy)methyl)-1*H*-1,2,3-triazole (**13a**) …..(31)

Figure 38. ^{1}H NMR spectrum of 1-benzyl-4-((4-bromophenoxy)methyl)-1*H*-1,2,3-triazole (**13a**) ..(31)

Figure 39. ^{13}C NMR spectrum of 1-benzyl-4-((4-bromophenoxy)methyl)-1*H*-1,2,3-triazole (**13a**). (32)

Figure 40. FT-IR spectrum of 1-allyl-4-(((7-methoxynaphthalen-2-yl)oxy)methyl)-1*H*-1,2,3-triazole (**14a**)…………………………………………………………………………………………….(32)

Figure 41. ^{1}H NMR spectrum of 1-allyl-4-(((7-methoxynaphthalen-2-yl)oxy)methyl)-1*H*-1,2,3-triazole (**14a**)…………………………………………………………………………………………... (33)

Figure 42. ^{13}C NMR spectrum of 1-allyl-4-(((7-methoxynaphthalen-2-yl)oxy)methyl)-1*H*-1,2,3-triazole (**14a**)……………………………………………………………………………...… (33)

Figure 43. FT-IR spectrum of 1-benzyl-4-(((7-methylnaphthalen-2-yl)oxy)methyl)-1*H*-1,2,3-triazole (**15a**)……………………………………………………………………………………………….(34)

Figure 44. ^{1}H NMR spectrum of 1-benzyl-4-(((7-methylnaphthalen-2-yl)oxy)methyl)-1*H*-1,2,3-triazole (**15a**)………………………………………………………………………………... (34)

Figure 45. ^{13}C NMR spectrum of 1-benzyl-4-(((7-methylnaphthalen-2-yl)oxy)methyl)-1*H*-1,2,3-triazole (**15a**)………………………………………………………………………………... (35)

Figure 46. FT-IR spectrum of 1-allyl-4-((4-phenoxyphenoxy)methyl)-1*H*-1,2,3-triazole (**16a**)…… (35)

Figure 47. ^{1}H NMR spectrum of 1-allyl-4-((4-phenoxyphenoxy)methyl)-1*H*-1,2,3-triazole (**16a**) …(36)

Figure 48. ^{13}C NMR spectrum of 1-allyl-4-((4-phenoxyphenoxy)methyl)-1*H*-1,2,3-triazole (**16a**)...(36)

Figure 49. FT-IR spectrum of 1-benzyl-4-((4-benzylphenoxy)methyl)-1*H*-1,2,3-triazole (**17a**)….. (37)

Figure 50. ^{1}H NMR spectrum of 1-benzyl-4-((4-benzylphenoxy)methyl)-1*H*-1,2,3-triazole (**17a**).. (37)

Figure 51. ^{13}C NMR spectrum of 1-benzyl-4-((4-benzylphenoxy)methyl)-1*H*-1,2,3-triazole (**17a**)..(38)

Figure 52. FT-IR spectrum of 1-allyl-4-((4-benzylphenoxy)methyl)-1*H*-1,2,3-triazole (**18a**) ……..(38)

Figure 53. ^{1}H NMR spectrum of 1-allyl-4-((4-benzylphenoxy)methyl)-1*H*-1,2,3-triazole (**18a**) ….(39)

Figure 54. ^{13}C NMR spectrum of 1-allyl-4-((4-benzylphenoxy)methyl)-1*H*-1,2,3-triazole (**18a**)….(39)

Figure 55. FT-IR spectrum of 1-benzyl-4-((4-nitrophenoxy)methyl)-1*H*-1,2,3-triazole (**19a**) …..…(40)

Figure 56. ^{1}H NMR spectrum of 1-benzyl-4-((4-nitrophenoxy)methyl)-1*H*-1,2,3-triazole (**19a**)….(40)

Figure 57. ^{13}C NMR spectrum of 1-benzyl-4-((4-nitrophenoxy)methyl)-1*H*-1,2,3-triazole (**19a**)…(41)

Figure 58. FT-IR spectrum of 1-allyl-4-((3-nitrophenoxy)methyl)-1*H*-1,2,3-triazole (**20a**)……….(41)

Figure 59. ^{1}H NMR spectrum of 1-allyl-4-((3-nitrophenoxy)methyl)-1*H*-1,2,3-triazole (**20a**)…….(42)

Figure 60. ^{13}C NMR spectrum of 1-allyl-4-((3-nitrophenoxy)methyl)-1*H*-1,2,3-triazole (**20a**)…….(42)

Figure 61. FT-IR spectrum of 5-phenyl-1*H*-tetrazole (**21a**)…………………… ……………..…(43)

Figure 62. ^{1}H NMR spectrum of 5-phenyl-1*H*-tetrazole (**21a**)…………………… ……….……(43)

Figure 63. ^{13}C NMR spectrum of 5-phenyl-1*H*-tetrazole (**21a**)…………………… ……………..(44)

Figure 64. FT-IR spectrum of 4-(1*H*-tetrazol-5-yl)phenol (**22a**)…………………… ……………(44)

Figure 65. ^{1}H NMR spectrum of 4-(1*H*-tetrazol-5-yl)phenol (**22a**)………………… ……………..(45)

Figure 66. ^{13}C NMR spectrum of 4-(1*H*-tetrazol-5-yl)phenol (**22a**)………………… ……….….(45)

Figure 67. FT-IR spectrum of 5-(4-(trifluoromethyl)phenyl)-1*H*-tetrazole (**23a**)...………… ...……(46)

Figure 68. ^{1}H NMR spectrum of 5-(4-(trifluoromethyl)phenyl)-1*H*-tetrazole (**23a**)………………..(46)

Figure 69. ^{13}C NMR spectrum of 5-(4-(trifluoromethyl)phenyl)-1*H*-tetrazole (**23a**)…… ………….(47)

Figure 70. FT-IR spectrum of 5-(4-chlorophenyl)-1*H*-tetrazole (**24a**)…………………….. ……(47)

Figure 71. ^{1}H NMR spectrum of 5-(4-chlorophenyl)-1*H*-tetrazole (**24a**) …………………………..(48)

Figure 72. ^{13}C NMR spectrum of 5-(4-chlorophenyl)-1*H*-tetrazole (**24a**)………………… ………..(48)

Figure 73. FT-IR spectrum of 5-(p-tolyl)-1*H*-tetrazole (**25a**)………………………….. ……..…...(49)

Figure 74. ^{1}H NMR spectrum of 5-(p-tolyl)-1*H*-tetrazole (**25a**)……………………….. ………….(49)

Figure 75. ^{13}C NMR spectrum of 5-(p-tolyl)-1*H*-tetrazole (**25a**)………………………… ..….…(50)

Figure 76. FT-IR spectrum of 5-(4-nitrophenyl)-1*H*-tetrazole (**26a**)……………………………….(50)

Figure 77. ^{1}H NMR spectrum of 5-(4-nitrophenyl)-1*H*-tetrazole (**26a**)………………… ……....…(51)

Figure 78. ^{13}C NMR spectrum of 5-(4-nitrophenyl)-1*H*-tetrazole (**26a**)…………………... ……(51)

Figure 79. FT-IR spectrum of 4-(1*H*-tetrazol-5-yl)pyridine (**27a**) ………………………………….(52)

Figure 80. ^{1}H NMR spectrum of 4-(1*H*-tetrazol-5-yl)pyridine (**27a**)…………………. ……..…..(52)

Figure 81. ^{13}C NMR spectrum of 4-(1*H*-tetrazol-5-yl)pyridine (**27a**)…………………… ………(53)

Figure 82. FT-IR spectrum of 5-benzyl-1*H*-tetrazole (**28a**)……………………………. …………(53)

Figure 83. ^{1}H NMR spectrum of 5-benzyl-1*H*-tetrazole (**28a**)……………………………………. …(54)

Figure 84. ^{13}C NMR spectrum of 5-benzyl-1*H*-tetrazole (**28a**)…………………….……… ……..(54)

Figure 85. FT-IR spectrum of 5-(4-bromophenyl)-1*H*-tetrazole (**29a**)…………………… ….…(55)

Figure 86. ^{1}H NMR spectrum of 5-(4-bromophenyl)-1*H*-tetrazole (**29a**)………………… …..…(55)

Figure 87. ^{13}C NMR spectrum of 5-(4-bromophenyl)-1*H*-tetrazole (**29a**)…………………… …..(56)

Figure 88. FT-IR spectrum of 5-(o-tolyl)-1*H*-tetrazole (**30a**)……………………………………..(56)

Figure 89. ^{1}H NMR spectrum of 5-(o-tolyl)-1*H*-tetrazole (**30a**)………………………… ……….(57)

Figure 90. ^{13}C NMR spectrum of 5-(o-tolyl)-1*H*-tetrazole (**30a**)…………………………… ……(57)

4-Phenyl-1*H*-1,2,3-triazole (1a)

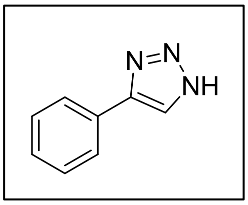

White solid; Mp: 152-154 °C (Lit. 150-151 °C)[37]; Yield: 95 %. IR (KBr), ῡ(cm^{-1}): 3472, 3419, 3122, 3096, 1607, 1467, 1455, 1358, 1224, 1075, 766, 729, 694; ^{1}H NMR (400 MHz, DMSO-d_6), δ (ppm): 5.66 (s, 1 H), 7.31-7.46 (m, 4 H, Ar-H), 7.87 (d, 1 H, *J* = 8 Hz, Ar-H), 8.66 (s, 1 H); ^{13}C NMR (100 MHz, DMSO-d_6), δ (ppm): 125.64, 128.36, 129.28, 129.36, 131.15, 147.64. Elemental Analysis: $C_8H_7N_3$: calcd: C, 66.19; H, 4.86; N, 28.95. Found: C, 66.00; H, 4.75; N, 28.98.

(1*H*-1,2,3-Triazol-4-yl)methyl 4-methoxybenzoate (2a)

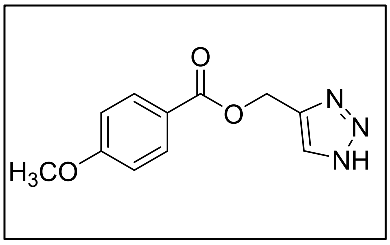

White solid; Mp: 211-213 °C; Yield: 75 %. IR (KBr), ῡ(cm^{-1}): 3307, 2971, 2932, 2847, 1712, 1702, 1606, 1515, 1321, 1260, 1169, 1108, 1021, 846, 769, 617; ^{1}H NMR (400 MHz, DMSO-d_6), δ (ppm): 3.84 (s, 3 H, OCH_3), 4.74 (s, 2 H, CH_2), 5.05 (s, 1 H, CH), 7.05 (d, 2 H, *J* = 8 Hz, Ar-H), 7.95 (d, 2 H, *J* = 8 Hz, Ar-H), 10.58 (s, 1 H, NH); ^{13}C NMR (100 MHz, DMSO-d_6), δ (ppm): 47.62, 57.27, 117.71, 123.94, 126.07, 128.49, 141.41, 162.69, 167.03. Elemental Analysis: $C_{11}H_{11}N_3O_3$: calcd: C, 56.65; H, 4.75; N, 18.02; O, 20.58. Found: C, 57.00; H, 4.58; N, 18.00.

(1*H*-1,2,3-Triazol-4-yl)methyl 4-nitrobenzoate (3a)

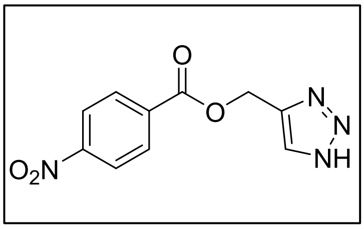

White solid; Mp: 242-244 °C; Yield: 78 %. IR (KBr), ῡ(cm^{-1}): 3538, 3425, 3389, 1727, 1606, 1519, 1352, 1292, 1280, 1112, 961, 719, 507; ^{1}H NMR (400 MHz, DMSO-d_6), δ (ppm): 4.78 (s, 2 H, CH_2), 5.14 (s, 1 H, CH), 8.23 (d, 2 H, *J* = 8 Hz, Ar-H), 8.37 (d, 2 H, *J* = 8 Hz, Ar-H), 10.86 (s, 1 H, NH); ^{13}C NMR (100 MHz, DMSO-d_6), δ (ppm): 56.44, 119.63, 126.22, 135.98, 142.88, 149.09, 159.39, 167.66. Elemental Analysis: $C_{10}H_8N_4O_4$: calcd: C, 48.39; H, 3.25; N, 22.57; O, 25.78. Found: C, 48.21; H, 3.15; N, 22.38.

(1*H*-1,2,3-Triazol-4-yl)methyl 4-(dimethylamino)benzoate (4a)

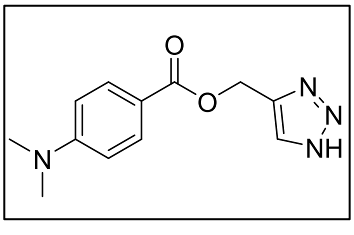

White solid; Mp: 194-196 °C; Yield: 73 %. IR (KBr), ῡ(cm^{-1}): 3473, 2920, 2824, 1678, 1617, 1377, 1291, 1192, 1129, 1112, 1041, 827, 768; ^{1}H NMR (400 MHz, DMSO-d_6), δ (ppm): 3.01 (s, 6 H, $2CH_3$), 4.70 (s, 2 H, CH_2), 4.99 (s, 1 H, CH), 6.73 (d, 2 H, *J* = 8 Hz, Ar-H), 7.80 (d, 2 H, *J* = 8 Hz, Ar-H), 10.75 (s, 1 H, NH); ^{13}C NMR (100 MHz, DMSO-d_6), δ (ppm): 44.27, 56.76, 112.62, 118.08, 126.03, 136.87, 143.34, 155.84, 165.10. Elemental Analysis: $C_{12}H_{14}N_4O_2$: calcd: C, 58.53; H, 5.73; N, 22.75; O, 12.99. Found: C, 58.60; H, 5.73; N, 22.65.

(1*H*-1,2,3-Triazol-4-yl)methyl 2-nitrobenzoate (5a)

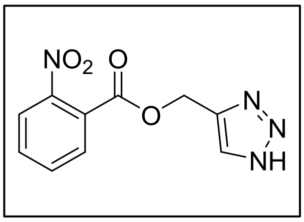

White solid; Mp: 268-270 °C; Yield: 68 %. IR (KBr), ῡ(cm^{-1}): 3551, 3742, 3414, 3243, 2398, 2332, 1682, 1629, 1378, 1310, 1292, 1193, 1113, 1068, 945, 762, 621, 524; ^{1}H NMR (400 MHz, DMSO-*d*$_6$), δ (ppm): 4.76 (s, 2 H, CH_2), 5.12 (s, 1 H, CH), 8.23 (d, 2 H, *J* = 8 Hz, Ar-H), 8.37 (t, 3 H, *J* = 8 Hz, Ar-H), 11.02 (s, 1 H, NH); ^{13}C NMR (100 MHz, DMSO-*d*$_6$), δ (ppm): 56.44, 119.63, 126.22, 129.27, 130.52, 133.58, 135.98, 142.88, 149.04, 167.66. Elemental Analysis: $C_{10}H_8N_4O_4$: calcd: C, 48.39; H, 3.25; N, 22.57; O, 25.78. Found: C, 48.00; H, 2.99; N, 22.41.

(1*H*-1,2,3-Triazol-4-yl)methyl 2-methylbenzoate (6a)

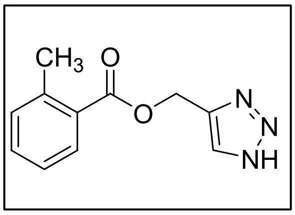

White solid; Mp: 198-200 °C; Yield: 71 %. IR (KBr), ῡ(cm^{-1}): 3422, 3243, 2953, 2123, 1719, 1662, 1595, 1578, 1448, 1434, 1370, 1317, 1262, 1153, 1135, 1080, 975, 937, 924, 741, 700, 686, 637; ^{1}H NMR (400 MHz, DMSO-*d*$_6$), δ (ppm): 3.44 (s, 3 H, CH_3), 4.75 (s, 2 H, CH_2), 5.04 (s, 1 H, CH), 7.04-7.07 (m, 3 H, Ar-H), 7.94-7.97 (m, 1 H, Ar-H), 10.69 (s, 1 H, NH); ^{13}C NMR (100 MHz, DMSO-*d*$_6$), δ (ppm): 27.24, 57.27, 123.94, 126.07, 128.49, 132.05, 133.26, 137.05, 141.41, 143.34, 163.90. Elemental Analysis: $C_{11}H_{11}N_3O_2$: calcd: C, 60.82; H, 5.10; N, 19.34; O, 14.73. Found: C, 60.75; H, 4.99; N, 19.50.

(1*H*-1,2,3-Triazol-4-yl)methyl 3,4-dichlorobenzoate (7a)

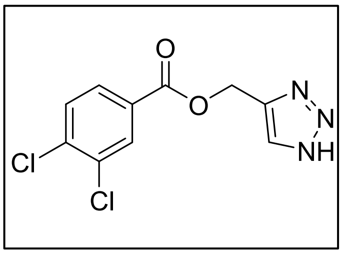

White solid; Mp: 222-224 °C; Yield: 69 %. IR (KBr), ῡ(cm^{-1}): 3393, 3090, 2962, 2678, 1723, 1590, 1546, 1416, 1384, 1295, 1274, 1234, 1115, 1030, 843, 773, 756, 675, 570; ^{1}H NMR (400 MHz, DMSO-*d*$_6$), δ (ppm): 4.33 (s, 2 H, CH_2), 4.35 (s, 1 H, CH), 7.82 (d, 1 H, *J* = 8 Hz, Ar-H), 7.94 (d, 1 H, *J* = 8 Hz, Ar-H), 7.95 (s, 1 H, Ar-H), 11.32 (s, 1 H, NH); ^{13}C NMR (100 MHz, DMSO-*d*$_6$), δ (ppm): 62.92, 129.79, 130.39, 130.81, 131.41, 131.64, 132.13, 136.70, 143.72, 164.46. Elemental Analysis: $C_{10}H_7Cl_2N_3O_2$: calcd: C, 44.14; H, 2.59; Cl, 26.06; N, 15.44; O, 11.76. Found: C, 44.00; H, 2.63; N, 15.41.

(1*H*-1,2,3-Triazol-4-yl)methyl 3-methoxybenzoate (8a)

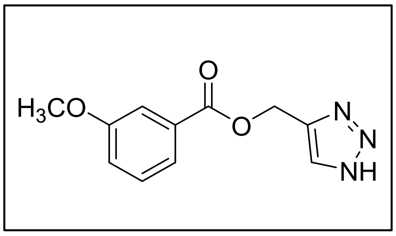

White solid; Mp: 190-192 °C; Yield: 75 %. IR (KBr), ῡ(cm^{-1}): 3413, 2977, 2932, 2352, 1710, 1703, 1606, 1515, 1455, 1356, 1321, 1261, 1169, 1108, 1022, 970, 882, 847, 762, 617; ^{1}H NMR (400 MHz, DMSO-*d*$_6$), δ (ppm): 3.87 (s, 3 H, OCH_3), 4.61 (s, 2 H, CH_2), 5.45 (s, 1 H, CH), 7.34 (s, 1 H, Ar-H), 7.47-7.54 (m, 3 H, Ar-H), 10.46 (s, 1 H, NH); ^{13}C NMR (100 MHz, DMSO-*d*$_6$), δ (ppm): 46.14, 57.59, 114.75, 118.29, 121.71, 129.11, 130.68, 131.37, 141.38, 160.58, 165.10. Elemental Analysis: $C_{11}H_{11}N_3O_3$: calcd: C, 56.65; H,

4.75; N, 18.02; O, 20.58. Found: C, 56.61; H, 4.38; N, 17.91.

(1*H*-1,2,3-Triazol-4-yl)methyl benzoate (9a)

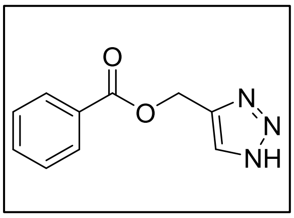

White solid; Mp: 135-137 °C; Yield: 72 %. IR (KBr), ῡ(cm^{-1}): 3547, 3479, 3415, 3250, 2127, 1706, 1640, 1427, 1370, 1280, 1262, 1174, 1126, 996, 966, 863, 744, 604; ^{1}H NMR (400 MHz, DMSO-d_6), δ (ppm): 4.66 (s, 2 H, CH_2), 5.25 (s, 1 H, CH), 7.47-7.61 (m, 3 H, Ar-H), 8.11 (d, 2 H, *J*= 8 Hz, Ar-H), 10.40 (s, 1 H, NH); ^{13}C NMR (100 MHz, DMSO-d_6), δ (ppm): 61.08, 128.18, 129.56, 130.73, 131.14, 133.21, 142.39, 164.97. Elemental Analysis: $C_{10}H_9N_3O_2$: calcd: C, 59.11; H, 4.46; N, 20.68; O, 15.75. Found: C, 58.98; H, 4.63; N, 20.53.

(1-Allyl-1*H*-1,2,3-triazol-4-yl)methyl 4-chlorobenzoate (10a)

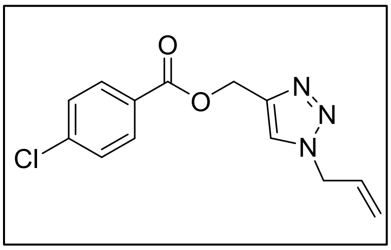

White solid; Mp: 118-120 °C; Yield: 45 %. IR (KBr), ῡ(cm^{-1}): 3428, 3141, 2938, 1622, 1459, 1388, 1255, 1214, 1205, 1184, 1002, 837; ^{1}H NMR (400 MHz, $CDCl_3$), δ (ppm): 5.01 (d, 2 H, *J*= 8 Hz), 5.13 (s, 2 H), 5.28-5.35 (dd, 2 H, *J*= 16, 12 Hz), 5.42 (s, 1 H), 5.75-5.88 (m, 1 H), 6.51 (d, 2 H, *J*= 8 Hz), 7.17 (d, 2 H, *J*= 8 Hz); ^{13}C NMR (100 MHz, $CDCl_3$), δ (ppm): 55.36, 60.37, 119.83, 127.60, 129.29, 130.88, 131.73, 133.33, 137.44, 142.66, 165.47. Elemental Analysis: $C_{13}H_{12}ClN_3O_2$: calcd: C, 56.23; H, 4.36; Cl, 12.77; N, 15.13; O, 11.52. Found: C, 57.00; H, 3.92; N, 15.12.

(1-Benzyl-1*H*-1,2,3-triazol-4-yl)methyl 4-methylbenzoate (11a)

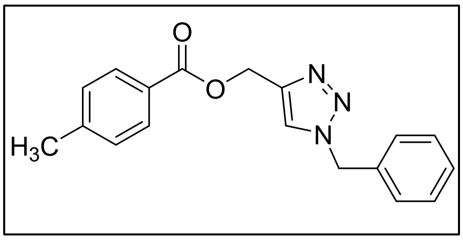

White solid; Mp: 120-122 °C; Yield: 50 %. IR (KBr), ῡ(cm^{-1}): 3419, 3243, 2953, 2342, 1708, 1636, 1430, 1371, 1210, 1188, 1170, 1099, 1029, 846, 770, 607, 532; ^{1}H NMR (400 MHz, $CDCl_3$), δ (ppm): 2.54 (s, 3 H), 5.35 (s, 2 H), 5.46 (s, 2 H), 5.62 (s, 1 H), 6.95 (d, 2 H, *J*= 8 Hz), 7.27-7.30 (m, 3 H), 7.60 (d, 2 H, *J*= 8 Hz), 7.93 (d, 2 H, *J*= 8 Hz); ^{13}C NMR (100 MHz, $CDCl_3$), δ (ppm): 21.76, 54.73, 57.97, 123.90, 126.09, 127.24, 128.38, 128.72, 129.13, 129.57, 132.55, 143.22, 144.10, 166.75. Elemental Analysis: $C_{18}H_{17}N_3O_2$: calcd: C, 70.34; H, 5.58; N, 13.67; O, 10.41. Found: C, 70.22; H, 5.23; N, 14.00.

1-Allyl-4-((4-chlorophenoxy)methyl)-1*H*-1,2,3-triazole (12a)

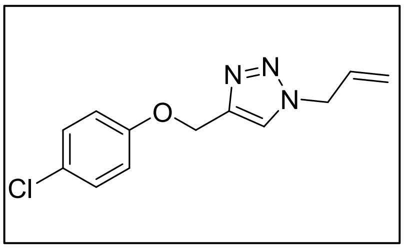

White solid; Mp: 104-106 °C; Yield: 88 %. IR (KBr), ῡ(cm^{-1}): 3419, 3200, 3113, 1724, 1607, 1525, 1349, 1272, 1245, 1120, 1105, 958, 876, 719, 635, 530; ^{1}H NMR (400 MHz, $CDCl_3$), δ (ppm): 4.99 (d, 2 H, *J* = 8 Hz), 5.19 (s, 2 H), 5.30-5.39 (dd, 2 H, *J* = 16, 12 Hz), 5.98-6.08 (m, 1 H), 6.88 (d, 2 H, *J* = 8 Hz), 7.38 (d, 2 H, *J* = 8 Hz), 7.64 (s, 1 H); ^{13}C NMR (100 MHz, $CDCl_3$), δ (ppm): 52.85, 62.22, 116.61, 120.88, 122.54, 128.14, 131.02, 132.36,

144.02, 157.29. Elemental Analysis: $C_{12}H_{12}ClN_3O$: calcd: C, 57.72; H, 4.84; Cl, 14.20; N, 16.83; O, 6.41. Found: C, 57.24; H, 4.68; N, 17.00.

1-Benzyl-4-((4-bromophenoxy)methyl)-1*H*-1,2,3-triazole (13a)

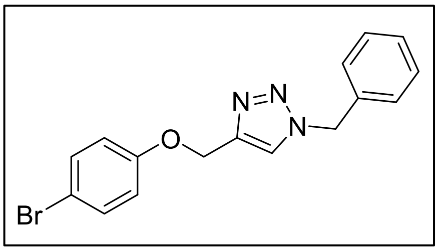

White solid; Mp: 132-134 ˚C (Lit. 131-133 ˚C)[42]; Yield: 90 %. IR (KBr), ῡ(cm^{-1}): 3426, 3121, 3096, 1615, 1464, 1446, 1418, 1223, 1076, 943, 765, 691; ^1H NMR (400 MHz, $CDCl_3$), δ (ppm): 5.07 (s, 2 H), 5.45 (s, 2 H), 6.78 (d, 2 H, J = 8 Hz), 7.20 (t, 2 H, J = 8 Hz), 7.27-7.34 (m, 5 H), 7.44 (s, 1 H); ^{13}C NMR (100 MHz, $CDCl_3$), δ (ppm): 52.92, 62.12, 113.51, 116.13, 116.64, 120.57, 122.70, 130.94, 132.36, 132.46, 143.90, 157.27. Elemental Analysis: $C_{16}H_{14}BrN_3O$: calcd: C, 55.83; H, 4.10; Br, 23.21; N, 12.21; O, 4.65. Found: C, 55.55; H, 4.13; N, 11.98.

1-Allyl-4-(((7-methoxynaphthalen-2-yl)oxy)methyl)-1*H*-1,2,3-triazole (14a)

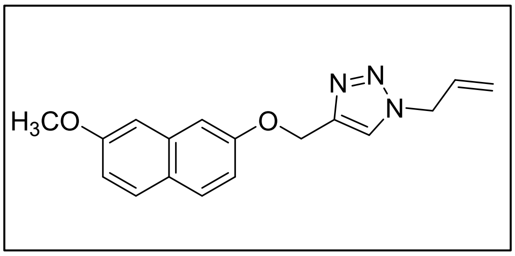

White solid; Mp: 101-103 ˚C (Lit. 102-104 ˚C)[43]; Yield: 80 %. IR (KBr), ῡ(cm^{-1}): 3428, 3141, 2938, 1622, 1459, 1388, 1255, 1214, 1205, 1184, 1002, 837; ^1H NMR (400 MHz, $CDCl_3$), δ (ppm): 3.91 (s, 3 H), 4.99 (dt, 2 H, J = 16, 12 Hz), 5.28-5.39 (m, 4 H), 5.95-6.11 (m, 1 H), 6.98-7.04 (m, 2 H), 7.06 (d, 1 H, J = 8 Hz), 7.19 (d, 1 H, J = 4 Hz), 7.63-7.68 (m, 3 H); ^{13}C NMR (100 MHz, $CDCl_3$), δ (ppm): 52.85, 55.31, 62.11, 105.31, 106.65, 116.15, 116.46, 120.43, 122.50, 124.47, 129.11, 129.26, 131.08, 135.77, 144.54, 156.74, 158.28. Elemental Analysis: $C_{17}H_{17}N_3O_2$: calcd: C, 69.14; H, 5.80; N, 14.23; O, 10.83. Found: C, 69.00; H, 5.92; N, 14.00.

1-Benzyl-4-(((7-methylnaphthalen-2-yl)oxy)methyl)-1*H*-1,2,3-triazole (15a)

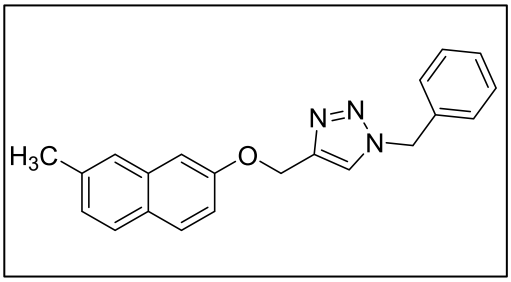

White solid; Mp: 142-144 ˚C; Yield: 83 %. IR (KBr), ῡ(cm^{-1}): 3435, 2928, 2400, 1620, 1254, 1214, 1130, 1009, 840, 748; ^1H NMR (400 MHz, $CDCl_3$), δ (ppm): 2.87 (s, 3 H), 5.23 (s, 2 H), 5.46 (s, 2 H), 6.91-6.98 (m, 3 H), 7.09-7.30 (m, 6 H), 7.48 (s, 1 H), 7.56-7.59 (m, 2 H); ^{13}C NMR (100 MHz, $CDCl_3$), δ (ppm): 23.71, 55.31, 62.14, 105.31, 106.71, 113.00, 116.14, 116.46, 122.61, 124.49, 128.14, 128.83, 129.10, 129.16, 129.26, 135.76, 144.71, 156.69, 158.23. Elemental Analysis: $C_{21}H_{19}N_3O$: calcd: C, 76.57; H, 5.81; N, 12.76; O, 4.86. Found: C, 76.24; H, 4.98; N, 13.00.

1-Allyl-4-((4-phenoxyphenoxy)methyl)-1*H*-1,2,3-triazole (16a)

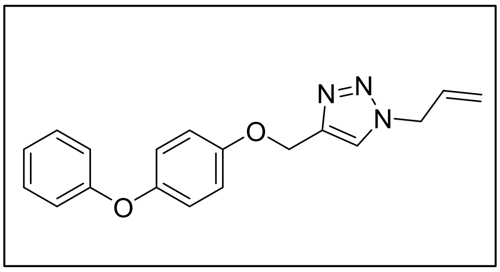

White solid; Mp: 100-102 °C (Lit. 99-102 °C)[43]; Yield: 78 %. IR (KBr), ῡ(cm^{-1}): 3434, 3140, 3102, 1590, 1504, 1489, 1210, 837, 691; ^1H NMR (400 MHz, $CDCl_3$), δ (ppm): 5.00 (d, 2 H, *J* = 8 Hz), 5.19-5.21 (m, 2 H), 5.29-5.39 (m, 2 H), 5.98-6.09 (m, 1 H), 6.93 (t, 1 H, *J* = 8 Hz), 6.95 -6.98 (m, 5 H), 7.26-7.33 (m, 3 H), 7.63 (s, 1 H); ^{13}C NMR (100 MHz, $CDCl_3$), δ (ppm): 52.82, 62.59, 115.90, 117.77, 120.39, 120.72, 122.50, 122.60, 129.65, 131.11, 144.45, 150.70, 154.45, 158.25. Elemental Analysis: $C_{18}H_{17}N_3O_2$: calcd: C, 70.34; H, 5.58; N, 13.67; O, 10.41. Found: C, 69.98; H, 5.68; N, 12.03.

1-Benzyl-4-((4-benzylphenoxy)methyl)-1*H*-1,2,3-triazole (17a)

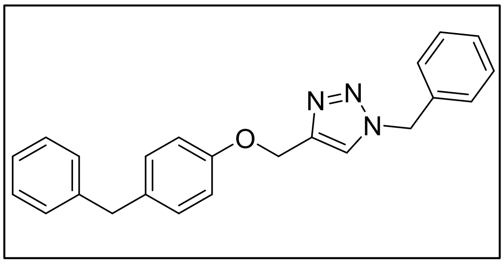

White solid; Mp: 104-106 °C (Lit. 101-102 °C)[44]; Yield: 85 %. IR (KBr), ῡ(cm^{-1}): 3433, 2926, 2873, 2373, 1498, 1232, 1217, 1007, 838, 702; ^1H NMR (400 MHz, $CDCl_3$), δ (ppm): 3.91 (s, 2 H), 5.15 (s, 2 H), 5.52 (s, 2 H), 6.88 (d, 2 H, *J* = 8 Hz), 7.07-7.21 (m, 6 H), 7.25-7.37 (m, 6 H), 7.50 (s, 1 H); ^{13}C NMR (100 MHz, $CDCl_3$), δ (ppm): 41.06, 54.26, 62.20, 114.78, 122.57, 126.04, 127.64, 128.14, 128.47, 128.84, 129.16, 129.93, 133.92, 134.49, 141.43, 144.79, 156.62. Elemental Analysis: $C_{23}H_{21}N_3O$: calcd: C, 77.72; H, 5.96; N, 11.82; O, 4.52. Found: C, 77.60; H, 5.69; N, 12.00.

1-Allyl-4-((4-benzylphenoxy)methyl)-1*H*-1,2,3-triazole (18a)

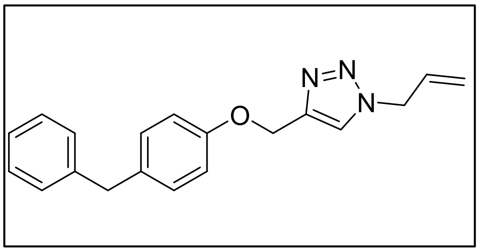

White solid; Mp: 60-62 °C (Lit. 59-61 °C)[44]; Yield: 83 %. IR (KBr), ῡ(cm^{-1}): 3481, 3128, 3082, 2919, 1607, 1508, 1493, 1243, 1223, 1019, 937, 696; ^1H NMR (400 MHz, $CDCl_3$), δ (ppm): 3.93 (s, 2 H), 4.97 (d, 2 H, *J* = 8 Hz), 5.18 (d, 2 H, *J* = 8 Hz), 5.27-5.38 (m, 2 H), 5.97-6.06 (m, 1 H), 6.92 (t, 2 H, *J* = 8 Hz), 7.08-7.31 (m, 7 H), 7.60 (s, 1 H); ^{13}C NMR (100 MHz, $CDCl_3$), δ (ppm): 42.21, 52.68, 62.31, 105.38, 120.13, 120.84, 122.08, 122.83, 125.33, 126.54, 127.56, 131.19, 134.55, 144.27, 154.00. Elemental Analysis: $C_{19}H_{19}N_3O$: calcd: C, 74.73; H, 6.27; N, 13.76; O, 5.24. Found: C, 74.62; H, 5.98; N, 13.43.

1-Benzyl-4-((4-nitrophenoxy)methyl)-1*H*-1,2,3-triazole (19a)

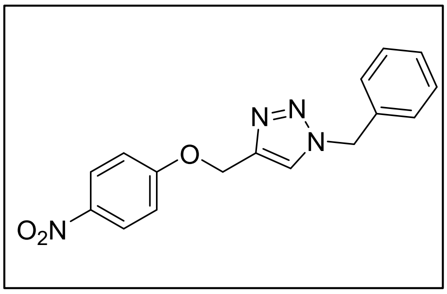

White solid; Mp: 104-106 °C (Lit. 102-104 °C)[45]; Yield: 92 %. IR (KBr), ῡ(cm^{-1}): 3421, 3147, 3116, 1593, 1492, 1339, 1266, 1113, 999, 845, 696; ^1H

NMR (400 MHz, $CDCl_3$), δ (ppm): 5.41 (s, 2 H), 5.47(s, 2 H), 7.19-7.24 (m, 2 H), 7.30-7.32 (m, 3 H), 7.54 (s, 1 H), 8.05-8.21 (m, 4 H); ^{13}C NMR (100 MHz, $CDCl_3$), δ (ppm): 54.40, 62.46, 114.84, 112.94, 125.93, 128.18, 128.98, 129.23, 134.21, 141.87, 143.17, 163.08. Elemental Analysis: $C_{16}H_{14}N_3O_3$: calcd: C, 61.93; H, 4.55; N, 18.06; O, 15.47. Found: C, 61.55; H, 4.13; N, 18.00.

1-Allyl-4-((3-nitrophenoxy)methyl)-1*H*-1,2,3-triazole (20a)

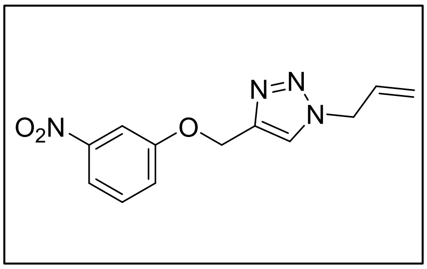

White solid; Mp: 204-206 ˚C; Yield: 90 %. IR (KBr), ῡ(cm^{-1}): 3421, 3153, 3013, 2929, 1595, 1508, 1340, 1262, 1111, 1040, 850, 638; ^1H NMR (400 MHz, $CDCl_3$), δ (ppm): 4.93 (d, 2 H, *J* = 8 Hz), 5.23-5.33 (m, 4 H), 5.88-6.04 (m, 1 H), 6.97-7.04 (m, 2 H), 7.60 (s, 2 H), 8.13 (d, 1 H, *J* = 8 Hz); ^{13}C NMR (100 MHz, $CDCl_3$), δ (ppm): 52.90, 62.47, 112.69, 114.80, 120.63, 122.86, 125.93, 130.905, 141.86, 143.00, 152.58, 163.12. Elemental Analysis: $C_{12}H_{12}N_4O_3$: calcd: C, 55.38; H, 4.65; N, 21.53; O, 18.44. Found: C, 54.99; H, 4.16; N, 21.01.

5-Phenyl-1*H*-tetrazole (21a)

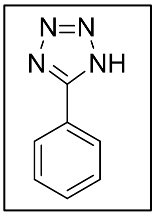

White solid; Mp: 211-213 ˚C (Lit. 212-214 ˚C)[46]; Yield: 97 %. IR (KBr), ῡ(cm^{-1}): 3437, 3129, 3056, 2981, 2863, 2702, 2608, 2553, 2482, 1869, 1608, 1564, 1486, 1466, 1410, 1164, 1084, 993, 704; ^1H NMR (400 MHz, DMSO-d_6), δ (ppm): 7.60-7.64 (m, 3 H), 8.04-8.06 (m, 2 H), 9.38 (s, 1 H, NH); ^{13}C NMR (100 MHz, DMSO-d_6), δ (ppm): 124.58, 127.43, 129.91, 131.75, 155.83. Elemental Analysis: $C_7H_6N_4$: calcd: C, 57.53; H, 4.14; N, 38.34. Found: C, 57.39; H, 4.16; N, 38.21.

4-(1*H*-Tetrazol-5-yl)phenol (22a)

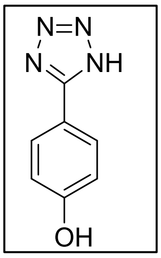

White solid; Mp: 235-236 ˚C (Lit. 234-235 ˚C)[47]; Yield: 92 %. IR (KBr), ῡ(cm^{-1}): 3362, 3129, 2848, 2747, 2632, 1613, 1598, 1505, 1408, 1292, 1267, 1182, 844; ^1H NMR (400 MHz, DMSO-d_6), δ (ppm): 4.77 (brs, 1 H, OH), 6.95 (d, 2 H, *J* = 8Hz), 7.87 (d, 2 H, *J* = 8Hz), 9.78 (s, 1 H, NH); ^{13}C NMR (100 MHz, DMSO-d_6), δ (ppm): 115.50, 116.55, 129.15, 155.59, 160.43. Elemental Analysis: $C_7H_6N_4O$: calcd: C, 51.85; H, 3.73; N, 34.55; O, 9.87. Found: C, 51.79; H, 3.56; N, 34.

5-(4-(Trifluoromethyl)phenyl)-1*H*-tetrazole (23a)

White solid; Mp: 219-221 ˚C (Lit. 220-222 ˚C)[48]; Yield: 97 %. IR (KBr), ῡ(cm^{-1}): 3432, 3073, 2989, 2926, 2836, 2686, 2610, 2476, 1905, 1574, 1442, 1331, 1163, 1134, 1073, 990, 848; ^1H NMR (400 MHz, DMSO-d_6), δ (ppm): 7.97 (d, 2 H, *J* = 8Hz), 8.25 (d, 2 H, *J* = 8Hz), 10.77 (s, 1 H, NH); ^{13}C NMR (100 MHz, DMSO-d_6), δ (ppm): 120.21, 122.92, 125.63, 126.76, 126.80 (d, *J* = 16 Hz), 126.84, 126.87 (d, *J* = 12 Hz), 128.18, 128.34, 128.86, 130.93, 131.25, 131.57, 131.89, 155.73. Elemental

Analysis: $C_8H_5F_3N_4$: calcd: C, 44.87; H, 2.35; F, 26.61; N, 26.16. Found: C, 45.00; H, 2.34; N, 26.10.

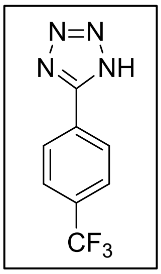

5-(4-Chlorophenyl)-1*H*-tetrazole (24a)

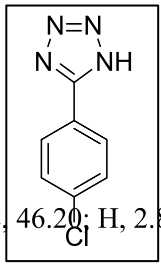

White solid; Mp: 263-265 °C (Lit. 261-263 °C)[49]; Yield: 94 %. IR (KBr), ῡ(cm^{-1}): 3473, 3095, 3070, 2998, 2909, 2851, 2732, 2637, 1718, 1609, 1564, 1486, 1435, 1407, 1160, 1095, 1054, 831, 745; ^{1}H NMR (400 MHz, DMSO-d_6), δ (ppm): 7.67 (d, 2 H, J = 8 Hz), 8.05 (d, 2 H, J = 8 Hz), 9.32 (s, 1 H, NH); ^{13}C NMR (100 MHz, DMSO-d_6), δ (ppm): 123.63, 129.15, 129.99, 136.37, 155.35. Elemental Analysis: $C_7H_5ClN_4$: calcd: C, 46.56; H, 2.79; Cl, 16.63; N, 31.02. Found: C, 46.20; H, 2.84; N, 30.98.

5-(*p*-Tolyl)-1*H*-tetrazole (25a)

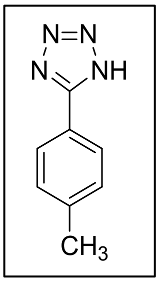

White solid; Mp: 252-254 °C (Lit. 250-251 °C)[50]; Yield: 92 %. IR (KBr), ῡ(cm^{-1}): 3317, 2995, 2439, 1630, 1510, 1484, 1161, 745, 616; ^{1}H NMR (400 MHz, DMSO-d_6), δ (ppm): 1.78 (s, 3 H), 6.90-6.93 (m, 2 H), 7.37-7.41 (m, 2 H), 10.48 (s, 1 H, NH); ^{13}C NMR (100 MHz, DMSO-d_6), δ (ppm): 28.19, 119.31, 125.38, 130.92, 133.83, 155.53. Elemental Analysis: $C_8H_8N_4$: calcd: C, 59.99; H, 5.03; N, 34.98. Found: C, 59.80; H, 5.00; N, 34.90.

5-(4-Nitrophenyl)-1*H*-tetrazole (26a)

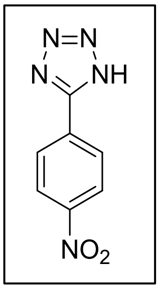

Yellow solid; Mp: 216-218 °C (Lit. 218-220 °C)[46]; Yield: 96 %. IR (KBr), ῡ(cm^{-1}): 3585, 3382, 3125, 2769, 2470, 1604, 1514, 1340, 1110, 856, 731; ^{1}H NMR (400 MHz, DMSO-d_6), δ (ppm): 8.24 (d, 2 H, J = 8 Hz), 8.29 (d, 2 H, J = 8 Hz), 10.45 (s, 1 H, NH); ^{13}C NMR (100 MHz, DMSO-d_6), δ (ppm): 124.58, 126.88, 138.81, 146.72, 159.65. Elemental Analysis: $C_7H_5N_5O_2$: calcd: C, 43.98; H, 2.64; N, 36.64; O, 16.74. Found: C, 43.82; H, 2.53; N, 36.70.

4-(1*H*-Tetrazol-5-yl)pyridine (27a)

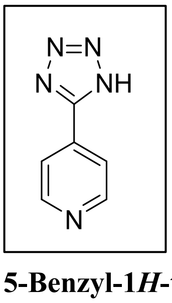

White solid; Mp: 256-258 °C (Lit. 254-258 °C)[51]; Yield: 92 %. IR (KBr), ῡ(cm^{-1}): 3362, 3259, 3100, 2927, 2445, 2365, 2106, 2018, 1910, 1719, 1618, 1420, 1388, 1042, 842; ^{1}H NMR (400 MHz, DMSO-d_6), δ (ppm): 6.30 (m, 2 H), 7.99 (d, 2 H, J = 8 Hz), 8.72 (s, 1 H, NH); ^{13}C NMR (100 MHz, DMSO-d_6), δ (ppm): 121.10, 136.41, 150.54, 157.48. Elemental Analysis: $C_6H_5N_5$: calcd: C, 48.98; H, 3.43; N, 47.60. Found: C, 48.90; H, 3.38; N, 47.48.

5-Benzyl-1*H*-tetrazole (28a)

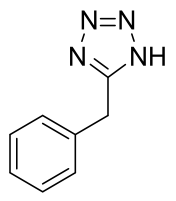

White solid; Mp: 124-126 °C (Lit. 123-125 °C)[49]; Yield: 90 %. IR (KBr), ῡ(cm^{-1}): 3420, 3108, 2860, 2778, 2600, 1549, 1533, 1457, 1252, 1084, 1057, 891, 734, 595; ^{1}H NMR (400 MHz, DMSO-d_6), δ (ppm): 4.30 (s, 2 H), 7.25-7.36 (m, 5 H), 9.20 (s, 1 H, NH); ^{13}C NMR (100 MHz, DMSO-d_6), δ (ppm): 29.35, 127.47, 129.12, 129.18, 136.42, 155.70. Elemental Analysis: $C_8H_8N_4$: calcd: C, 59.99; H, 5.03; N, 34.98. Found: C, 59.82; H, 5.00; N, 34.90.

5-(4-Bromophenyl)-1*H*-tetrazole (29a)

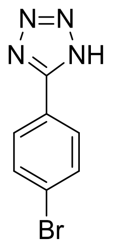

White solid; Mp: 268-270 °C (Lit. 268-270 °C)[52]; Yield: 94 %. IR (KBr), ῡ(cm^{-1}): 3442, 3119, 2901, 2847, 2769, 2729, 2634, 1608, 1566, 1482, 1431, 1277, 1157, 1054, 988, 828, 743; ^{1}H NMR (400 MHz, DMSO-d_6), δ (ppm): 7.79 (d, 2 H, *J* = 8 Hz), 7.97 (d, 2 H, *J* = 8 Hz), 9.77 (s, 1 H, NH); ^{13}C NMR (100 MHz, DMSO-d_6), δ (ppm): 124.12, 125.10, 129.29, 132.89, 155.53. Elemental Analysis: $C_7H_5BrN_4$: calcd: C, 37.36; H, 2.24; Br, 35.51; N, 24.90. Found: C, 37.31; H, 2.00; N, 24.70.

5-(*o*-Tolyl)-1*H*-tetrazole (30a)

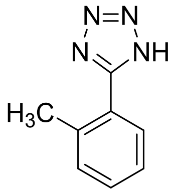

White solid; Mp: 152-154 °C (Lit. 153-155 °C)[40]; Yield: 92 %. IR (KBr), ῡ(cm^{-1}): 3129, 2963, 2824, 2715, 2607, 1832, 1607, 1563, 1485, 1464, 1386, 1251, 1160, 1060, 1049, 991, 782, 746; ^{1}H NMR (400 MHz, DMSO-d_6), δ (ppm): 2.50 (s, 3 H), 7.38-7.46 (m, 3 H), 7.48-7.70 (m, 1 H), 10.11 (s, 1 H, NH); ^{13}C NMR (100 MHz, DMSO-d_6), δ (ppm): 20.55, 124.32, 126.77, 129.87, 131.19, 131.80, 137.59, 155.74. Elemental Analysis: $C_8H_8N_4$: calcd: C, 59.99; H, 5.03; N, 34.98. Found: C, 59.90; H, 5.07; N, 34.87.

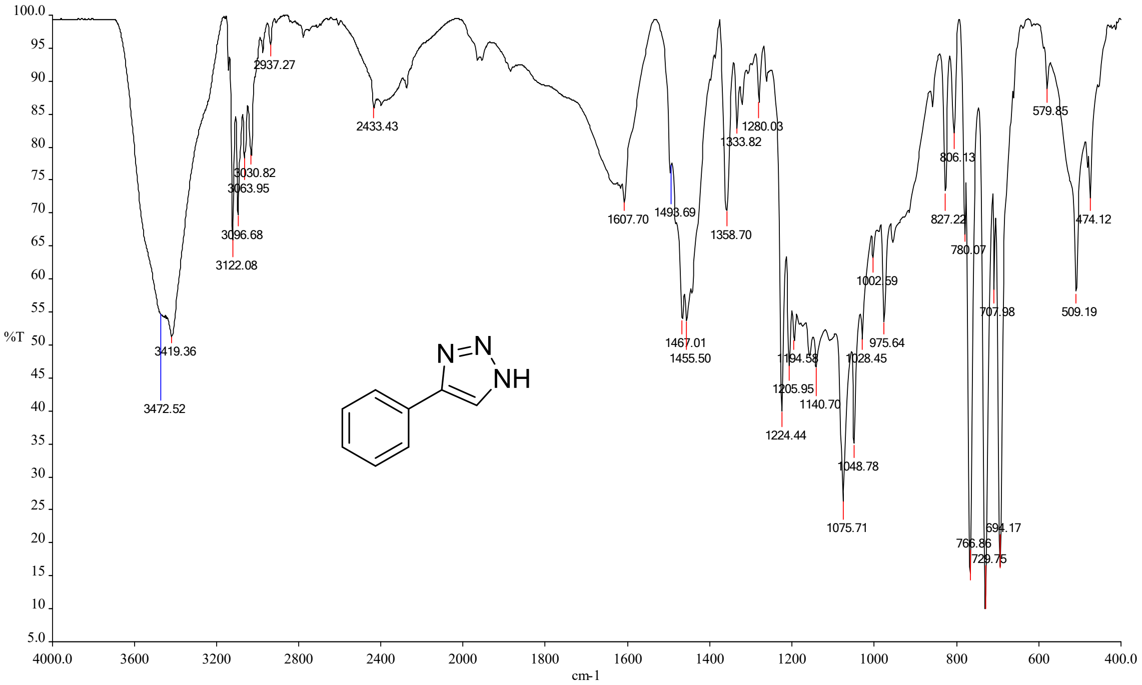

Figure 1. FT-IR spectrum of 4-Phenyl-1*H*-1,2,3-triazole (**1a**) in KBr

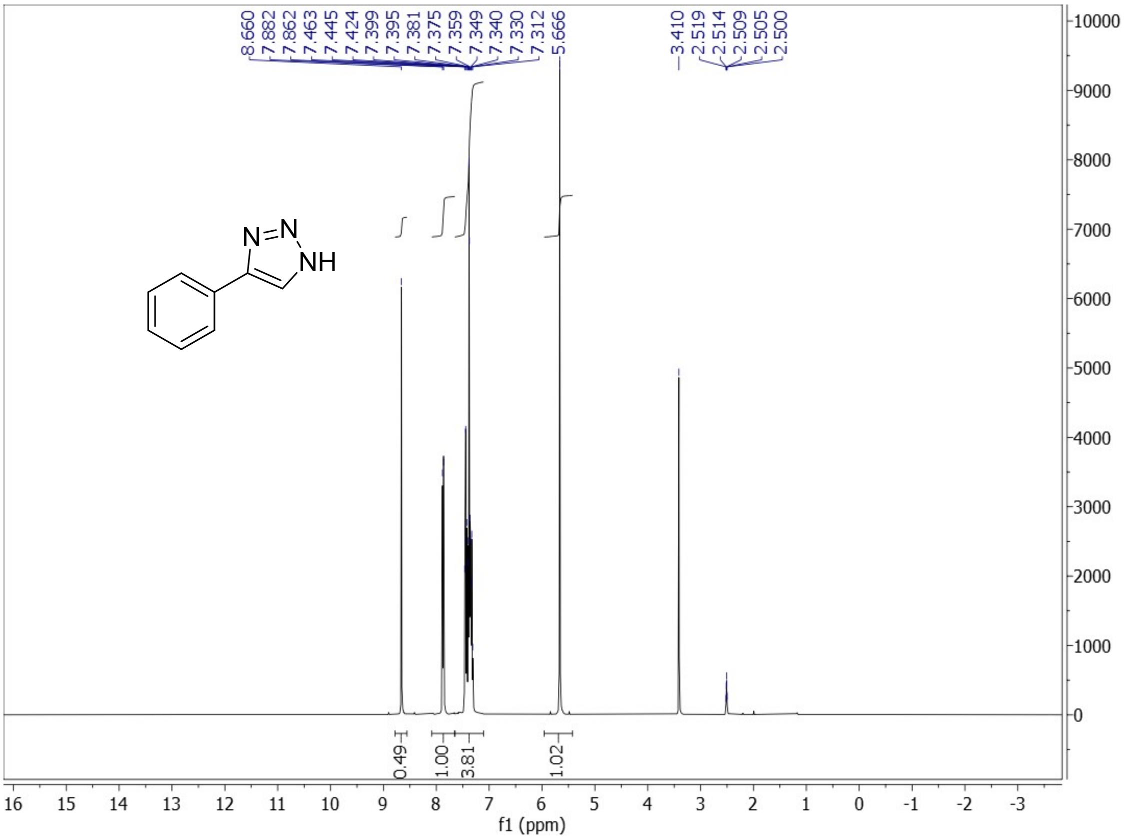

Figure 2. ^{1}H NMR spectrum of 4-Phenyl-1*H*-1,2,3-triazole (**1a**) in DMSO-d_6

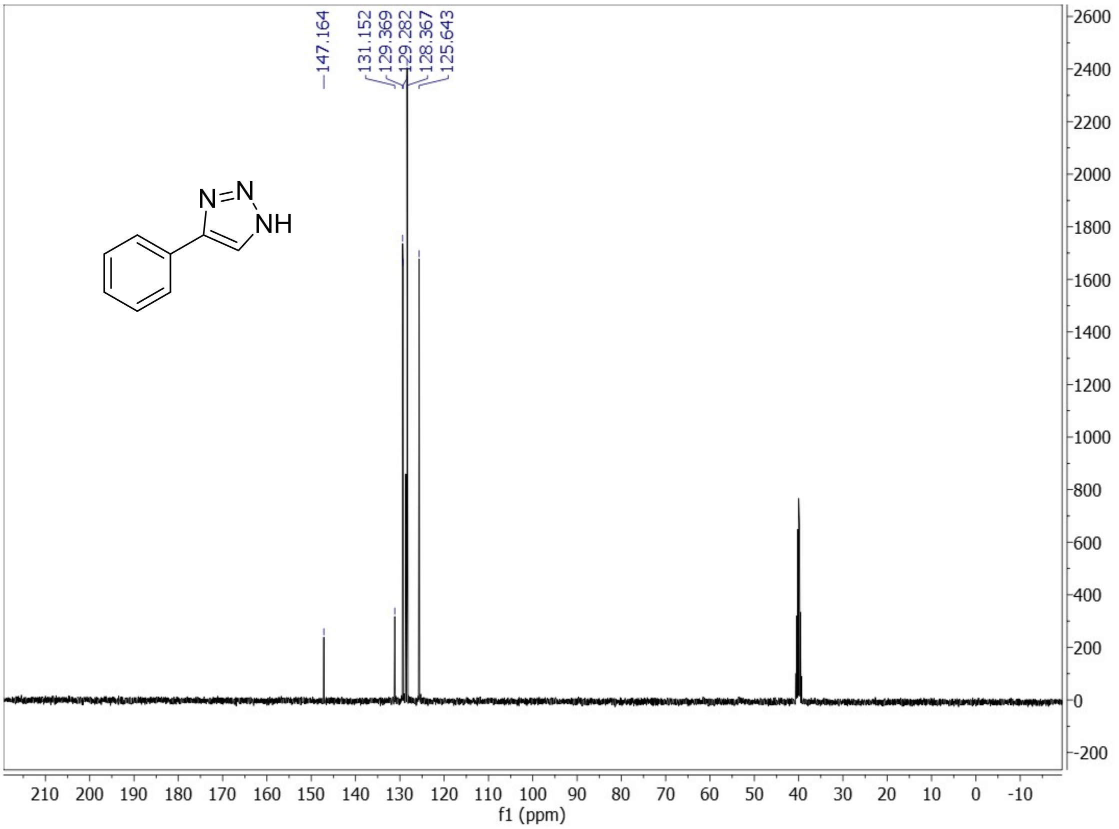

Figure 3. ^{13}C NMR spectrum of 4-Phenyl-1*H*-1,2,3-triazole (**1a**) in DMSO-d_6

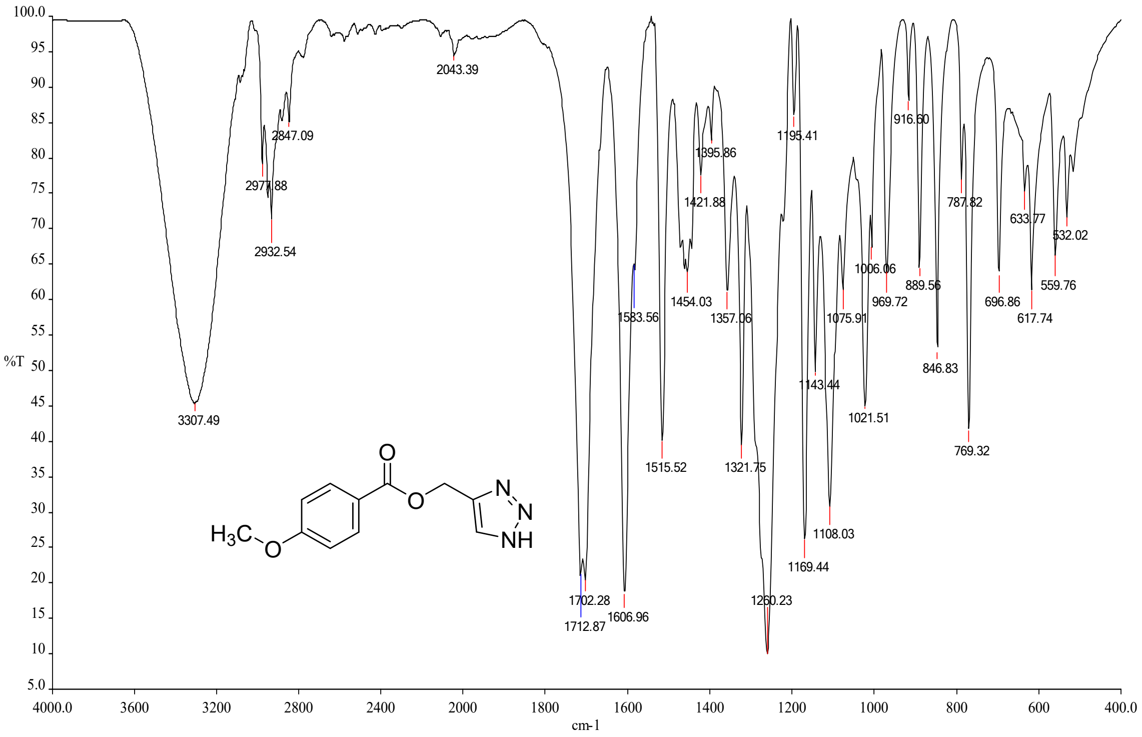

Figure 4. FT-IR spectrum of (1*H*-1,2,3-Triazol-4-yl)methyl 4-methoxybenzoate **(2a)** in KBr

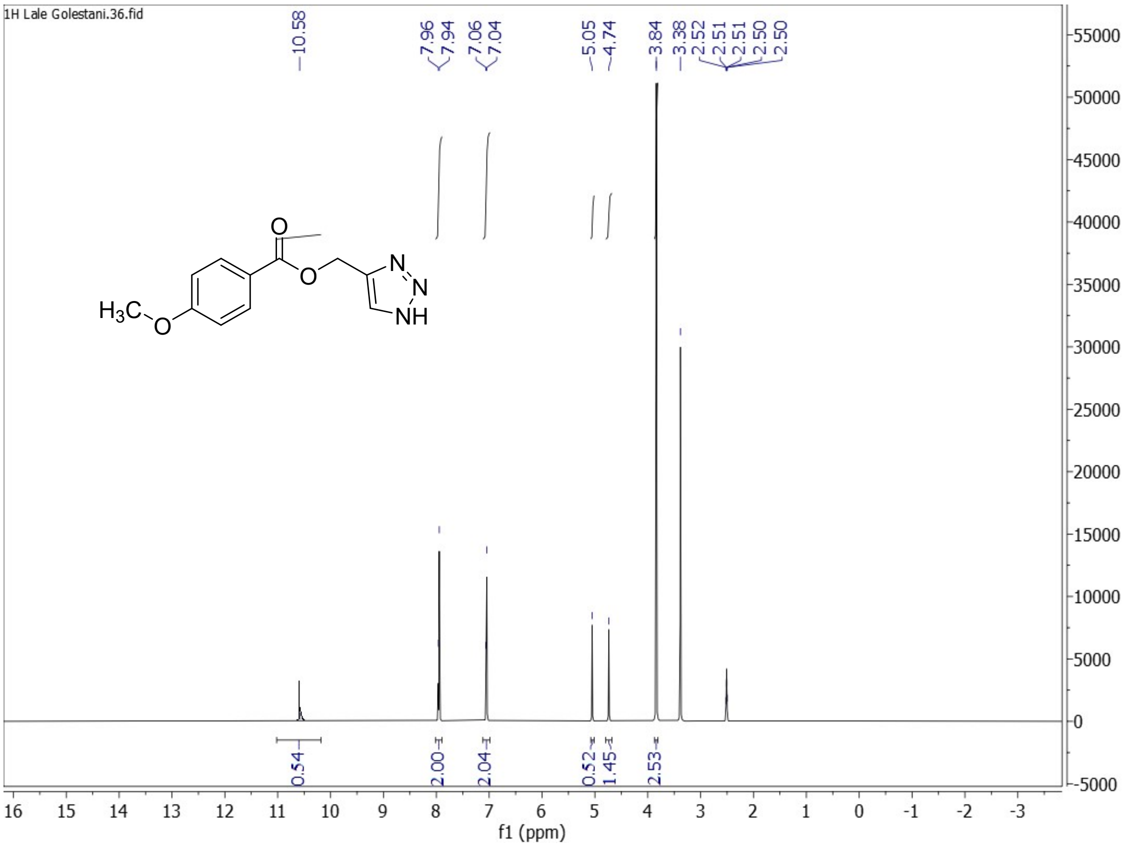

Figure 5. ^{1}H NMR spectrum of (1*H*-1,2,3-Triazol-4-yl)methyl 4-methoxybenzoate (**2a**) in DMSO-d_6

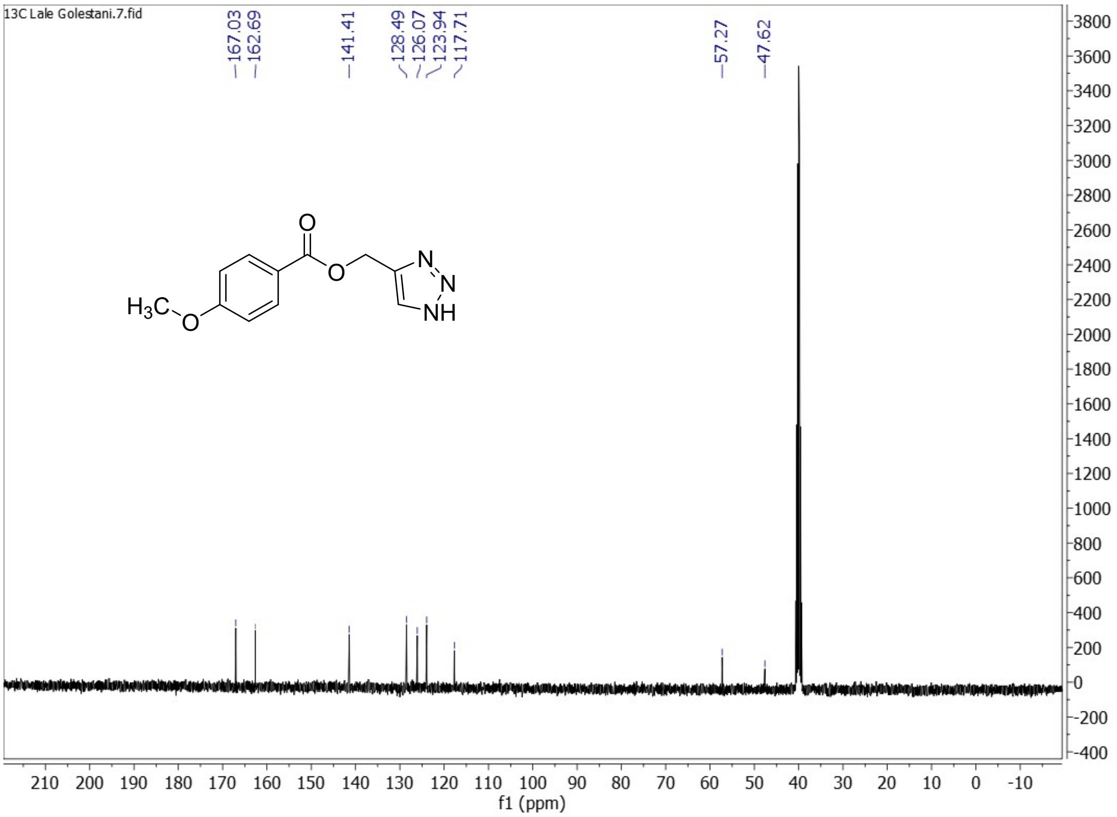

Figure 6. ^{13}C NMR spectrum of (1*H*-1,2,3-Triazol-4-yl)methyl 4-methoxybenzoate (**2a**) in DMSO-d_6

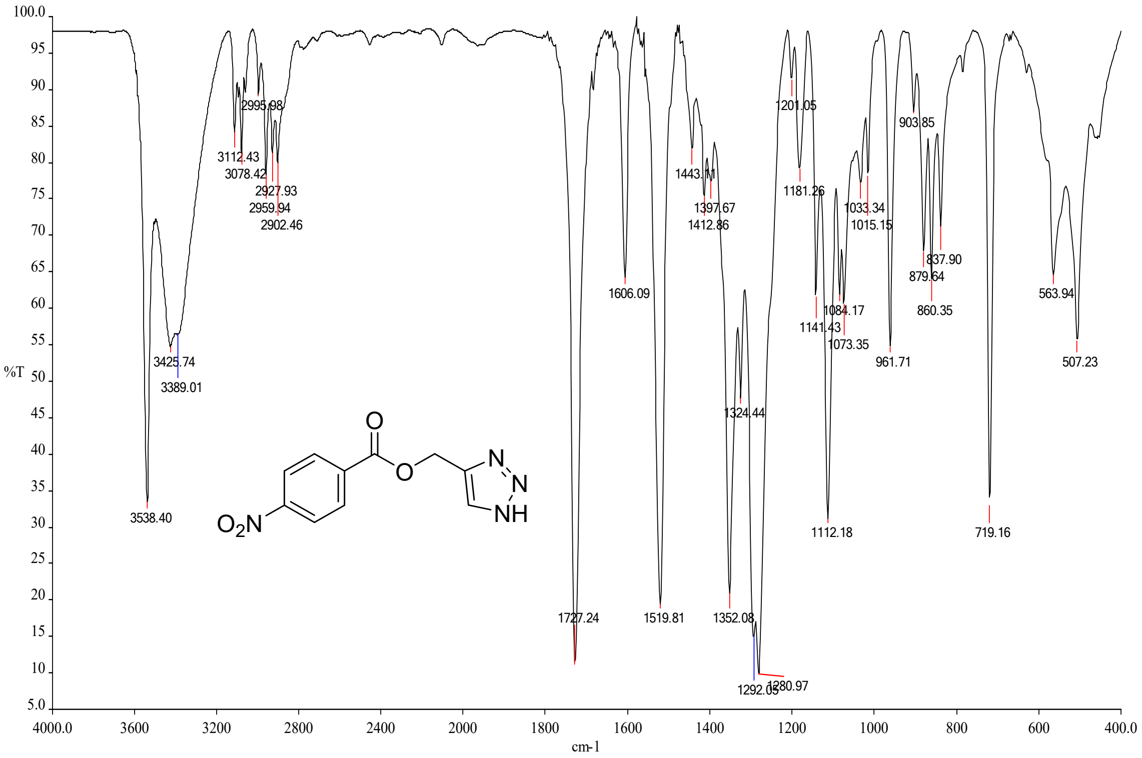

Figure 7. FT-IR spectrum of (1*H*-1,2,3-Triazol-4-yl)methyl 4-nitrobenzoate (**3a**) in KBr

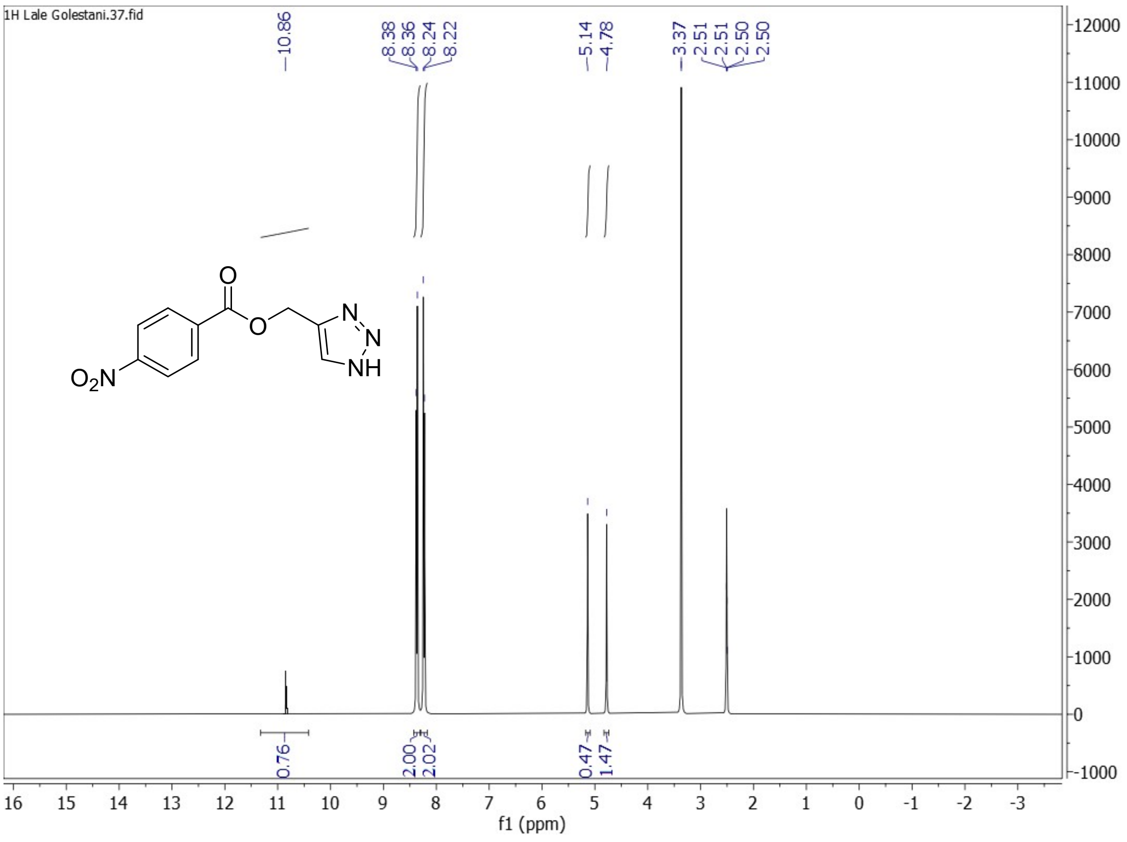

Figure 8. ^{1}H NMR spectrum of (1*H*-1,2,3-Triazol-4-yl)methyl 4-nitrobenzoate (**3a**) in DMSO-d_6

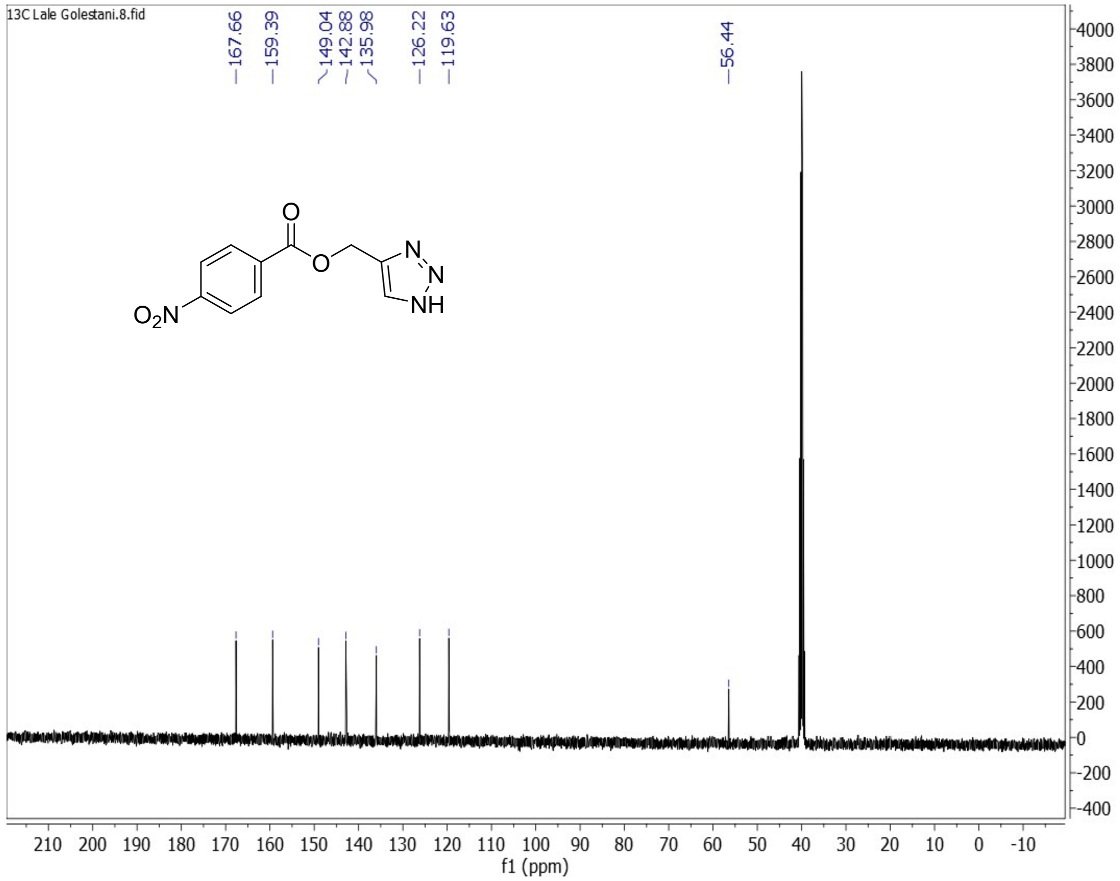

Figure 9. ^{13}C NMR spectrum of (1*H*-1,2,3-Triazol-4-yl)methyl 4-nitrobenzoate (**3a**) in DMSO-d_6

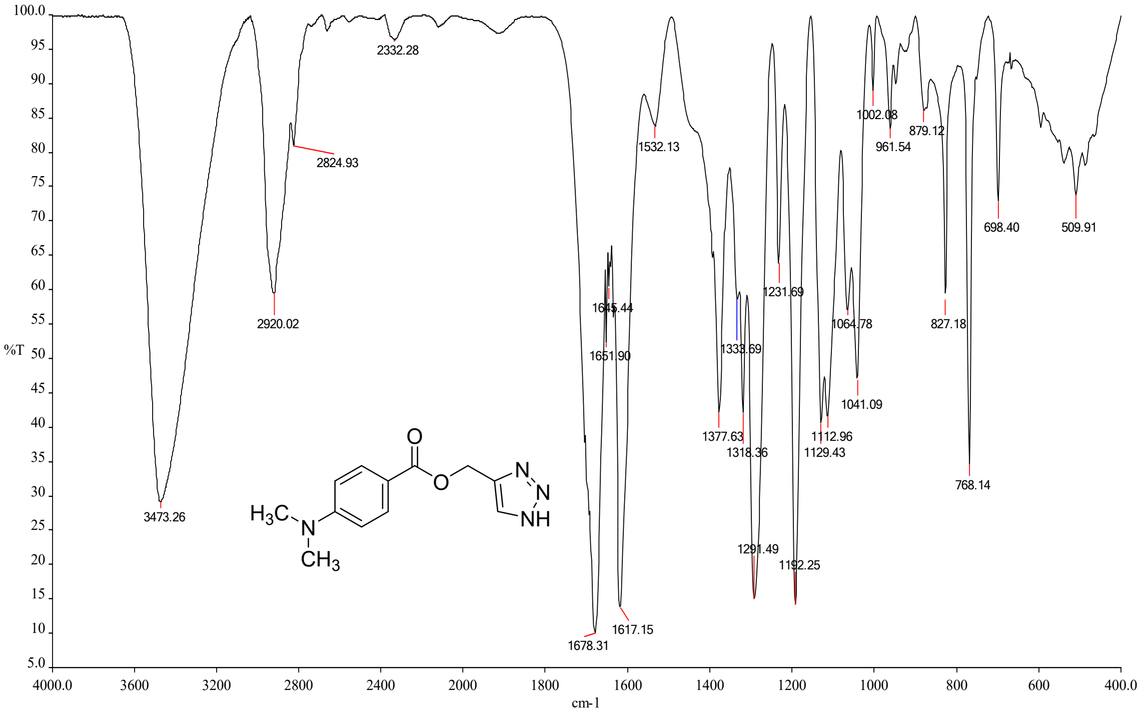

Figure 10. FT-IR spectrum of (1*H*-1,2,3-Triazol-4-yl)methyl 4-(dimethylamino)benzoate (**4a**) in KBr

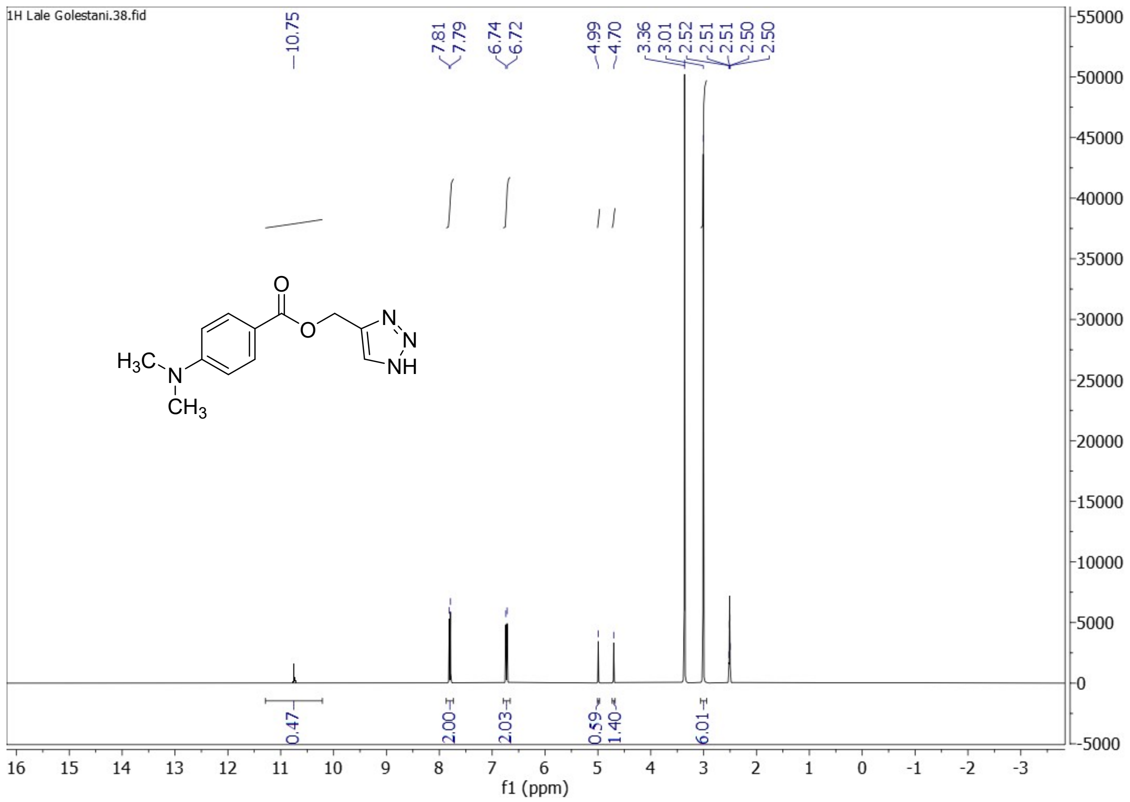

Figure 11. ^{1}H NMR spectrum of (1*H*-1,2,3-Triazol-4-yl)methyl 4-(dimethylamino)benzoate (**4a**) in DMSO-d_6

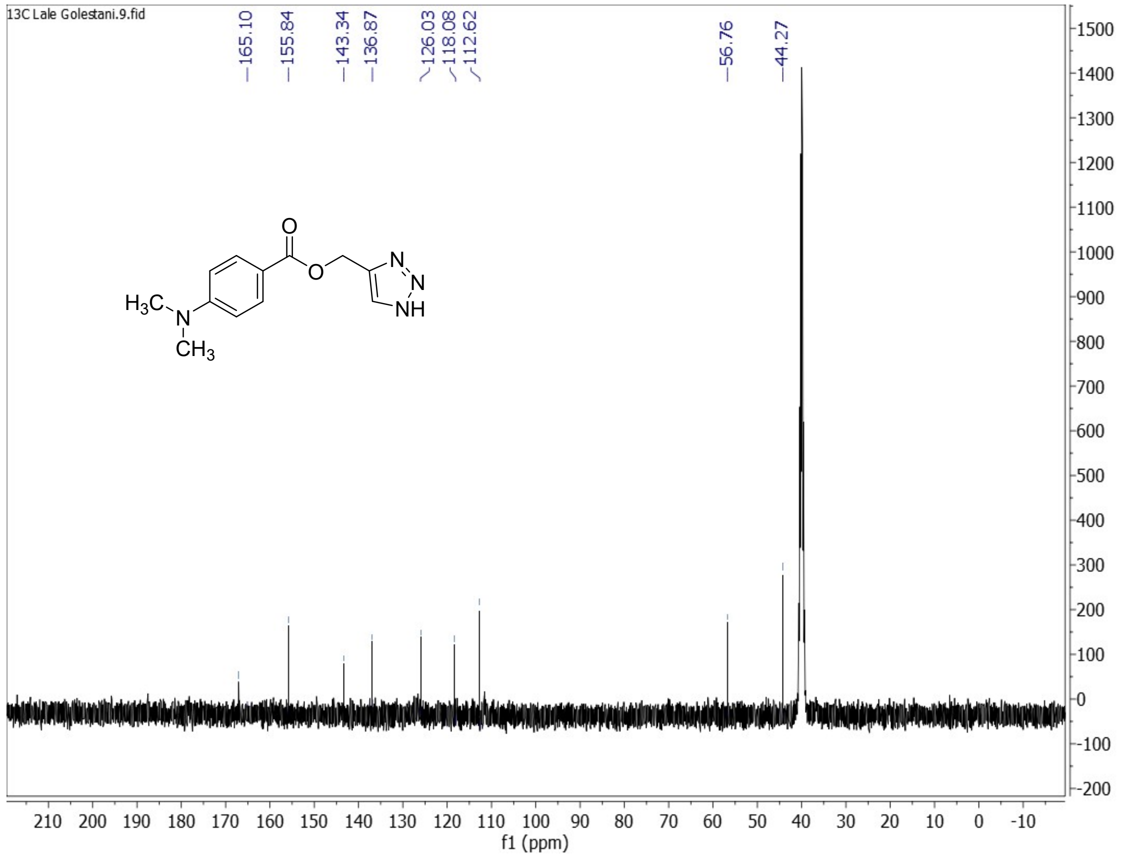

Figure 12. ^{13}C NMR spectrum of (1*H*-1,2,3-Triazol-4-yl)methyl 4-(dimethylamino)benzoate (**4a**) in DMSO-d_6

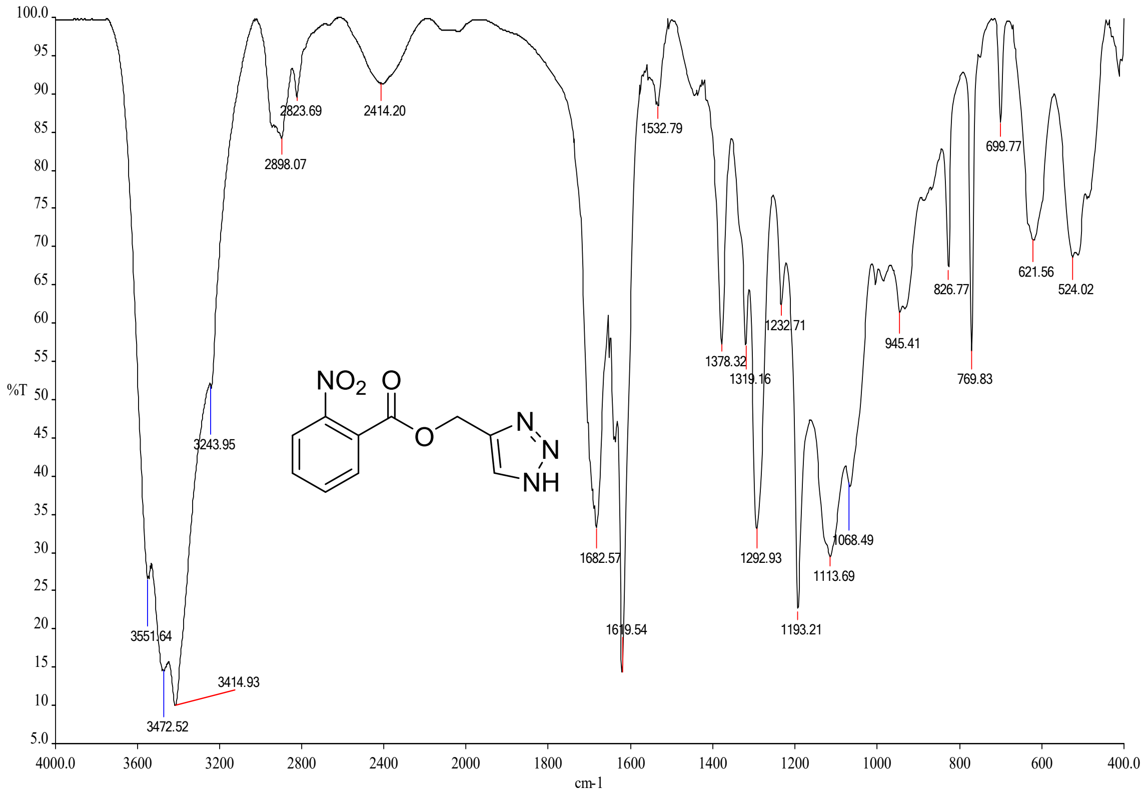

Figure 13. FT-IR spectrum of (1*H*-1,2,3-triazol-4-yl)methyl 2-nitrobenzoate (**5a**) in KBr

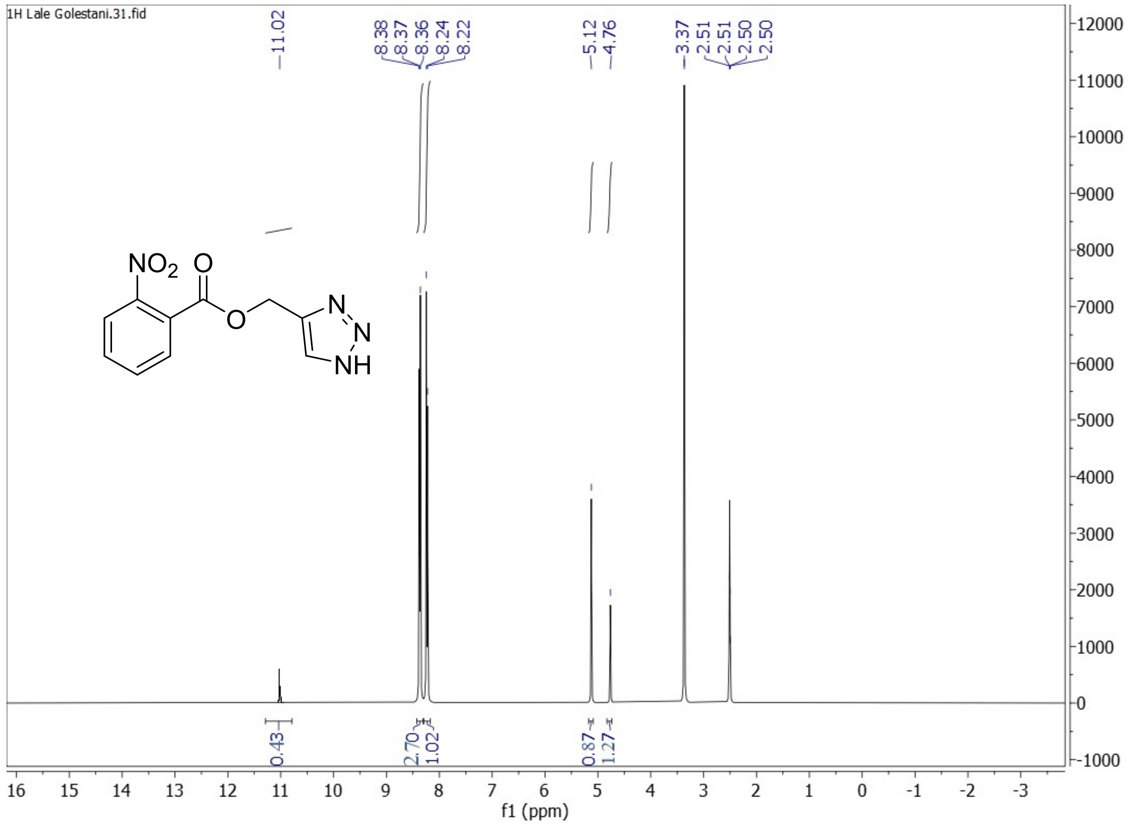

Figure 14. ^{1}H NMR spectrum of (1*H*-1,2,3-triazol-4-yl)methyl 2-nitrobenzoate (**5a**) in DMSO-d_6

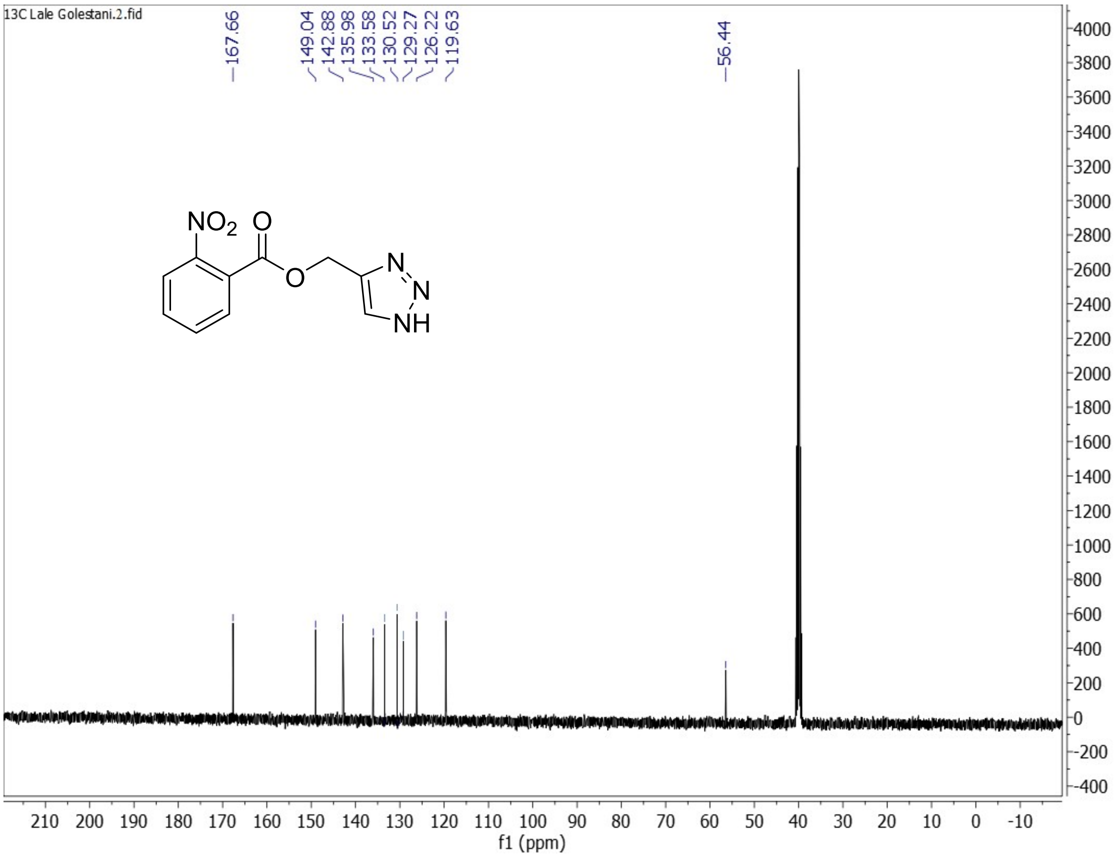

Figure 15. ^{13}C NMR spectrum of (1*H*-1,2,3-triazol-4-yl)methyl 2-nitrobenzoate (**5a**) in DMSO-d_6

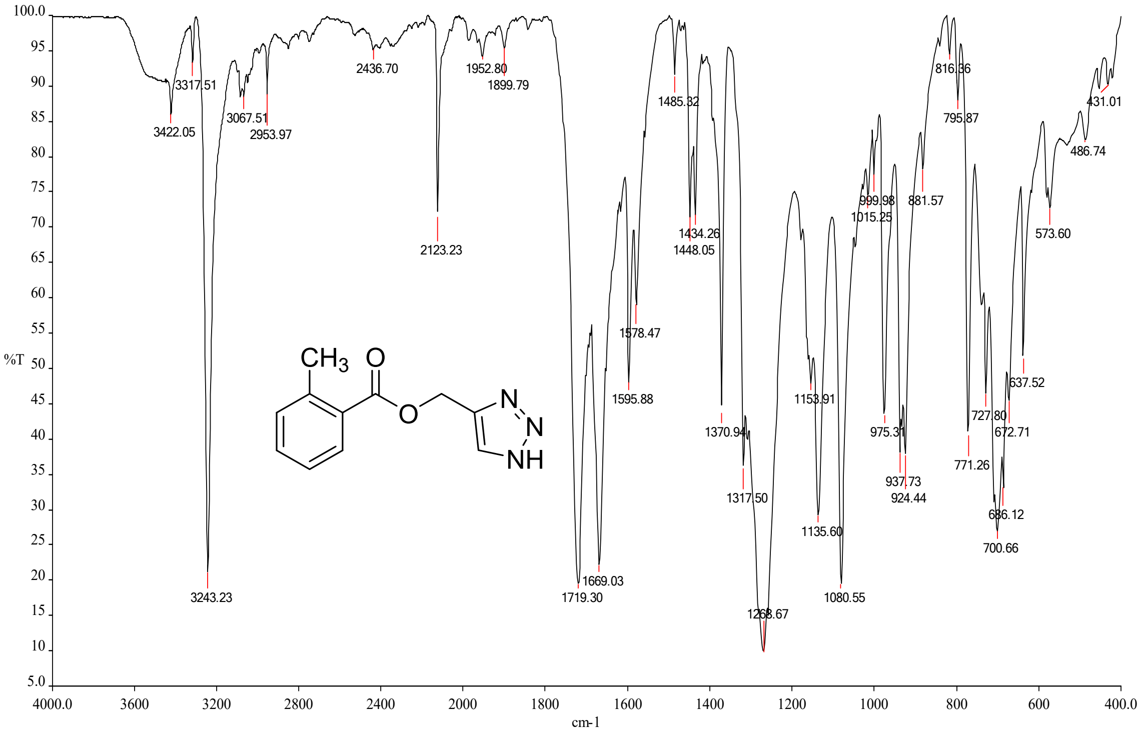

Figure 16. FT-IR spectrum of (1*H*-1,2,3-triazol-4-yl)methyl 2-methylbenzoate (**6a**) in KBr

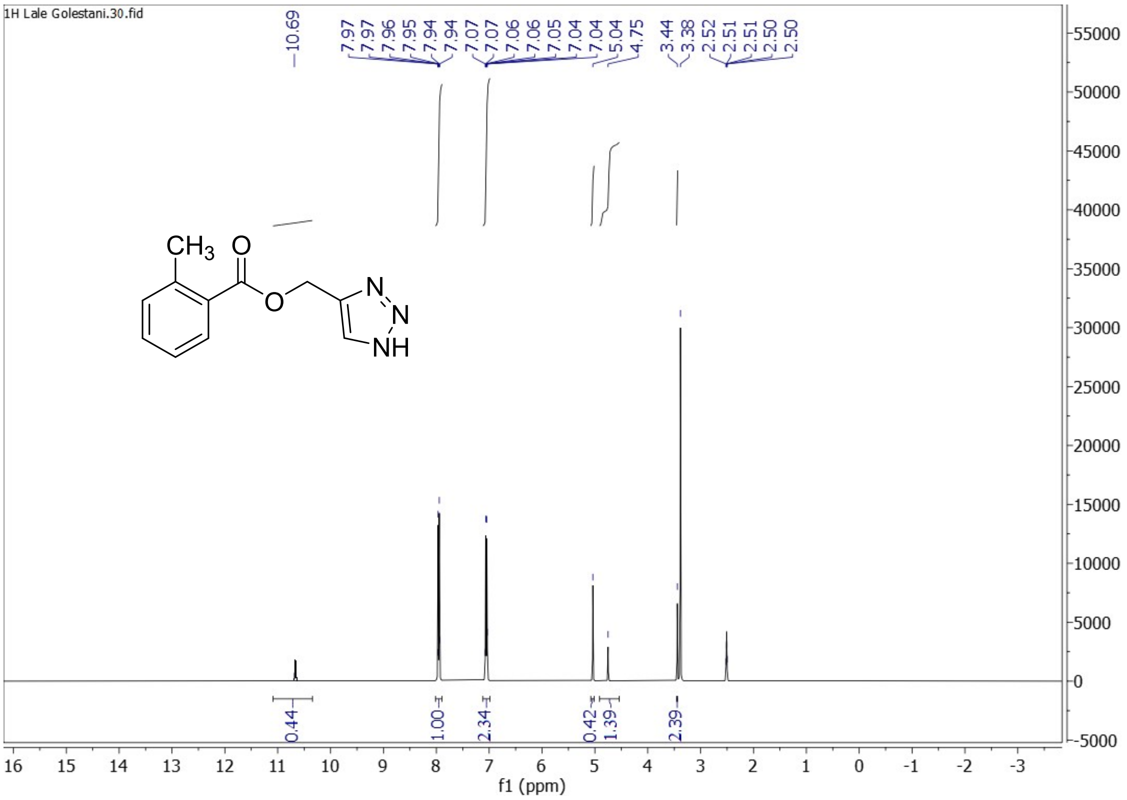

Figure 17. ^{1}H NMR spectrum of (1*H*-1,2,3-triazol-4-yl)methyl 2-methylbenzoate (**6a**) in DMSO-d_6

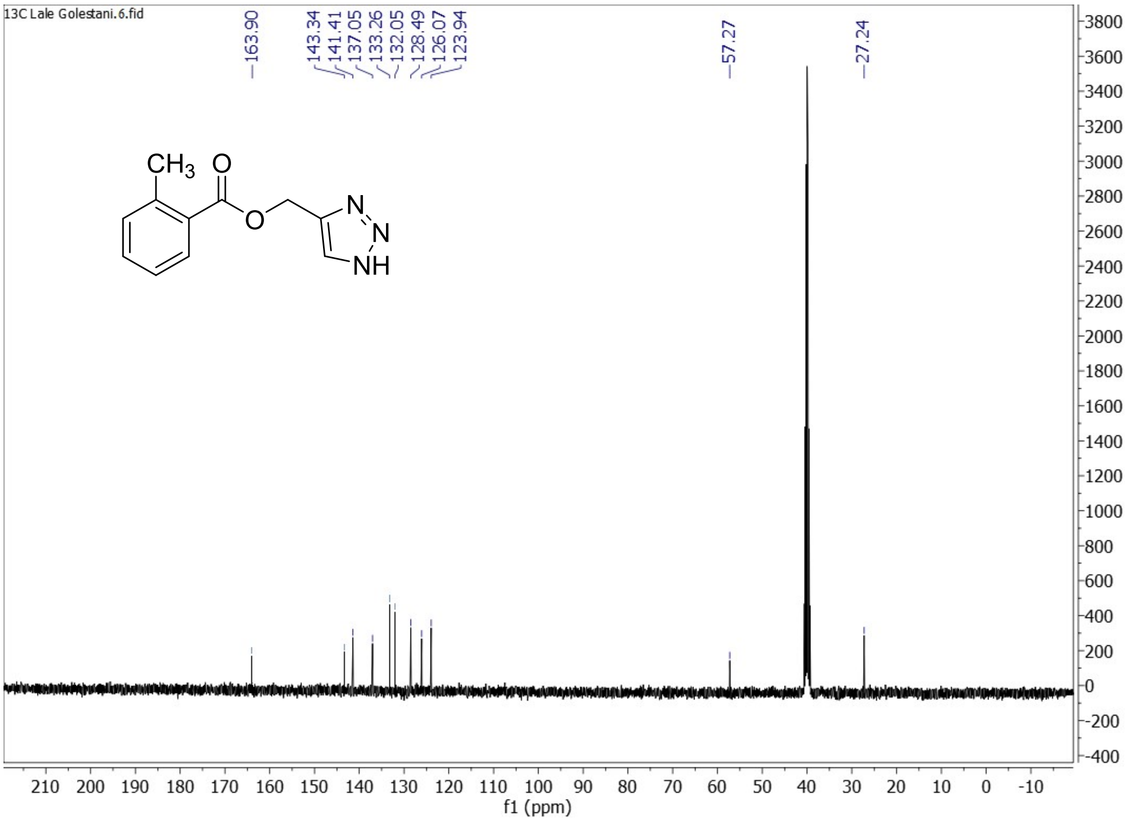

Figure 18. ^{13}C NMR spectrum of (1*H*-1,2,3-triazol-4-yl)methyl 2-methylbenzoate (**6a**) in DMSO-d_6

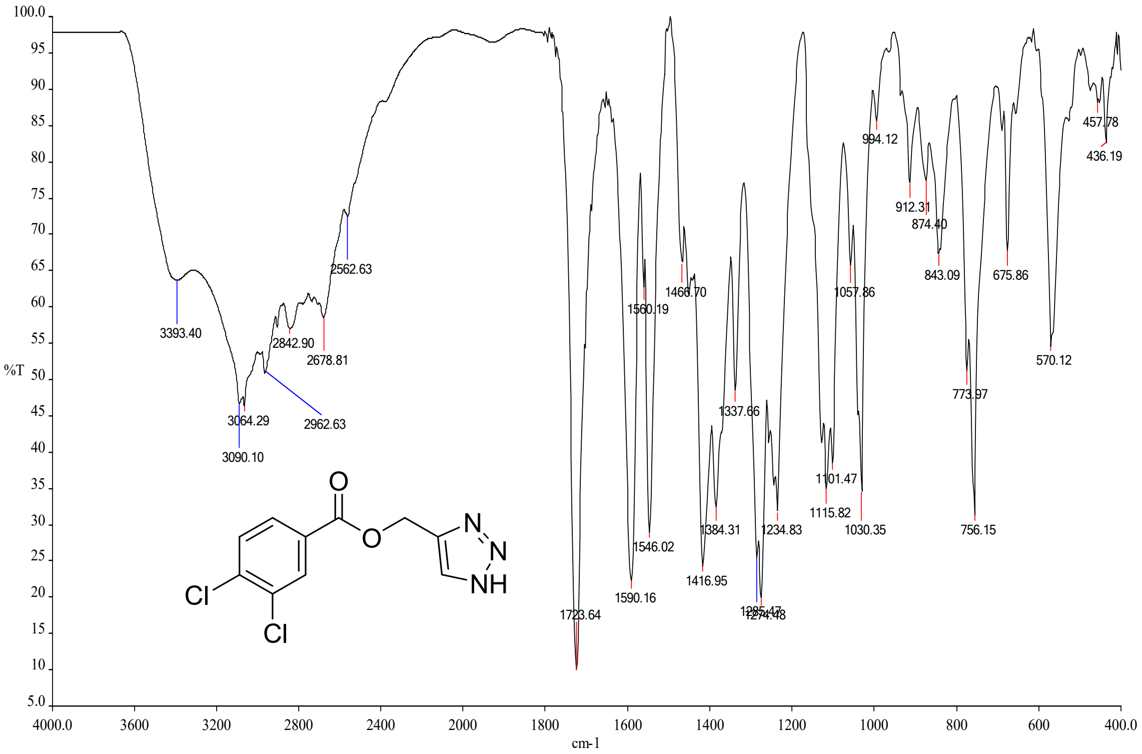

Figure 19. FT-IR spectrum of (1*H*-1,2,3-triazol-4-yl)methyl 3,4-dichlorobenzoate (**7a**) in KBr

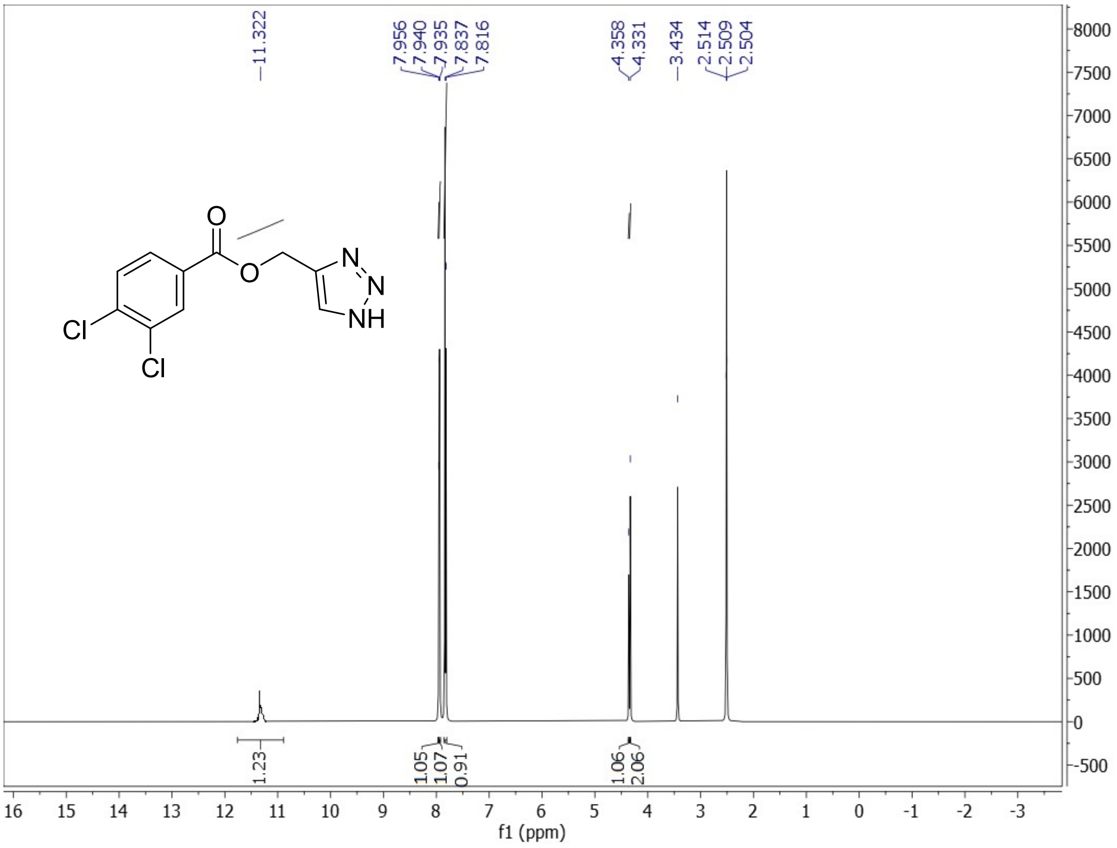

Figure 20. ^{1}H NMR spectrum of (1*H*-1,2,3-triazol-4-yl)methyl 3,4-dichlorobenzoate (**7a**) in DMSO-d_6

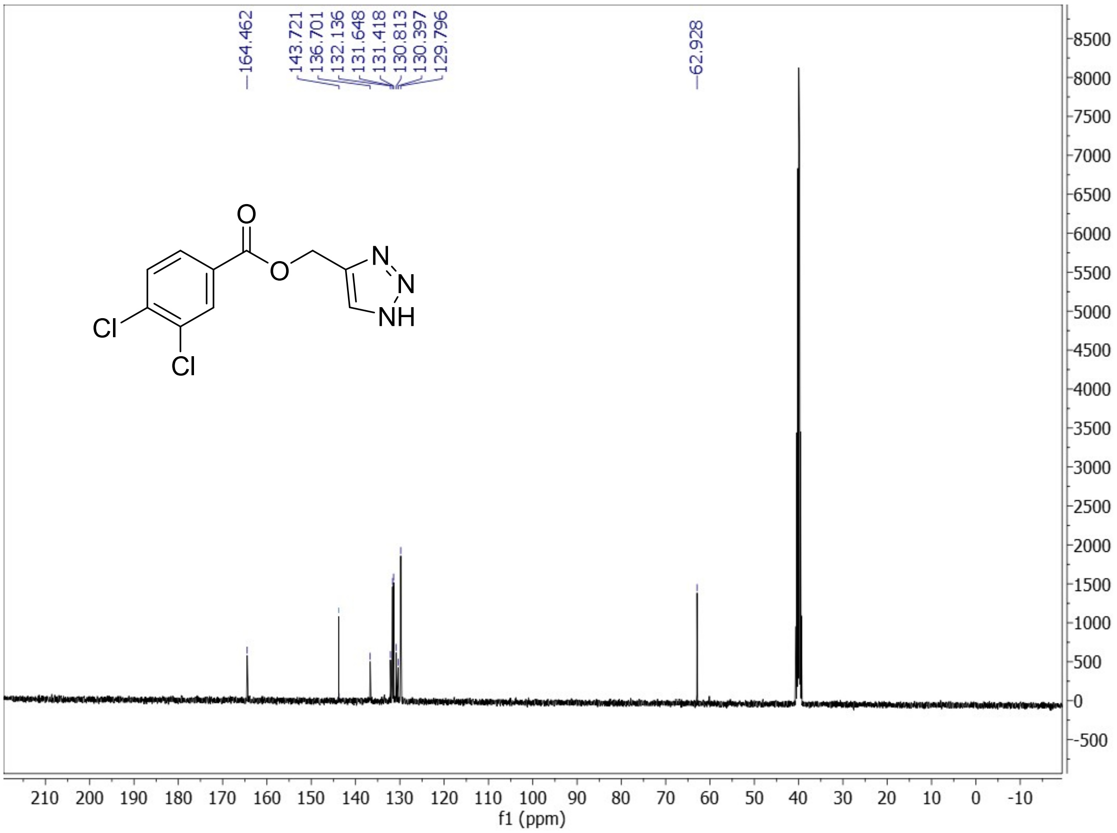

Figure 21. ^{13}C NMR spectrum of (1*H*-1,2,3-triazol-4-yl)methyl 3,4-dichlorobenzoate (**7a**) in DMSO-d_6

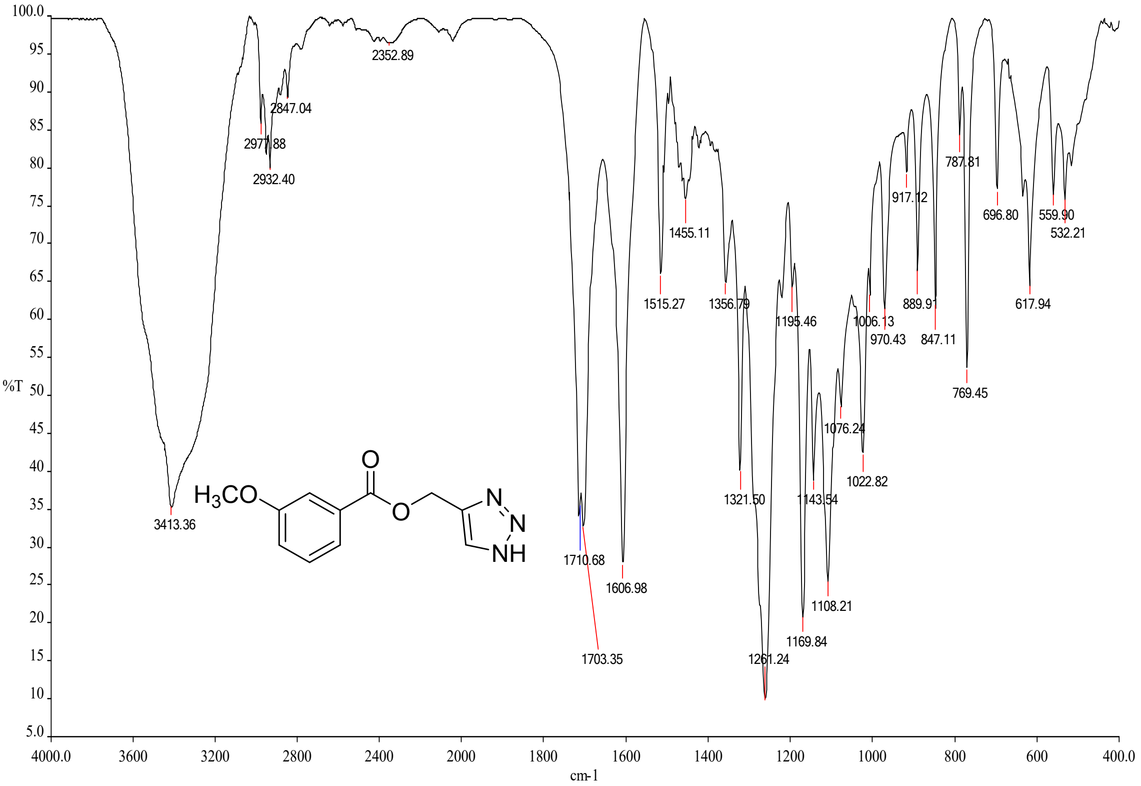

Figure 22. FT-IR spectrum of (1*H*-1,2,3-triazol-4-yl)methyl 3-methoxybenzoate (**8a**) in KBr

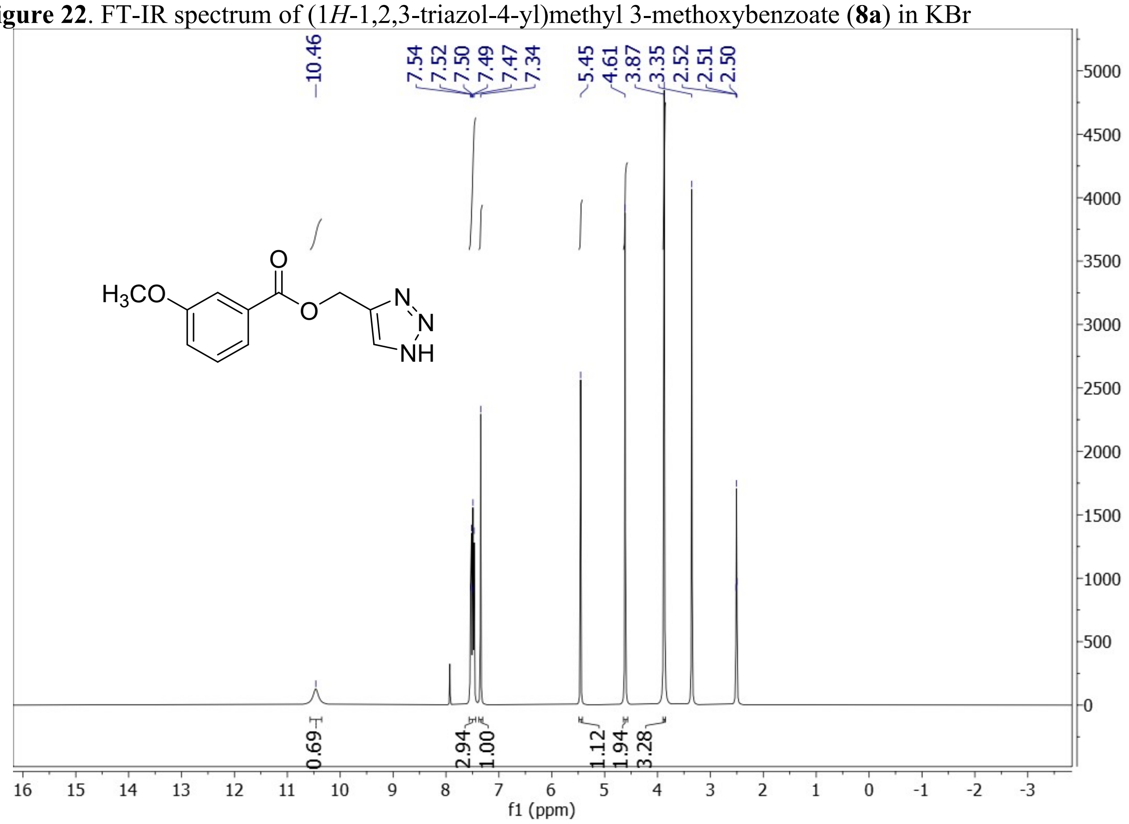

Figure 23. ^{1}H NMR spectrum of (1*H*-1,2,3-triazol-4-yl)methyl 3-methoxybenzoate (**8a**) in DMSO-d_6

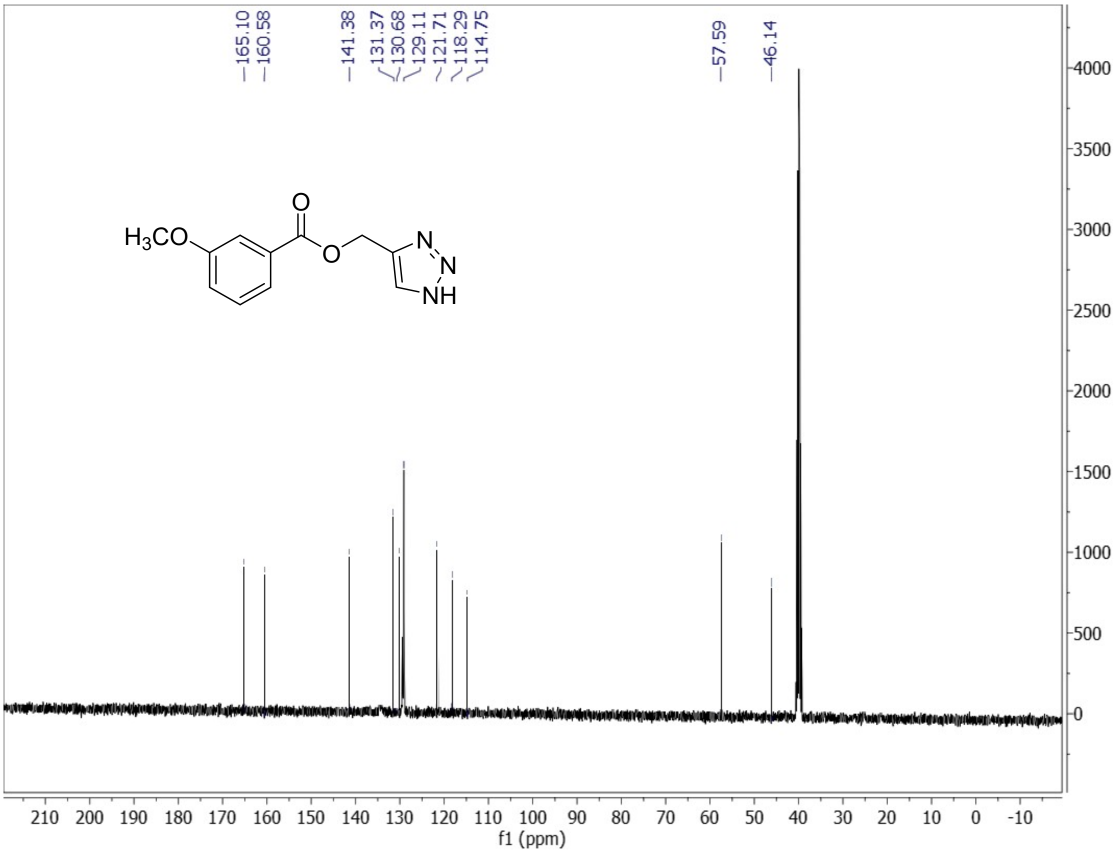

Figure 24. ^{13}C NMR spectrum of (1*H*-1,2,3-triazol-4-yl)methyl 3-methoxybenzoate (**8a**) in DMSO-d_6

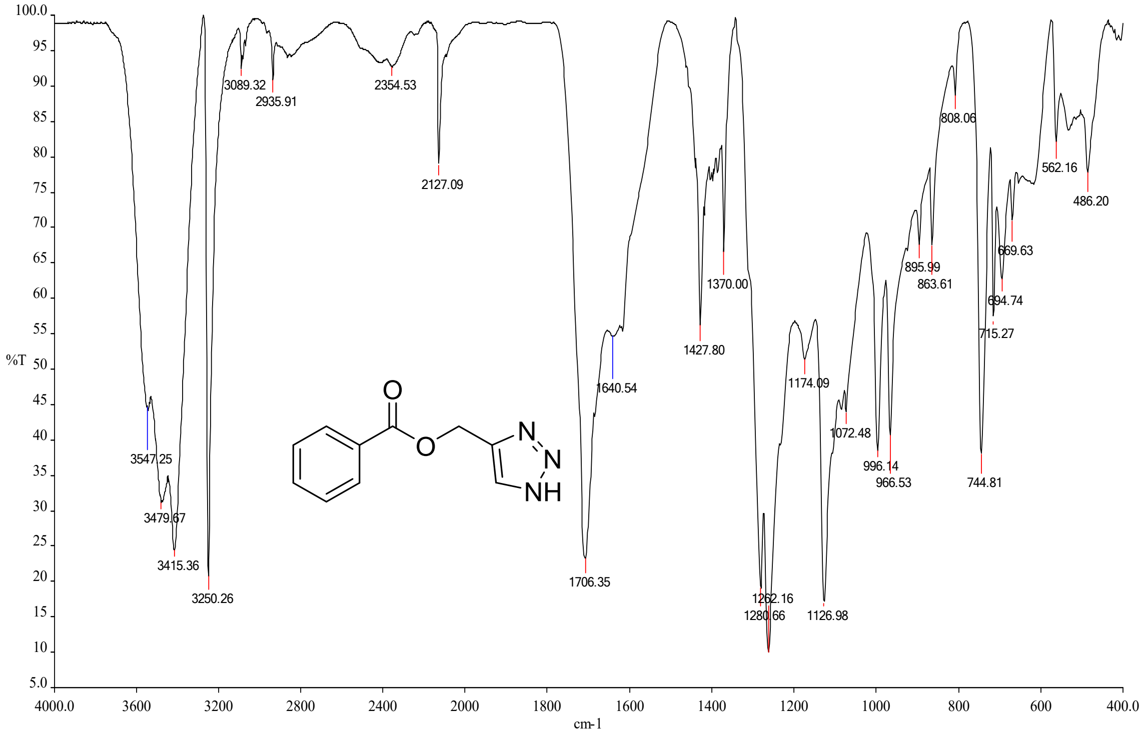

Figure 25. FT-IR spectrum of (1*H*-1,2,3-triazol-4-yl)methyl benzoate (**9a**) in KBr

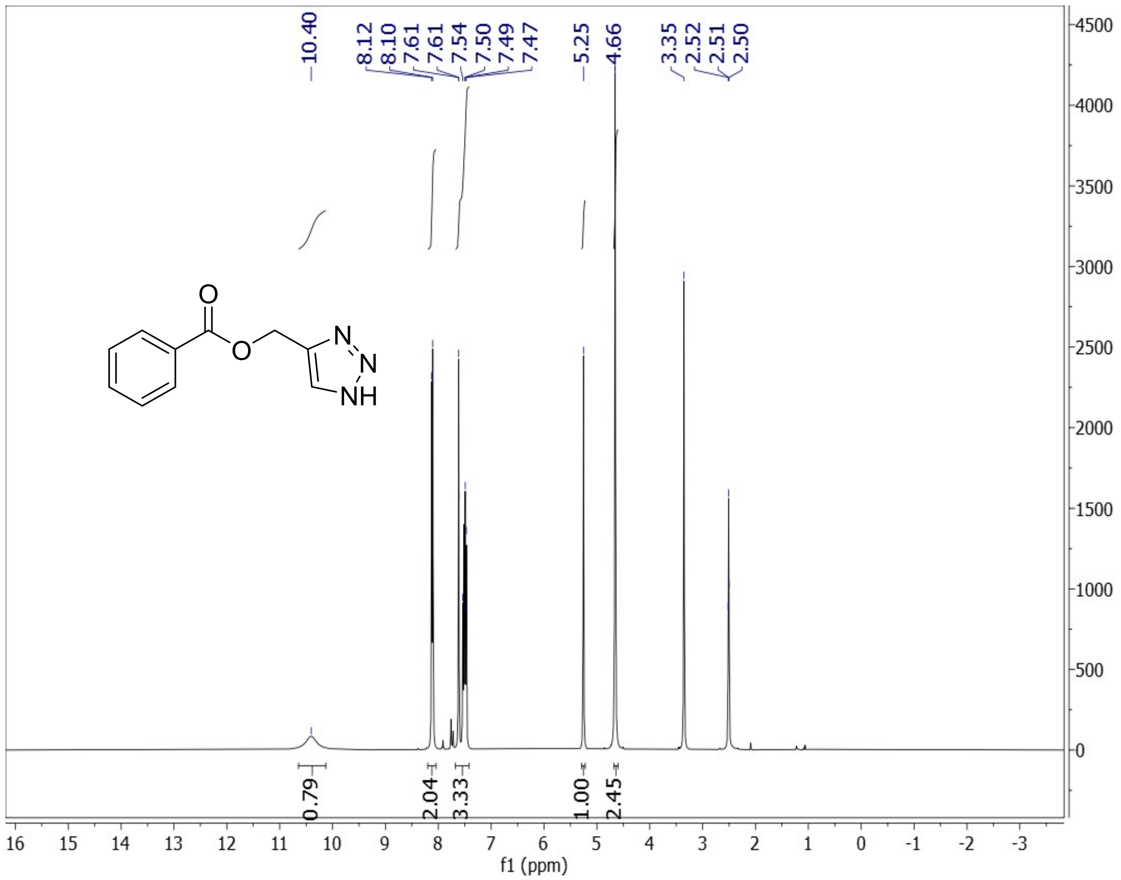

Figure 26. ^{1}H NMR spectrum of (1*H*-1,2,3-triazol-4-yl)methyl benzoate (**9a**) in DMSO-d_6

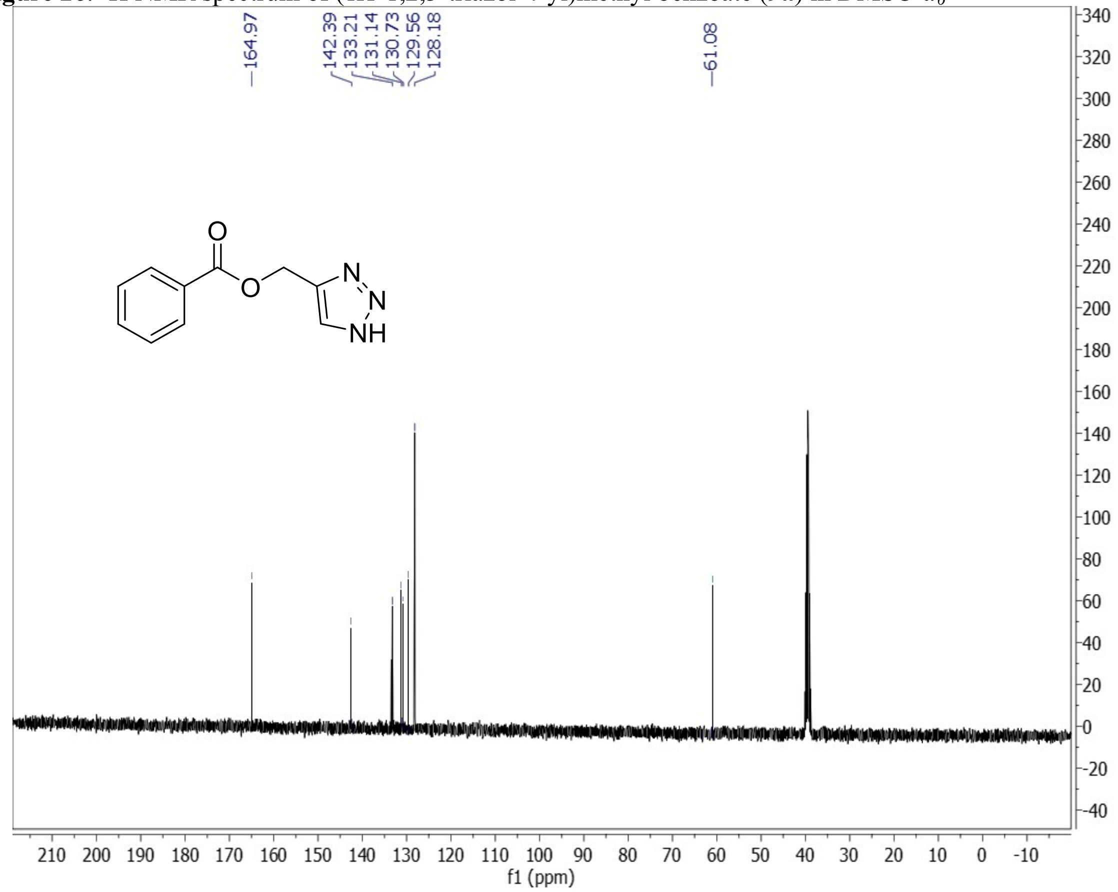

Figure 27. ^{13}C NMR spectrum of (1*H*-1,2,3-triazol-4-yl)methyl benzoate (**9a**) in DMSO-d_6

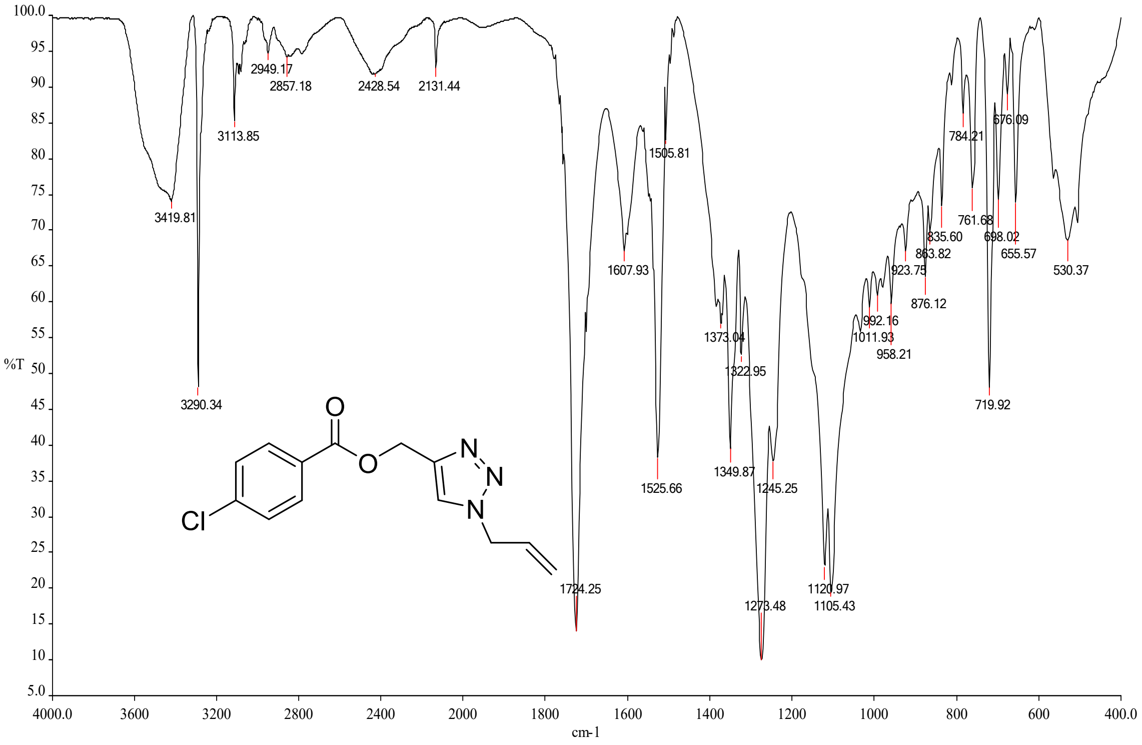

Figure 28. FT-IR spectrum of (1-allyl-1H-1,2,3-triazol-4-yl)methyl 4-chlorobenzoate (**10a**) in KBr

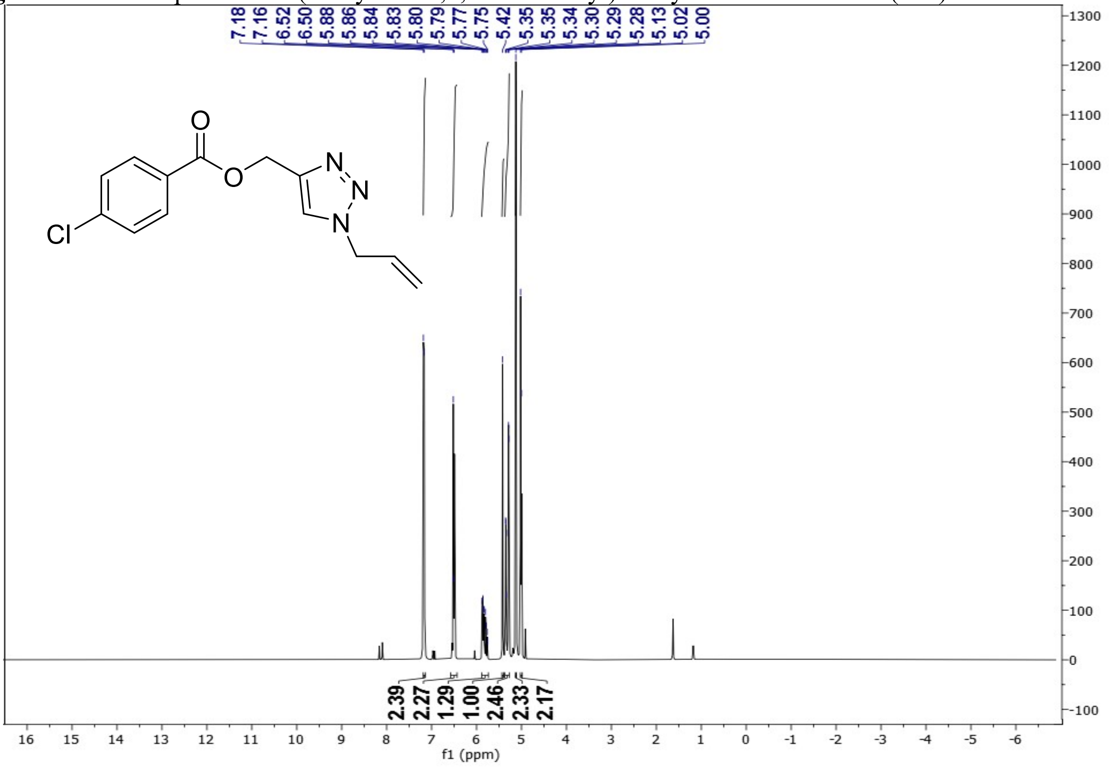

Figure 29. ^{1}H NMR spectrum of (1-allyl-1H-1,2,3-triazol-4-yl)methyl 4-chlorobenzoate (**10a**) in $CDCl_3$

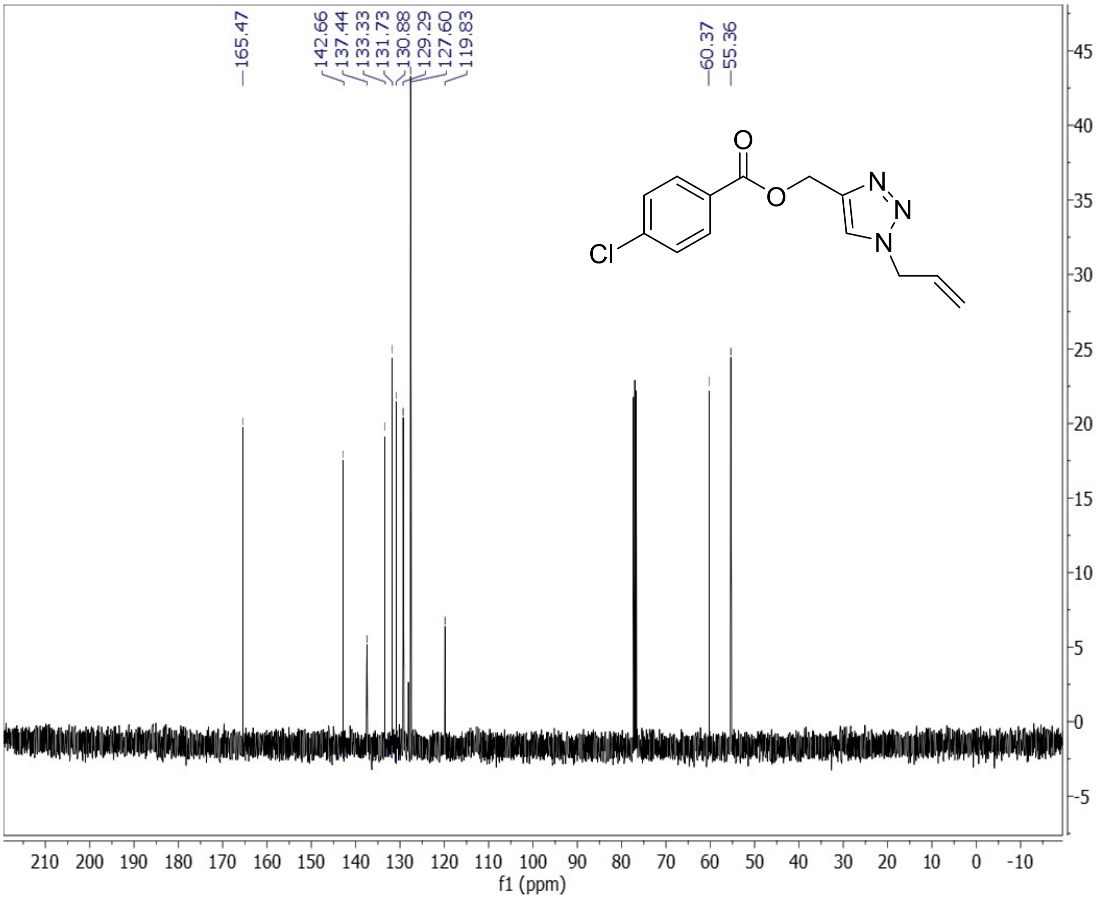

Figure 30. ^{13}C NMR spectrum of (1-allyl-1*H*-1,2,3-triazol-4-yl)methyl 4-chlorobenzoate (**10a**) in $CDCl_3$

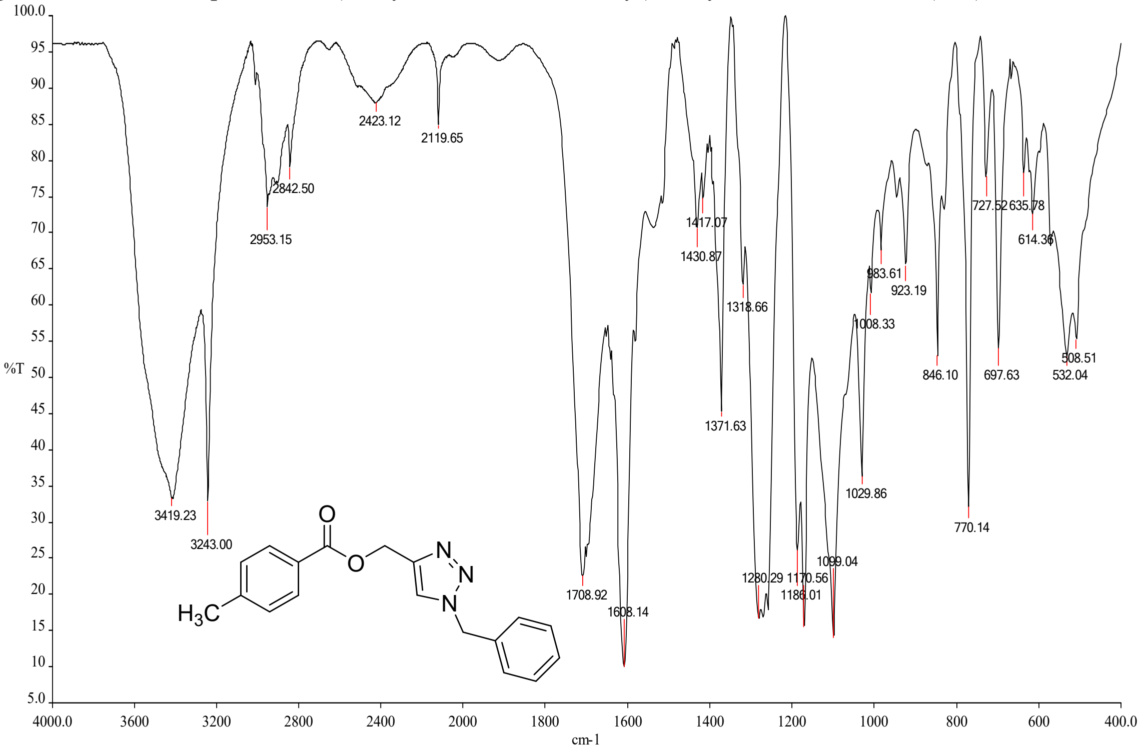

Figure 31. FT-IR spectrum of (1-benzyl-1*H*-1,2,3-triazol-4-yl)methyl 4-methylbenzoate (**11a**) in KBr

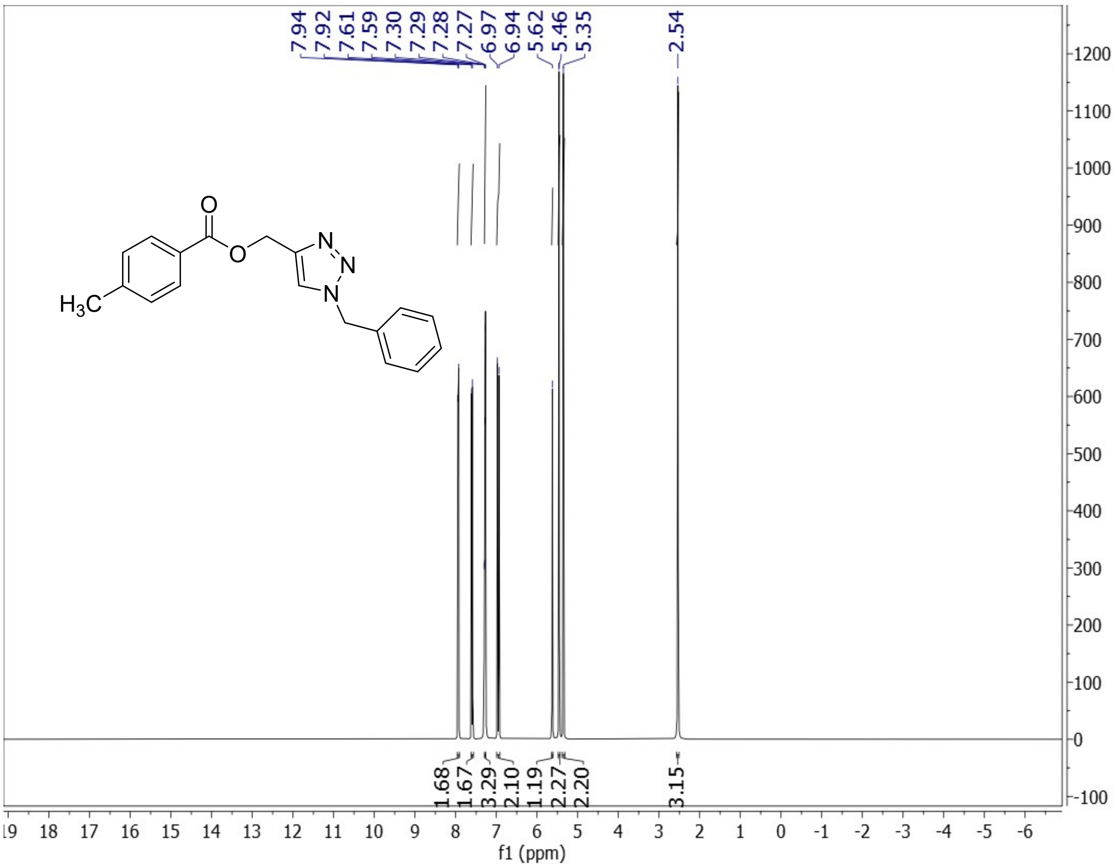

Figure 32. ^{1}H NMR spectrum of (1-benzyl-1*H*-1,2,3-triazol-4-yl)methyl 4-methylbenzoate (**11a**) in $CDCl_3$

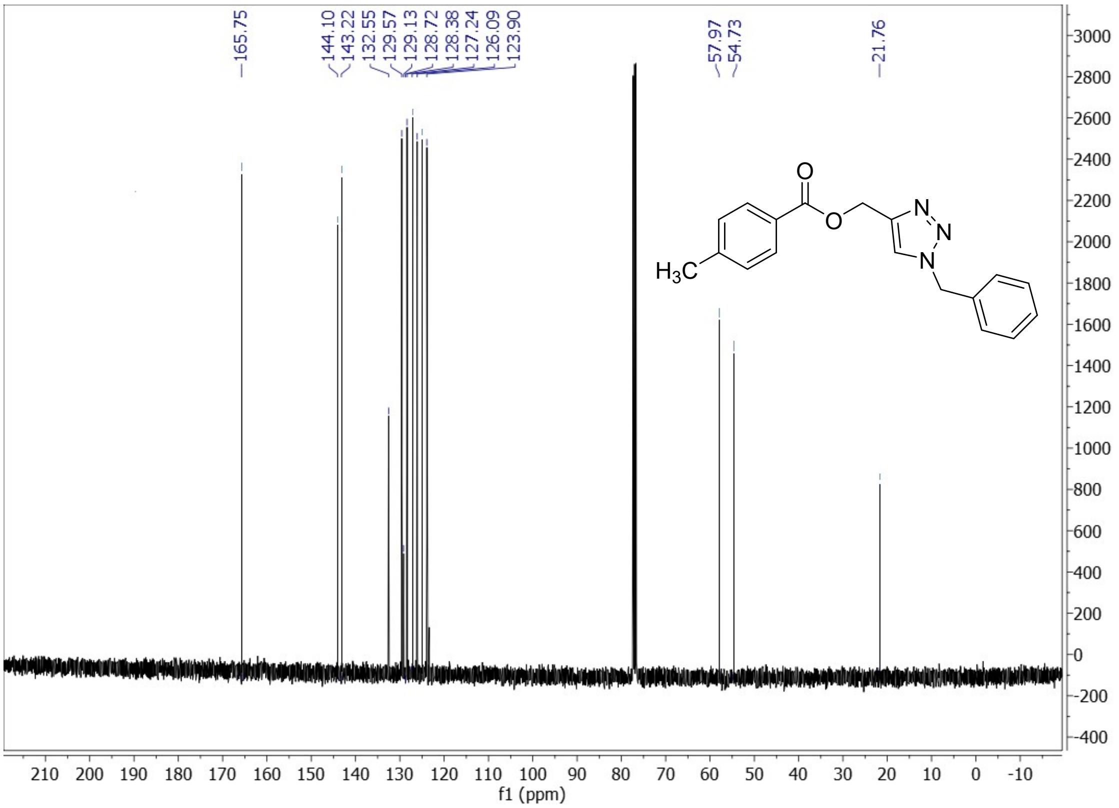

Figure 33. ^{13}C NMR spectrum of (1-benzyl-1*H*-1,2,3-triazol-4-yl)methyl 4-methylbenzoate (**11a**) in $CDCl_3$

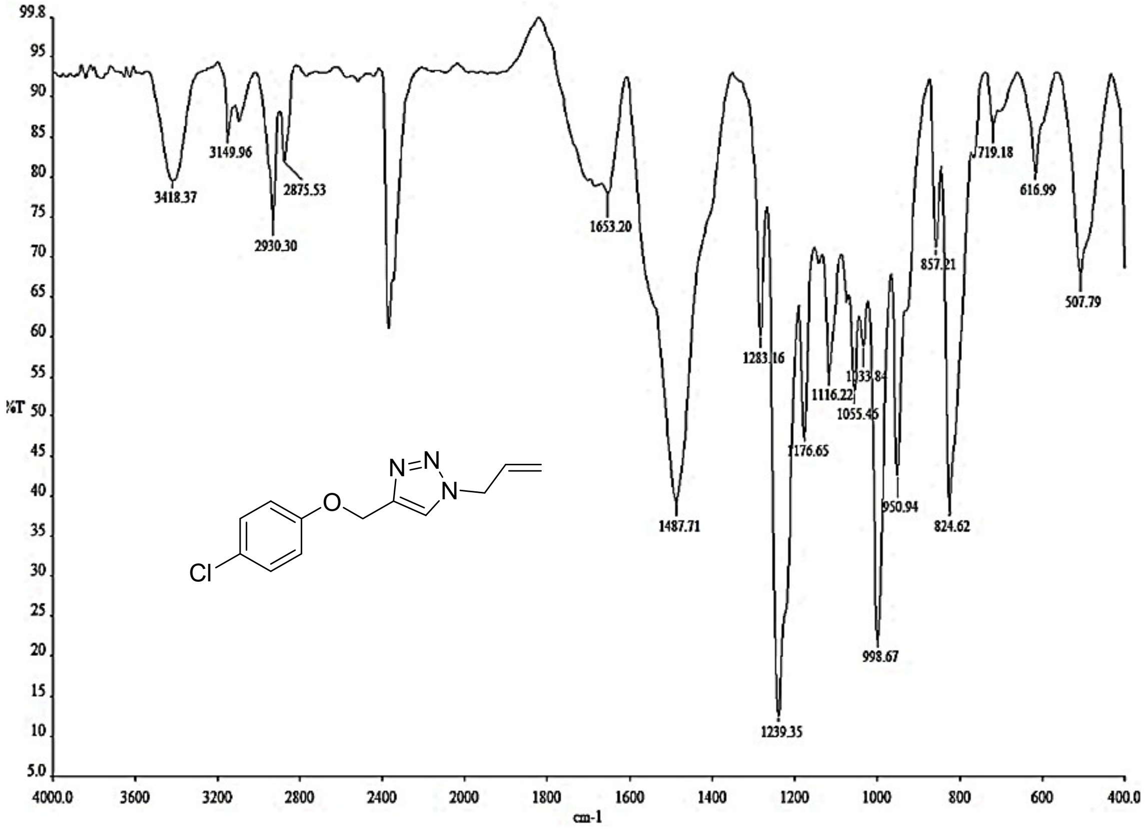

Figure 34. FT-IR spectrum of 1-allyl-4-((4-chlorophenoxy)methyl)-1H-1,2,3-triazole (**12a**) in KBr

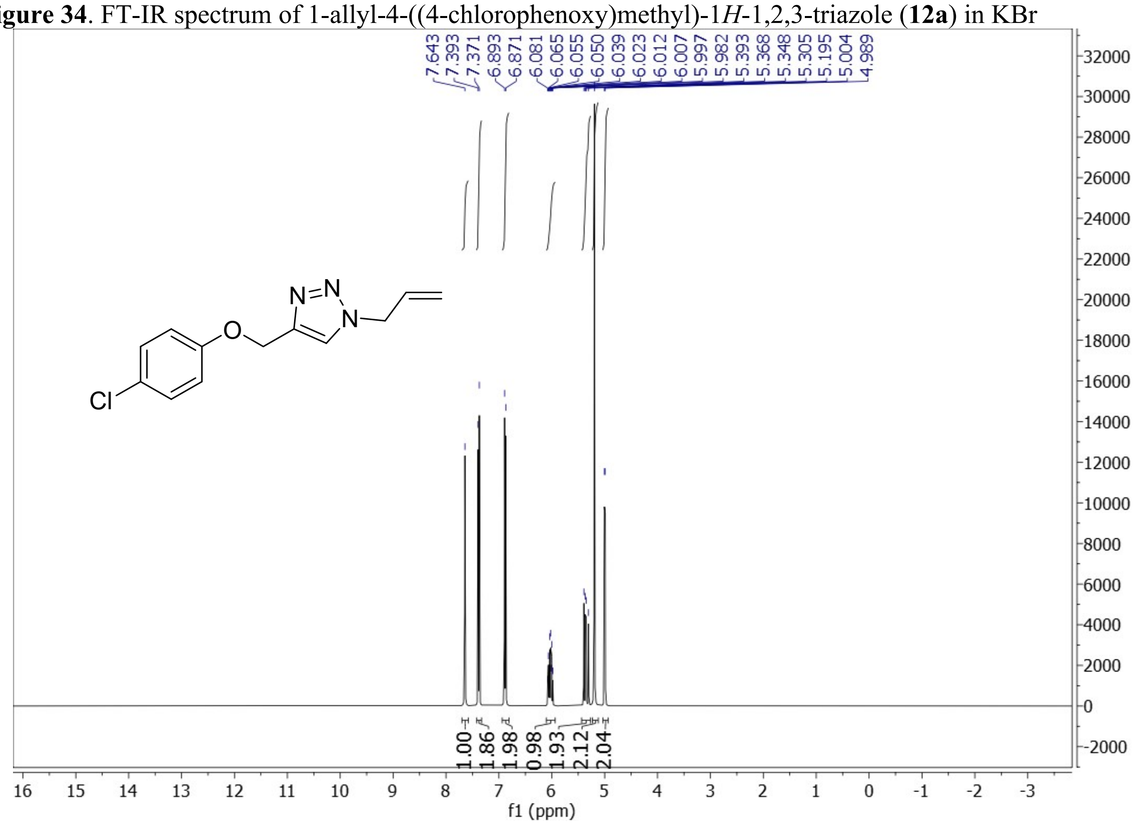

Figure 35. ^{1}H NMR spectrum of 1-allyl-4-((4-chlorophenoxy)methyl)-1H-1,2,3-triazole (**12a**) in $CDCl_3$

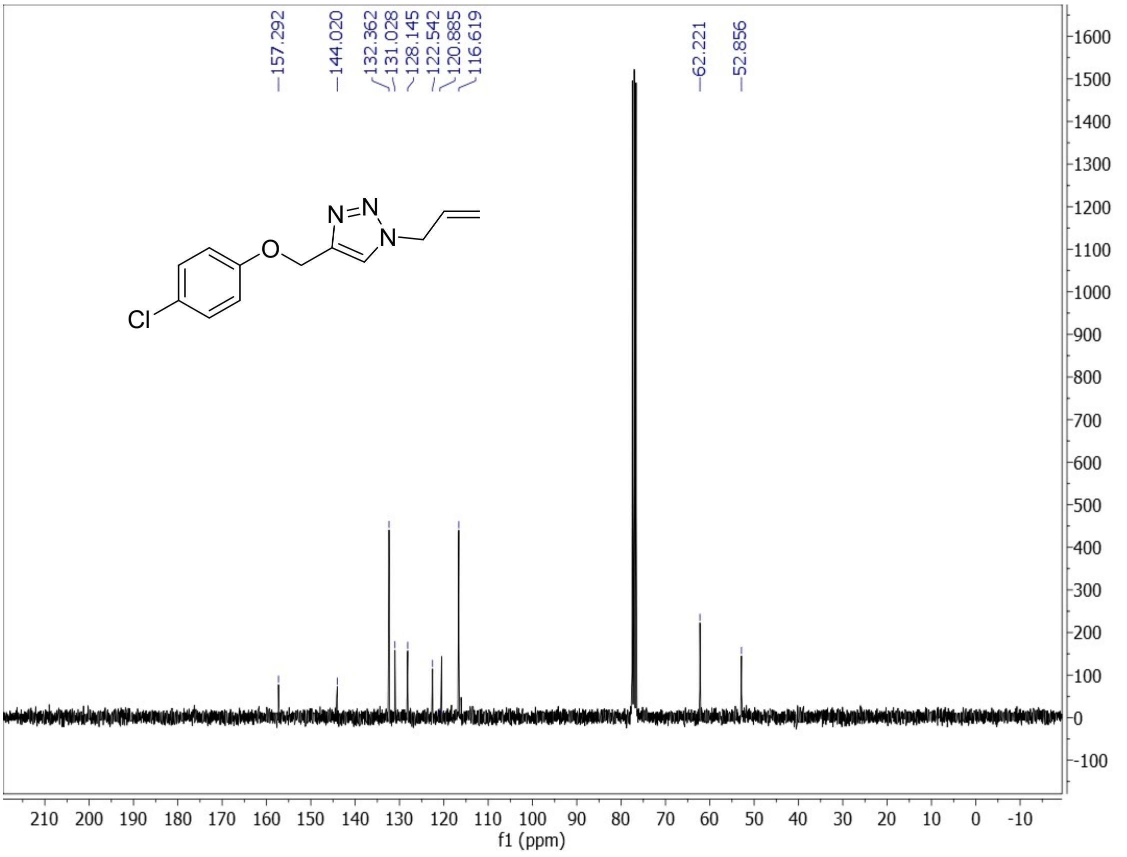

Figure 36. ^{13}C NMR spectrum of 1-allyl-4-((4-chlorophenoxy)methyl)-1*H*-1,2,3-triazole (**12a**) in $CDCl_3$

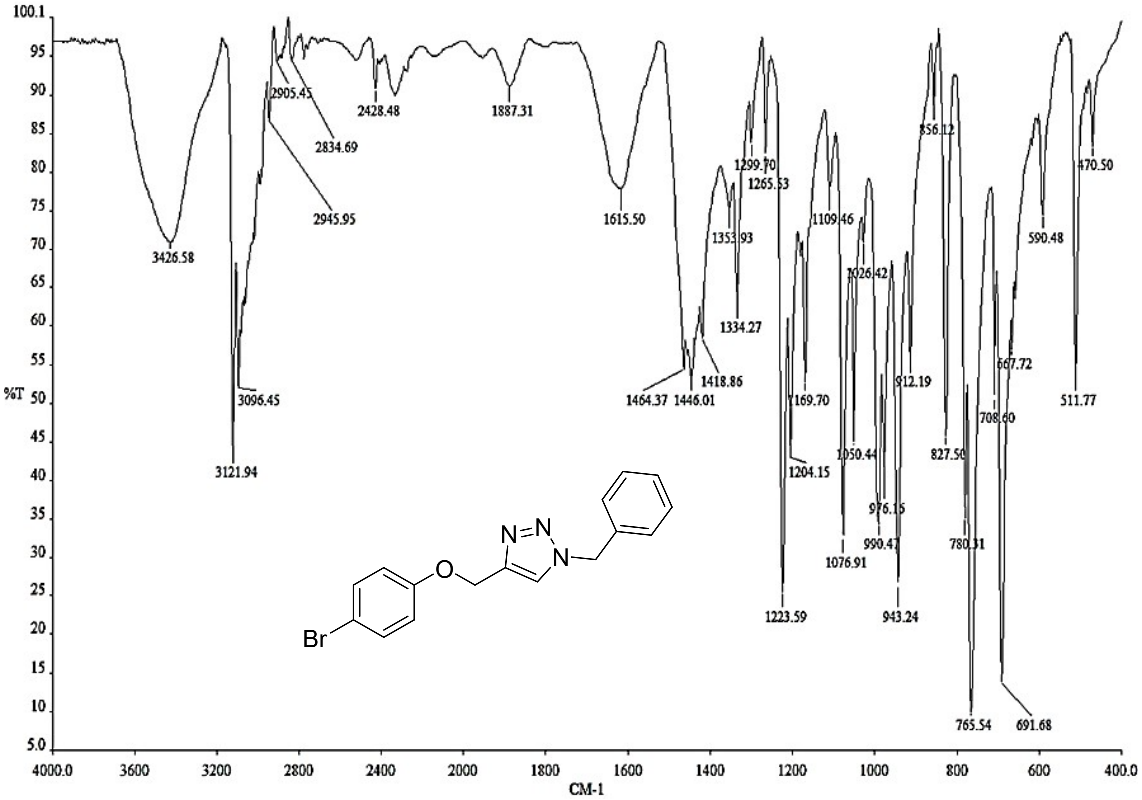

Figure 37. FT-IR spectrum of 1-benzyl-4-((4-bromophenoxy)methyl)-1*H*-1,2,3-triazole (**13a**) in KBr

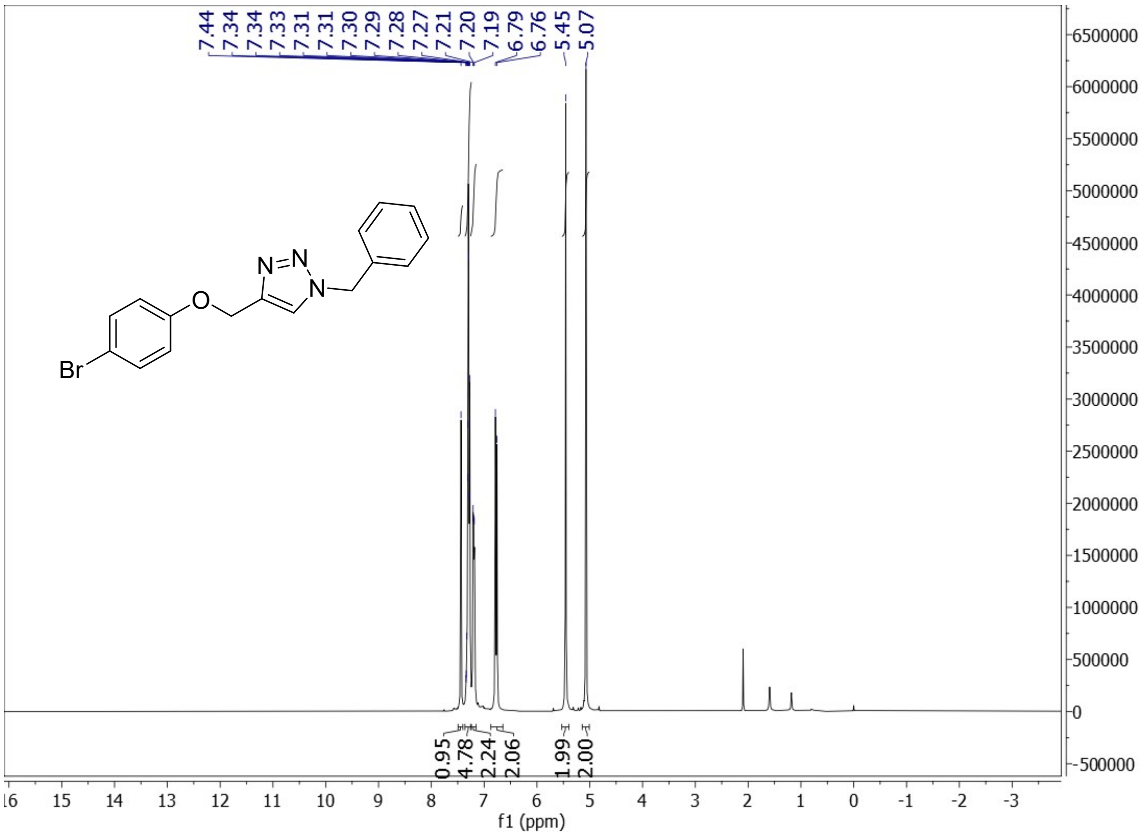

Figure 38. ^{1}H NMR spectrum of 1-benzyl-4-((4-bromophenoxy)methyl)-1*H*-1,2,3-triazole (**13a**) in $CDCl_3$

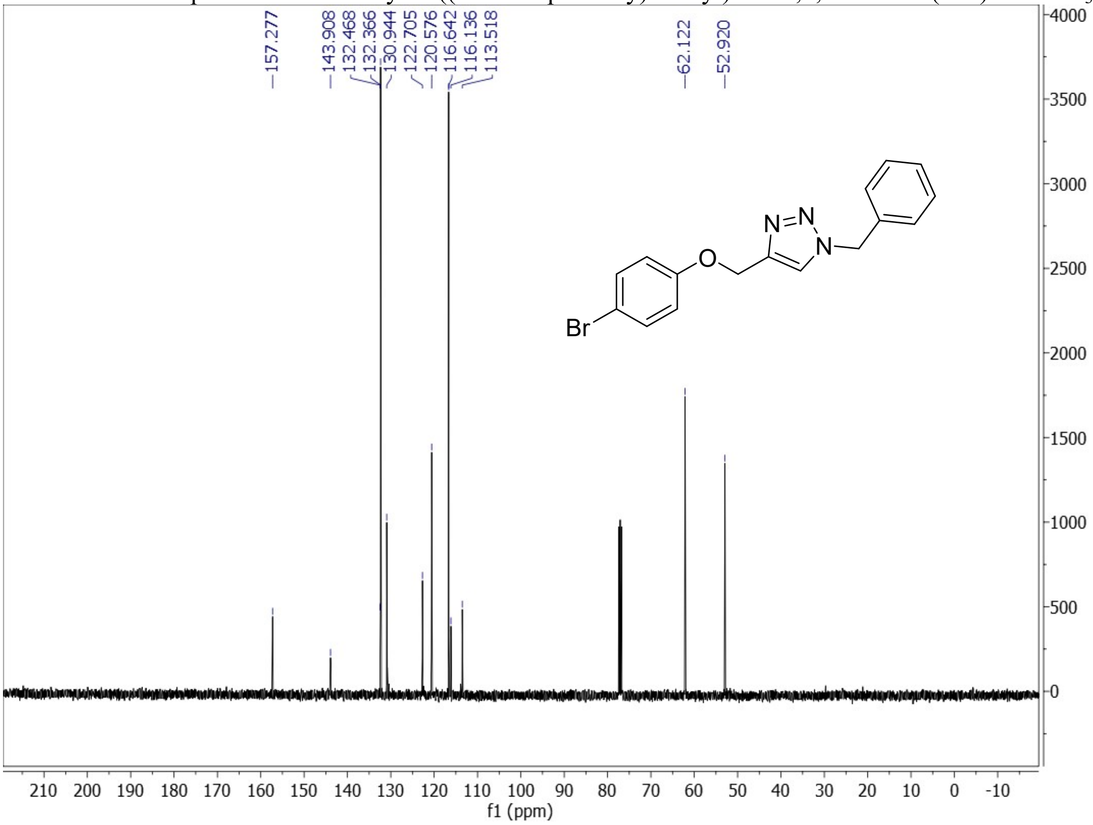

Figure 39. ^{13}C NMR spectrum of 1-benzyl-4-((4-bromophenoxy)methyl)-1*H*-1,2,3-triazole (**13a**) in $CDCl_3$

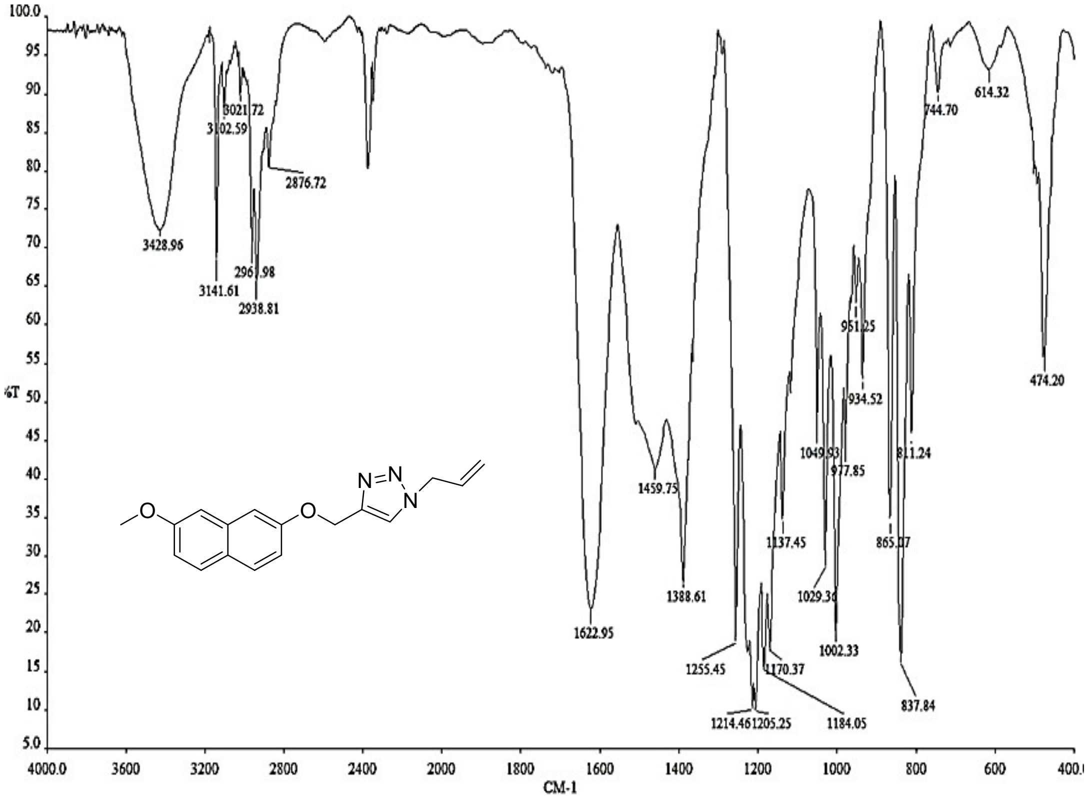

Figure 40. FT-IR spectrum of 1-allyl-4-(((7-methoxynaphthalen-2-yl)oxy)methyl)-1*H*-1,2,3-triazole (**14a**) in KBr

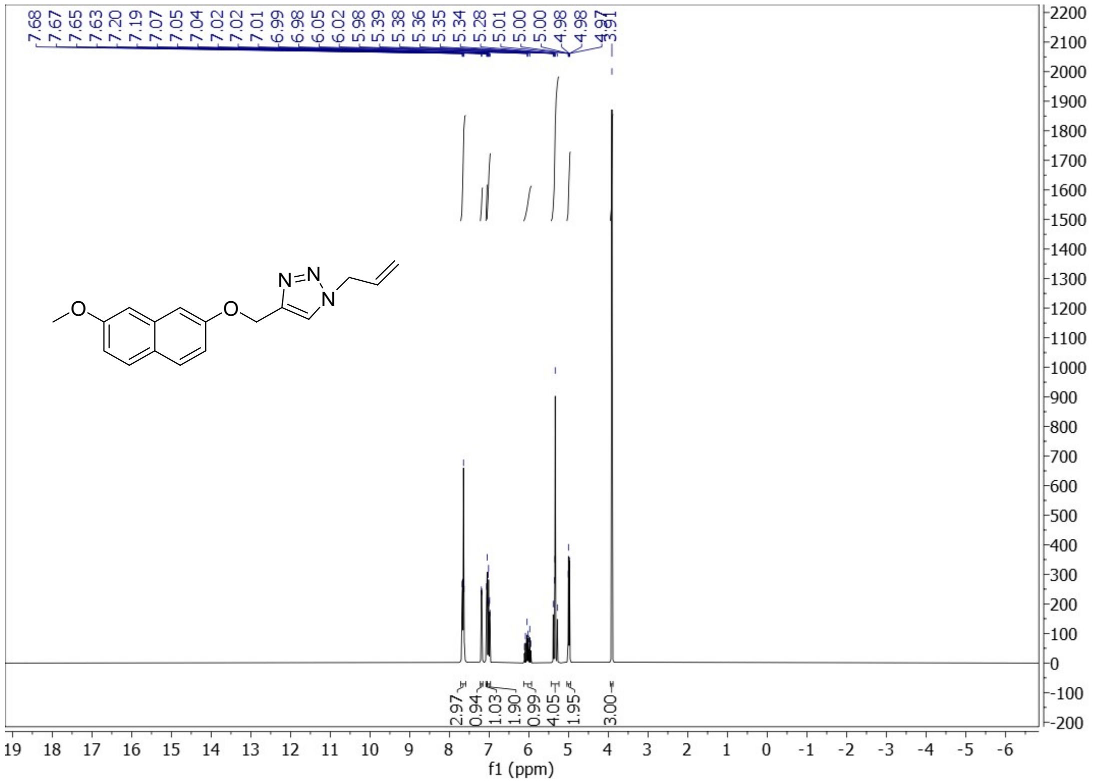

Figure 41. ^{1}H NMR spectrum of 1-allyl-4-(((7-methoxynaphthalen-2-yl)oxy)methyl)-1*H*-1,2,3-triazole (**14a**) in $CDCl_3$

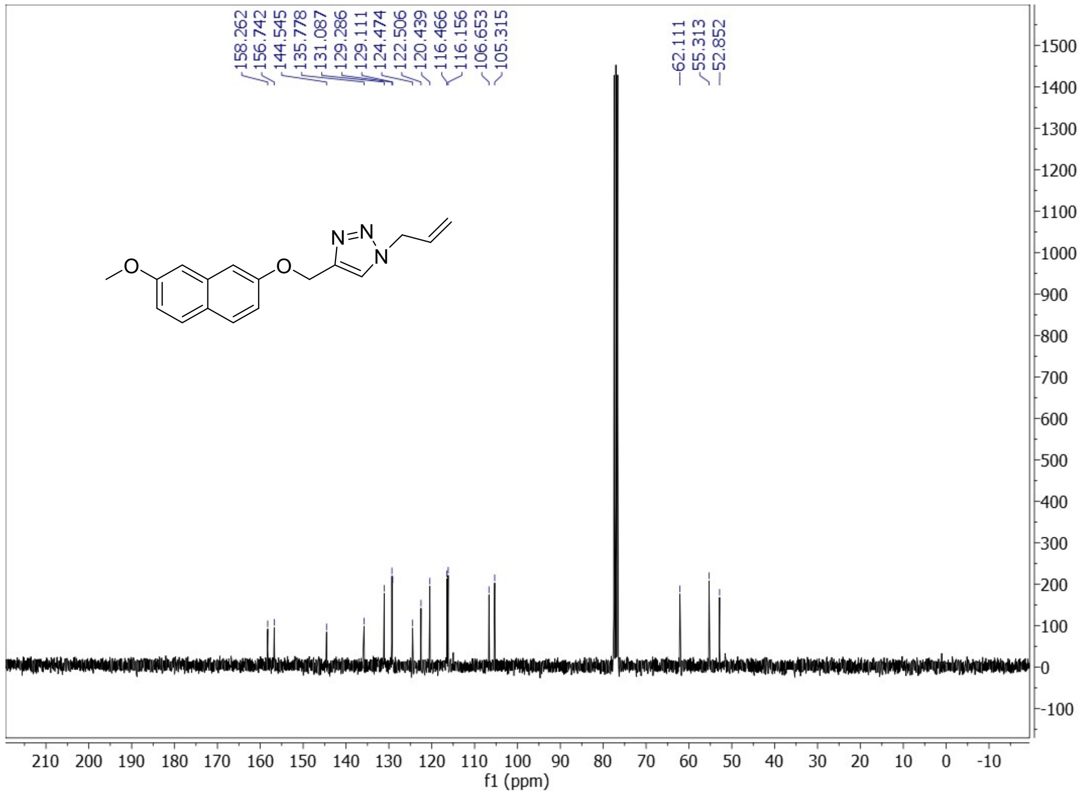

Figure 42. ^{13}C NMR spectrum of 1-allyl-4-(((7-methoxynaphthalen-2-yl)oxy)methyl)-1*H*-1,2,3-triazole (**14a**) in $CDCl_3$

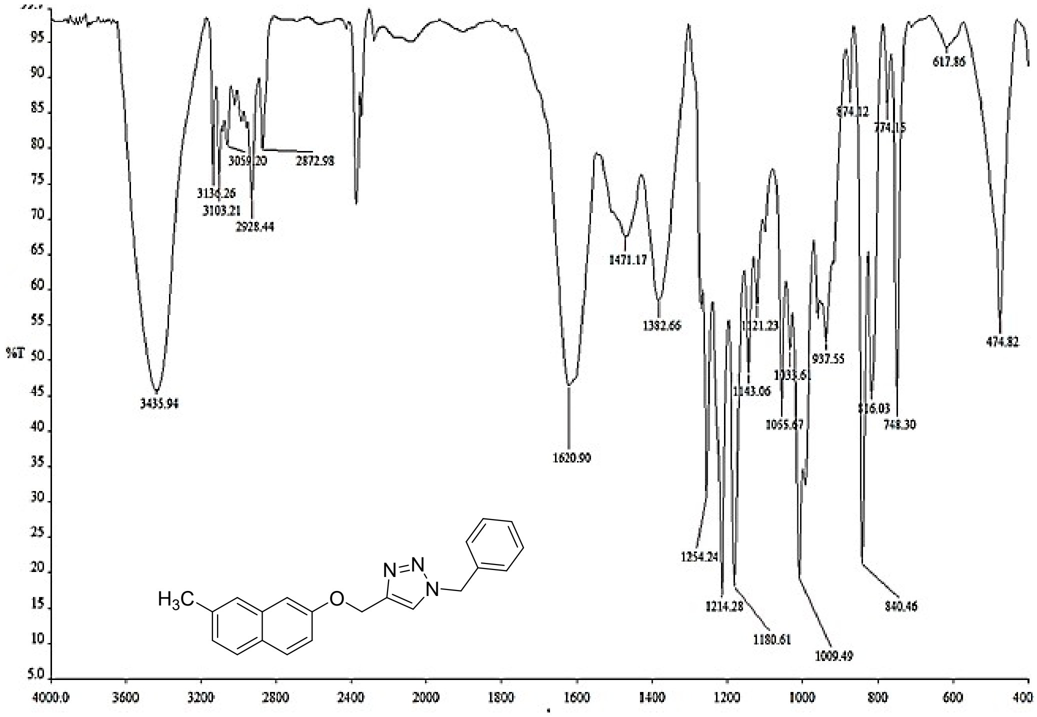

Figure 43. FT-IR spectrum of 1-benzyl-4-(((7-methylnaphthalen-2-yl)oxy)methyl)-1*H*-1,2,3-triazole (**15a**) in KBr

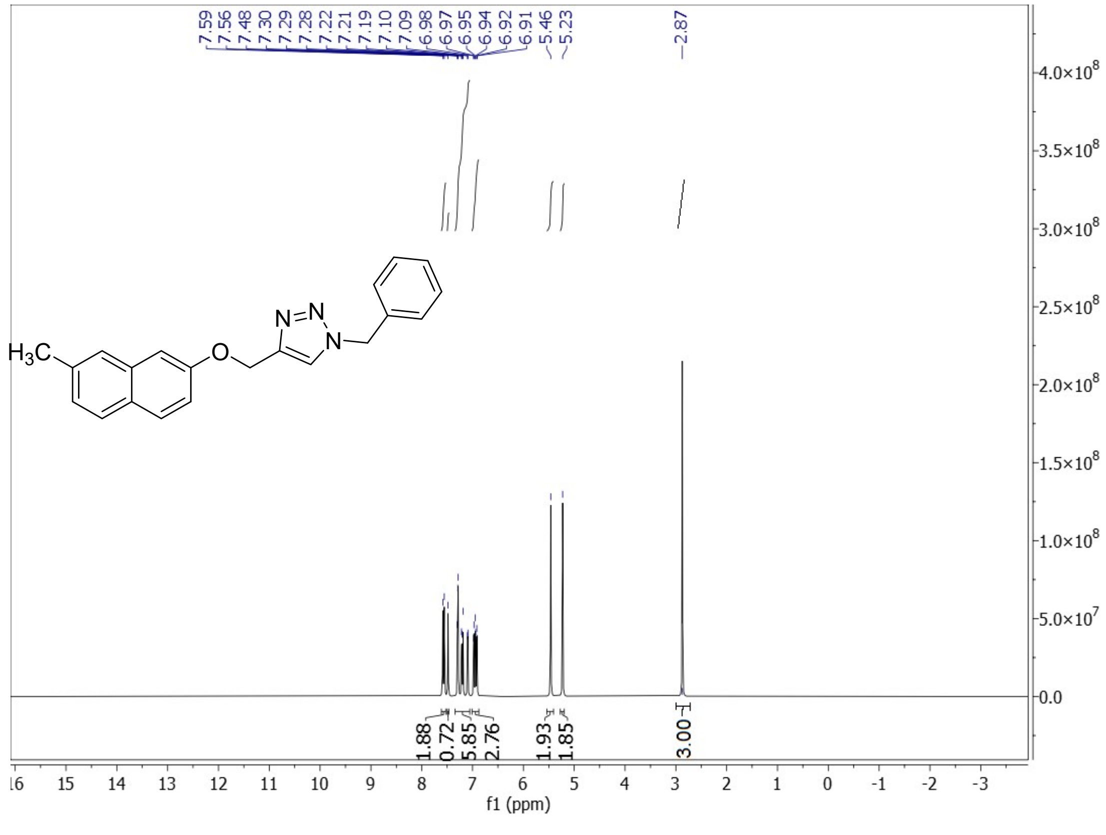

Figure 44. ^{1}H NMR spectrum of 1-benzyl-4-(((7-methylnaphthalen-2-yl)oxy)methyl)-1*H*-1,2,3-triazole (**15a**) in $CDCl_3$

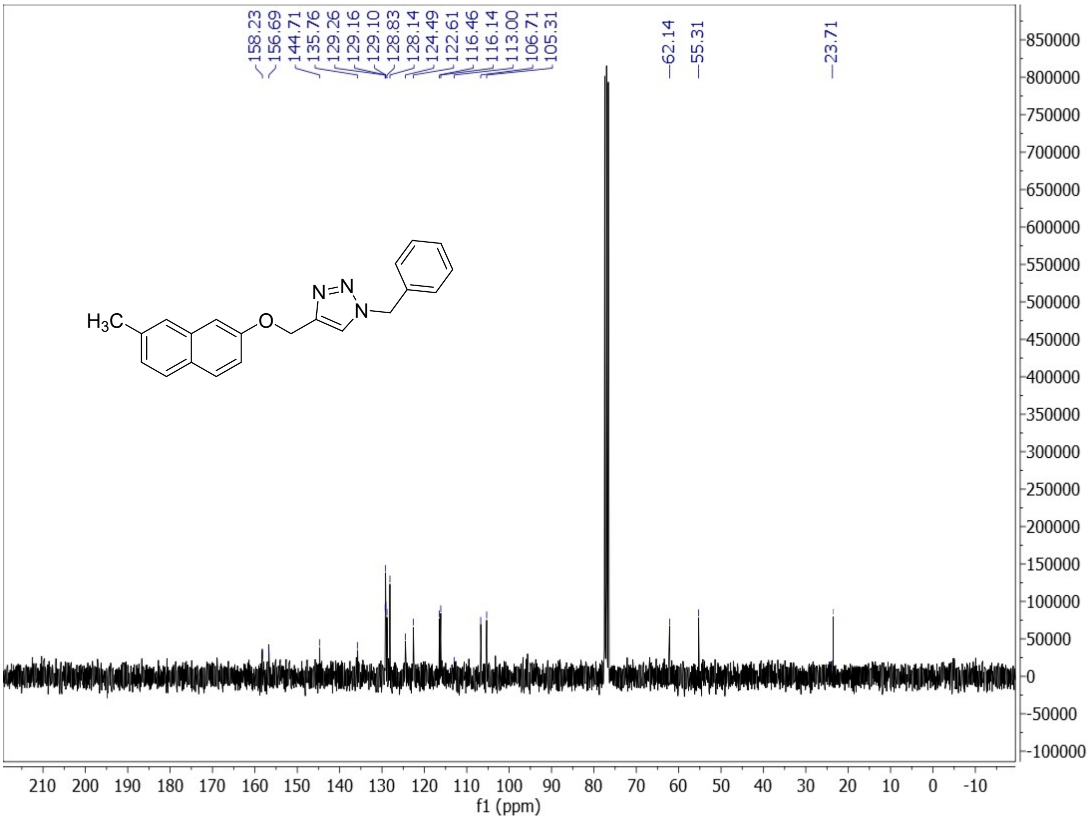

Figure 45. ^{13}C NMR spectrum of 1-benzyl-4-(((7-methylnaphthalen-2-yl)oxy)methyl)-1*H*-1,2,3-triazole (**15a**) in $CDCl_3$

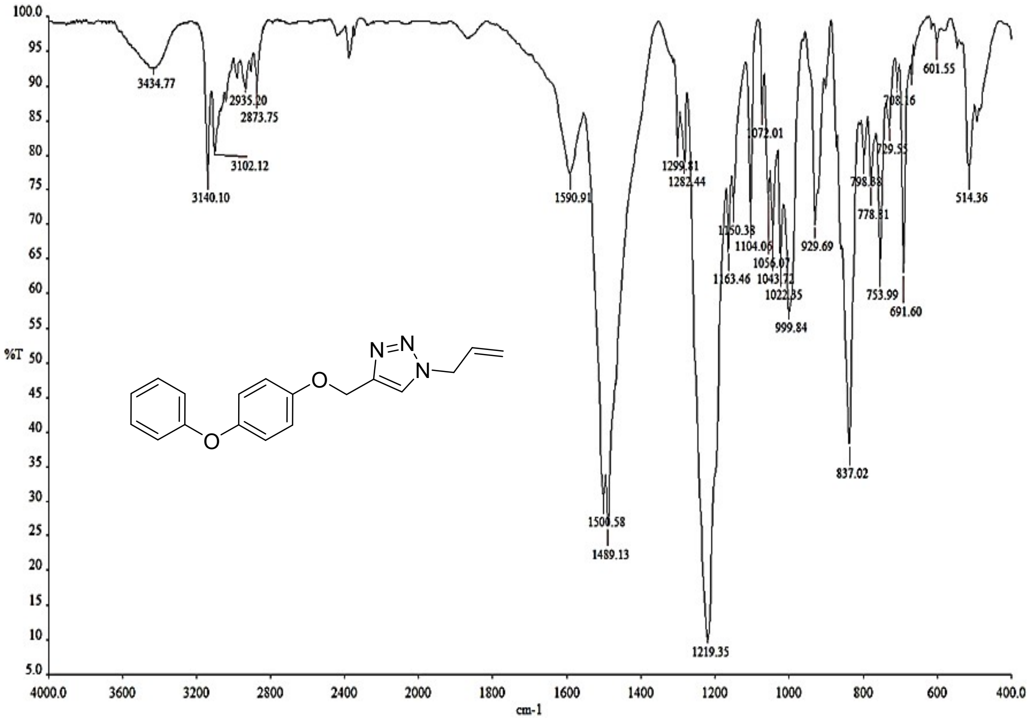

Figure 46. FT-IR spectrum of 1-allyl-4-((4-phenoxyphenoxy)methyl)-1*H*-1,2,3-triazole (**16a**) in KBr

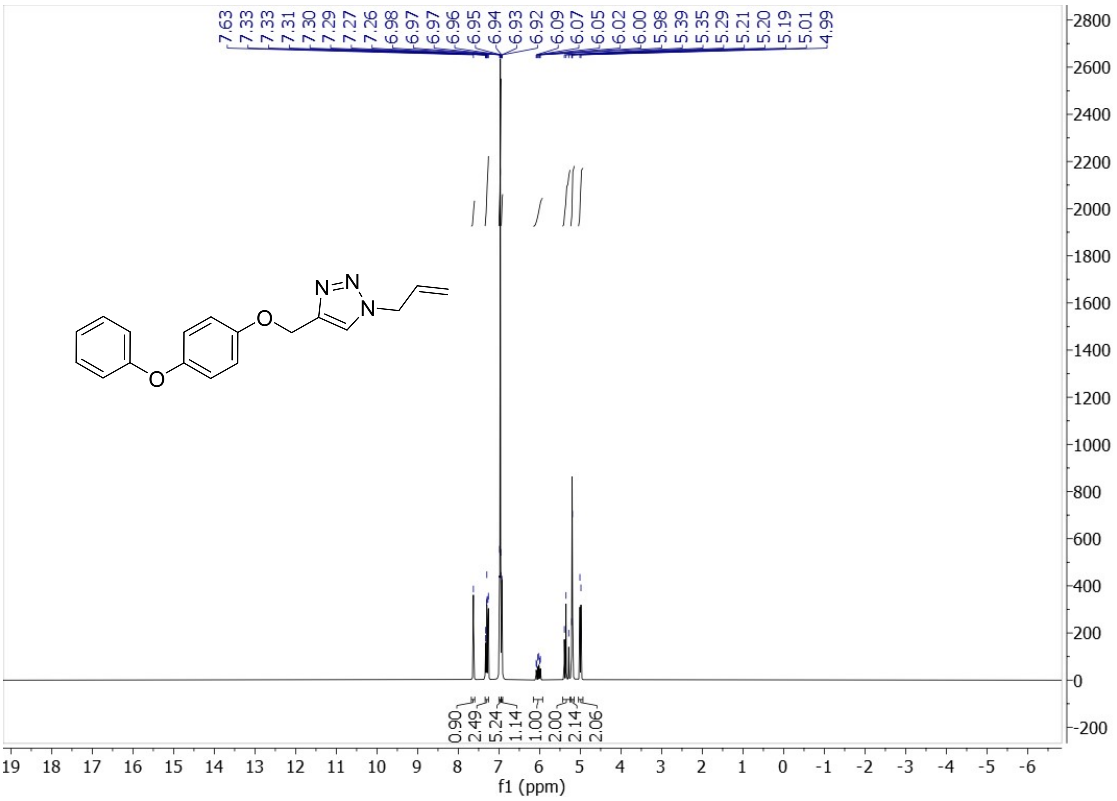

Figure 47. ^{1}H NMR spectrum of 1-allyl-4-((4-phenoxyphenoxy)methyl)-1*H*-1,2,3-triazole (**16a**) in $CDCl_3$

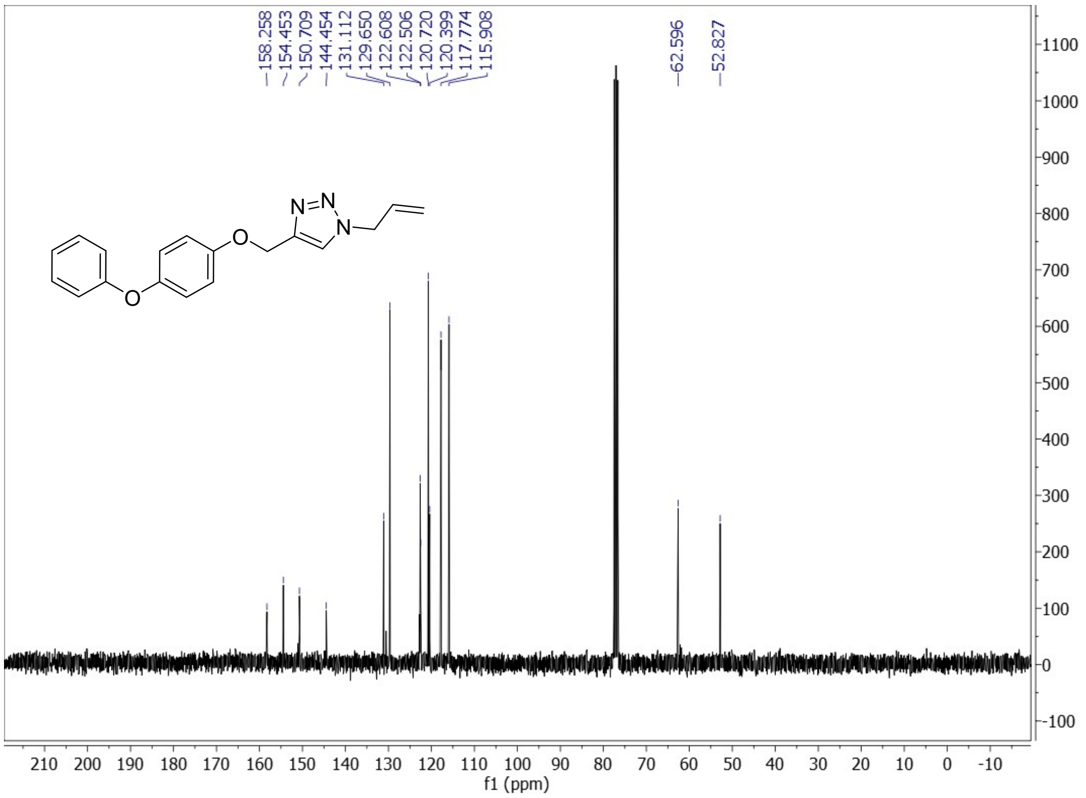

Figure 48. ^{13}C NMR spectrum of 1-allyl-4-((4-phenoxyphenoxy)methyl)-1*H*-1,2,3-triazole (**16a**) in $CDCl_3$

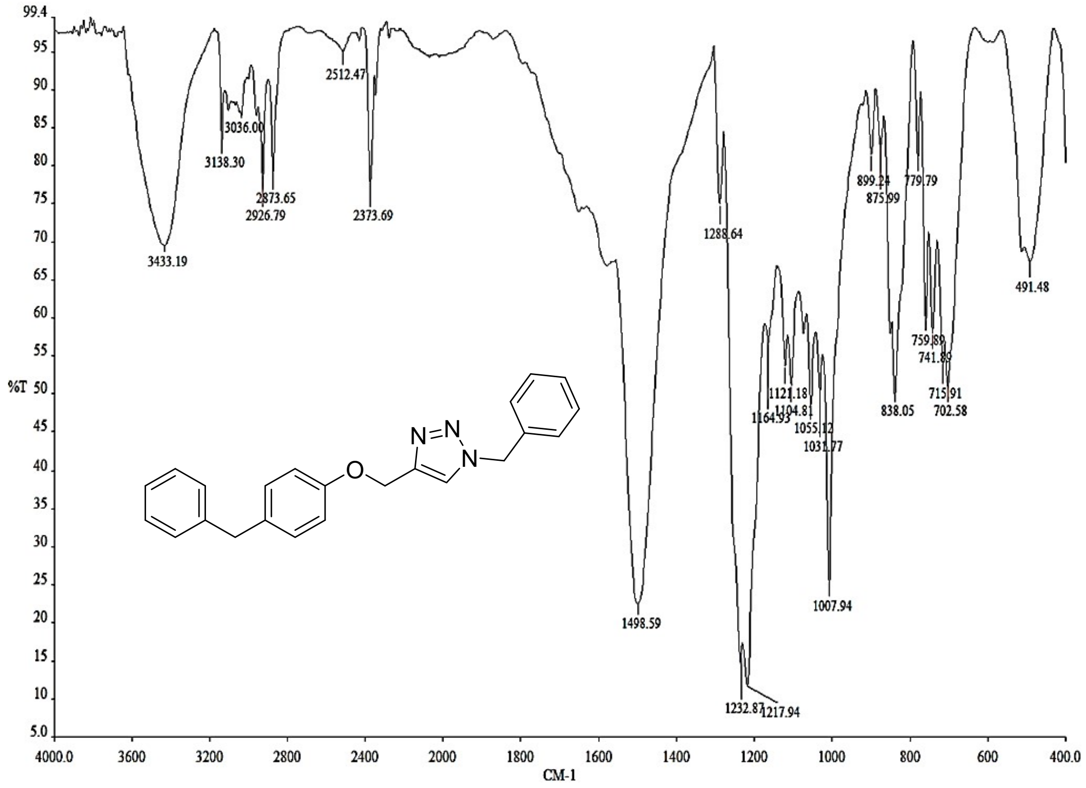

Figure 49. FT-IR spectrum of 1-benzyl-4-((4-benzylphenoxy)methyl)-1*H*-1,2,3-triazole (**17a**) in KBr

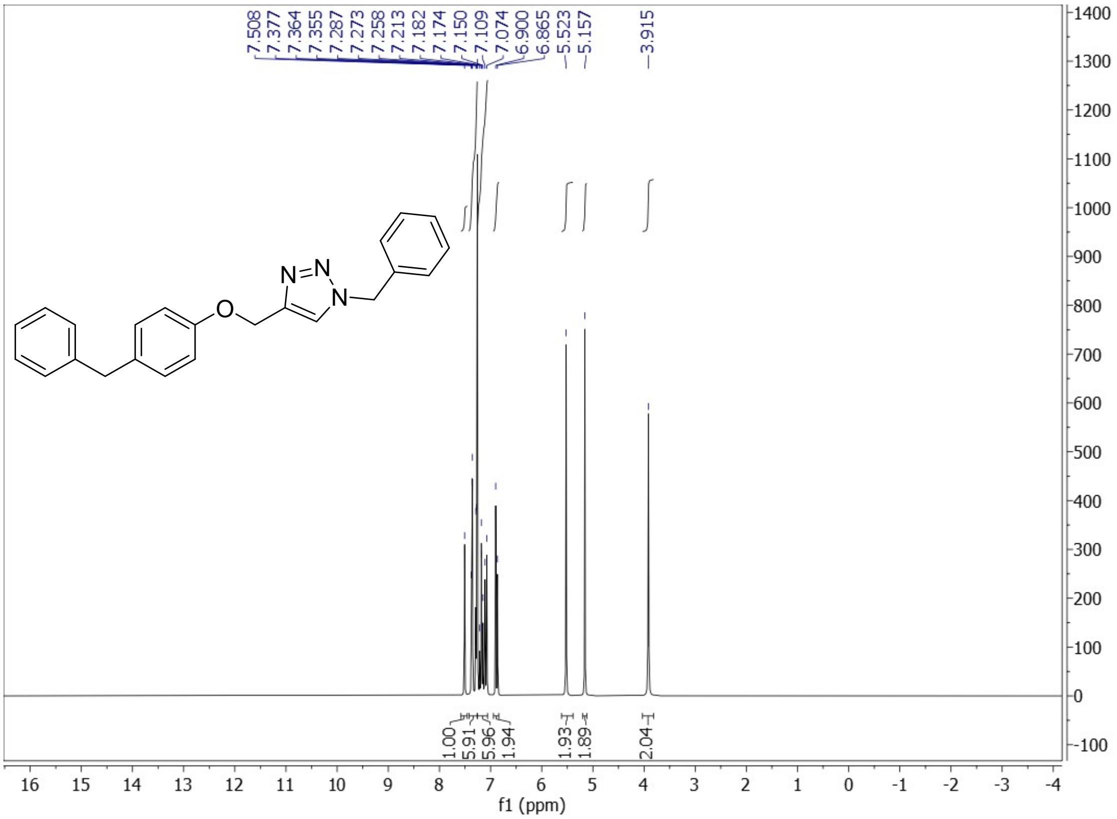

Figure 50. ^{1}H NMR spectrum of 1-benzyl-4-((4-benzylphenoxy)methyl)-1*H*-1,2,3-triazole (**17a**) in $CDCl_3$

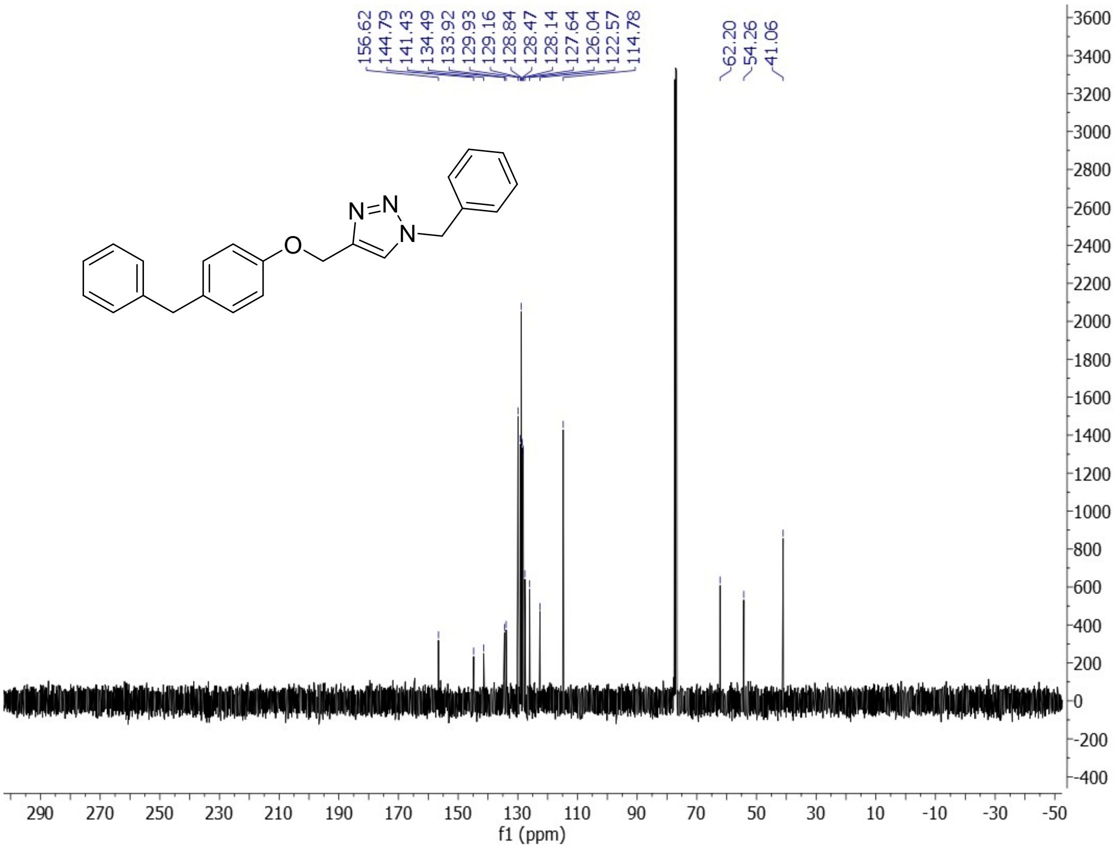

Figure 51. ^{13}C NMR spectrum of 1-benzyl-4-((4-benzylphenoxy)methyl)-1*H*-1,2,3-triazole (**17a**) in $CDCl_3$

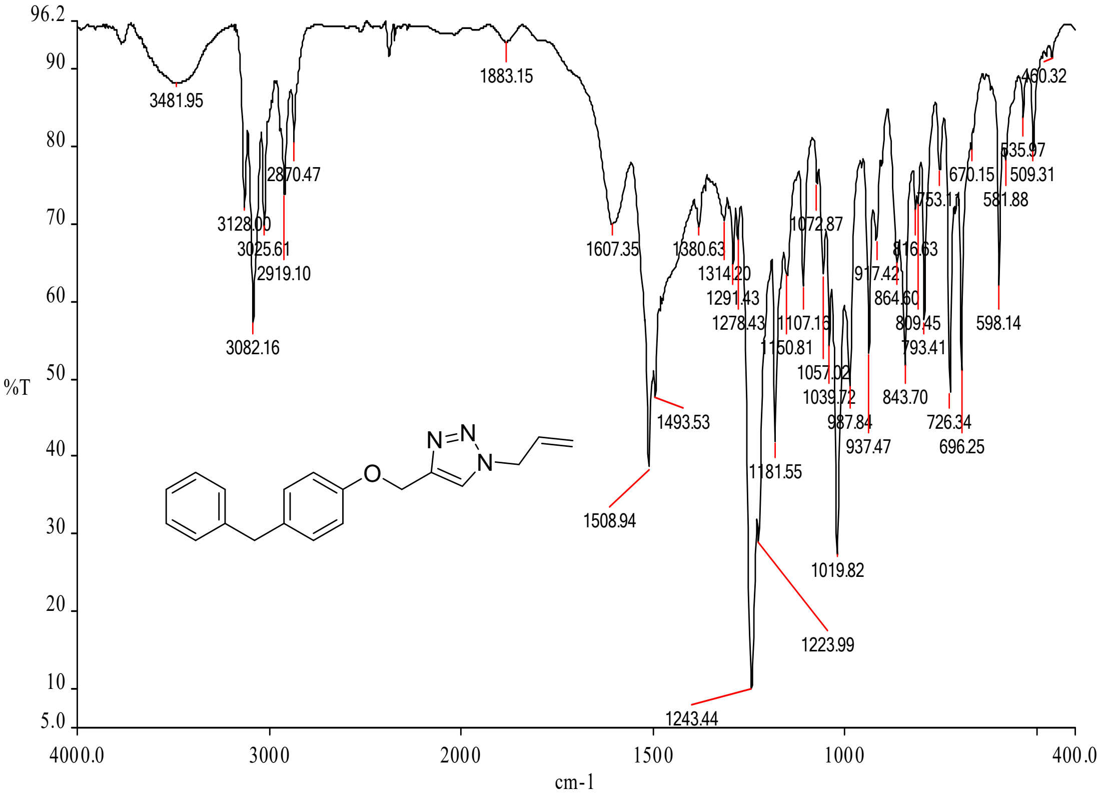

Figure 52. FT-IR spectrum of 1-allyl-4-((4-benzylphenoxy)methyl)-1*H*-1,2,3-triazole (**18a**) in KBr

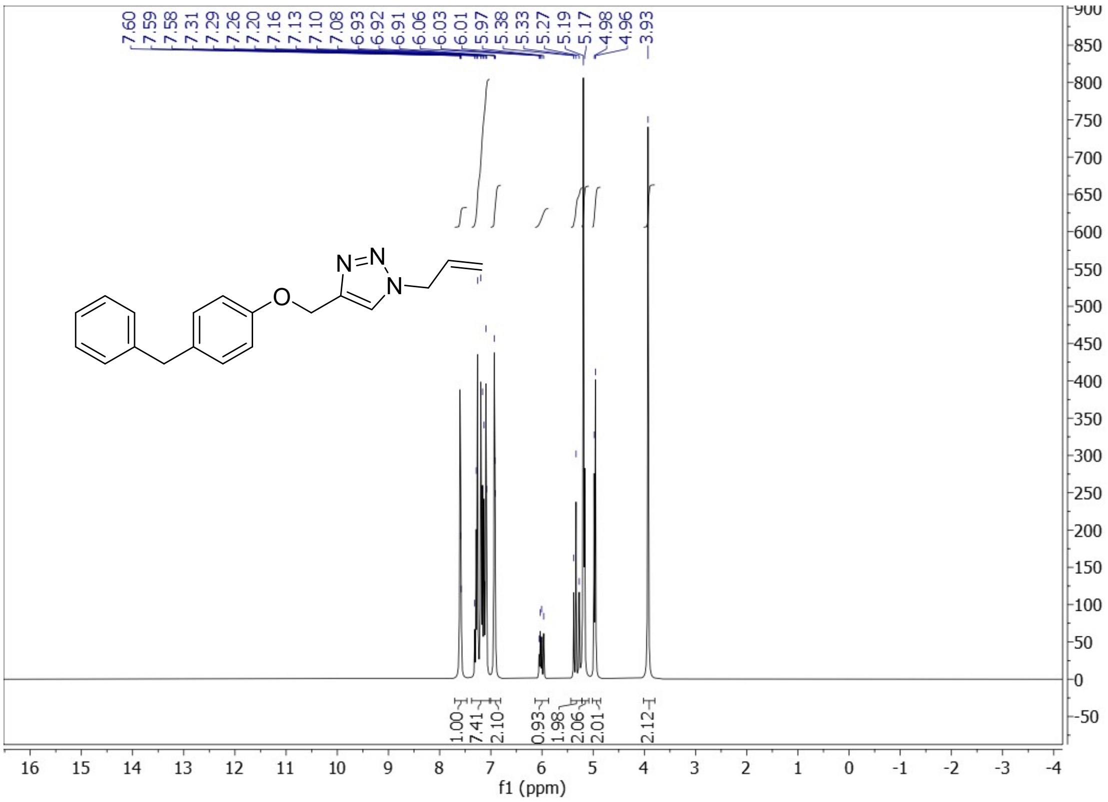

Figure 53. ^{1}H NMR spectrum of 1-allyl-4-((4-benzylphenoxy)methyl)-1*H*-1,2,3-triazole (**18a**) in $CDCl_3$

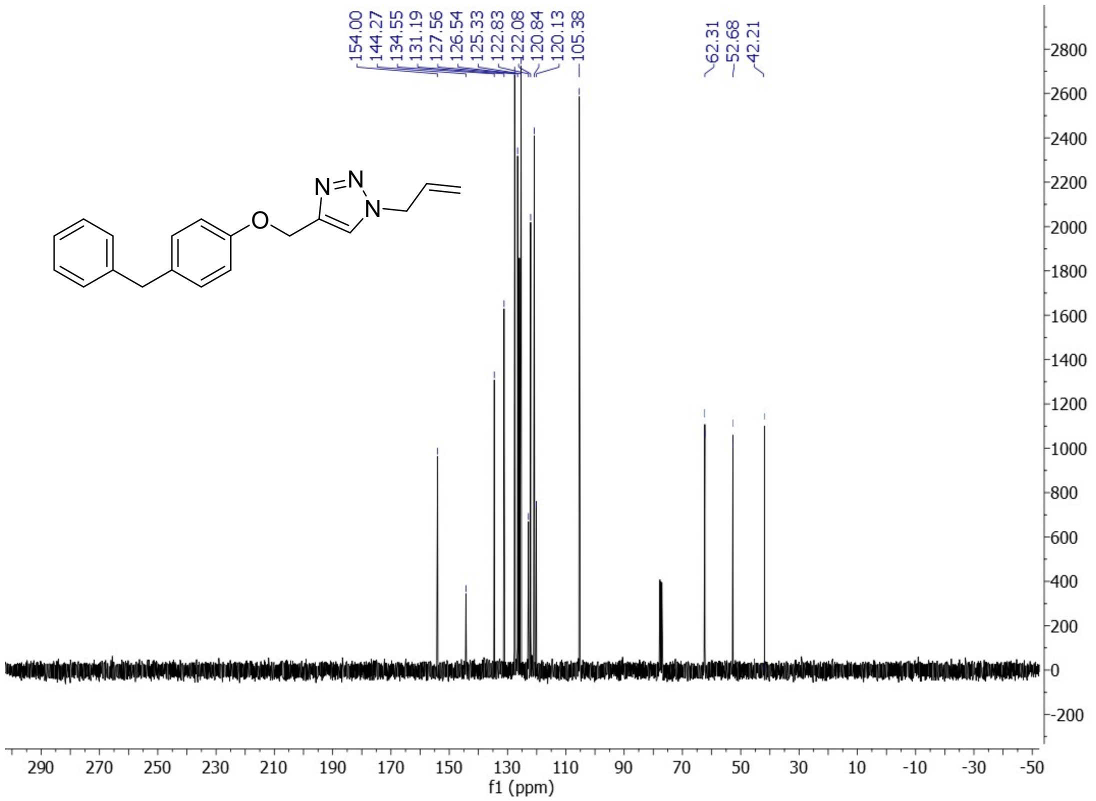

Figure 54. ^{13}C NMR spectrum of 1-allyl-4-((4-benzylphenoxy)methyl)-1*H*-1,2,3-triazole (**18a**) in $CDCl_3$

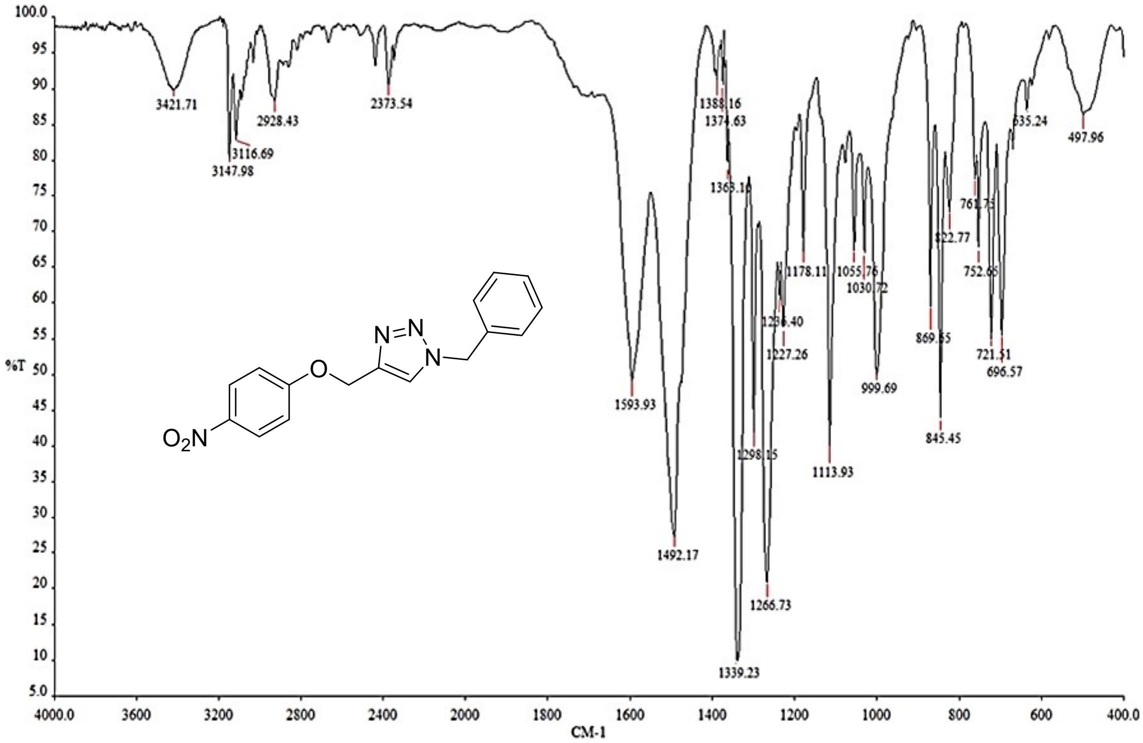

Figure 55. FT-IR spectrum of 1-benzyl-4-((4-nitrophenoxy)methyl)-1*H*-1,2,3-triazole (**19a**) in KBr

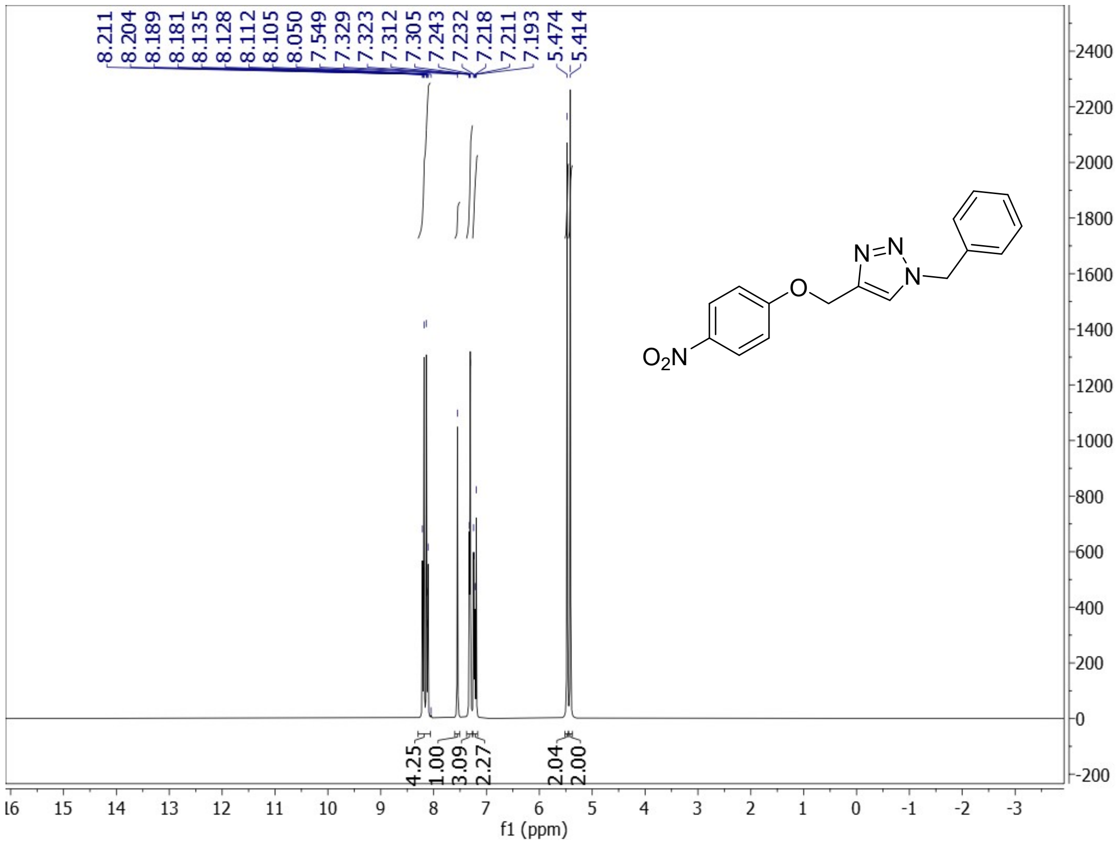

Figure 56. ^{1}H NMR spectrum of 1-benzyl-4-((4-nitrophenoxy)methyl)-1*H*-1,2,3-triazole (**19a**) in $CDCl_3$

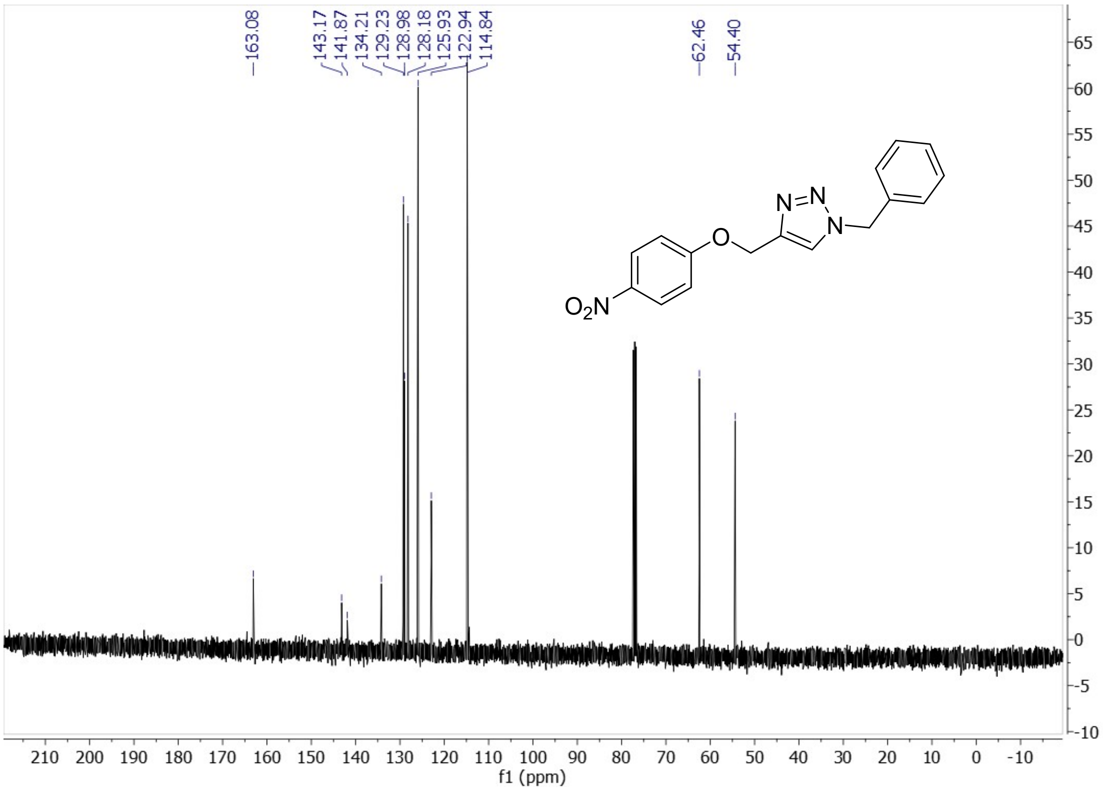

Figure 57. ^{13}C NMR spectrum of 1-benzyl-4-((4-nitrophenoxy)methyl)-1*H*-1,2,3-triazole (**19a**) in $CDCl_3$

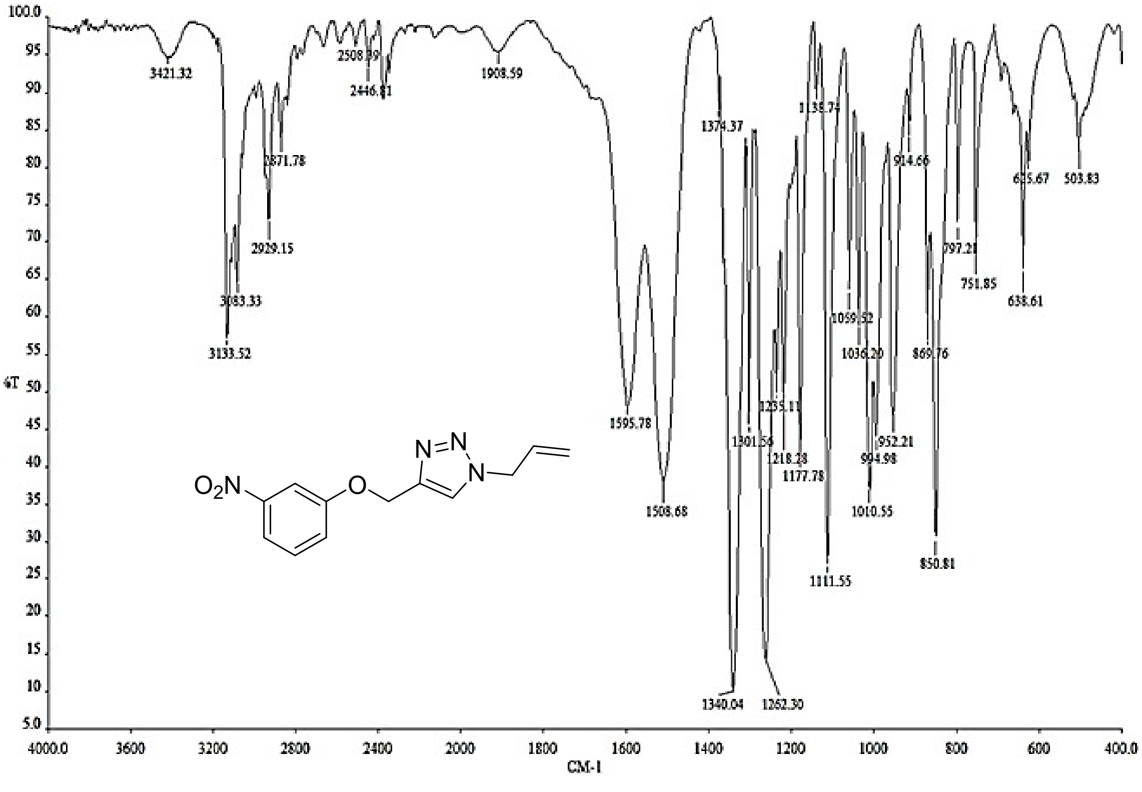

Figure 58. FT-IR spectrum of 1-allyl-4-((3-nitrophenoxy)methyl)-1*H*-1,2,3-triazole (**20a**) in KBr

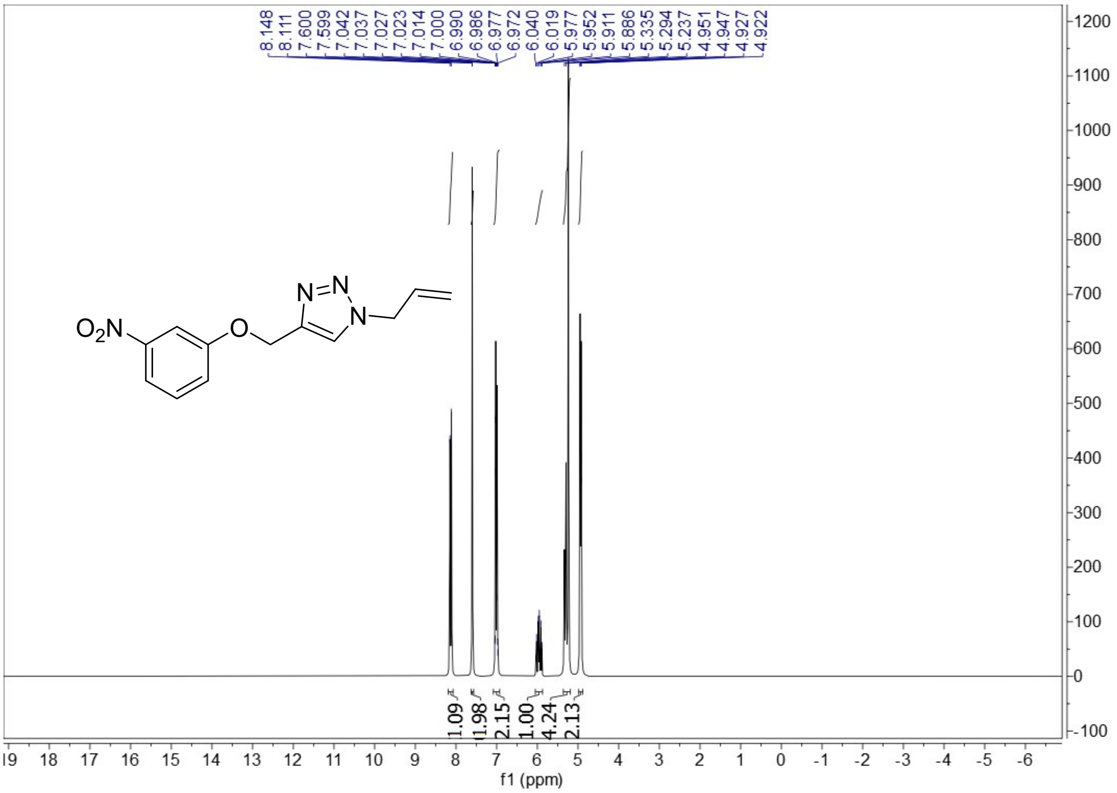

Figure 59. ^{1}H NMR spectrum of 1-allyl-4-((3-nitrophenoxy)methyl)-1*H*-1,2,3-triazole (**20a**) in $CDCl_3$

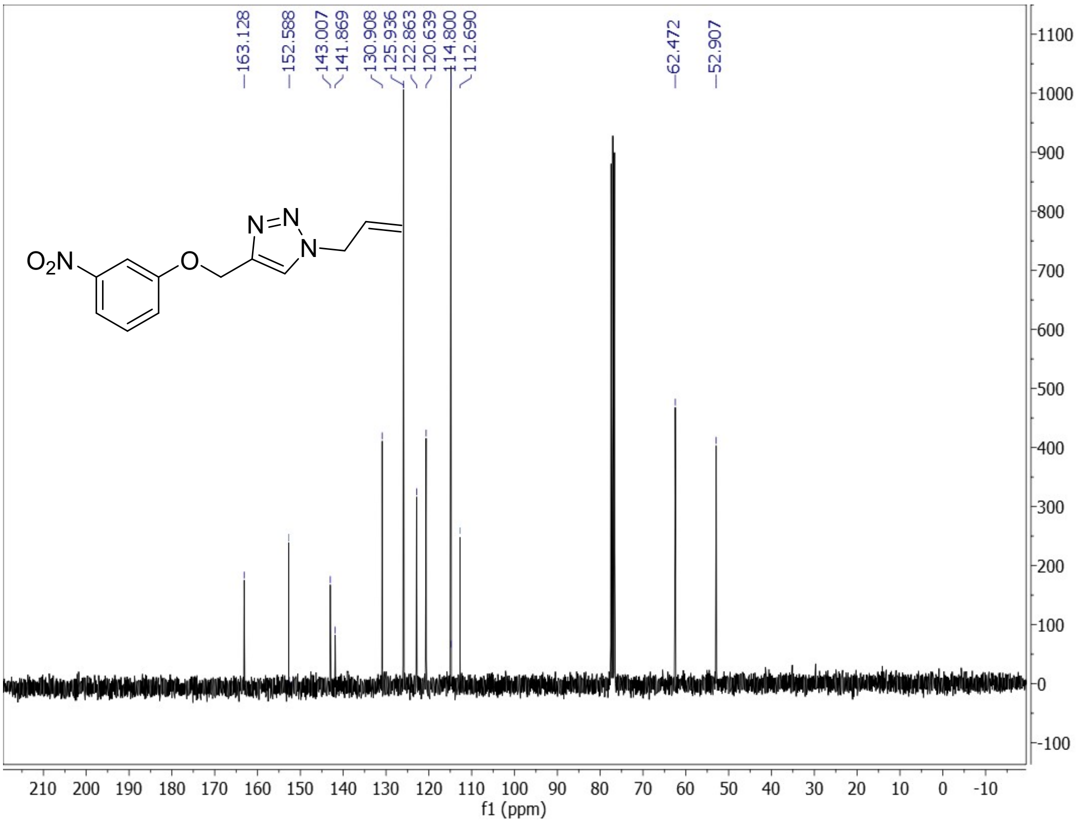

Figure 60. ^{13}C NMR spectrum of 1-allyl-4-((3-nitrophenoxy)methyl)-1*H*-1,2,3-triazole (**20a**) in $CDCl_3$

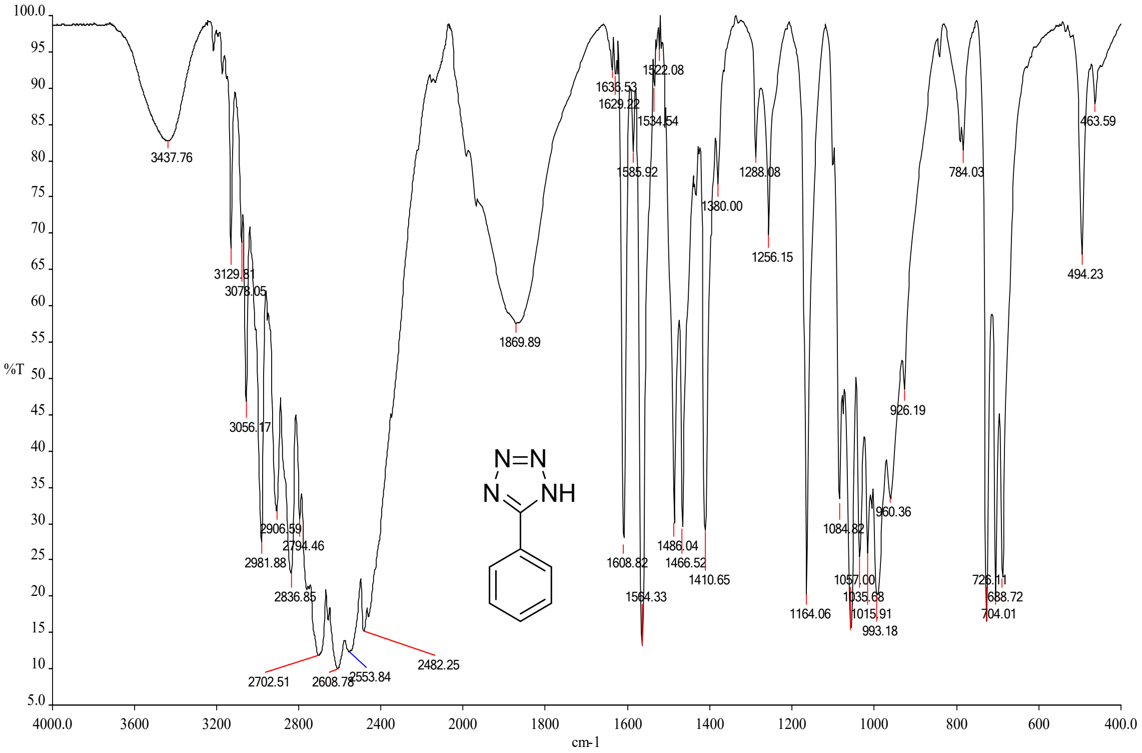

Figure 61. FT-IR spectrum of 5-phenyl-1*H*-tetrazole (**21a**) in KBr

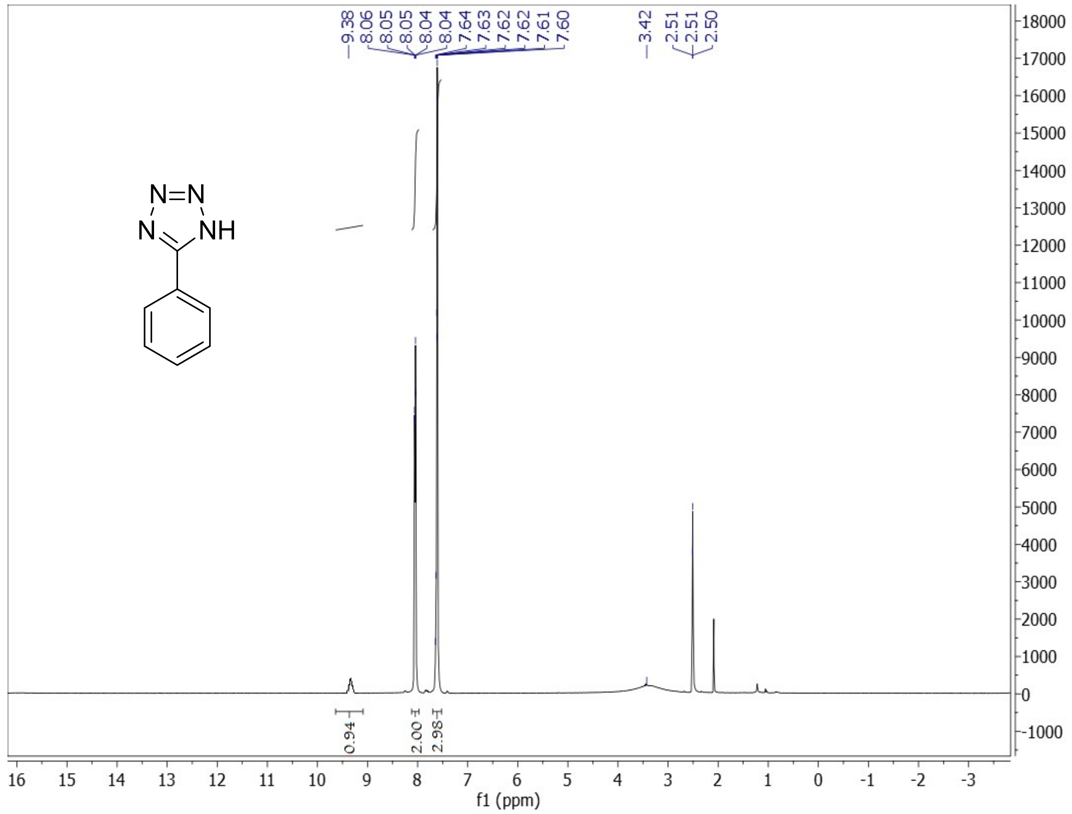

Figure 62. ^{1}H NMR spectrum of 5-phenyl-1*H*-tetrazole (**21a**) in DMSO-d_6

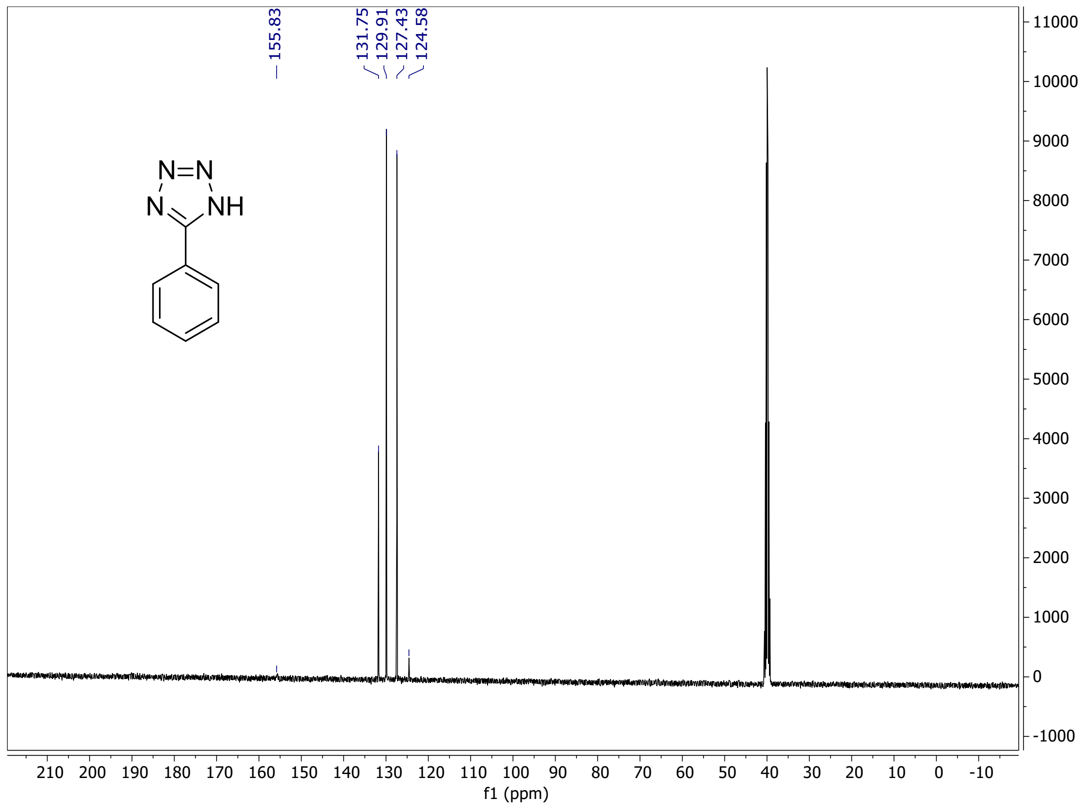

Figure 63. ^{13}C NMR spectrum of 5-phenyl-1*H*-tetrazole (**21a**) in DMSO-d_6

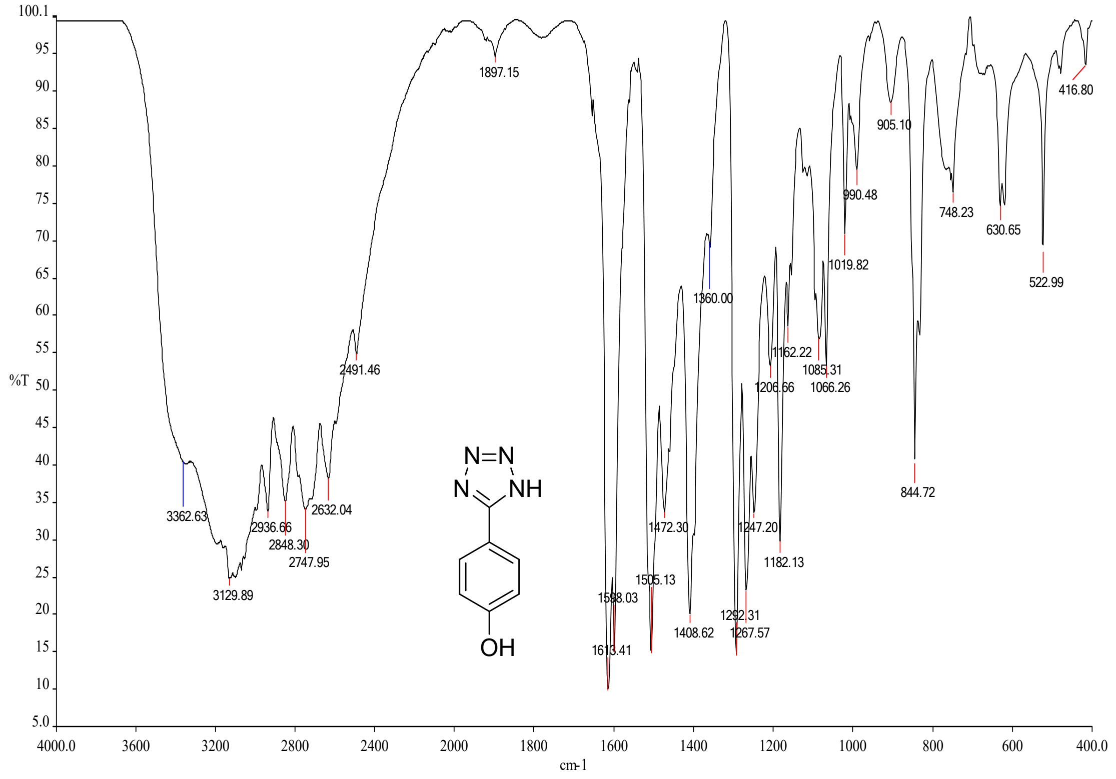

Figure 64. FT-IR spectrum of 4-(1*H*-tetrazol-5-yl)phenol (**22a**) in KBr

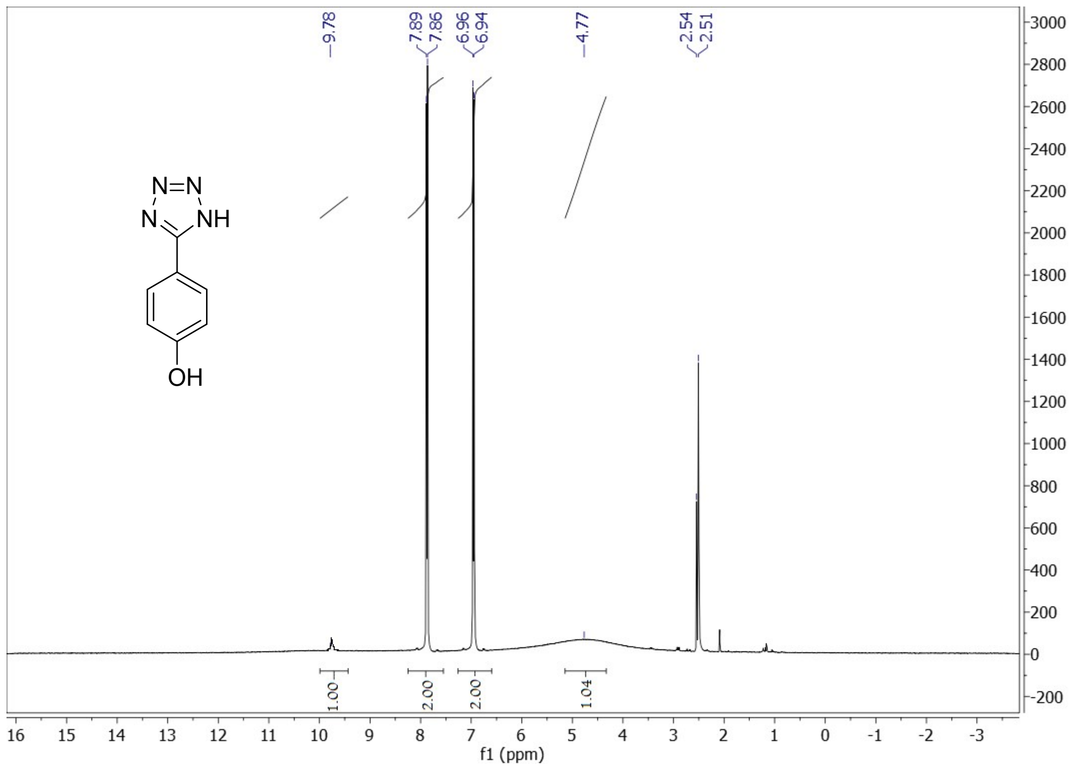

Figure 65. ^{1}H NMR spectrum of 4-(1*H*-tetrazol-5-yl)phenol (**22a**) in DMSO-d_6

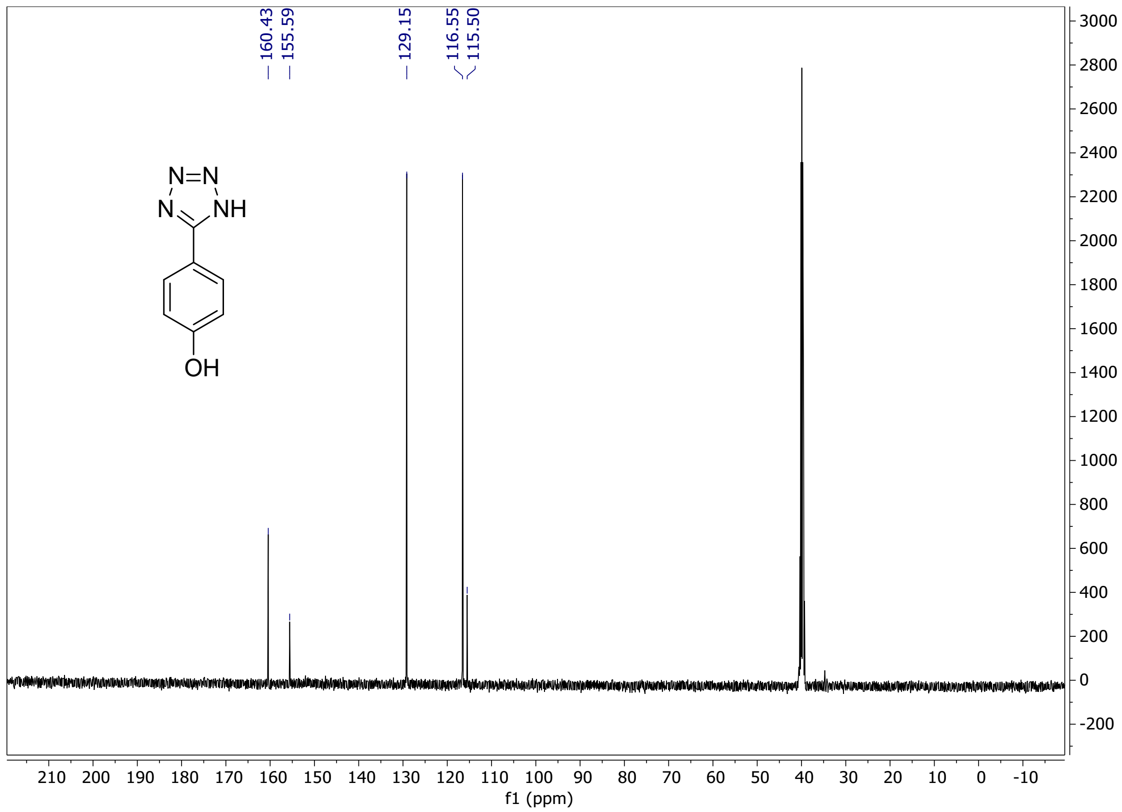

Figure 66. ^{13}C NMR spectrum of 4-(1*H*-tetrazol-5-yl)phenol (**22a**) in DMSO-d_6

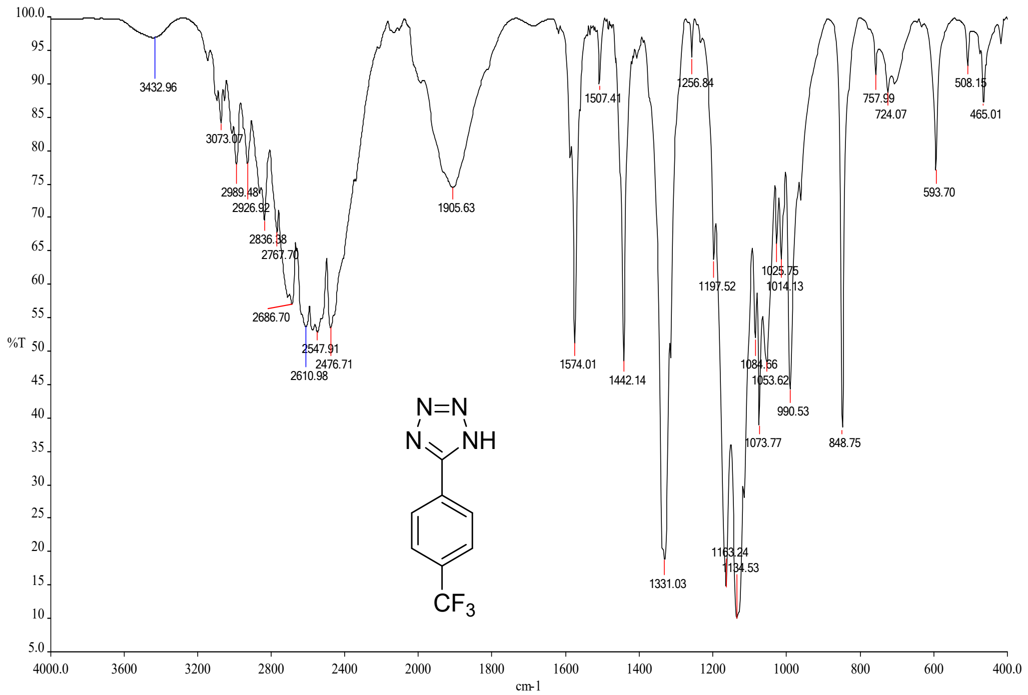

Figure 67. FT-IR spectrum of 5-(4-(trifluoromethyl)phenyl)-1*H*-tetrazole (**23a**) in KBr

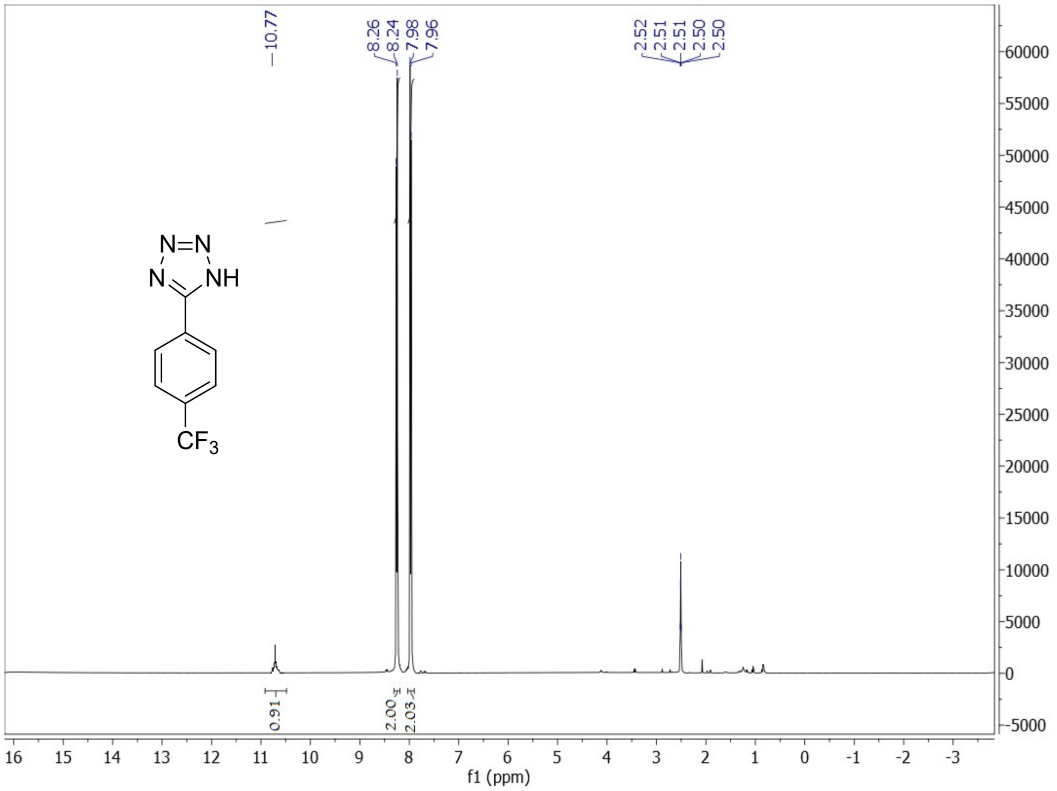

Figure 68. ^{1}H NMR spectrum of 5-(4-(trifluoromethyl)phenyl)-1*H*-tetrazole (**23a**) in DMSO-d_6

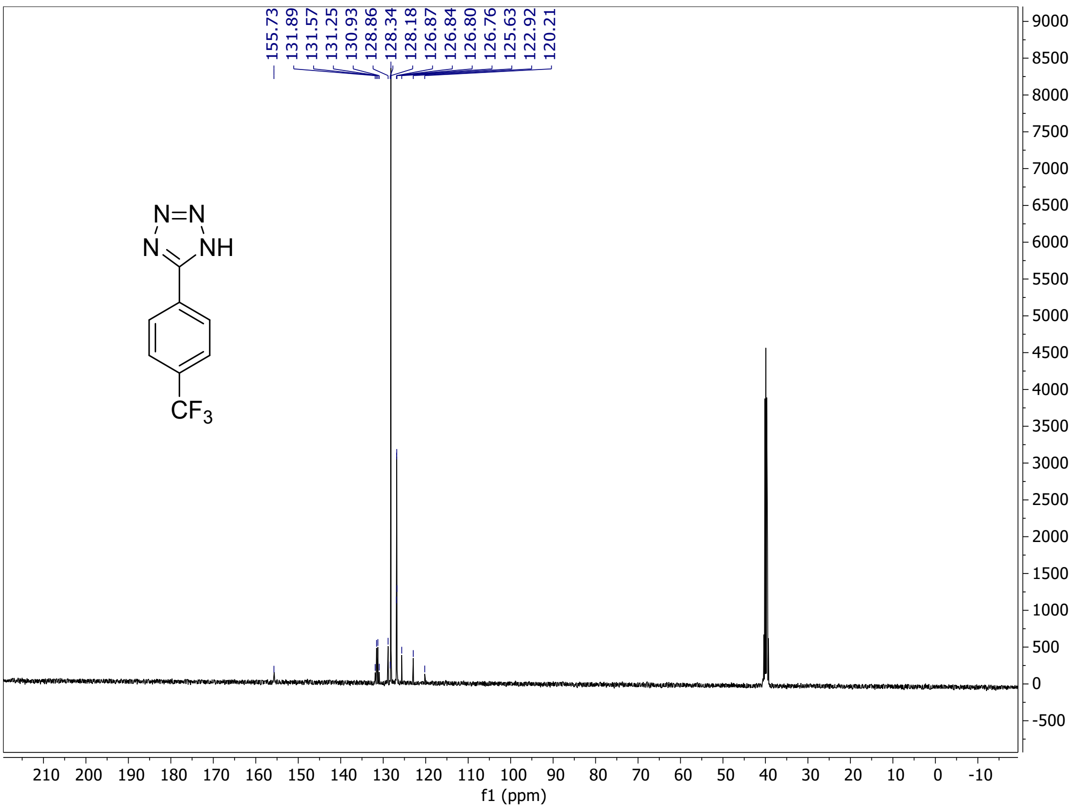

Figure 69. ^{13}C NMR spectrum of 5-(4-(trifluoromethyl)phenyl)-1*H*-tetrazole (**23a**) in DMSO-d_6

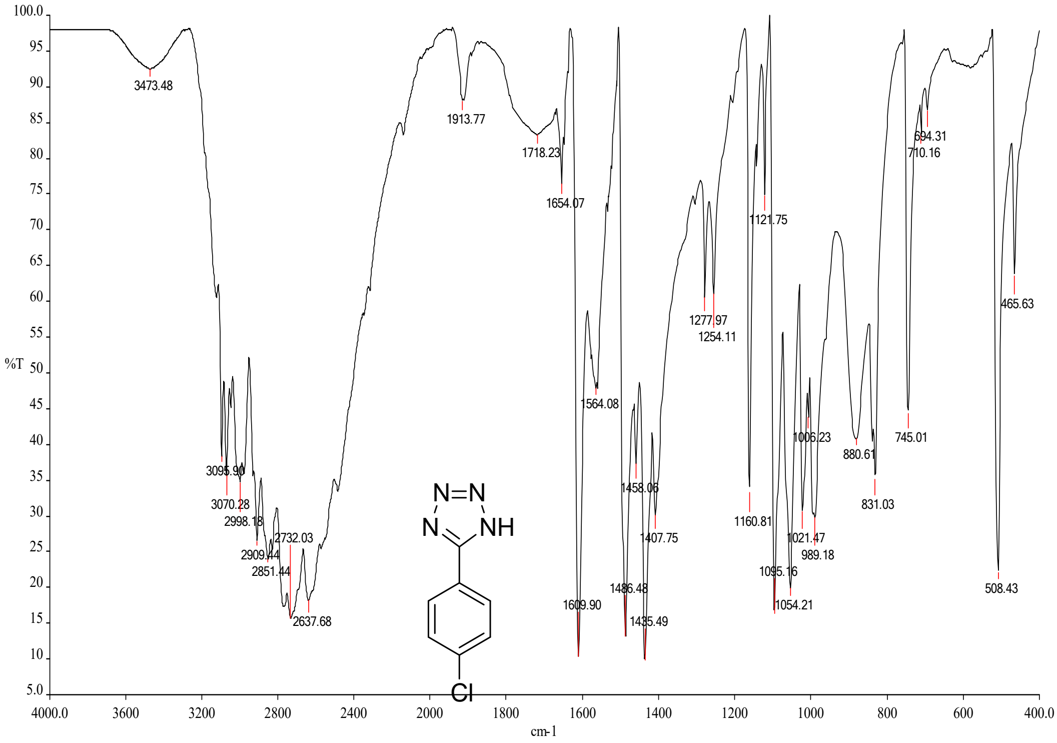

Figure 70. FT-IR spectrum of 5-(4-chlorophenyl)-1*H*-tetrazole (**24a**) in KBr

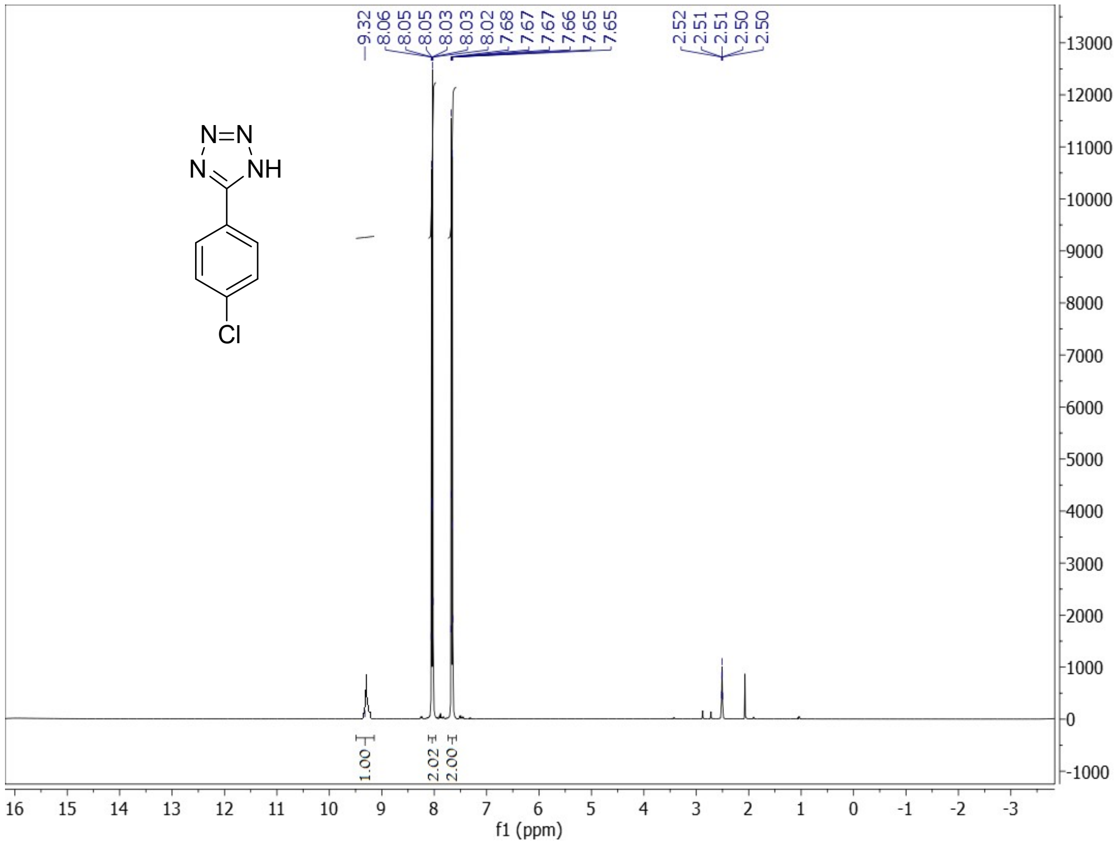

Figure 71. ^{1}H NMR spectrum of 5-(4-chlorophenyl)-1*H*-tetrazole (**24a**) in DMSO-d_6

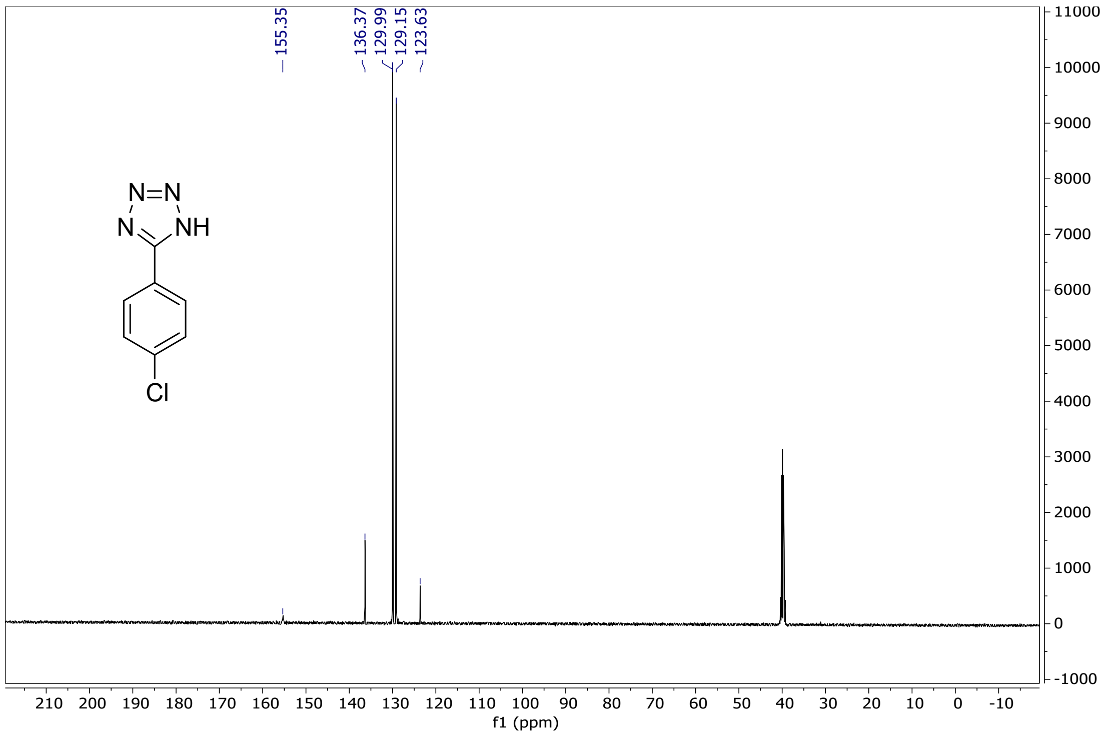

Figure 72. ^{13}C NMR spectrum of 5-(4-chlorophenyl)-1*H*-tetrazole (**24a**) in DMSO-d_6

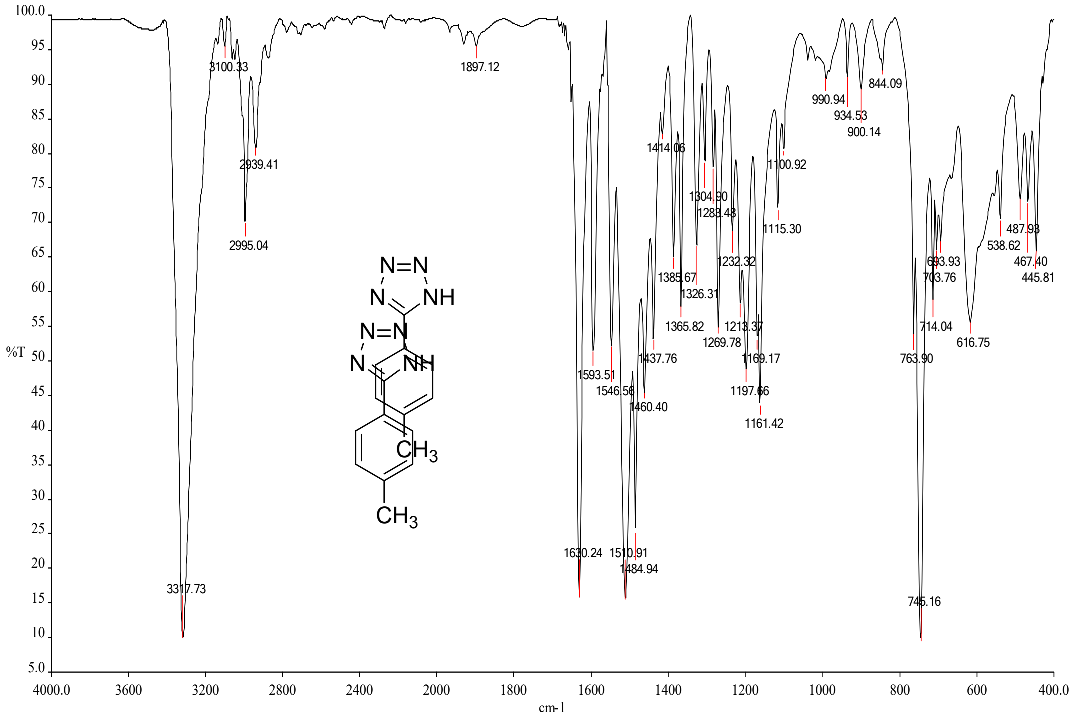

Figure 73. FT-IR spectrum of 5-(p-tolyl)-1*H*-tetrazole (**25a**) in KBr

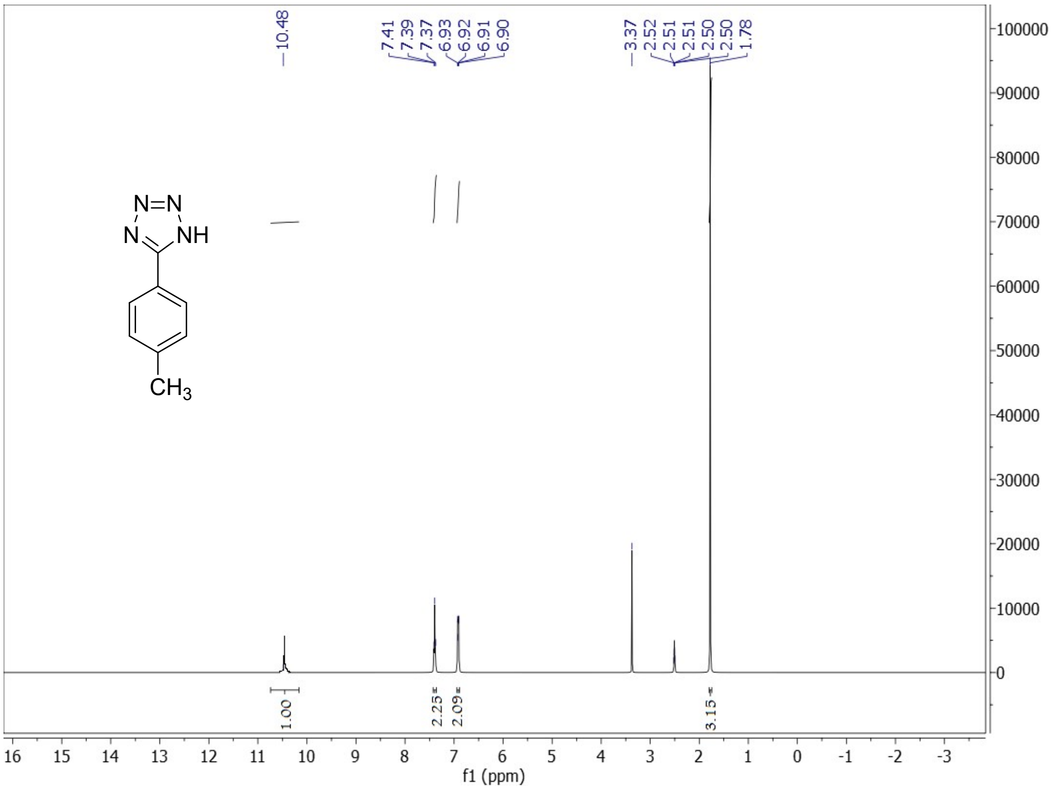

Figure 74. ^{1}H NMR spectrum of 5-(p-tolyl)-1*H*-tetrazole (**25a**) in DMSO-d_6

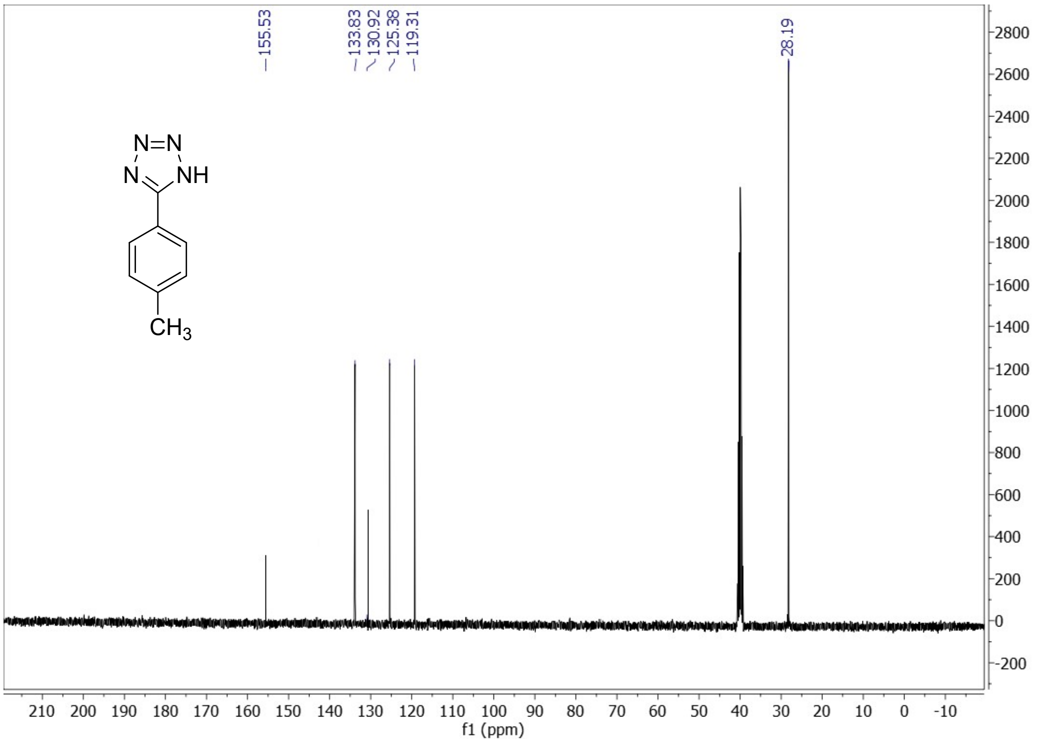

Figure 75. ^{13}C NMR spectrum of 5-(p-tolyl)-1*H*-tetrazole (**25a**) in DMSO-d_6

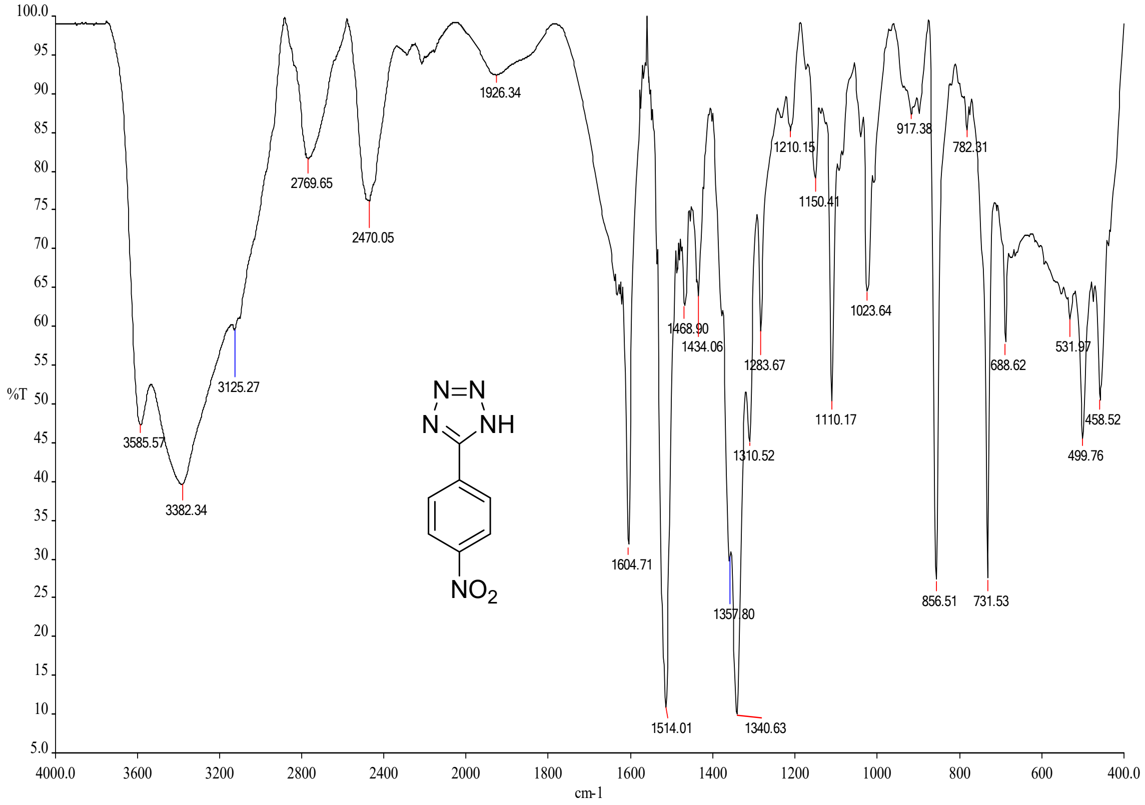

Figure 76. FT-IR spectrum of 5-(4-nitrophenyl)-1*H*-tetrazole (**26a**)in KBr

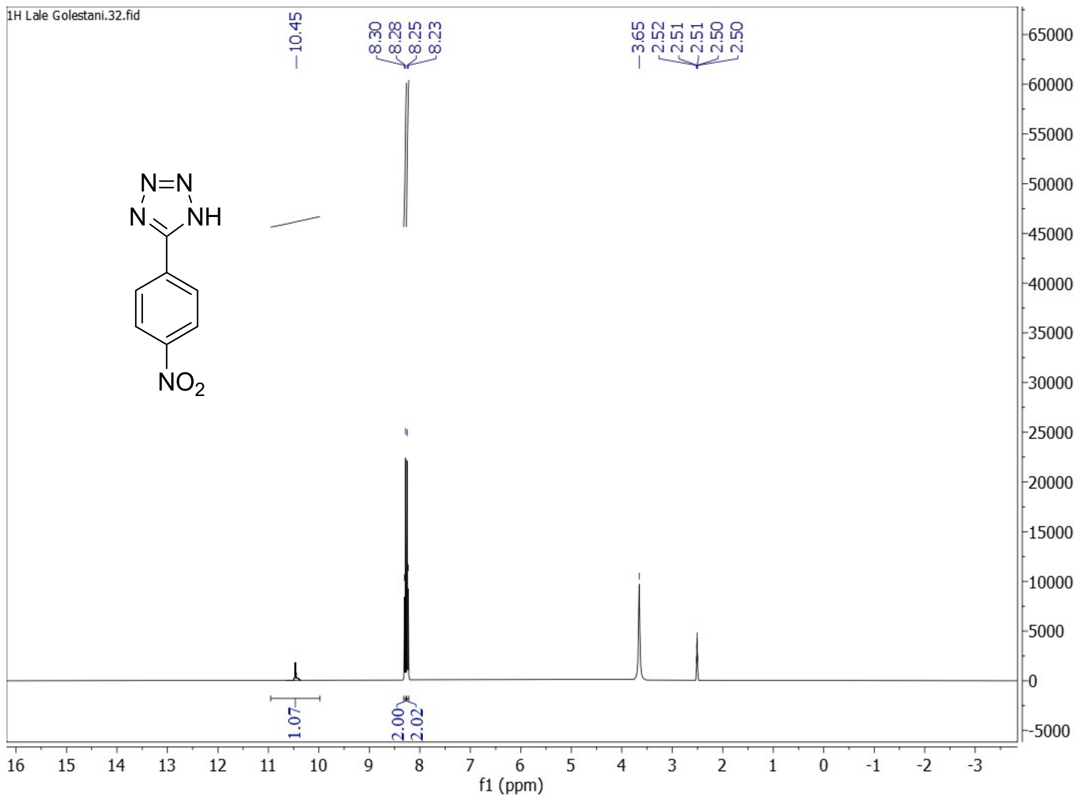

Figure 77. ^{1}H NMR spectrum of 5-(4-nitrophenyl)-1*H*-tetrazole (**26a**) in DMSO-d_6

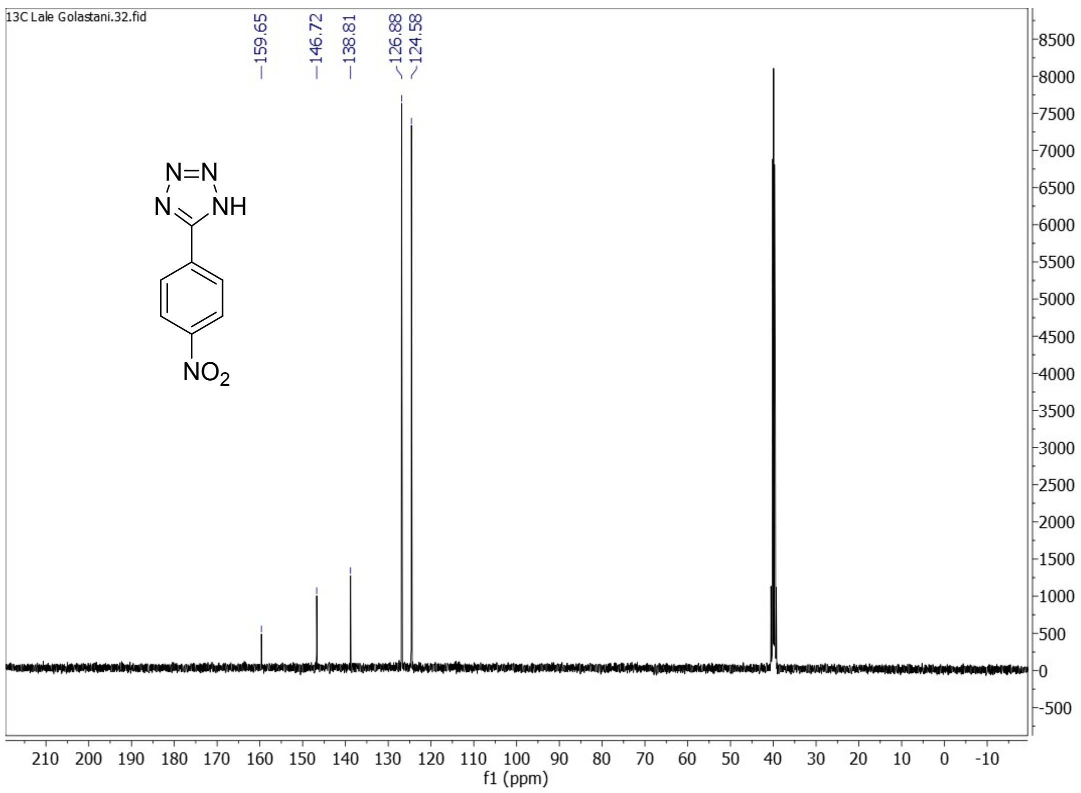

Figure 78. ^{13}C NMR spectrum of 5-(4-nitrophenyl)-1*H*-tetrazole (**26a**) in DMSO-d_6

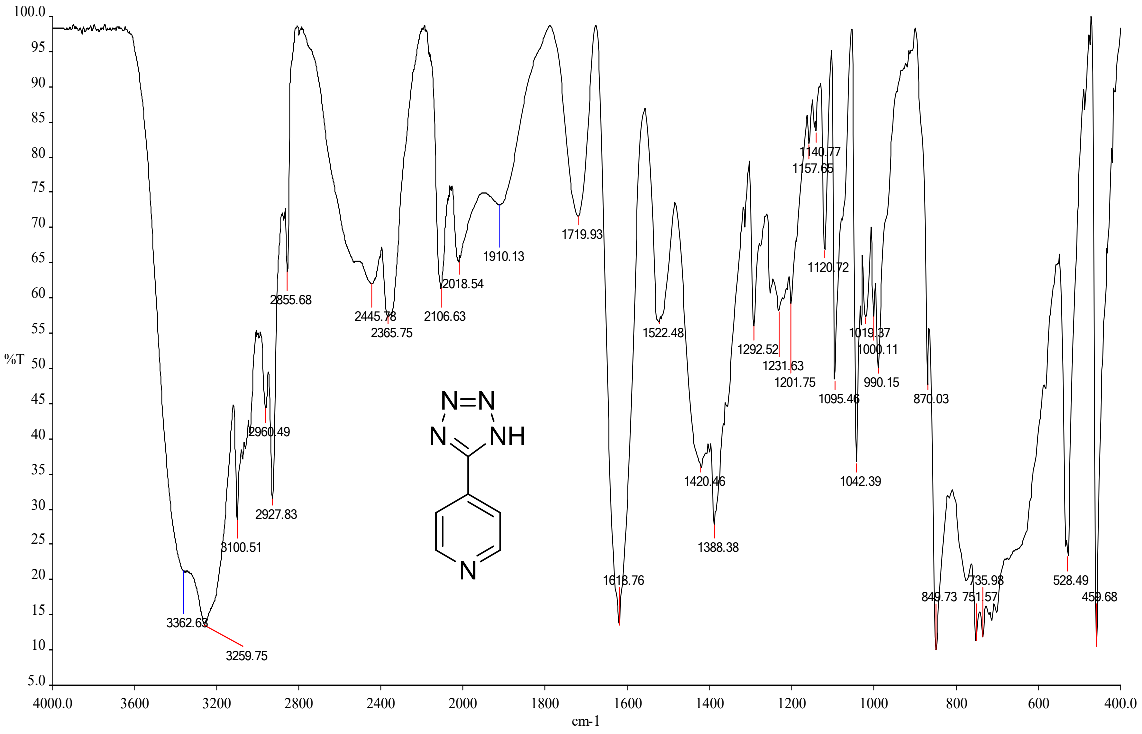

Figure 79. FT-IR spectrum of 4-(1*H*-tetrazol-5-yl)pyridine (**27a**) in KBr

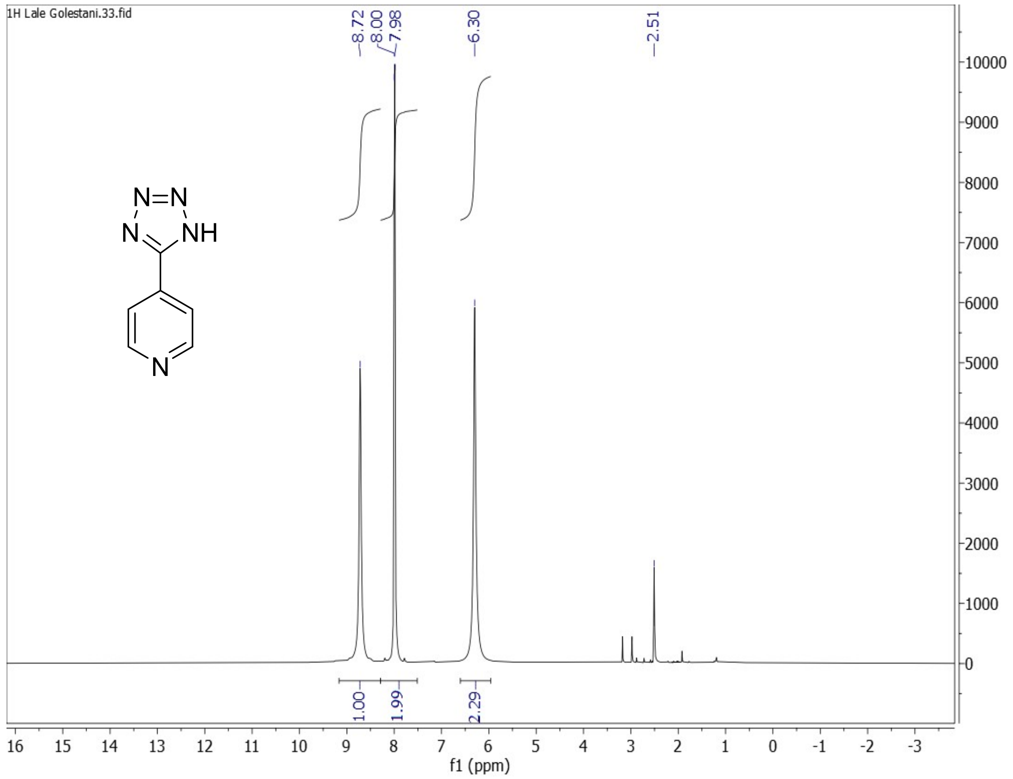

Figure 80. ^{1}H NMR spectrum of 4-(1*H*-tetrazol-5-yl)pyridine (**27a**) in DMSO-d_6

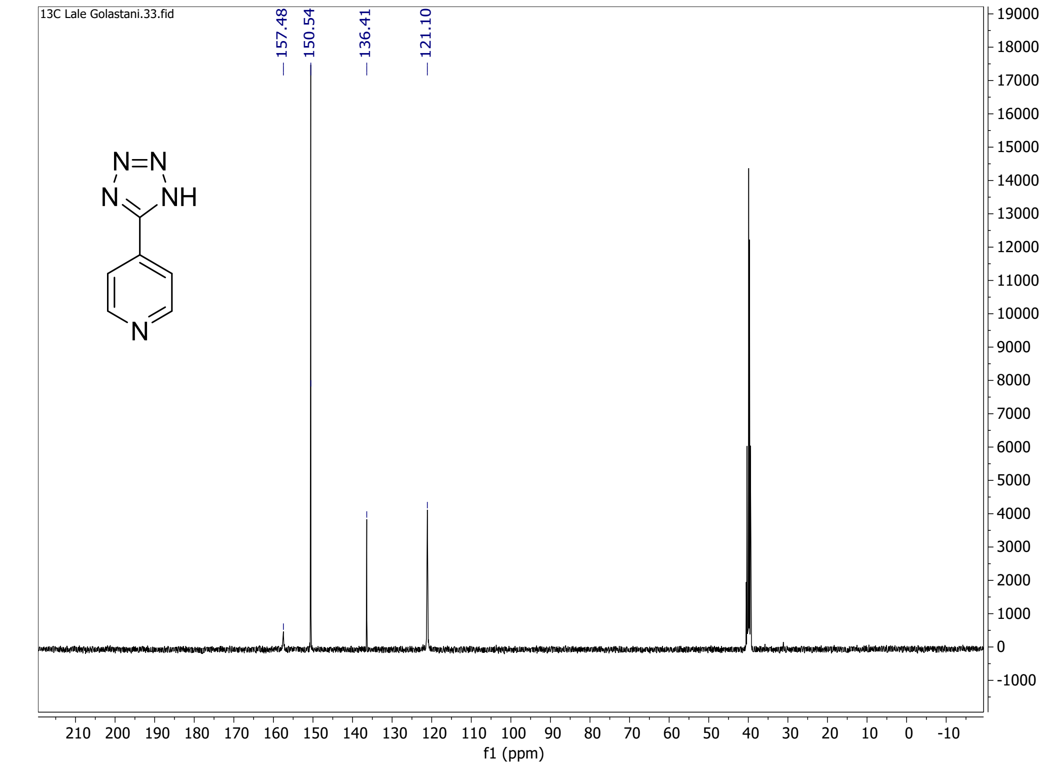

Figure 81. ^{13}C NMR spectrum of 4-(1*H*-tetrazol-5-yl)pyridine (**27a**) in DMSO-d_6

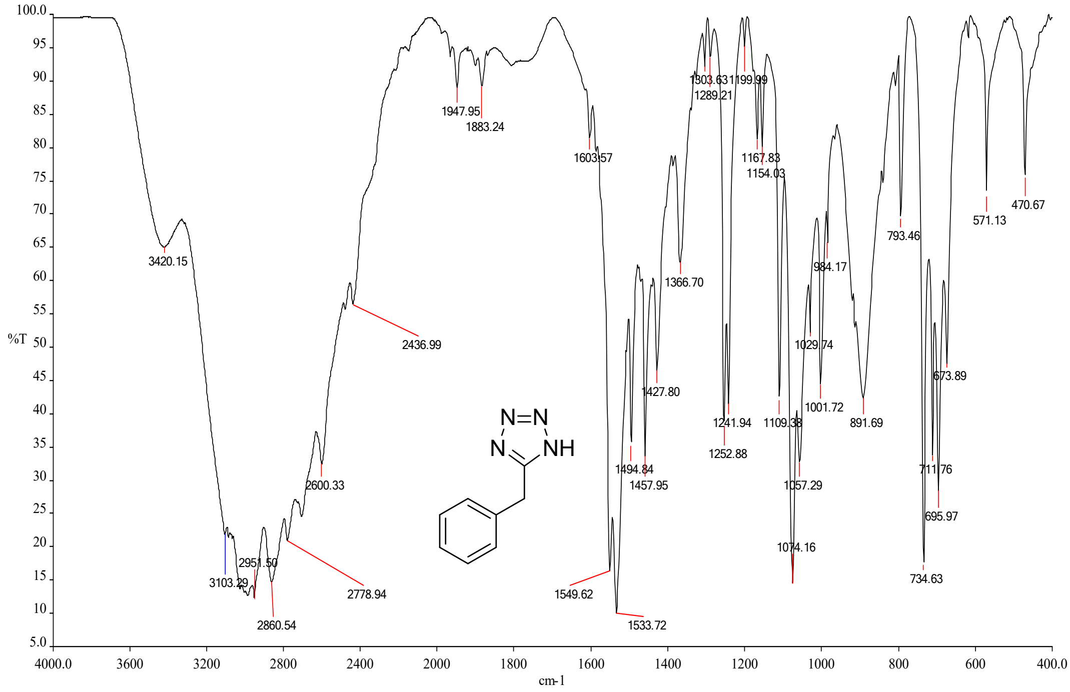

Figure 82. FT-IR spectrum of 5-benzyl-1*H*-tetrazole (**28a**) in KBr

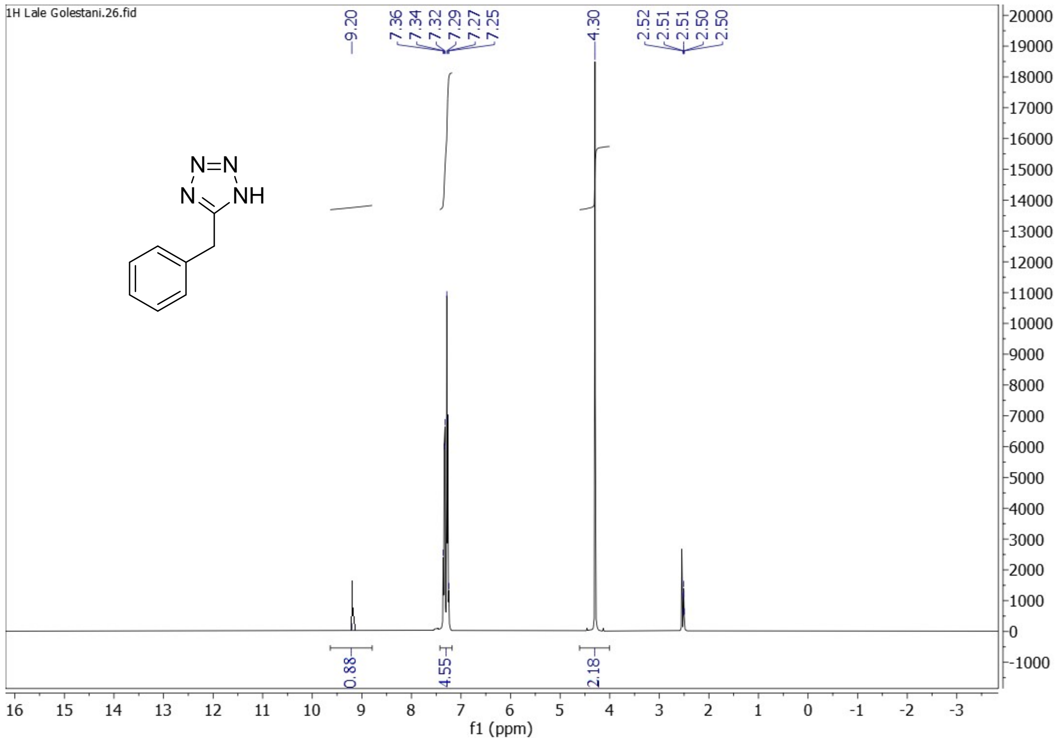

Figure 83. ^{1}H NMR spectrum of 5-benzyl-1*H*-tetrazole (**28a**) in DMSO-d_6

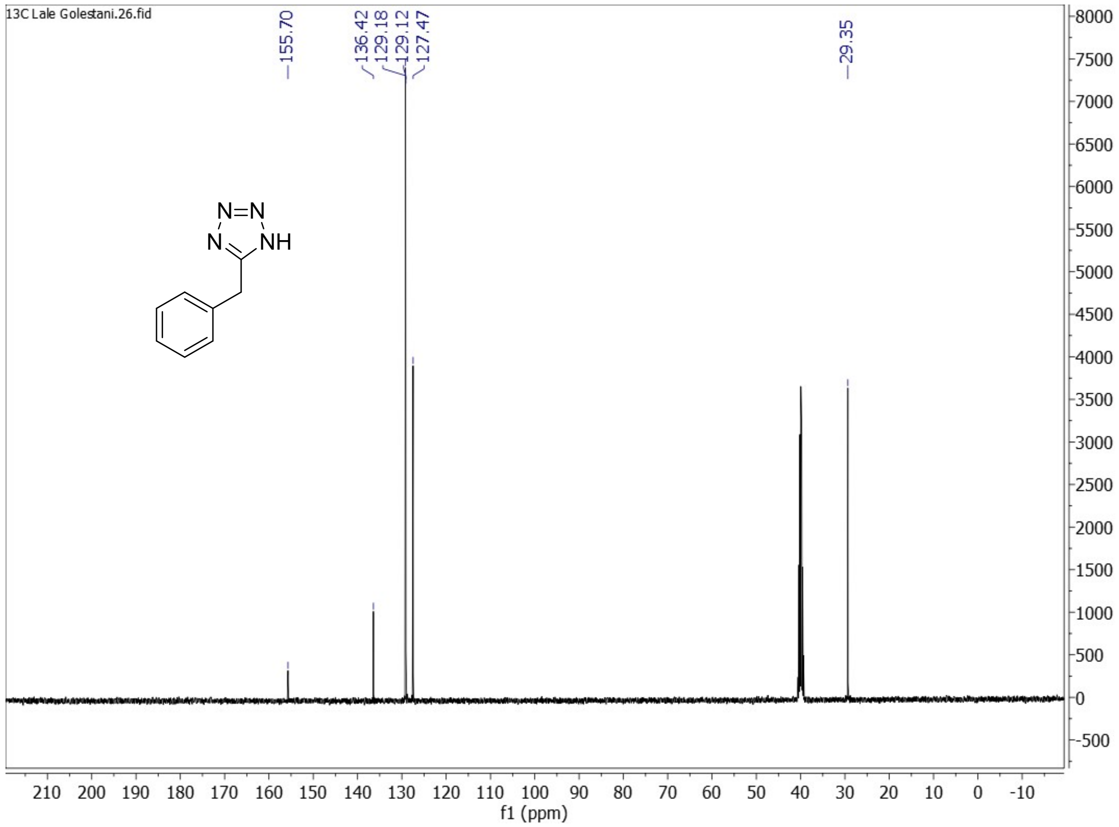

Figure 84. ^{13}C NMR spectrum of 5-benzyl-1*H*-tetrazole (**28a**) in DMSO-d_6

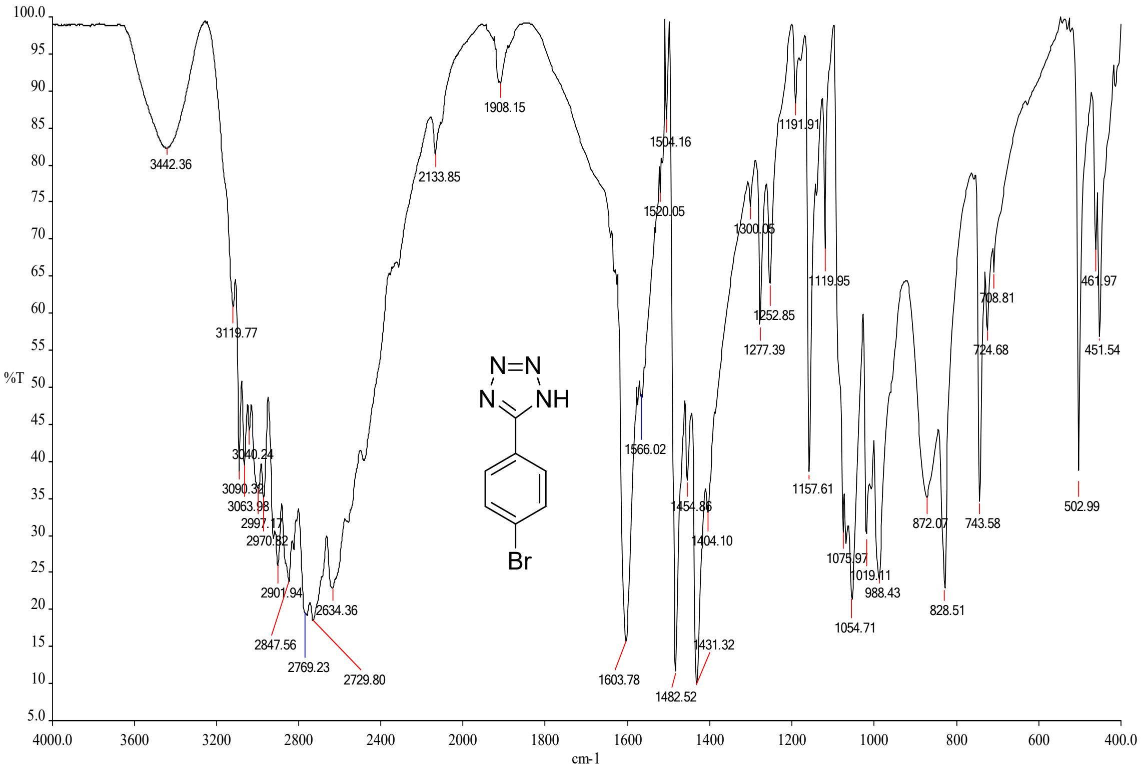

Figure 85. FT-IR spectrum of 5-(4-bromophenyl)-1*H*-tetrazole (**29a**) in KBr

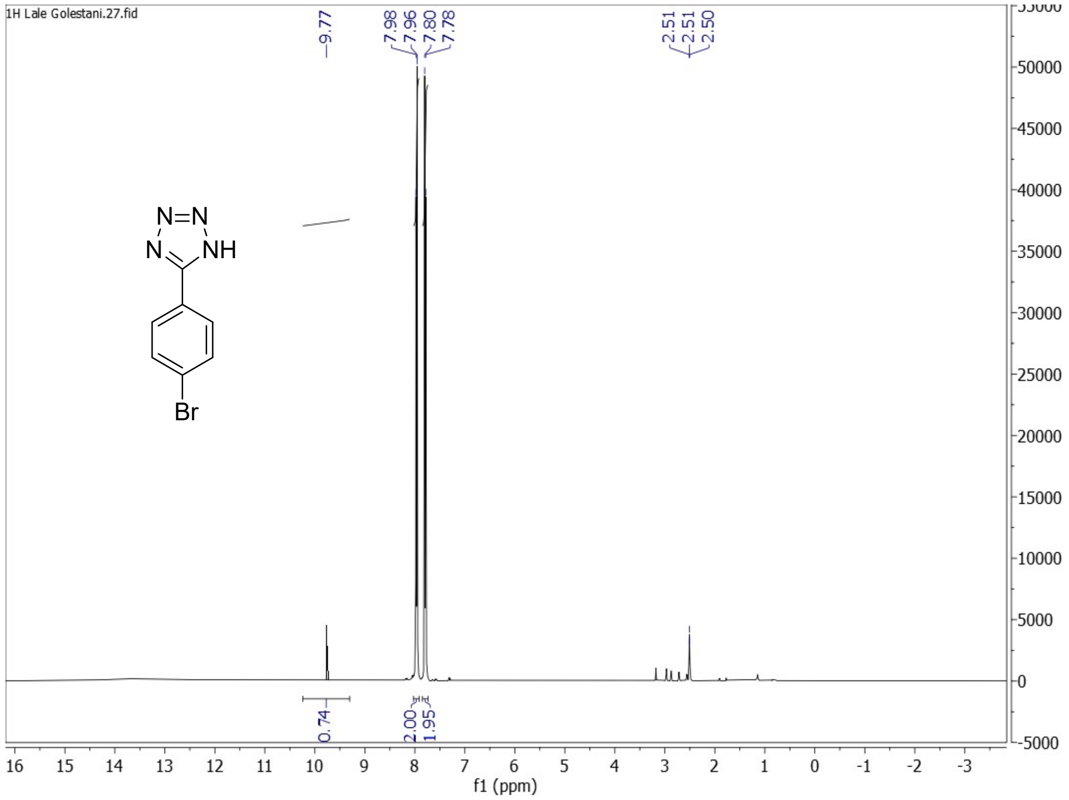

Figure 86. ^{1}H NMR spectrum of 5-(4-bromophenyl)-1*H*-tetrazole (**29a**) in DMSO-d_6

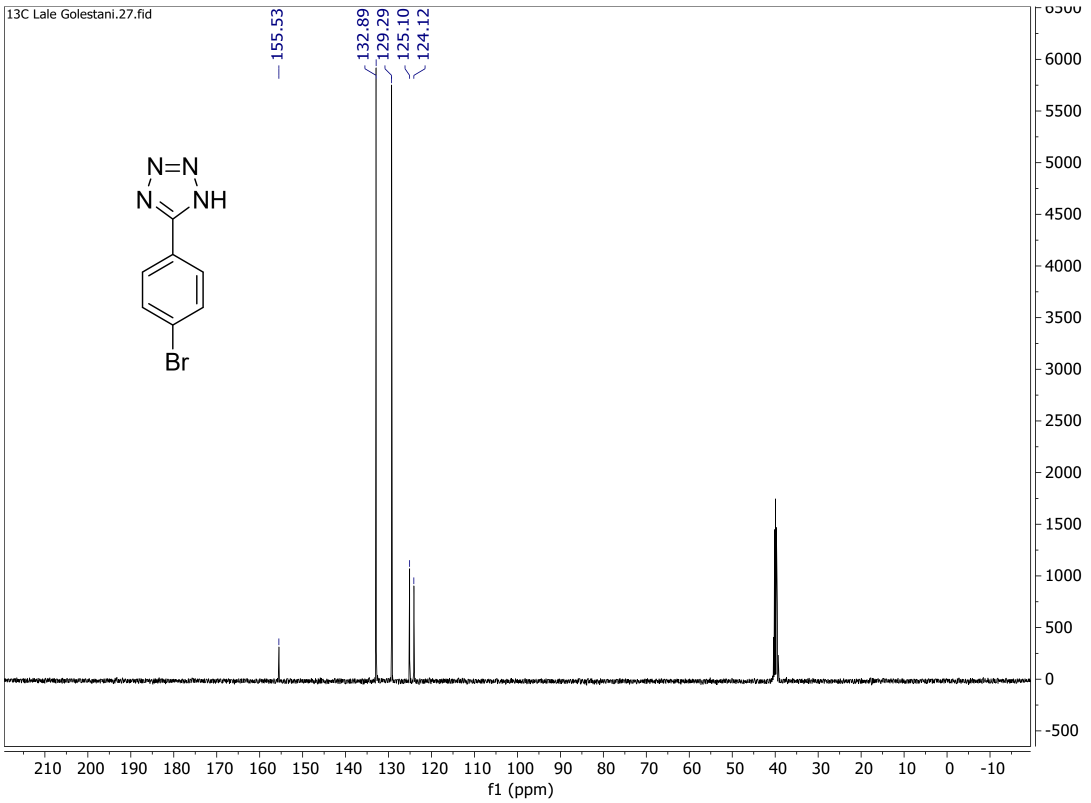

Figure 87. ^{13}C NMR spectrum of 5-(4-bromophenyl)-1*H*-tetrazole (**29a**) in DMSO-d_6

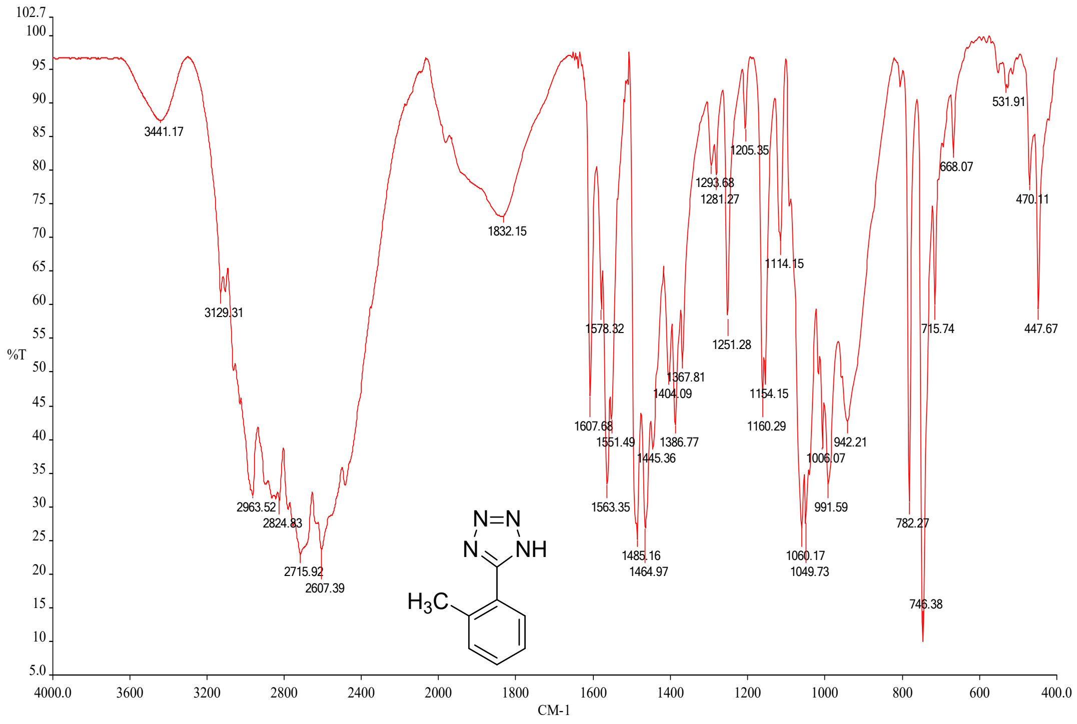

Figure 88. FT-IR spectrum of 5-(o-tolyl)-1*H*-tetrazole (**30a**) in KBr

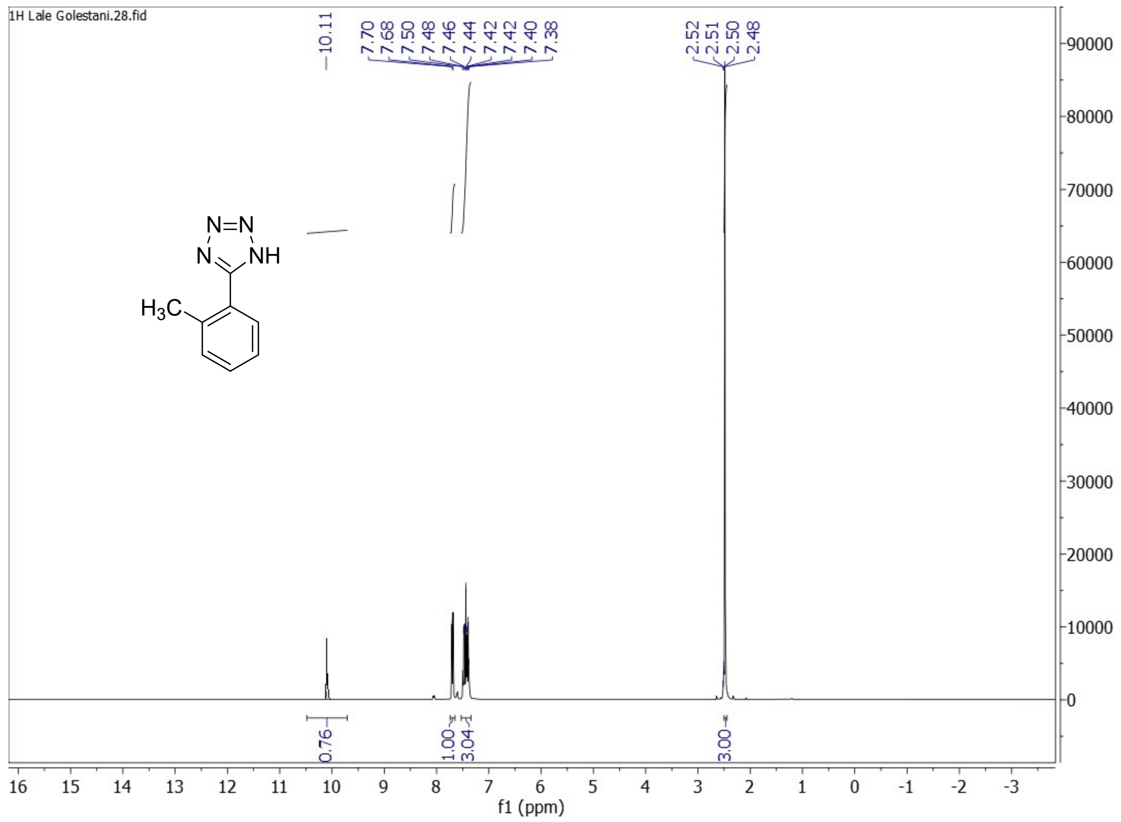

Figure 89. ^{1}H NMR spectrum of 5-(o-tolyl)-1*H*-tetrazole (**30a**) in DMSO-d_6

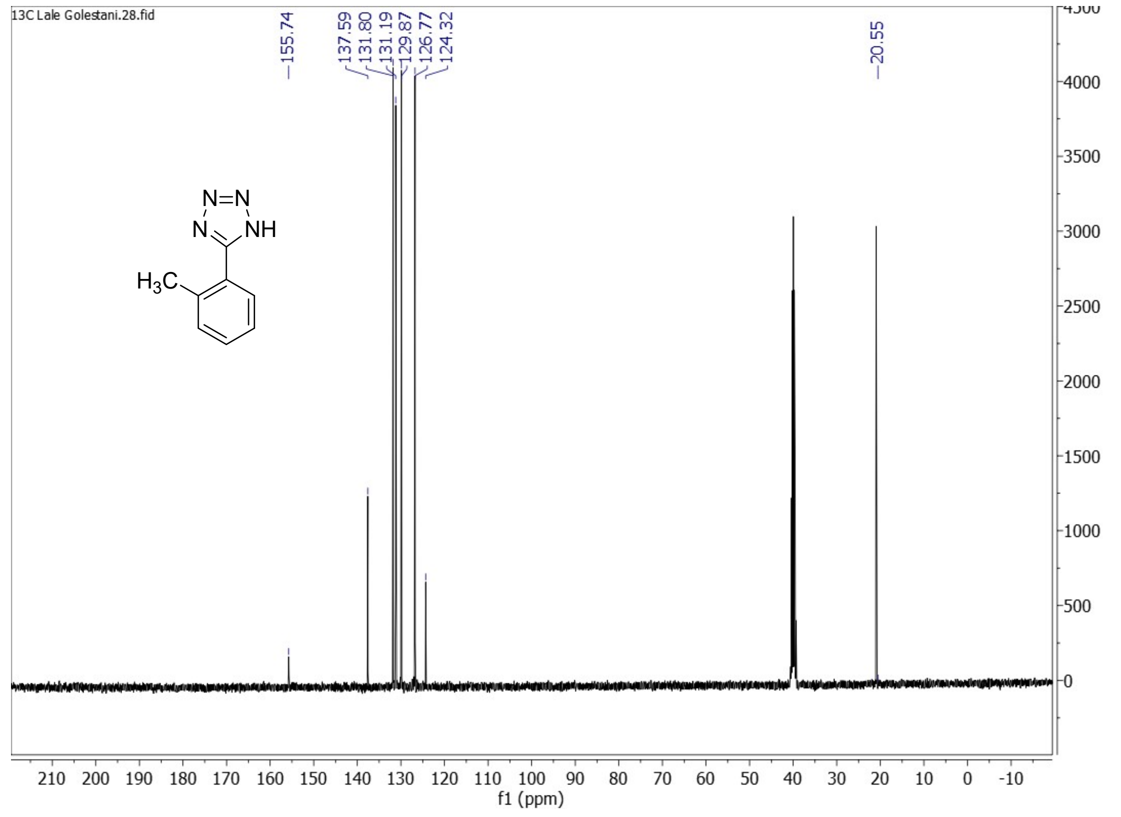

Figure 90. ^{13}C NMR spectrum of 5-(o-tolyl)-1*H*-tetrazole (**30a**) in DMSO-d_6

Reference

37 S. B. Autade, K. G. Akamanchi, K. G. *Synth. Commun.*, 2019, 49, 1947-1956.
40 F. Matloubi Moghaddam, M. Eslami, N. Ghadirian, *Sci. Iran.,* 2019, 26, 1463-1473.
42 H. López-Ruiz, J. E. De La Cerda-Pedro, S. Rojas-Lima, I. Pérez-Pérez, B. V. Rodríguez-Sánchez, R. Santillan, O. Coreño, *Arkivoc.*, 2013, 3, 139-164.
43 A. R. Sardarian, F. Abbasi, M. Esmaeilpour, *Sustain. Chem. Pharm.,* 2023, 36, 101256.
44 D. Khalili, R. Evazi, A. Neshat, J. Aboonajmi, F. Osanlou, *Inorganica Chim. Acta.*, 2020, 506, 119470.
45 R. Sasikala, S. K. Rani, D. Easwaramoorthy, k. Karthikeyan, *RSC Adv.,* 2015, 5, 56507-56517.
46 A. Teimouri, A. N. Chermahini, *Polyhedron*, 2011, 30, 2606-2610.
47 M. Sridhar, K. K. R. Mallu, R. Jillella, K. R. Godala, C. R. Beeram, N. Chinthala, *Synthesis.*, 2013, 45, 507-510.
48 G. Aridoss, K. K. Laali, Eur. J. Org. Chem., 2011, 2011, 6343–6355.
49 V. Rama, K. Kanagaraj, K. Pitchumani, *J. Org. Chem.,* 2011, 76, 9090-9095.
50 B. Sreedhar, A. S. Kumar, D. Yada, *Tetrahedron lett.*, 2011, 52, 3565-3569.
51 D. R. Patil, M. B. Deshmukh, D. S. Dalal, *J. Iran. Chem. Soc.*, 2012, 9, 799-803.
52 Z. Du, C. Si, Y. Li, Y. Wang, J. Lu, *Int. J. Mol. Sci.*, 2012, 13, 4696-4703.
